# Transcriptomic Analysis of Human Astrocytes In Vitro Reveals Hypoxia-Induced Mitochondrial Dysfunction, Modulation of Metabolism, and Dysregulation of the Immune Response

**DOI:** 10.3390/ijms21218028

**Published:** 2020-10-28

**Authors:** Scott P. Allen, Rajpinder Singh Seehra, Paul R. Heath, Benjamin P. C. Hall, Jessica Bates, Claire J. Garwood, Martyna M. Matuszyk, Stephen B. Wharton, Julie E. Simpson

**Affiliations:** Sheffield Institute for Translational Neuroscience, 385a Glossop Road, University of Sheffield, Sheffield S10 2HQ, UK; s.p.allen@sheffield.ac.uk (S.P.A.); rsseehra1@sheffield.ac.uk (R.S.S.); p.heath@sheffield.ac.uk (P.R.H.); bpchall1@sheffield.ac.uk (B.P.C.H.); jessicabates0896@gmail.com (J.B.); c.garwood@sheffield.ac.uk (C.J.G.); mmmatuszyk1@sheffield.ac.uk (M.M.M.); s.wharton@sheffield.ac.uk (S.B.W.)

**Keywords:** astrocyte, hypoxia, immune response, metabolism, mitochondrial dysfunction, transcriptome

## Abstract

Hypoxia is a feature of neurodegenerative diseases, and can both directly and indirectly impact on neuronal function through modulation of glial function. Astrocytes play a key role in regulating homeostasis within the central nervous system, and mediate hypoxia-induced changes in response to reduced oxygen availability. The current study performed a detailed characterization of hypoxia-induced changes in the transcriptomic profile of astrocytes in vitro. Human astrocytes were cultured under normoxic (5% CO_2_, 95% air) or hypoxic conditions (1% O_2_, 5% CO_2_, 94% N_2_) for 24 h, and the gene expression profile assessed by microarray analysis. In response to hypoxia 4904 genes were significantly differentially expressed (1306 upregulated and 3598 downregulated, FC ≥ 2 and *p* ≤ 0.05). Analysis of the significant differentially expressed transcripts identified an increase in immune response pathways, and dysregulation of signalling pathways, including HIF-1 (*p* = 0.002), and metabolism, including glycolysis (*p* = 0.006). To assess whether the hypoxia-induced metabolic gene changes observed affected metabolism at a functional level, both the glycolytic and mitochondrial flux were measured using an XF bioanalyser. In support of the transcriptomic data, under physiological conditions hypoxia significantly reduced mitochondrial respiratory flux (*p* = 0.0001) but increased basal glycolytic flux (*p* = 0.0313). However, when metabolically stressed, hypoxia reduced mitochondrial spare respiratory capacity (*p* = 0.0485) and both glycolytic capacity (*p* = 0.0001) and glycolytic reserve (*p* < 0.0001). In summary, the current findings detail hypoxia-induced changes in the astrocyte transcriptome in vitro, identifying potential targets for modifying the astrocyte response to reduced oxygen availability in pathological conditions associated with ischaemia/hypoxia, including manipulation of mitochondrial function, metabolism, and the immune response.

## 1. Introduction

Sufficient oxygenation of the central nervous system (CNS) is essential to maintain cellular homeostasis and metabolism. However, if the blood supply to the CNS is reduced, levels of available oxygen decrease, resulting in a hypoxic environment. Hypoxia is also implicated in neurodegenerative diseases and their animal models, including Alzheimer’s disease (AD) [1,2,3], amyotrophic lateral sclerosis (ALS) [4,5,6], and Parkinson’s disease (PD) [7,8]. Although the response to hypoxia is initially neuroprotective with mechanisms in place to enable cells to adapt to intermittent low levels of oxygen, extended periods of hypoxia can trigger detrimental events including neuroinflammation and oxidative stress resulting in neuronal dysfunction and ultimately neurodegeneration [9,10].

While hypoxia has a direct impact on neuronal function, reduced oxygen levels also indirectly impact neurones through modulation of glial function. Astrocytes play a key role in regulating homeostasis within the CNS, including providing neuronal support, maintaining the extracellular environment and regulating blood flow to the brain [11], and mediate hypoxia-induced changes in blood–brain barrier permeability, neuroinflammation and neuroprotection against ischaemic injury [12]. Reduced oxygen levels increase expression of the cytokine secretome of rat astrocytes in vitro [13], regulate innate immune responses and reactive oxygen production [14], and induce mitochondrial dysfunction [15]. This suggests that hypoxia plays a role in modulating astrocyte function and contributes to neurodegenerative pathology.

The transcription factor hypoxia inducible factor-1 (HIF-1) comprises a heterodimer consisting of a constitutively expressed β subunit and an oxygen-regulated α subunit, and responds to reduced oxygen availability by coordinating gene expression to adapt to the hypoxic environment. Under normoxic conditions, HIF-1α is rapidly degraded; however, under hypoxic conditions expression of HIF-1α is stabilised and its nuclear translocation enables it to form a heterodimer with HIF-1β. This then binds to hypoxia responsive elements (HREs) to regulate the transcription of a variety of genes, including those involved in physiological and pathophysiological processes such as angiogenesis, remodelling of the extracellular matrix, cytokine production, apoptosis, and mitochondrial respiration [16,17].

Large scale transcriptomic analysis enables the genetic profile of cells/tissue to be characterized in detail. While some studies have investigated the astrocyte response to hypoxia using a hypothesis-driven approach studying specific candidates of interest, to date few studies have investigated the transcriptomic profile of astrocytes in response to hypoxia in vitro. The expression profile of rat astrocytes in response to hypoxia identified significantly differentially expressed genes associated with energy metabolism, survival and lipoprotein binding [18], and the immune response [19], while transcriptomic analysis of primary human astrocytes cultured under hypoxic conditions for 6 h [20] or 24 h [21] primarily identified the significant induction of genes including cell signalling and glycolytic enzymes, respectively.

Gene expression profiling of human astrocytes in response to hypoxia may elucidate pathological processes that can be manipulated through therapeutic intervention to ultimately prevent neurodegeneration. Therefore, the current study performed a detailed characterization of the astrocyte gene expression profile in response to hypoxia in vitro, extending the findings of previous reports and identifying novel candidate gene expression changes.

## 2. Results

### 2.1. Hypoxia Significantly Alters the Astrocyte Transcriptome

**Figure 1:**
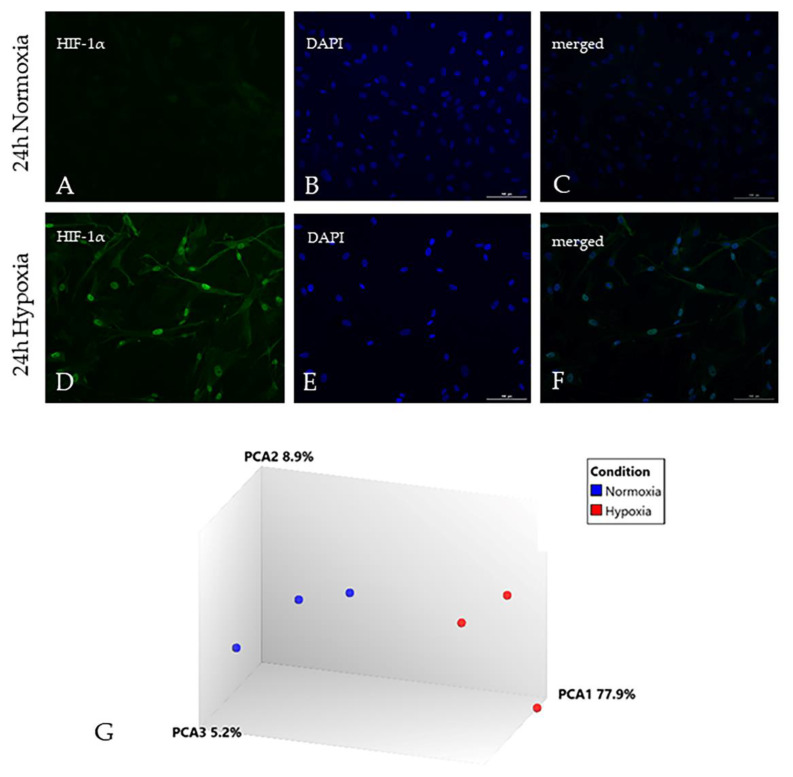
Hypoxia induced changes in the astrocyte gene expression profile. (**A**–**C**) Under normoxic conditions, low levels of hypoxia inducible factor-1α (HIF-1α) are present in the cytoplasm of astrocytes; (**D**–**F**) In response to 24 h hypoxia, HIF-1α translocates to the nucleus of astrocytes; (**G**) Principal component analysis (PCA) of the transcriptomic profile of human astrocytes in response to hypoxia demonstrates clear separation between normoxia (blue) and hypoxia (red) gene expression profiles. Scale bar represents 100 µm (**A**–**F**).

Nuclear translocation of the transcription factor HIF-1α was observed in astrocytes in response to hypoxia for 24 h (Figure 1A–F). All extracted RNA profiles from human primary astrocytes showed distinct 18 s and 28 s ribosomal peaks by picochip analysis and had RNA Integrity Numbers (RIN) greater than 8.5. Hypoxia-induced changes in the transcriptomic profile of human astrocytes were generated using Human Genome U133 Plus 2.0 Arrays, which comprise 1.3 × 10^6^ unique oligonucleotide sequences, including >47,000 transcripts and variants of 33,000 genes. Between 46% and 60% of the probe set sequences were present across all samples (mean [range]: normoxic 59.5% [59.2–59.8%]; hypoxic 50.5% [46.3–53.2%]). No sample outliers were identified by visual inspection of hierarchical clustering and principal component analysis, which also showed clear separation of hypoxic and non-hypoxic samples (Figure 1G). In response to hypoxia 4904 genes were significantly differentially expressed (1306 upregulated (Supplementary Table S1) and 3598 downregulated (Supplementary Table S2), FC ≥ 2 and *p* ≤ 0.05). The expression dataset is freely available at Gene Expression Omnibus, accession number GSE145935. To confirm a robust response to hypoxia was induced, the dataset was interrogated to identify a comprehensive panel of published hypoxia-inducible genes [22,23,24], as shown in Table 1.

The data was initially analysed using the Database for Annotation Visualisation and Integrated Discovery (DAVID) to identify Kyoto Encyclopedia of Genes and Genomes (KEGG) pathways and functional groups of differentially expressed transcripts. Analysis of all significantly upregulated genes identified signalling pathways including HIF-1 (*p* = 0.002) and PI3K-Akt (*p* = 0.005), metabolism (glycolysis *p* = 0.006; fructose and mannose metabolism *p* = 0.04), insulin resistance (*p* = 0.01), and an increased immune response (*Staphylococcus aureus* infection *p* = 0.007; HTLV-I infection *p* = 0.02; Intestinal immune network for IgA production *p* = 0.05) (Table 2).

Analysis of all significantly downregulated genes identified dysregulation of pathways including metabolism (purine metabolism *p* = 0.02; pyrimidine metabolism *p* = 0.03, tricarboxylic acid cycle (TCA) *p* = 0.02), ubiquitin-mediated proteolysis (*p* < 0.001), and cell signalling (Wnt signalling *p* = 0.002; p53 signalling *p* = 0.02) (Table 3). Subsequent detailed interrogation of the data focused on hypoxic modulation of metabolism and the immune response.

### 2.2. Hypoxia Modulates the Metabolic Profile of Astrocytes

**Figure 2:**
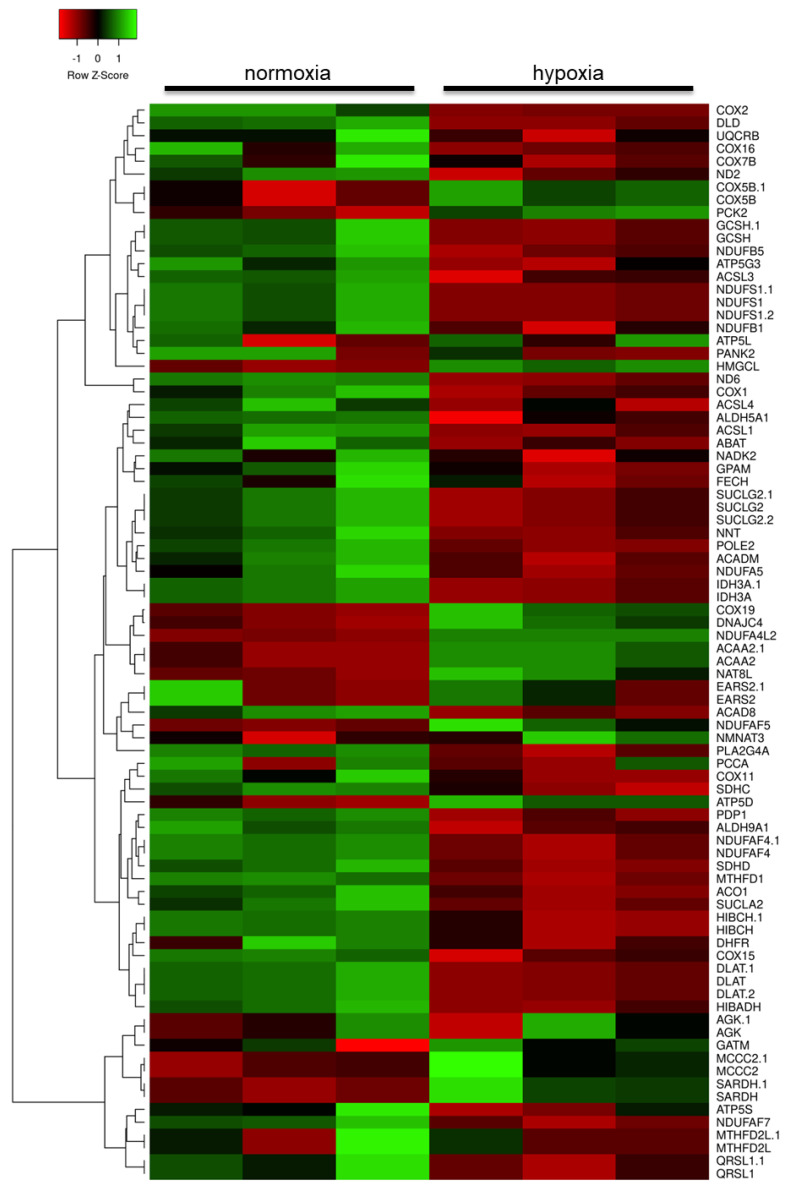
Heat map of mitochondrial and metabolic gene expression changes in human astrocytes in response to hypoxia. Red: downregulated transcripts; green: upregulated transcripts.

Insulin and insulin-like growth factor (IGF) primarily signal through phosphatidylinositol 3-kinase and protein kinase B (PI3K/Akt) and mitogen-activated protein kinase (MAPK) pathways to regulate cell growth, survival and metabolism [25]. Genes encoding insulin receptors (IR) and IGF receptors (IGFR), as well as their receptor substrates (IRS), were significantly differentially expressed in human astrocytes in response to hypoxia. Analysis of hypoxia-induced changes in the astrocyte transcriptome also identified the significant dysregulation of mitochondrial-associated genes and genes associated with metabolism (Figure 2). Transcripts associated with mitochondrial function were primarily significantly downregulated, including: nicotinamide adenine dinucleotide plus hydrogen (NADH) dehydrogenase subunit 3 (complex I) (*ND3* probe set id: 1553588_at, FC = −2.5, *p* = 0.003) and NADH dehydrogenase (ubiquinone) complex I assembly factor 4 (*NDUFAF4.1* probe set id: 219006_at, FC = −2.5, *p* < 0.001; probe set id: 227559_at, FC = −2.5, *p* = 0.004) which are involved in electron transport chain complex 1 assembly and function; the TCA cycle associated synthetase succinate-CoA ligase GDP-forming beta subunit (*SUCLG2* probeset id: 214835_s_at, FC = −2.9, *p* = 0.014; probeset id: 215772_x_at, FC = −3.0, *p* = 0.014; probeset id: 212459_x_at, FC = −3.6, *p* = 0.007); and the adenosine triphosphate (ATP) synthase subunit ATP synthase, H+ transporting, mitochondrial Fo complex subunit C3 (subunit 9) (*ATP5G3* probeset id: 207507_s_at, FC = −2.0, *p* = 0.025). Mitochondria and the endoplasmic reticulum (ER) interact with each other and play a key role in a number of critical cellular functions, including mitochondrial dynamics and bioenergetics [26]. Transcripts encoding structural components of ER-mitochondria contact sites were dysregulated in response to hypoxia, including mitofusin 1 (*MFN1* probeset id: 207098_s_at, FC = −2.1, *p* = 0.025; probeset id: 217043_s_at, FC = −1.8, *p* = 0.048)

Conversely, genes associated with glycolytic function were significantly upregulated, including: phosphoglycerate kinase 1 (*PGK1* probeset id: 200737_at, FC = 2.3, *p* < 0.001), pyruvate kinase (*PKM* probeset id: 213700_s_at, FC = 2.8, *p* = 0.008), and pyruvate dehydrogenase kinase, isozyme 3 (*PDK3* probeset id: 228959_at, FC = 4.2, *p* < 0.001; probeset id: 206348_s_at, FC = 3.2, *p* < 0.001; probeset id: 230085_at, FC = 2.7, *p* < 0.001), indicating a switch to a more anaerobic metabolic profile to meet bioenergetic demands under hypoxia.

### 2.3. Hypoxia Modulates the Astrocyte Immune Response

**Figure 3:**
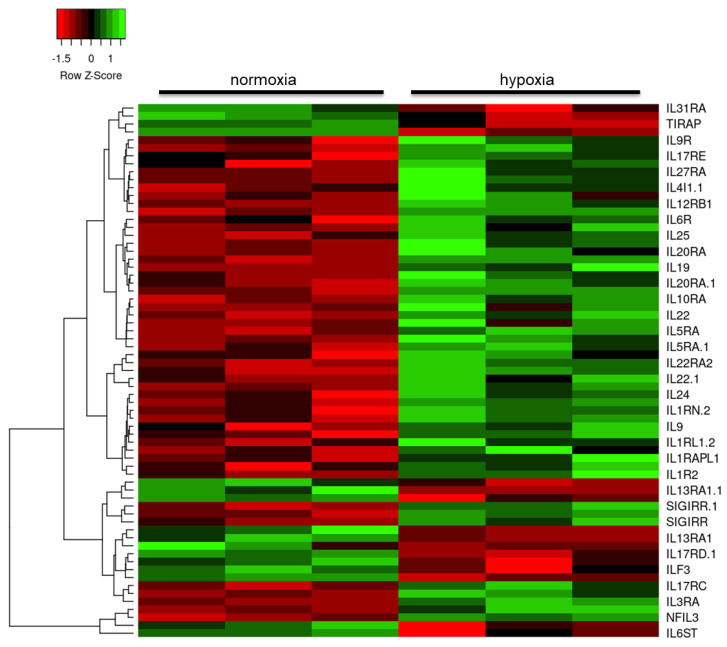
Heat map of interleukin gene expression changes in human astrocytes in response to hypoxia. Red: downregulated transcripts; green: upregulated transcripts.

**Figure 4:**
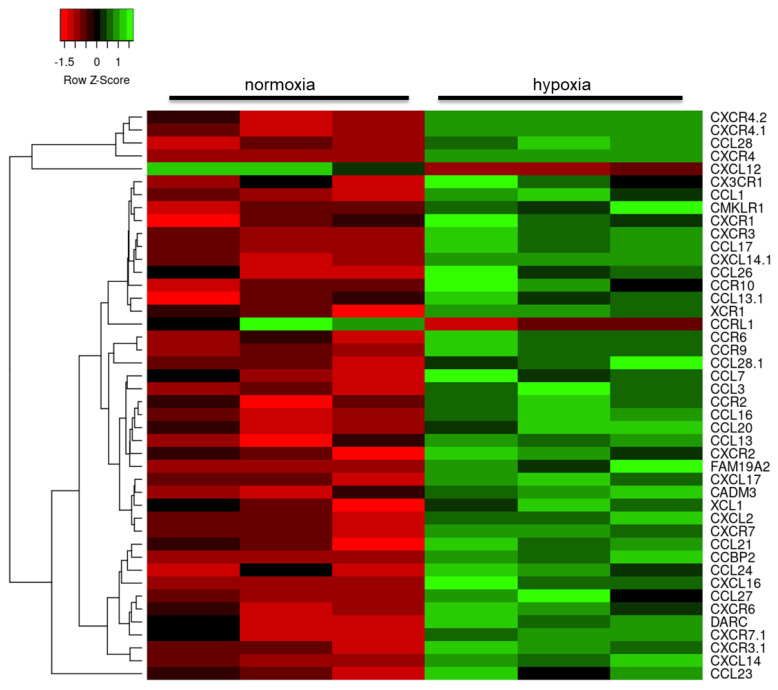
Heat map of chemokine gene expression changes in human astrocytes in response to hypoxia. Red: downregulated transcripts; green: upregulated transcripts.

Further interrogation of the datasets reducing the stringency to FC > 1.2 and focusing on the immune response identified dysregulation in the expression of cytokines and their receptors, predominantly upregulation of the interleukin (IL) family of cytokines (Figure 3), including *IL34* (probeset id: 237046_x_at, FC = 2.1, *p* = 0.001), and chemoattractant cytokines (Figure 4), including several highly upregulated transcripts for chemokine receptor 4 (*CXCR4* probeset id: 217028_at, FC = 4.2, *p* < 0.001; probeset id: 209201_x_at, FC = 4.0, *p* < 0.001; probeset id: 211919_s_at, FC = 3.6, *p* = 0.003).

**Figure 5:**
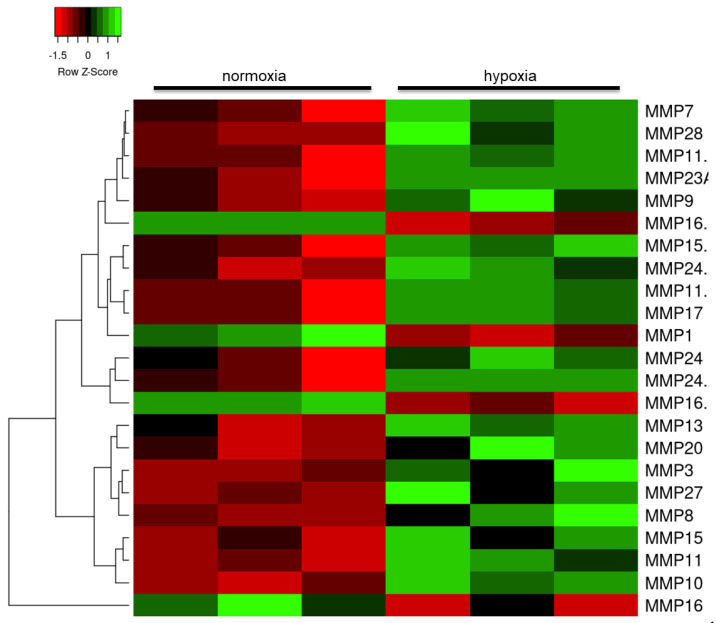
Heat map of matrix metalloproteinase (MMP) gene expression changes in human astrocytes in response to hypoxia. Red: downregulated transcripts; green: upregulated transcripts.

Matrix metalloproteinases (MMP) are involved in remodeling of the extracellular matrix and are upregulated as part of the neuroinflammatory response [27]. Hypoxia significantly altered the transcription of several members of these proteases (Figure 5), including *MMP10* (probeset id: 205680_at, FC = 2.0, *p* = 0.004), as well as tissue inhibitor of metalloproteinase (TIMP) and a disintegrin and metalloproteinase (ADAM) expression.

### 2.4. Validation of Candidate Gene Expression Changes

**Figure 6:**
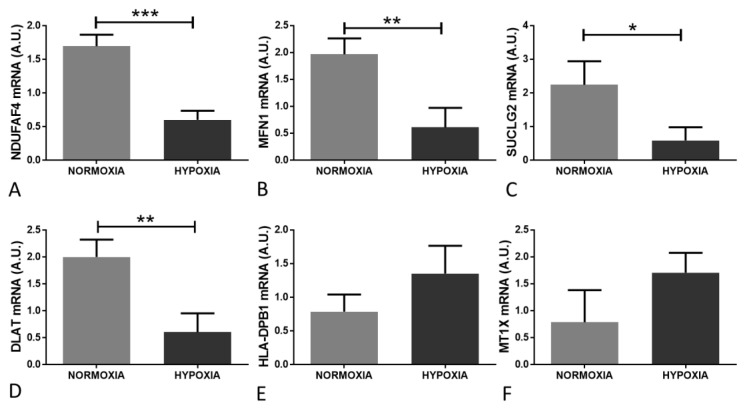
qPCR validation of hypoxia induced gene expression changes in human astrocytes. qPCR confirmed a significant downregulation of (**A**) NDUFAF4 (*p* = 0.001), (**B**) MFN1 (*p* = 0.007), (**C**) SUCLG2 (*p* = 0.02), (**D**) DLAT (*p* = 0.007); and a non-significant upregulation of (**E**) HLA-DPB1 (*p* = 0.11) and (**F**) MT1X (*p* = 0.08) in response to hypoxia. * *p* ≤ 0.05, ** *p* ≤ 0.01, *** *p* ≤ 0.001.

The altered expression of candidate genes was validated by qPCR on RNA extracted from astrocytes in response to hypoxia, compared to normoxia, in additional independent experiments. qPCR confirmed a significant downregulation of NDUFAF4 (Figure 6A, *p* = 0.001; microarray probe set id: 219006_at, FC = −2.5, *p* < 0.001), MFN1 (Figure 6B, *p* = 0.007; microarray probeset id: 207098_s_at, FC = −2.1, *p* = 0.025), SUCLG2 (Figure 6C, *p* = 0.02; microarray probeset id: 212459_x_at, FC = −3.6, *p* = 0.007), dihydrolipoamide S-acetyltransferase (DLAT Figure 6D, *p* = 0.007; microarray 211150_s_at, FC = −2.9, *p* < 0.001) in response to hypoxia. While the changes were non-significant, the directional changes observed confirmed the increased expression of major histocompatibility complex, class II, DPβ1 (HLA-DPB1 Figure 6E, *p* = 0.11; microarray probe set id 215193_x_at, FC = 2.6, *p* = 0.029), and metallothionein 1X (MT1X Figure 6C, *p* = 0.08; microarray probe set id 208581_x_at, FC = 2.1, *p* = 0.041) in response to hypoxia.

### 2.5. Functional Validation of Hypoxia-Induced Changes in Astrocyte Metabolism

**Figure 7:**
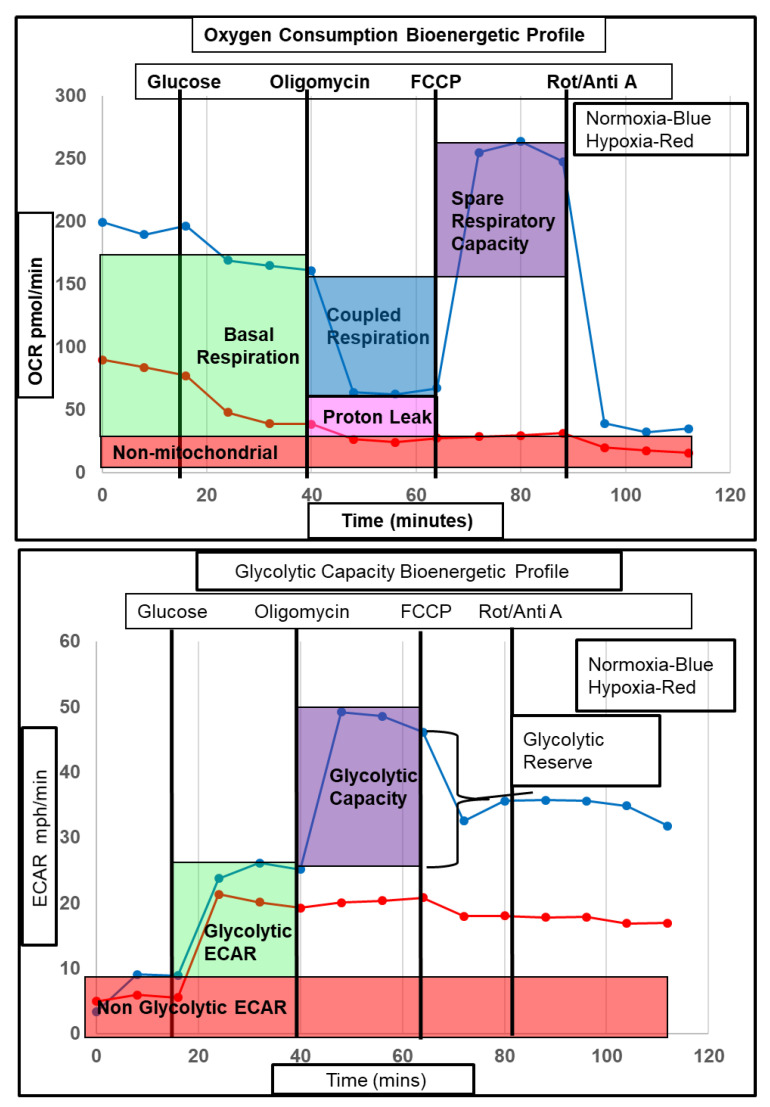
Representative bioenergetic profile of astrocyte mitochondrial oxygen consumption and glycolytic flux during hypoxia. (**Upper panel**) Representative astrocyte mitochondrial oxygen consumption bioenergetic profile, showing the response of astrocyte oxygen consumption to injection of glucose, oligomycin, carbonyl cyanide p-trifluoromethoxy phenylhydrazone (FCCP), and rotenone/antimycin A. Blue line = normoxia, red line = hypoxia; (**Lower panel**) Representative astrocyte glycolytic flux bioenergetic profile showing the response of astrocyte glycolytic flux to injection of glucose, oligomycin, FCCP and rotenone/ antimycin A. Blue line = normoxia, red line = hypoxia. OCR = oxygen consumption rate, ECAR = extracellular acidification rate.

To assess whether the hypoxia induced metabolic gene changes observed affected metabolism at a functional level, astrocytes were incubated under hypoxic conditions and both glycolytic and mitochondrial flux were measured. To assess the response to stress under hypoxia, mitochondrial and glycolytic flux were assessed under physiological conditions and under stress. Injection of oligomycin and carbonyl cyanide p-trifluoromethoxy phenylhydrazone (FCCP) onto human astrocytes to inhibit the mitochondrial ATP synthase and uncouple the electron transport chain (ETC) from ATP production respectively impacted both the metabolic mitochondrial and glycolytic stress response under hypoxic conditions (Figure 7).

**Figure 8:**
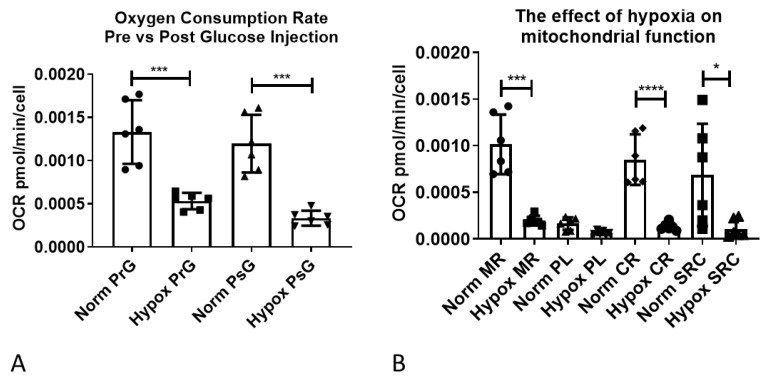

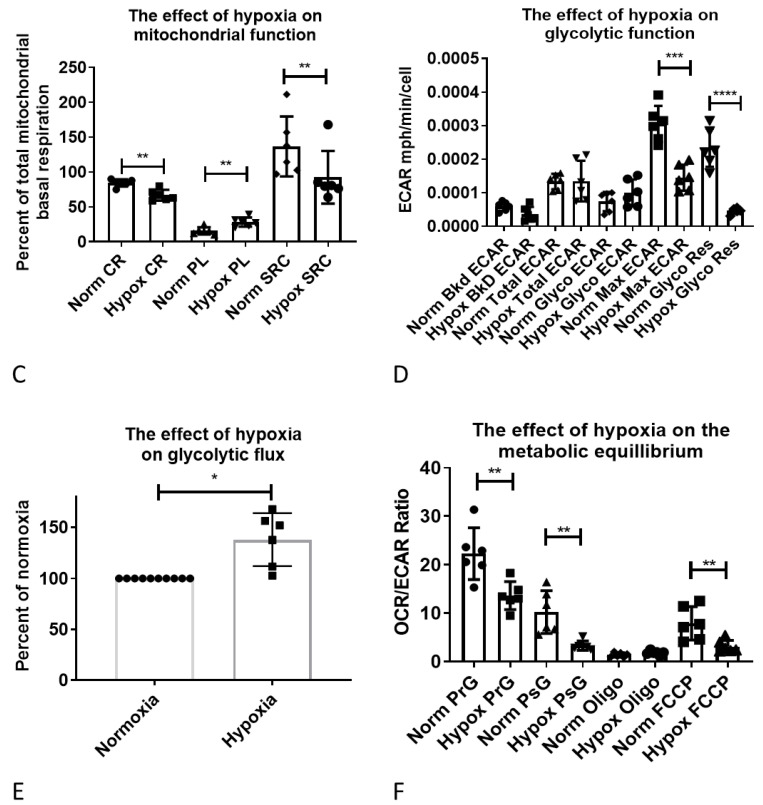
Hypoxia reduces mitochondrial respiratory function and glycolytic capacity under stress. (**A**) Oxygen consumption rate in astrocytes under normoxia and hypoxia; (**B**) The effect of hypoxia on mitochondrial function; (**C**) The effect of hypoxia on mitochondrial function as a percentage of mitochondrial respiration; (**D**) The effect of hypoxia on glycolytic function; (**E**) The effect of hypoxia on glycolytic flux as a percentage of normoxia; (**F**) The effect of hypoxia on astrocyte metabolic equilibrium. Data presented as mean with standard deviation, each shape on the graph represents one biological replicate, which was an average of 9–10 wells of a 24-well plate. All assays were performed in triplicate on two independent astrocyte cell lines. Prior to statistical analysis all percentage data was transformed 1=1/Y and 1=logit(Y) prior to analysis and all data underwent distribution analysis. Subsequently an unpaired *t*-test or Mann–Whitney test was performed except for panel (**E**) where a Wilcoxon paired rank analysis was performed. * *p* ≤ 0.05, ** *p* ≤ 0.01, *** *p* ≤ 0.001, **** *p* ≤ 0.0001. OCR = oxygen consumption rate, ECAR = extracellular acidification rate, PrG = pre glucose, PsG = post glucose, norm = normoxia, Hypox = hypoxia, MR = mitochondrial respiration, PL = proton leak, CR = coupled respiration, SRC = spare respiratory capacity, NG = non-glycolytic, Glyco = glycolytic, Cap = capacity, Res = reserve, Oligo = oligomycin.

Astrocytes were initially starved of glucose to measure non glycolytic extracellular acidification rate (ECAR, predominantly a measure of bicarbonated CO_2_ produced by the TCA cycle) and therefore calculate glycolytic specific ECAR. Oxygen consumption rate (OCR) under starvation conditions was significantly lower in the hypoxia treated astrocytes (82.6% reduction, *p* = 0.0004), as shown in Figure 8A (normoxia pre-glucose versus hypoxia pre-glucose). After injection of glucose a similar significant reduction was observed (72%, *p* = 0.001), as shown in Figure 8A (normoxia post-glucose versus hypoxia post-glucose). Mitochondrial respiratory (MR) flux was significantly lower in hypoxic astrocytes (80.7% reduction, *p* = 0.001) as shown in Figure 8B (normoxia MR versus hypoxia MR), and as expected, was coupled to respiration ATP-linked flux (CR), proton leak linked flux (PL), and mitochondrial spare respiratory capacity (SRC) linked flux (Figure 8B). As CR, PL, and SRC flux levels are intrinsically linked to the overall level of MR flux, we analysed CR, PL, and SRC as a percentage of MR, and detected a significant reduction in the percentage of CR in hypoxic astrocytes (84.3 to 66.7% *p* < 0.0011) and an increase in the percentage of PR (15.7 to 28.2%, *p* = 0.0031) (Figure 8C). Therefore, although both CR and PL flux rates were reduced in astrocytes under hypoxia the remaining MR was less coupled than in normoxic astrocytes. Similarly, hypoxia significantly reduced SRC as a percentage of MR (135.5 to 92.2% *p* = 0.0485), indicating a reduced mitochondrial capacity in hypoxia under stress (Figure 8C). When we assessed glycolytic ECAR flux, non-glycolytic flux (NG-ECAR) was reduced in hypoxia treated astrocytes but just failed to reach significance (*p* = 0.0502, unpaired t-test) (Figure 8D). Total ECAR however, was unaltered due to a 38% increase in glycolytic specific ECAR, which failed to reach significance when analysing raw data, but reached significance when assessing percentage increase compared to normoxia (*p* = 0.0313) (Figure 8D,E). Interestingly, glycolytic capacity and glycolytic reserve were both significantly reduced in hypoxic astrocytes (52%, *p* = 0.001 and 90.7% *p* < 0.0001, respectively) (Figure 8D). As the XF bioanalyser measures mitochondrial and glycolytic flux simultaneously, the metabolic equilibrium (the balance between aerobic and non-aerobic metabolism) can be assessed by calculating the OCR/ECAR ratio [28]. The higher the ratio, the more aerobic the metabolic phenotype of the cell. Under conditions of starvation (PrG, *p* = 0.0057), glucose (PsG, *p* = 0.0022), and stress (FCCP, *p* = 0.0096), the OCR/ECAR ratio was significantly lower in hypoxic astrocytes, indicating a more anaerobic metabolic state (Figure 8F).

## 3. Discussion

Hypoxia and an impaired response to hypoxia are implicated in the pathogenesis of several neurodegenerative diseases. Dysregulation of both HIF-1α expression and the downstream pathways in response to hypoxia induces motor neuron degeneration in ALS [6,29,30], and stimulates the production of β-amyloid peptides and the accumulation of amyloid plaques in AD [3,31]. While reduced oxygen availability directly impairs neuronal function, it can also indirectly impact neurones through modulation of glial function. Astrocytes play a key role in neuronal support and maintaining homeostasis within the CNS, and react to changes in their environment including at the genomic level [11]. In the current study we characterized hypoxia-induced changes in the transcriptomic profile of human astrocytes in vitro, demonstrating that reduced oxygen availability causes significant dysregulation of genes associated with processes which play a role in neurodegenerative pathologies, including mitochondrial function, metabolism, and immune response.

The current study assessed the effect of reduced oxygen availability (1%) on the gene expression profile of astrocytes after 24 h. This hypoxic condition was selected based on previous studies of the cellular response to hypoxia in vitro [19], and the time point selected to ensure that the astrocyte response to hypoxia was optimally induced [13]. Transcriptomic analyses enable the identification of coordinated gene expression trends that are often missed in targeted approaches [32]. To our knowledge, only four studies have previously characterized hypoxia-associated changes in the gene expression profile of astrocytes in vitro [18,19,20,21], and only one of these studies has assessed the human astrocyte response to reduced oxygen availability after 24 h [21]. In contrast to the study by Mense et al. who reported 5-fold more genes were induced as suppressed (FC > 2) [21], we detected almost 3-fold more genes were suppressed as induced in human astrocytes in response to hypoxia using the same U133 plus 2.0 microarray platform. Consistent with the Mense study we detected dysregulation of genes associated with carbohydrate metabolism, signaling pathways (including insulin signaling), apoptosis, cytokines, and growth factors. However, we also identified significant dysregulation of mitochondrial-associated genes and a much greater number of immune-associated transcripts, although it should be noted that at the time less than half of the genes identified in the Mense study were annotated and had known structural or functional information.

Astrocytes play a key role maintaining homeostasis within the CNS, including regulating synaptic transmission, modulating blood flow and permeability of the blood–brain barrier, contributing to the immune response, and maintaining energy levels [33]. The CNS has a very demanding energy requirement, consuming approximately 20% of the oxygen that enters the body. Mitochondria are key organelles for cell bioenergetics, with mitochondrial oxidative phosphorylation accounting for the vast majority of ATP generation in the CNS [34]. In contrast to neurones, astrocytes produce significantly less ATP by oxidative phosphorylation and mainly rely on ATP generated by aerobic glycolysis [35]. In the current study we demonstrate hypoxia-induced downregulation of a number of transcripts associated with mitochondrial function, including those involved in electron transport chain complex 1 assembly and function and the TCA cycle, and a significant increase in genes associated with glycolytic function indicating a switch to a more anaerobic metabolic profile to meet bioenergetic demands under hypoxia. These findings were validated at a functional level, by measuring metabolic flux in real time after hypoxic treatment. Hypoxia reduced coupled mitochondrial respiration in astrocytes and increased basal glycolytic flux, altering the metabolic equilibrium in the cell to a less aerobic phenotype. Interestingly, when the cells were stressed by addition of mitochondrial inhibitors, both mitochondrial and glycolytic capacity were decreased in astrocytes under hypoxia. These data suggest that although astrocytes have the intrinsic metabolic flexibility to change to a more anaerobic phenotype under hypoxia, if they are exposed to further stress they have less aerobic and anaerobic bioenergetic capability. These findings have potential implications for neurodegenerative disorders, which are multifactorial in nature involving multiple pathogenic mechanisms that lead to cellular stress. Mitochondrial dysfunction contributes to the pathogenesis of several neurodegenerative diseases [36]. Exposure of the CNS to hypoxia may leave cells such as astrocytes metabolically vulnerable with less capacity to respond to stress. This could potentially exacerbate any disease state, especially in astrocytes which play an important metabolic support role for neurones and are highly susceptible to inflammation, for example significant metabolic defects have recently been detected in astrocytes derived from ALS patients [28,37].

A loss of mitochondrial maximal respiratory capacity after hypoxia has been reported in neonatal rat cardiac myocyte cultures treated with glucose and palmitate [38]. Although maximal reserve capacity was decreased the authors did not take into account the level of spare respiratory capacity as a factor of the baseline reading of oxygen consumption as we have done in this study. As the level of reserve capacity respiration is intrinsically linked to the level of basal respiration, analysing the data in terms of percentage changes in capacity gives a true reflection of the capacity of the mitochondria at any given time. The authors measured maximal glycolytic capacity which they reported to be increased after hypoxia but again they did not measure reserve capacity and, unlike in this study, only looked at total ECAR rather than non-glycolytic and glycolytic ECAR. A loss of mitochondrial spare respiratory capacity after hypoxia has also been reported in CD4+ T cells, eosinophils, and neutrophils [39,40]. In these studies, glycolytic reserve capacity was not lost in CD4+ T cells; however, non-glycolytic ECAR was not taken into account or was not reported in eosinophils and neutrophils.

An elevated glycolytic flux rate (as measured by the rate of tritium removal from d-[5-3H (*N*)]-glucose) has been observed in solid tumour models exposed to chronic hypoxia [41], whilst elevated glycolytic flux after hypoxia has been reported in blood CD34+ hematopoietic stem cells [42]. However, neither study reported a loss of glycolytic capacity nor measured glycolytic reserve. To the best of our knowledge, our study is the first to report a loss of mitochondrial and glycolytic capacity in astrocytes. Future studies to detail the relationship between astrocyte metabolism and hypoxia in neurodegenerative diseases could identify novel targets to modify astrocyte function.

Mitochondria and the endoplasmic reticulum (ER) interact with each other and play a key role in a number of critical cellular functions, including Ca^2+^ homeostasis, mitochondrial dynamics, bioenergetics, ER stress, apoptotic signalling, and inflammation [26]. Under hypoxic conditions, mitochondria and the (ER) move closer together, enabling the exchange of lipids and ions between these organelles [43]. Our finding that hypoxia impacts expression of a number of transcripts encoding structural components of ER-mitochondria contact sites, including genes encoding mitofusin, suggesting hypoxia may contribute to the structural and functional defects of the ER-mitochondria interface observed in a range of neurodegenerative diseases, including AD, PD, and ALS [44,45].

Neuroinflammation is a pathogenic hallmark of neurodegenerative diseases, proposed to be involved in both their initiation and progression [46]. While some components of the inflammatory response are neuroprotective, increased levels of neuroinflammation are associated with neuronal dysfunction in AD [47], ALS [48], and PD [49]. Evidence from in vitro and in vivo models suggests that neuroinflammation increases the production of oxidative species, disrupts metabolic processes, impacts neuronal function, and causes further glial activation, as reviewed [50,51,52]. In the current study, we demonstrate astrocytes respond to hypoxia by significantly upregulating expression of genes associated with immune function.

Along with microglia, astrocytes contribute to the neuroinflammatory response in neurodegenerative diseases by releasing effector molecules, such as cytokines, that co-ordinate the immune response within the CNS [53,54]. The current study demonstrates that hypoxia significantly upregulates the expression of a number of cytokine genes, predominantly the interleukin family, and chemokine transcripts, including CXCR4. Binding to CXCR4 activates several intracellular signalling pathways associated with chemotaxis, cell survival and/or proliferation, regulating levels of intracellular calcium and gene transcription [55]. CXCR4-dependent astrocyte-microglia signalling has also been shown to prevent neuronal apoptosis [56].

MMPs and their physiological inhibitors TIMPs play a role in regulating the neuroinflammatory response, disruption of the blood–brain barrier (BBB), remodeling of the extracellular matrix and synaptic dysfunction [27]. The expression of most MMPs, including MMP-10, is induced by cytokines and also upregulated after cerebral ischaemia, where they have been implicated in BBB leakage and neurodegeneration [57]. Further work is required to assess how hypoxia-induced astrocyte expression of MMPs and TIMPs relate to neurodegenerative pathology.

The substantive expression of immune-associated transcripts suggests a mechanism whereby astrocytes interact with and modulate their local environment in response to reduced oxygen availability. Targeting the immune response is a potential therapeutic strategy for a range of neurodegenerative diseases [58]. However, while epidemiological studies originally linked the use of non-steroidal anti-inflammatory drugs with a reduced risk of cognitive decline in AD [59], it should be noted that more recent clinical trials suggest this treatment does not slow down the progression of dementia [60]. Furthermore, while prolonged HIF-1 activation is neurotoxic, the initial response is neuroprotective, targeting genes which improve the supply of oxygen, promote glucose metabolism [61], and prevent apoptosis [62]. Indeed the results of recent clinical and experimental studies suggest that increasing HIF-1 signalling may be a strategy to postpone the onset of neurodegenerative diseases or to ameliorate their outcome [8,63,64].

While a major limitation of the current study is the assessment of hypoxia-induced changes in the astrocyte transcriptome in vitro, it should be noted that many of the findings correlate with in vivo studies, thereby demonstrating the translational potential of this research. For example, post-mortem studies of AD cerebral tissue have detected reduced cytochrome oxidase activity associated with a reduction in energy stores which may contribute to the neurodegeneration [65]. Dysregulation in the expression of the chemokine receptor CXCR4 has been demonstrated in a number of neurodegenerative diseases, including AD and PD, particularly in brain regions associated with pathology [66]. Also, MMP-9 expression by reactive astrocytes has been detected in the spinal cord of a rat model of ischaemia-reperfusion injury [67].

In summary, the current study characterises significant changes in the transcriptomic profile of human astrocytes in response to hypoxia in vitro, identifying potential targets for modifying the astrocyte response to reduced oxygen availability in pathological conditions associated with ischaemia/hypoxia, including manipulation of mitochondrial function, metabolism, and the immune response. Future studies using a similar strategy to determine the gene expression profile of patient-derived astrocytes may elucidate disease-specific responses. Additional histopathological studies in well characterized patient cohorts are also required to confirm in vivo the biological relevance of the gene expression changes identified in the human astrocyte monoculture response to hypoxia.

## 4. Materials and Methods

### 4.1. Primary Human Astrocytes

Human primary astrocytes, which we have previously characterized, were obtained from ScienCell Research Laboratories (Carlsbad, CA, USA) [68], grown in Human Astrocyte media (ScienCell Research Laboratories) that was supplemented with 2% fetal bovine serum (FBS), 1% penicillin streptomycin, and 1% Astrocyte Growth Supplement (ScienCell Research Laboratories).

To confirm a response to hypoxia, astrocytes were grown to 80% confluency on coverslips in 12-well plates and incubated under either normoxic (5% CO_2_, 95% air) or hypoxic conditions (1% O_2_, 5% CO_2_, 94% N_2_) for 24 h. After fixation in 4% paraformaldehyde for 20 min at 4 °C the cells were washed in tris buffered saline (TBS) and permeabilised in TBS-tween (0.1% TBS-T) for 15 min at room temperature (RT). Cells were blocked in 1.5% normal goat serum (Vector Laboratories, Peterborough, UK) for 30 min at RT and incubated with rabbit anti-human HIF-1α (1:500 in blocking solution) (Sigma, Gillingham, UK) overnight at 4 °C. After washing in TBS the cells were incubated with 1% biotinylated goat anti-rabbit antibody (Vector Laboratories, Peterborough, UK) for 60 min at RT. Cells were washed in TBS and incubated with streptavidin Alexa Fluor-488 (1:500) (AbCam, Cambridge, UK) for 60 min at RT. Cells were washed in TBS, mounted in Vectastain mounting media with 4′,6-diamidino-2-phenylindole (DAPI) (Vector Laboratories, Peterborough, UK) and viewed using a Nikon microscope (Nikon, Kingston Upon Thames, UK).

To characterize the effect of hypoxia on the astrocyte transcriptome, astrocytes were cultured under normoxic or hypoxic conditions for 24 h. Astrocytes were lysed in 110 μL (1 mL/10 cm^2^) Trizol (Life Technologies, Warrington, UK), and the RNA isolated using the Direct-Zol RNA Miniprep Kit with Zymo-Spin™ IIC Columns (Zymo, Irvine, CA, USA). The quantity and quality of the extracted RNA from biological repeats was assessed using the NanoDrop ND-1000 and Agilent 2100 Bioanalyser, respectively. Samples with an RNA integrity number (RIN) ≥ 8.5 were taken forward for transcriptomic analysis.

### 4.2. Microarray Analysis of Hypoxia-Induced Gene Expression Changes

Double-stranded cDNA was generated from total extracted RNA using an oligo-d(T) primer with a T7 polymerase binding site. Following this, copy RNA (cRNA) was generated to form an RNA template which was used for a second round of amplification to synthesise double-stranded cDNA. Biotin-labelled cRNA was generated using the Affymetrix Gene Chip in vitro transcription labelling kit (Affymetrix, Santa Clara, CA, USA), and the quality and quantity assessed (Agilent Bioanalyser 2100, Agilent, Cheadle, UK). The biotin-labelled cRNA (15µg) was fragmented and hybridised to Human Genome U133 plus 2.0 Arrays for 16 h at 45 °C. Following hybridisation the arrays were washed and stained using the Fluidics Station 400 and the Gene Chip Operating System (GCOS). The microarrays were analysed using the GC3000 7G scanner and quality controls assessed using Expression Console software (Affymetrix) and Qlucore Omics Explorer (Qlucore, Lund, Sweden). The transcriptomic data was normalised using the robust multi-array average (RMA). A principal component analysis (PCA) analysis was used to identify sample outliers. Significant differentially expressed genes (fold change (FC) ≥ 2, *p* ≤ 0.05) were analysed using Qlucore Omics Explorer, and differentially expressed genes visualised using Heatmapper [69]. Functional grouping and pathway analysis of the significantly differentially expressed genes was performed using the DAVID. The dataset generated by this study is freely available at Gene Expression Omnibus, accession number GSE145935.

### 4.3. Validation of Microarray Data: Quantitative Real-Time Polymerase Chain Reaction (qPCR)

RNA was extracted astrocytes cultured under normoxic or hypoxic conditions for 24 h. An IDT PrimeTime qPCR assay (Integrated DNA Technologies, Leuven, Belgium) was performed on a total volume of 10 μL, containing 50 ng cDNA, 500 nM primers, 250 nM probe, and Brilliant qPCR mix (Agilent, Cheadle, UK). Details of primer sequences are shown in Table 4. The samples were denaturated at 95 °C for 3 min and then amplified (40 cycles at 95 °C for 10 s and 60 °C for 30 s) using a CFX384 Touch^TM^ RT PCR detection system (Bio-Rad, Watford, UK). The target genes were normalised to a housekeeping gene (β-actin or GAPDH) using the ΔΔCt calculation (ABI) [70]. Statistical analyses were performed using IBM SPSS Statistics version 23 and GraphPad Prism version 7, and the qPCR data analysed by Student’s paired *t*-test.

### 4.4. XF Bioanalyser Metabolic Flux Analysis

On day 1, astrocytes were plated at 20,000 cells per well in two 24-well assay plates (Agilent), in 500 µL Human Astrocyte media (minus penicillin and streptomycin) and incubated at 37 °C under normoxic conditions for 24 h. Culture media was low oxygen primed for 18 h at 37 °C (5% CO_2_, 1% O_2_, 94% N_2_, hypoxic conditions) in a H35 HEPA filtered and humidity controlled anaerobic chamber (DW Scientific, Bingley, UK). On day 2, 1 astrocyte plate was transferred to the H35 chamber and the media was changed for the low oxygen primed media under sterile conditions. The astrocytes were incubated for 24 h under hypoxia_._ The corresponding control plate also went under a full media change under normoxic conditions and was incubated for 24 h. On day 3, 2 XF24 probe plates (Agilent), were calibrated with 1 ml calibration solution (Agilent) per well and incubated at 37 °C 0% CO_2_ under normoxia or hypoxia for 24 h. Meanwhile XF assay media which consisted of XF base media (Agilent) plus 1.5 mM glutamine and 1.0 mM sodium pyruvate was adjusted to pH 7.4, sterile filtered and half of the media was low oxygen primed for 18 h under 3% O_2_ 37 °C 0% CO_2_ 97% N_2_ hypoxic conditions, in a i2 anaerobic chamber (DW Scientific) whilst the other half was incubated under normoxic conditions in the absence of 5% CO_2._ On the day of the assay, the hypoxic plate was transferred to the i2 chamber through a sealed transfer tunnel to maintain the hypoxic conditions and the media was changed to 675 µL of the low oxygen primed XF assay media. The cells were incubated in the i2 chamber for 1 h prior to the assay starting. In the meantime, the hypoxic XF probe plate had 75 µL 58mM glucose, 82.5 µL 10 µM oligomycin, 91.6 µL 17.5 µM FCCP added to ports A, B, and C respectively. Finally, 10 µM rotenone and antimycin A was added in 100 µL of XF media to port D or 1 M Sodium sulphite was added to port D to assess oxygen levels in the assay. All compounds were diluted 1 in 10 in the assay. The probe plate was calibrated on the XF24 bioanalyser to quality control the probes before the cell plate was added to the XF24 and the assay was started. At the end of the assay, all media was removed from the wells and the plates were stored at −80 °C. The i2 was then opened to reach ambient oxygen levels in the atmosphere and the assay was repeated under normoxic conditions. The next day, the plates were defrosted and cell numbers were determined by addition of CyQUANT (Invitrogen, Inchinnin, UK) to the cells following the manufacturer’s instructions. Briefly, a solution of 1× HBSS buffer and 1 in 400 dilution cyquant dye was made up in water. 100 µL of this solution was added to each well of the 24-well plate, incubated for 10 min in the dark and then fluorescence was read on a BMG pherastar plate reader at 480 mm excitation 520 mm emission. The gain was set to 1% on a blank well to control for cell numbers between assays.

Data analysis: The hypoxic plate was corrected for low oxygen tension using the XF hypoxia rate calculator software (Agilent) with sodium sulphite as a reference. All data was then transferred to Microsoft Excel and normalised to the CyQUANT data to correct for any cell number differences. Mitochondrial respiration was calculated by subtracting oxygen consumption rate (OCR) in the presence of rotenone/antimycin A from OCR in the presence of glucose only. Coupled respiration was calculated by removing OCR in the presence of oligomycin away from mitochondrial respiration. Uncoupled respiration was calculated by removing OCR in the presence of rotenone/antimycin A from OCR in the presence of oligomycin. Spare respiratory capacity was calculated by subtracting mitochondrial respiration away from OCR in the presence of FCCP. Non glycolytic extracellular acidification rate was classed as ECAR in the absence of glucose. Glycolytic ECAR was calculated by subtracting ECAR in the absence of glucose from ECAR in the presence of glucose. Glycolytic capacity was calculated by removing ECAR in the presence of glucose from ECAR in the presence of oligomycin. Finally, glycolytic reserve was calculated by subtracting glycolytic ECAR from ECAR in the presence of oligomycin.

Statistical analysis: For the XF metabolic flux analysis to test the data for Gaussian distribution, prior to statistical analysis, all data were assessed for normal distribution by the Kolmogorov–Smirnov, D’Agostino–Pearson, and Sharpiro–Wilk normality tests. If normal, an unpaired *t*-test was performed; if not normal, then a Mann–Whitney was performed. All percentage data were first transformed Y = 1/Y, then subsequently Y = logit(Y) prior to analysis by unpaired *t*-test or Wilcoxon rank test analysis. Statistical analysis was performed on GraphPad Prism software (version 6.0, San Diego, CA, USA).

## Figures and Tables

**Table 1:** Expression of hypoxia-induced transcripts

Transcript	Gene Symbol	Gene Name	FC	*p*-Value
202912_at	*ADM*	adrenomedullin	3.4	0.011
217254_s_at	*EPO*	erythropoietin	1.9	0.039
209328_x_at	*HIGD2A*	HIG1 hypoxia inducible domain family, member 2A	1.3	0.042
209329_x_at	*HIGD2A*	HIG1 hypoxia inducible domain family, member 2A	1.6	0.040
218507_at	*HILPDA*	hypoxia inducible lipid droplet-associated	6.1	0.018
1554452_a_at	*HILPDA*	hypoxia inducible lipid droplet-associated	4.4	0.018
207092_at	*LEP*	leptin	1.6	0.030
229093_at	*NOS3*	nitric oxide synthase 3 (endothelial cell)	6.4	0.028
205581_s_at	*NOS3*	nitric oxide synthase 3 (endothelial cell)	1.8	0.035
217112_at	*PDGFB*	platelet-derived growth factor beta	2.3	0.048
216061_x_at	*PDGFB*	platelet-derived growth factor beta	1.8	0.028
203400_s_at	*TF*	transferrin	1.8	0.031
212171_x_at	*VEGFA*	vascular endothelial growth factor A	4.2	0.020
211527_x_at	*VEGFA*	vascular endothelial growth factor A	2.7	0.054
210513_s_at	*VEGFA*	vascular endothelial growth factor A	2.7	0.030
203683_s_at	*VEGFB*	vascular endothelial growth factor B	2.0	0.052
209946_at	*VEGFC*	vascular endothelial growth factor C	1.3	0.051

**Table 2:** Kyoto encyclopedia of genes and genomes (KEGG) pathway analysis of all significantly upregulated genes in response to hypoxia in human astrocytes

KEGG Pathway	*p*-Value
Hypoxia inducible factor-1 (HIF-1) signalling pathway	0.002
Phosphatidylinositol 3-kinase and protein kinase B (PI3K-Akt) signalling pathway	0.005
Renal cell carcinoma	0.006
Glycolysis/Gluconeogenesis	0.006
Staphylococcus aureus infection	0.007
Oxytocin signalling pathway	0.010
Pathways in cancer	0.013
Insulin resistance	0.014
Human T- cell leukemia virus, type 1 (HTLV-I) infection	0.019
Biosynthesis of amino acids	0.030
adenosine monophosphate-activated protein kinase (AMPK) signalling pathway	0.032
Cell adhesion molecules (CAMs)	0.035
Circadian rhythm	0.038
Fructose and mannose metabolism	0.043
Intestinal immune network for IgA production	0.045
Graft-versus-host disease	0.047

**Table 3:** KEGG pathway analysis of all significantly downregulated genes in response to hypoxia in human astrocytes

KEGG Pathway	*p*-Value
Cell cycle	6.1 × 10^−9^
RNA transport	1.9 × 10^−^^7^
Ubiquitin mediated proteolysis	4.5 × 10^−^^7^
Oocyte meiosis	1.9 × 10^−^^5^
Protein processing in endoplasmic reticulum	5.6 × 10^−^^5^
RNA degradation	1.6 × 10^−^^4^
Spliceosome	3.6 × 10^−^^4^
Wnt signalling	0.002
Endocytosis	0.006
Pyramidine metabolism	0.008
Progesterone-mediated oocyte maturation	0.012
Peroxisome	0.015
Tricarboxylic acid cycle (TCA cycle)	0.016
p53 signalling	0.016
DNA replication	0.019
Pathways in cancer	0.02
Purine metabolism	0.02
Valine, leucine and isoleucine degradation	0.02
RNA polymerase	0.02
Fatty acid metabolism	0.03
Protein export	0.03
Chronic myeloid leukemia	0.03
mRNA surveillance pathway	0.04
HTLV-I infection	0.04

**Table 4:** qPCR primer-probe set sequences

Gene		Sequence
*DLAT*	Probe	56-FAM/CGCTGTGCA/ZEN/ATAACCCGACGAATG/3IABkFQ
	Primer 1	CCAGTTCCTACAGGTGTCTTC
	Primer 2	TGAGGTATGGTTTGCTTTGATTG
*HLADPB1*	Probe	56-FAM/CCCACTCCA/ZEN/CAGATGATGAGCCC/3IABkFQ
	Primer 1	GCTCCTCCTGTGCATGAAG
	Primer 2	CAAGTGGAGCACACCAG
*MFN1*	Probe	56-FAM/AGCTTCTAC/ZEN/TCCCACTGCTCCTACC/3IABkFQ
	Primer 1	GAAATGCTCAAAGGGTGCTC
	Primer 2	GTGATGCATTATCTGGCGTTG
*MT1X*	Probe	56-FAM/AGCTCGCCA/ZEN/TGGATCCCAACT/3IABkFQ
	Primer 1	GCAACCTGTCCCGACTCTA
	Primer 2	AGCTTTTCTTGCAGGAGGTG
*NDUFAF4*	Probe	56-FAM/CTGTGTCTT/ZEN/CCTTGCAGGTAAAAGCTG/3IABkFQ
	Primer 1	CCAGAAGTTAAAGGAGAGATTGCT
	Primer 2	GAATTCCTTCGGCTCTTGAC
*SUCLG2*	Probe	56-FAM/CACAGCTGA/ZEN/TCCTAAGGTTGAAGCCA/3IABkFQ
	Primer 1	TTGGAGGTGGTGTAAAGGAAG
	Primer 2	GGCAATGATGGCACAGTTG
*ACTB*	Probe	56-FAM/CTGCCTCCA/ZEN/CCCACTCCCA/3IABkFQ
	Primer 1	GTCCCCCAACTTGAGATGTATG
	Primer 2	AAGTCAGTGTACAGGTAAGCC
*GAPDH*	Probe	56-FAM/AAGGTCGGA/ZEN/GTCAACGGATTTGGTC/3IABkFQ
	Primer 1	ACATCGCTCAGACACCATG
	Primer 2	TGTAGTTGAGGTCAATGAAGGG

## Supplementary Materials

The following are available online at https://www.mdpi.com/1422-0067/21/21/8028/s1, Table S1: Genes upregulated in human astrocytes in response to hypoxia. Table S2: Genes downregulated in human astrocytes in response to hypoxia.

## Abbreviations

AD	Alzheimer’s disease
ADAM	a disintegrin and metalloproteinase
ALS	amyotrophic lateral sclerosis
ATP	adenosine tri-phosphate
CNS	central nervous system
CR	coupled to respiration ATP-linked flux
DAVID	Database for Annotation Visualisation and Integrated Discovery
ECAR	extracellular acidification rate
ETC	electron transport chain
FC	fold change
FCCP	carbonyl cyanide p-trifluoromethoxy phenylhydrazone
HIF-1	hypoxia inducible factor-1
HREs	hypoxia responsive elements
IGF	insulin-like growth factor
IGFR	insulin-like growth factor receptors
IL	interleukin
IR	insulin receptor
IRS	receptor substrates
KEGG	Kyoto Encyclopedia of Genes and Genomes
MMP	matrix metalloproteinases
MR	mitochondrial respiratory flux
NGF	non-glycolytic flux
OCR	oxygen consumption rate
PD	Parkinson’s disease
PL	proton leak linked flux
PrG	pre-glucose
PsG	post-glucose
QPCR	quantitative polymerase chain reaction
RIN	RNA Integrity Number
SRC	spare respiratory capacity
TCA	tri-carboxylic acid cycle
TIMP	tissue inhibitor of metalloproteinase
