# Supplementary material for: Transcriptomic Analysis of Human Astrocytes In Vitro Reveals Hypoxia-Induced Mitochondrial Dysfunction, Modulation of Metabolism, and Dysregulation of the Immune Response
Source: Int J Mol Sci. 2020 Oct 28;21(21):8028. doi: 10.3390/ijms21218028 (PMC7672558; doi:10.3390/ijms21218028)
Supplement: Supplementary file 1 [file ijms-21-08028-s001.zip › Supplementary Table 1.docx]

**Supplementary Table 1.** Genes upregulated in human astrocytes in response to hypoxia (FC≥2, p<0.05)

| **Transcript** | **Gene symbol** | **Gene name** | **FC** | **p-value** |
| --- | --- | --- | --- | --- |
| 205199_at | *CA9* | carbonic anhydrase IX | 17.87 | 6.2x10^-5^ |
| 219670_at | *BEND5* | BEN domain containing 5 | 16.12 | 4x10^-6^ |
| 204596_s_at | *STC1* | stanniocalcin 1 | 16.12 | 2.8x10^-5^ |
| 222847_s_at | *EGLN3* | egl nine homolog 3 (C. elegans) | 15.98 | 3.6x10^-5^ |
| 204595_s_at | *STC1* | stanniocalcin 1 | 14.31 | 8x10^-6^ |
| 231478_at | *LOC729966* | uncharacterized LOC729966; phosphodiesterase 4C, cAMP-specific | 14.22 | 1.7x10^-5^ |
| 224646_x_at | *H19* | H19, imprinted maternally expressed transcript (non-protein coding); microRNA 675 | 13.65 | 9.7x10^-5^ |
| 219232_s_at | *EGLN3* | egl nine homolog 3 (C. elegans) | 10.7 | 2.3x10^-5^ |
| 219888_at | *SPAG4* | sperm associated antigen 4 | 10.69 | 0.00013 |
| 214978_s_at | *PPFIA4* | protein tyrosine phosphatase, receptor type, f polypeptide (PTPRF), interacting protein (liprin), alpha 4 | 9.66 | 0.00032 |
| 218149_s_at | *ZNF395* | zinc finger protein 395 | 9.64 | 4x10^-6^ |
| 221009_s_at | *ANGPTL4* | angiopoietin-like 4 | 9.32 | 8.6x10^-8^ |
| 200632_s_at | *NDRG1* | N-myc downstream regulated 1 | 8.86 | 2.1x10^-5^ |
| 204597_x_at | *STC1* | stanniocalcin 1 | 8.51 | 8.5x10^-5^ |
| 230864_at | *NIM1* | serine/threonine-protein kinase NIM1 | 8.37 | 0.00066 |
| 236474_at |  |  | 8.26 | 9.4x10^-5^ |
| 230746_s_at |  |  | 7.86 | 0.00052 |
| 210479_s_at | *RORA* | RAR-related orphan receptor A | 7.69 | 7.8x10^-5^ |
| 229252_at | *ATG9B* | autophagy related 9B | 7.61 | 7.6x10^-5^ |
| 231803_at | *FGF11* | fibroblast growth factor 11 | 7.44 | 0.01221 |
| 209183_s_at | *C10orf10* | chromosome 10 open reading frame 10 | 7.4 | 4.3x10^-5^ |
| 1556364_at | *ADAMTS9-AS2* | ADAMTS9 antisense RNA 2 | 7.02 | 0.00028 |
| 227271_at | *FGF11* | fibroblast growth factor 11 | 6.76 | 0.00043 |
| 239619_at |  |  | 6.69 | 5.9x10^-5^ |
| 204508_s_at | *CA12* | carbonic anhydrase XII | 6.62 | 3.6x10^-5^ |
| 200986_at | *SERPING1* | serpin peptidase inhibitor, clade G (C1 inhibitor), member 1 | 6.55 | 0.00012 |
| 221123_x_at | *FBXO16* | F-box protein 16; zinc finger protein 395 | 6.47 | 2.2x10^-5^ |
| 223216_x_at | *FBXO16* | F-box protein 16; zinc finger protein 395 | 6.36 | 1.5x10^-5^ |
| 229093_at | *NOS3* | nitric oxide synthase 3 (endothelial cell) | 6.36 | 0.00095 |
| 228499_at | *PFKFB4* | 6-phosphofructo-2-kinase/fructose-2,6-biphosphatase 4 | 6.29 | 0.00032 |
| 226682_at | *RORA* | RAR-related orphan receptor A | 6.21 | 0.00047 |
| 218507_at | *HILPDA* | hypoxia inducible lipid droplet-associated | 6.08 | 0.00005 |
| 228770_at | *GPR146* | G protein-coupled receptor 146 | 6.04 | 0.00038 |
| 224348_s_at | *RPSA* | ribosomal protein SA | 6.03 | 0.00029 |
| 232693_s_at | *FBXO16* | F-box protein 16; zinc finger protein 395 | 5.86 | 0.00024 |
| 203961_at | *NEBL* | nebulette | 5.8 | 0.00024 |
| 228051_at | *KIAA1244* | KIAA1244 | 5.74 | 0.00027 |
| 228658_at | *MIAT* | myocardial infarction associated transcript (non-protein coding) | 5.62 | 0.00105 |
| 201650_at | *KRT19* | keratin 19 | 5.54 | 4.8x10^-5^ |
| 203963_at | *CA12* | carbonic anhydrase XII | 5.32 | 1.1x10^-5^ |
| 224997_x_at | *H19* | H19, imprinted maternally expressed transcript (non-protein coding); microRNA 675 | 5.26 | 0.00012 |
| 223193_x_at | *FAM162A* | family with sequence similarity 162, member A | 5.19 | 5.2x10^-5^ |
| 206730_at | *GRIA3* | glutamate receptor, ionotropic, AMPA 3 | 5.17 | 0.00027 |
| 224345_x_at | *FAM162A* | family with sequence similarity 162, member A | 5.15 | 0.00011 |
| 205492_s_at | *DPYSL4* | dihydropyrimidinase-like 4 | 5.07 | 8.6x10^-5^ |
| 32137_at | *JAG2* | jagged 2 | 5.04 | 0.0002 |
| 1557803_at |  |  | 5.02 | 0.01947 |
| 220942_x_at | *FAM162A* | family with sequence similarity 162, member A | 4.94 | 6.9x10^-5^ |
| 236480_at | *MIR210HG* | MIR210 host gene (non-protein coding) | 4.82 | 0.00136 |
| 210426_x_at | *RORA* | RAR-related orphan receptor A | 4.78 | 0.00097 |
| 230695_s_at | *RSPH9* | radial spoke head 9 homolog (Chlamydomonas) | 4.71 | 0.00026 |
| 204430_s_at | *SLC2A5* | solute carrier family 2 (facilitated glucose/fructose transporter), member 5 | 4.68 | 0.00115 |
| 1554384_at | *PADI2* | peptidyl arginine deiminase, type II | 4.63 | 4.8x10^-5^ |
| 232914_s_at | *SYTL2* | synaptotagmin-like 2 | 4.61 | 2.4x10^-5^ |
| 225496_s_at | *SYTL2* | synaptotagmin-like 2 | 4.61 | 0.00032 |
| 215867_x_at | *CA12* | carbonic anhydrase XII | 4.52 | 8.9x10^-5^ |
| 201249_at | *SLC2A1* | solute carrier family 2 (facilitated glucose transporter), member 1 | 4.52 | 0.00088 |
| 240991_at |  |  | 4.49 | 0.0019 |
| 229001_at | *PPP1R3E* | protein phosphatase 1, regulatory subunit 3E | 4.48 | 1.3x10^-5^ |
| 219213_at | *JAM2* | junctional adhesion molecule 2 | 4.48 | 0.0003 |
| 229327_s_at |  |  | 4.48 | 0.00058 |
| 202575_at | *CRABP2* | cellular retinoic acid binding protein 2 | 4.43 | 4.5x10^-5^ |
| 238750_at | *CCL28* | chemokine (C-C motif) ligand 28 | 4.39 | 0.001 |
| 232694_at | *ZNF395* | zinc finger protein 395 | 4.39 | 0.00384 |
| 1554452_a_at | *HILPDA* | hypoxia inducible lipid droplet-associated | 4.35 | 4.3x10^-5^ |
| 205738_s_at | *FABP3* | fatty acid binding protein 3, muscle and heart (mammary-derived growth inhibitor) | 4.29 | 0.00042 |
| 233242_at | *WDR73* | WD repeat domain 73 | 4.27 | 0.00197 |
| 1566633_at |  |  | 4.23 | 9.8x10^-5^ |
| 212171_x_at | *VEGFA* | vascular endothelial growth factor A | 4.22 | 0.00011 |
| 201313_at | *ENO2* | enolase 2 (gamma, neuronal) | 4.21 | 7.2x10^-5^ |
| 217028_at | *CXCR4* | chemokine (C-X-C motif) receptor 4 | 4.19 | 3x10^-6^ |
| 228959_at | *PDK3* | pyruvate dehydrogenase kinase, isozyme 3 | 4.19 | 3.6x10^-5^ |
| 227717_at | *ARHGEF37* | Rho guanine nucleotide exchange factor (GEF) 37 | 4.19 | 0.00016 |
| 219747_at | *NDNF* | neuron-derived neurotrophic factor | 4.17 | 0.00028 |
| 213693_s_at | *MUC1* | mucin 1, cell surface associated | 4.16 | 0.0003 |
| 204347_at | *AK4* | adenylate kinase 4; adenylate kinase isoenzyme 4, mitochondrial-like | 4.15 | 4.8x10^-5^ |
| 211312_s_at | *WISP1* | WNT1 inducible signaling pathway protein 1 | 4.15 | 0.00036 |
| 1553572_a_at | *CYGB* | cytoglobin | 4.11 | 0.0012 |
| 1560259_at |  |  | 4.09 | 0.00914 |
| 227868_at | *LOC154761* | family with sequence similarity 115, member C pseudogene | 4.08 | 3.5x10^-5^ |
| 230630_at | *AK4* | adenylate kinase 4; adenylate kinase isoenzyme 4, mitochondrial-like | 4.05 | 0.00591 |
| 1556684_at | *RPPH1* | ribonuclease P RNA component H1 | 4.05 | 0.01494 |
| 209201_x_at | *CXCR4* | chemokine (C-X-C motif) receptor 4 | 4.02 | 0.00038 |
| 232602_at | *WFDC3* | WAP four-disulfide core domain 3 | 3.99 | 0.00044 |
| 204348_s_at | *AK4* | adenylate kinase 4; adenylate kinase isoenzyme 4, mitochondrial-like | 3.98 | 4.9x10^-5^ |
| 208032_s_at | *GRIA3* | glutamate receptor, ionotropic, AMPA 3 | 3.98 | 0.00121 |
| 235821_at | *WISP1* | WNT1 inducible signaling pathway protein 1 | 3.95 | 0.00017 |
| 236180_at |  |  | 3.95 | 0.0013 |
| 233388_at |  |  | 3.92 | 5.7x10^-5^ |
| 240988_x_at |  |  | 3.92 | 0.03667 |
| 227412_at | *PPP1R3E* | protein phosphatase 1, regulatory subunit 3E | 3.9 | 6.1x10^-5^ |
| 53991_at | *DENND2A* | DENN/MADD domain containing 2A | 3.88 | 1.4x10^-5^ |
| 209596_at | *MXRA5* | matrix-remodelling associated 5 | 3.88 | 0.00123 |
| 222536_s_at | *ZNF395* | zinc finger protein 395 | 3.85 | 8x10^-6^ |
| 206796_at | *WISP1* | WNT1 inducible signaling pathway protein 1 | 3.84 | 0.00025 |
| 228362_s_at | *FAM26F* | family with sequence similarity 26, member F | 3.83 | 0.00217 |
| 202022_at | *ALDOC* | aldolase C, fructose-bisphosphate | 3.75 | 0.00015 |
| 1557545_s_at | *RNF165* | ring finger protein 165 | 3.75 | 0.00347 |
| 242517_at | *KISS1R* | KISS1 receptor | 3.74 | 7x10^-6^ |
| 224314_s_at | *EGLN1* | egl nine homolog 1 (C. elegans) | 3.74 | 0.00011 |
| 207279_s_at | *NEBL* | nebulette | 3.74 | 0.01505 |
| 209784_s_at | *JAG2* | jagged 2 | 3.73 | 0.00021 |
| 201848_s_at | *BNIP3* | BCL2/adenovirus E1B 19kDa interacting protein 3 | 3.7 | 0.0006 |
| 202286_s_at | *TACSTD2* | tumor-associated calcium signal transducer 2 | 3.69 | 0.00011 |
| 218537_at | *HCFC1R1* | host cell factor C1 regulator 1 (XPO1 dependent) | 3.67 | 0.0043 |
| 209566_at | *INSIG2* | insulin induced gene 2 | 3.65 | 0.00022 |
| 225342_at | *AK4* | adenylate kinase 4; adenylate kinase isoenzyme 4, mitochondrial-like | 3.64 | 0.00038 |
| 218625_at | *NRN1* | neuritin 1 | 3.62 | 0.00018 |
| 211919_s_at | *CXCR4* | chemokine (C-X-C motif) receptor 4 | 3.59 | 0.00302 |
| 217840_at | *DDX41* | DEAD (Asp-Glu-Ala-Asp) box polypeptide 41 | 3.56 | 0.00039 |
| 206039_at | *RAB33A* | RAB33A, member RAS oncogene family | 3.56 | 0.00846 |
| 225750_at | *ERO1L* | ERO1-like (S. cerevisiae) | 3.53 | 1.3x10^-5^ |
| 230612_at | *WDR73* | WD repeat domain 73 | 3.53 | 0.00079 |
| 205970_at | *MT3* | metallothionein 3 | 3.53 | 0.00126 |
| 209496_at | *RARRES2* | retinoic acid receptor responder (tazarotene induced) 2 | 3.51 | 0.00056 |
| 229802_at | *WISP1* | WNT1 inducible signaling pathway protein 1 | 3.5 | 0.0002 |
| 224602_at | *C4orf3* | chromosome 4 open reading frame 3 | 3.48 | 0.00105 |
| 205493_s_at | *DPYSL4* | dihydropyrimidinase-like 4 | 3.48 | 0.00253 |
| 217380_s_at | *ADD3-AS1* | ADD3 antisense RNA 1 | 3.47 | 1.6x10^-5^ |
| 202499_s_at | *SLC2A3* | solute carrier family 2 (facilitated glucose transporter), member 3 | 3.46 | 0.00022 |
| 219622_at | *RAB20* | RAB20, member RAS oncogene family | 3.45 | 0.00011 |
| 212444_at | *GPRC5A* | G protein-coupled receptor, family C, group 5, member A | 3.45 | 0.00022 |
| 228844_at | *SLC13A5* | solute carrier family 13 (sodium-dependent citrate transporter), member 5 | 3.44 | 0.00388 |
| 204865_at | *CA3* | carbonic anhydrase III, muscle specific | 3.43 | 0.0002 |
| 207847_s_at | *MUC1* | mucin 1, cell surface associated | 3.43 | 0.0015 |
| 200831_s_at | *SCD* | stearoyl-CoA desaturase (delta-9-desaturase) | 3.42 | 0.00238 |
| 216236_s_at | *SLC2A14* | solute carrier family 2 (facilitated glucose transporter), member 14; solute carrier family 2 (facilitated glucose transporter), member 3 | 3.41 | 3x10^-6^ |
| 212091_s_at | *COL6A1* | collagen, type VI, alpha 1 | 3.41 | 0.00072 |
| 209968_s_at | *NCAM1* | neural cell adhesion molecule 1 | 3.37 | 0.00016 |
| 232481_s_at | *SLITRK6* | SLIT and NTRK-like family, member 6 | 3.36 | 0.0006 |
| 202912_at | *ADM* | adrenomedullin | 3.35 | 6x10^-6^ |
| 232176_at | *SLITRK6* | SLIT and NTRK-like family, member 6 | 3.35 | 0.0018 |
| 217685_at |  |  | 3.33 | 0.00026 |
| 237137_at | *SCARNA2* | small Cajal body-specific RNA 2 | 3.33 | 0.01437 |
| 227526_at | *CDON* | cell adhesion associated, oncogene regulated | 3.3 | 0.00023 |
| 209791_at | *PADI2* | peptidyl arginine deiminase, type II | 3.29 | 0.00025 |
| 232983_s_at | *SERGEF* | secretion regulating guanine nucleotide exchange factor | 3.29 | 0.00123 |
| 242972_at | *HCG18* | HLA complex group 18 (non-protein coding) | 3.28 | 0.00024 |
| 229391_s_at | *FAM26F* | family with sequence similarity 26, member F | 3.28 | 0.00026 |
| 236037_at | *KIAA1244* | KIAA1244 | 3.28 | 0.00034 |
| 210735_s_at | *CA12* | carbonic anhydrase XII | 3.28 | 0.00034 |
| 223121_s_at | *SFRP2* | secreted frizzled-related protein 2 | 3.27 | 0.00732 |
| 201673_s_at | *GYS1* | glycogen synthase 1 (muscle) | 3.26 | 0.00037 |
| 209383_at | *DDIT3* | DNA-damage-inducible transcript 3 | 3.26 | 0.00117 |
| 225762_x_at | *RNA45S5* | RNA, 45S pre-ribosomal 5 | 3.25 | 0.00838 |
| 217590_s_at | *TRPA1* | transient receptor potential cation channel, subfamily A, member 1 | 3.24 | 0.00021 |
| 220382_s_at | *ARHGAP28* | Rho GTPase activating protein 28 | 3.24 | 0.01024 |
| 235563_at | *GPRC5A* | G protein-coupled receptor, family C, group 5, member A | 3.24 | 0.01059 |
| 207366_at | *KCNS1* | potassium voltage-gated channel, delayed-rectifier, subfamily S, member 1 | 3.22 | 0.0001 |
| 225898_at | *WDR54* | WD repeat domain 54 | 3.22 | 0.00026 |
| 227285_at | *C1orf51* | chromosome 1 open reading frame 51 | 3.22 | 0.00084 |
| 239370_at |  |  | 3.21 | 1.6x10^-5^ |
| 223333_s_at | *ANGPTL4* | angiopoietin-like 4 | 3.2 | 3.4x10^-5^ |
| 224240_s_at | *CCL28* | chemokine (C-C motif) ligand 28 | 3.2 | 0.00172 |
| 234370_at | *VSIG1* | V-set and immunoglobulin domain containing 1 | 3.2 | 0.0047 |
| 210881_s_at | *IGF2* | insulin-like growth factor 2 (somatomedin A); INS-IGF2 readthrough | 3.2 | 0.00685 |
| 227337_at | *ANKRD37* | ankyrin repeat domain 37 | 3.18 | 5.1x10^-5^ |
| 206348_s_at | *PDK3* | pyruvate dehydrogenase kinase, isozyme 3 | 3.18 | 0.00013 |
| 222245_s_at | *FER1L4* | fer-1-like 4 (C. elegans) pseudogene | 3.18 | 0.00062 |
| 205258_at | *INHBB* | inhibin, beta B | 3.18 | 0.00087 |
| 230710_at | *MIR210HG* | MIR210 host gene (non-protein coding) | 3.18 | 0.00111 |
| 222088_s_at | *SLC2A14* | solute carrier family 2 (facilitated glucose transporter), member 14; solute carrier family 2 (facilitated glucose transporter), member 3 | 3.17 | 0.00045 |
| 227501_at |  |  | 3.17 | 0.00103 |
| 221885_at | *DENND2A* | DENN/MADD domain containing 2A | 3.17 | 0.00116 |
| 214560_at | *FPR3* | formyl peptide receptor 3 | 3.15 | 0.00027 |
| 232336_at | *ZSWIM5* | zinc finger, SWIM-type containing 5 | 3.14 | 4.9x10^-5^ |
| 220994_s_at | *STXBP6* | syntaxin binding protein 6 (amisyn) | 3.14 | 0.00085 |
| 209156_s_at | *COL6A2* | collagen, type VI, alpha 2 | 3.13 | 0.00311 |
| 1562682_at | *RORA* | RAR-related orphan receptor A | 3.13 | 0.01928 |
| 206758_at | *EDN2* | endothelin 2 | 3.12 | 0.00095 |
| 1562770_at |  |  | 3.11 | 0.00015 |
| 202219_at | *SLC6A8* | solute carrier family 6 (neurotransmitter transporter, creatine), member 8 | 3.11 | 0.00018 |
| 234151_at |  |  | 3.11 | 0.00033 |
| 226382_at | *CAMK1D* | calcium/calmodulin-dependent protein kinase ID; uncharacterized LOC283070 | 3.1 | 0.0004 |
| 224131_at | *HCAR1* | hydroxycarboxylic acid receptor 1 | 3.09 | 4.8x10^-5^ |
| 236036_at |  |  | 3.09 | 8.3x10^-5^ |
| 237322_at | *MIAT* | myocardial infarction associated transcript (non-protein coding) | 3.09 | 0.00173 |
| 223168_at | *RHOU* | ras homolog family member U | 3.07 | 0.00156 |
| 235857_at | *KCTD11* | potassium channel tetramerisation domain containing 11 | 3.07 | 0.00305 |
| 214036_at | *EFNA5* | ephrin-A5 | 3.06 | 0.00025 |
| 227573_s_at | *OBSL1* | obscurin-like 1 | 3.05 | 8.8x10^-5^ |
| 227892_at | *PRKAA2* | protein kinase, AMP-activated, alpha 2 catalytic subunit | 3.03 | 0.01408 |
| 1554079_at | *GALNT18* | UDP-N-acetyl-alpha-D-galactosamine:polypeptide N-acetylgalactosaminyltransferase 18 | 3.02 | 0.00027 |
| 234973_at | *SLC38A5* | solute carrier family 38, member 5 | 3.02 | 0.00109 |
| 226388_at | *TCEA3* | transcription elongation factor A (SII), 3 | 3.02 | 0.00177 |
| 1560049_at |  |  | 3.01 | 0.01197 |
| 212143_s_at | *IGFBP3* | insulin-like growth factor binding protein 3 | 3 | 0.00032 |
| 211996_s_at |  |  | 3 | 0.00068 |
| 1553288_a_at | *NYAP1* | neuronal tyrosine-phosphorylated phosphoinositide-3-kinase adaptor 1 | 3 | 0.01033 |
| 210424_s_at | *GOLGA8A* | golgin A8 family, member A; golgin A8 family, member B | 2.99 | 0.00812 |
| 218484_at | *NDUFA4L2* | NADH dehydrogenase (ubiquinone) 1 alpha subcomplex, 4-like 2 | 2.98 | 2x10^-6^ |
| 227168_at | *MIAT* | myocardial infarction associated transcript (non-protein coding) | 2.98 | 0.00073 |
| 229215_at | *ASCL2* | achaete-scute complex homolog 2 (Drosophila) | 2.98 | 0.00073 |
| 206686_at | *PDK1* | pyruvate dehydrogenase kinase, isozyme 1 | 2.97 | 0.00011 |
| 1554385_a_at | *PADI2* | peptidyl arginine deiminase, type II | 2.97 | 0.00103 |
| 238965_at | *AP001062.7* | NULL | 2.97 | 0.00918 |
| 211818_s_at | *LOC729966* | uncharacterized LOC729966; phosphodiesterase 4C, cAMP-specific | 2.96 | 0.00024 |
| 206363_at | *MAF* | v-maf musculoaponeurotic fibrosarcoma oncogene homolog (avian) | 2.96 | 0.00024 |
| 221756_at | *PIK3IP1* | phosphoinositide-3-kinase interacting protein 1 | 2.96 | 0.00261 |
| 244249_at |  |  | 2.96 | 0.00279 |
| 235948_at | *RIMKLA* | ribosomal modification protein rimK-like family member A | 2.96 | 0.00305 |
| 218274_s_at | *ANKZF1* | ankyrin repeat and zinc finger domain containing 1 | 2.96 | 0.00417 |
| 205613_at | *SYT17* | synaptotagmin XVII | 2.95 | 0.00479 |
| 210365_at |  |  | 2.94 | 0.02035 |
| 223484_at | *C15orf48* | chromosome 15 open reading frame 48 | 2.93 | 0.00679 |
| 237940_s_at |  |  | 2.92 | 0.00024 |
| 233234_at | *KCTD16* | potassium channel tetramerisation domain containing 16 | 2.91 | 0.00081 |
| 227452_at | *LINC00511* | long intergenic non-protein coding RNA 511; long intergenic non-protein coding RNA 673 | 2.91 | 0.00352 |
| 208300_at | *PTPRH* | protein tyrosine phosphatase, receptor type, H | 2.9 | 0.00037 |
| 211182_x_at |  |  | 2.9 | 0.00192 |
| 230941_at |  |  | 2.89 | 0.00014 |
| 229543_at |  |  | 2.89 | 0.00914 |
| 221567_at | *NOL3* | nucleolar protein 3 (apoptosis repressor with CARD domain) | 2.88 | 5.1x10^-5^ |
| 45714_at | *HCFC1R1* | host cell factor C1 regulator 1 (XPO1 dependent) | 2.87 | 0.00105 |
| 229189_s_at |  |  | 2.87 | 0.00417 |
| 1553392_at | *EFCAB3* | EF-hand calcium binding domain 3 | 2.87 | 0.00955 |
| 214164_x_at | *CA12* | carbonic anhydrase XII | 2.86 | 0.00098 |
| 207230_at | *CDON* | cell adhesion associated, oncogene regulated | 2.85 | 0.00125 |
| 36129_at | *SGSM2* | small G protein signaling modulator 2 | 2.85 | 0.00225 |
| 202856_s_at | *SLC16A3* | solute carrier family 16, member 3 (monocarboxylic acid transporter 4) | 2.84 | 0.00064 |
| 200884_at | *CKB* | creatine kinase, brain | 2.84 | 0.00639 |
| 218848_at | *THOC6* | THO complex 6 homolog (Drosophila) | 2.83 | 0.00106 |
| 209305_s_at | *GADD45B* | growth arrest and DNA-damage-inducible, beta | 2.83 | 0.00225 |
| 213650_at | *GOLGA8A* | golgin A8 family, member A; golgin A8 family, member B | 2.83 | 0.00373 |
| 218180_s_at | *EPS8L2* | EPS8-like 2 | 2.82 | 0.00075 |
| 1559296_at | *ADAMTS9-AS2* | ADAMTS9 antisense RNA 2 | 2.82 | 0.00104 |
| 205553_s_at | *CSRP3* | cysteine and glycine-rich protein 3 (cardiac LIM protein) | 2.82 | 0.02605 |
| 214927_at | *ITGBL1* | integrin, beta-like 1 (with EGF-like repeat domains) | 2.81 | 9x10^-6^ |
| 201009_s_at |  | thioredoxin-interacting protein-like; thioredoxin interacting protein | 2.81 | 6.2x10^-5^ |
| 1553770_a_at | *SLAMF9* | SLAM family member 9 | 2.81 | 0.00018 |
| 226121_at | *DHRS13* | dehydrogenase/reductase (SDR family) member 13 | 2.8 | 1.2x10^-5^ |
| 221221_s_at | *KLHL3* | kelch-like family member 3 | 2.8 | 0.00164 |
| 1555753_x_at | *ERVH-6* | endogenous retrovirus group H, member 6 | 2.8 | 0.02776 |
| 223172_s_at | *MTFP1* | mitochondrial fission process 1 | 2.79 | 0.00121 |
| 213700_s_at | *PKM* | pyruvate kinase, muscle | 2.78 | 0.00392 |
| 224425_x_at | *ACTR3BP2* | ARP3 actin-related protein 3 homolog B (yeast) pseudogene 2 | 2.78 | 0.02751 |
| 216351_x_at | *DAZ1* | deleted in azoospermia 1; deleted in azoospermia 2; deleted in azoospermia 3; deleted in azoospermia 4 | 2.77 | 0.01805 |
| 222856_at | *APLN* | apelin | 2.76 | 0.00044 |
| 213996_at | *YPEL1* | yippee-like 1 (Drosophila) | 2.76 | 0.00059 |
| 231590_at | *GATM* | glycine amidinotransferase (L-arginine:glycine amidinotransferase) | 2.76 | 0.00878 |
| 225391_at | *LOC93622* | Morf4 family associated protein 1-like 1 pseudogene | 2.75 | 4.3x10^-5^ |
| 210367_s_at | *PTGES* | prostaglandin E synthase | 2.75 | 0.00084 |
| 204698_at | *ISG20* | interferon stimulated exonuclease gene 20kDa | 2.74 | 0.00019 |
| 205015_s_at | *TGFA* | transforming growth factor, alpha | 2.74 | 0.00051 |
| 201294_s_at | *WSB1* | WD repeat and SOCS box containing 1 | 2.74 | 0.00239 |
| 206062_at | *GUCA1A* | guanylate cyclase activator 1A (retina) | 2.73 | 0.00029 |
| 202364_at | *MXI1* | MAX interactor 1, dimerization protein | 2.73 | 0.00142 |
| 211527_x_at | *VEGFA* | vascular endothelial growth factor A | 2.73 | 0.01264 |
| 202481_at | *DHRS3* | dehydrogenase/reductase (SDR family) member 3 | 2.72 | 0.00011 |
| 1570447_at | *LINC00452* | long intergenic non-protein coding RNA 452 | 2.72 | 0.00026 |
| 201008_s_at |  | thioredoxin-interacting protein-like; thioredoxin interacting protein | 2.72 | 0.00072 |
| 229626_at | *C12orf68* | chromosome 12 open reading frame 68 | 2.72 | 0.00277 |
| 205158_at | *RNASE4* | ribonuclease, RNase A family, 4 | 2.72 | 0.0039 |
| 202497_x_at | *SLC2A3* | solute carrier family 2 (facilitated glucose transporter), member 3 | 2.71 | 4.7x10^-5^ |
| 208729_x_at | *HLA-B* | major histocompatibility complex, class I, B | 2.71 | 0.0014 |
| 213270_at | *MPP2* | membrane protein, palmitoylated 2 (MAGUK p55 subfamily member 2) | 2.71 | 0.00299 |
| 1570329_at |  |  | 2.7 | 0.0031 |
| 238869_at |  |  | 2.7 | 0.00377 |
| 1558448_a_at |  |  | 2.7 | 0.02177 |
| 1564028_s_at | *FAM115C* | family with sequence similarity 115, member C; protein FAM115C-like; family with sequence similarity 115, member C pseudogene | 2.7 | 0.03032 |
| 239903_at | *TPBG* | trophoblast glycoprotein | 2.69 | 0.00009 |
| 205141_at | *ANG* | angiogenin, ribonuclease, RNase A family, 5 | 2.69 | 0.00125 |
| 203940_s_at | *VASH1* | vasohibin 1 | 2.69 | 0.0018 |
| 216882_s_at | *NEBL* | nebulette | 2.69 | 0.00183 |
| 235850_at | *FAM162A* | family with sequence similarity 162, member A | 2.69 | 0.00932 |
| 230085_at | *PDK3* | pyruvate dehydrogenase kinase, isozyme 3 | 2.68 | 0.00013 |
| 210513_s_at | *VEGFA* | vascular endothelial growth factor A | 2.68 | 0.00155 |
| 207292_s_at | *MAPK7* | mitogen-activated protein kinase 7 | 2.68 | 0.00202 |
| 201654_s_at | *HSPG2* | heparan sulfate proteoglycan 2 | 2.68 | 0.00826 |
| 242266_x_at |  |  | 2.68 | 0.00868 |
| 219958_at | *TMEM74B* | transmembrane protein 74B | 2.68 | 0.0181 |
| 1556465_at |  |  | 2.68 | 0.01969 |
| 238208_at |  |  | 2.68 | 0.03508 |
| 213113_s_at | *SLC43A3* | solute carrier family 43, member 3 | 2.67 | 0.00014 |
| 237585_at | *C4orf47* | chromosome 4 open reading frame 47 | 2.67 | 0.0002 |
| 227467_at | *RDH10* | retinol dehydrogenase 10 (all-trans) | 2.67 | 0.0003 |
| 1569206_at | *TCP11L2* | t-complex 11, testis-specific-like 2 | 2.67 | 0.0003 |
| 206801_at | *NPPB* | natriuretic peptide B | 2.67 | 0.0013 |
| 200085_s_at | *TCEB2* | transcription elongation factor B (SIII), polypeptide 2 (18kDa, elongin B) | 2.67 | 0.00817 |
| 213462_at | *NPAS2* | neuronal PAS domain protein 2 | 2.66 | 4.2x10^-5^ |
| 226542_at |  |  | 2.66 | 6.4x10^-5^ |
| 202718_at | *IGFBP2* | insulin-like growth factor binding protein 2, 36kDa | 2.66 | 0.00142 |
| 233280_at |  |  | 2.66 | 0.00153 |
| 210794_s_at | *MEG3* | maternally expressed 3 (non-protein coding) | 2.66 | 0.00474 |
| 211103_at | *MYO7A* | myosin VIIA | 2.66 | 0.0205 |
| 213089_at |  |  | 2.66 | 0.03779 |
| 210059_s_at | *MAPK13* | mitogen-activated protein kinase 13 | 2.65 | 0.00123 |
| 213345_at | *NFATC4* | nuclear factor of activated T-cells, cytoplasmic, calcineurin-dependent 4 | 2.65 | 0.00394 |
| 233664_at |  |  | 2.65 | 0.01426 |
| 215193_x_at | *HLA-DRB1* | major histocompatibility complex, class II, DR beta 1; major histocompatibility complex, class II, DR beta 3; major histocompatibility complex, class II, DR beta 4; HLA class II histocompatibility antigen, DRB1-7 beta chain-like | 2.64 | 0.00124 |
| 221236_s_at | *STMN4* | stathmin-like 4 | 2.64 | 0.00448 |
| 235968_at | *AGAP1* | ArfGAP with GTPase domain, ankyrin repeat and PH domain 1 | 2.63 | 0.00177 |
| 217807_s_at | *GLTSCR2* | glioma tumor suppressor candidate region gene 2; small nucleolar RNA, C/D box 23 | 2.63 | 0.0018 |
| 209324_s_at | *RGS16* | regulator of G-protein signaling 16 | 2.63 | 0.00236 |
| 236787_at |  |  | 2.63 | 0.00748 |
| 229296_at |  |  | 2.63 | 0.0085 |
| 215059_at |  |  | 2.62 | 0.00003 |
| 221524_s_at | *RRAGD* | Ras-related GTP binding D | 2.62 | 0.00292 |
| 211341_at | *POU4F1* | POU class 4 homeobox 1 | 2.62 | 0.00799 |
| 226632_at | *CYGB* | cytoglobin | 2.62 | 0.01002 |
| 1562222_at |  |  | 2.62 | 0.02512 |
| 240944_at |  |  | 2.62 | 0.03249 |
| 202855_s_at | *SLC16A3* | solute carrier family 16, member 3 (monocarboxylic acid transporter 4) | 2.61 | 0.00313 |
| 241980_at | *MAP6* | microtubule-associated protein 6 | 2.61 | 0.00476 |
| 204929_s_at | *VAMP5* | vesicle-associated membrane protein 5 | 2.61 | 0.00658 |
| 223276_at | *SMIM3* | small integral membrane protein 3 | 2.6 | 0.00025 |
| 231519_at |  |  | 2.6 | 0.00092 |
| 200075_s_at | *GUK1* | guanylate kinase 1 | 2.59 | 0.00351 |
| 219877_at | *ZMAT4* | zinc finger, matrin-type 4 | 2.58 | 7.3x10^-5^ |
| 209962_at | *EPOR* | erythropoietin receptor | 2.58 | 0.00054 |
| 236915_at | *C4orf47* | chromosome 4 open reading frame 47 | 2.58 | 0.00205 |
| 217054_at |  |  | 2.58 | 0.00317 |
| 243117_at |  |  | 2.58 | 0.00325 |
| 230319_at |  |  | 2.57 | 0.00045 |
| 215812_s_at |  | sodium- and chloride-dependent creatine transporter 1-like; solute carrier family 6 (neurotransmitter transporter, creatine), member 10, pseudogene; solute carrier family 6 (neurotransmitter transporter, creatine), member 8 | 2.57 | 0.00135 |
| 201169_s_at | *BHLHE40* | basic helix-loop-helix family, member e40 | 2.57 | 0.00579 |
| 224027_at | *CCL28* | chemokine (C-C motif) ligand 28 | 2.57 | 0.01918 |
| 228302_x_at | *CAMK2N1* | calcium/calmodulin-dependent protein kinase II inhibitor 1 | 2.57 | 0.02133 |
| 235781_at | *CACNA1B* | calcium channel, voltage-dependent, N type, alpha 1B subunit | 2.57 | 0.02164 |
| 209581_at | *PLA2G16* | phospholipase A2, group XVI | 2.56 | 0.00022 |
| 218451_at | *CDCP1* | CUB domain containing protein 1 | 2.56 | 0.00084 |
| 225573_at | *ACAD11* | acyl-CoA dehydrogenase family, member 11; nephronophthisis 3 (adolescent); NPHP3-ACAD11 readthrough | 2.56 | 0.01666 |
| 240385_at |  |  | 2.55 | 0.00075 |
| 230236_at | *TDRG1* | testis development related 1 (non-protein coding) | 2.55 | 0.00225 |
| 234339_s_at | *GLTSCR2* | glioma tumor suppressor candidate region gene 2; small nucleolar RNA, C/D box 23 | 2.55 | 0.00273 |
| 209325_s_at | *RGS16* | regulator of G-protein signaling 16 | 2.55 | 0.00293 |
| 212191_x_at | *RPL13* | ribosomal protein L13; small nucleolar RNA, C/D box 68 | 2.55 | 0.00454 |
| 244541_x_at |  |  | 2.55 | 0.02495 |
| 238385_at | *C6orf58* | chromosome 6 open reading frame 58 | 2.55 | 0.03715 |
| 214824_at |  |  | 2.54 | 0.00152 |
| 223169_s_at | *RHOU* | ras homolog family member U | 2.54 | 0.00265 |
| 241795_at | *RHEB* | Ras homolog enriched in brain | 2.54 | 0.00287 |
| 1556117_at |  |  | 2.54 | 0.00289 |
| 1568780_at |  |  | 2.54 | 0.02858 |
| 213148_at | *C2orf72* | chromosome 2 open reading frame 72 | 2.53 | 0.00078 |
| 219386_s_at | *SLAMF8* | SLAM family member 8 | 2.53 | 0.00135 |
| 227409_at | *PPP1R3E* | protein phosphatase 1, regulatory subunit 3E | 2.53 | 0.00167 |
| 228066_at | *C17orf96* | chromosome 17 open reading frame 96 | 2.53 | 0.00208 |
| 1559425_at |  |  | 2.53 | 0.00223 |
| 1564027_a_at | *FAM115C* | family with sequence similarity 115, member C; protein FAM115C-like; family with sequence similarity 115, member C pseudogene | 2.52 | 0.00036 |
| 230144_at | *GRIA3* | glutamate receptor, ionotropic, AMPA 3 | 2.52 | 0.00045 |
| 236709_at |  |  | 2.52 | 0.00131 |
| 202466_at | *PAPD7* | PAP associated domain containing 7 | 2.52 | 0.00213 |
| 228081_at | *CCNG2* | cyclin G2 | 2.52 | 0.00224 |
| 219425_at | *SULT4A1* | sulfotransferase family 4A, member 1 | 2.52 | 0.00294 |
| 203995_at | *C21orf2* | chromosome 21 open reading frame 2 | 2.52 | 0.0043 |
| 242632_at | *FGD2* | FYVE, RhoGEF and PH domain containing 2 | 2.52 | 0.02667 |
| 210706_s_at | *RNF24* | ring finger protein 24 | 2.51 | 0.00025 |
| 218731_s_at | *VWA1* | von Willebrand factor A domain containing 1 | 2.51 | 0.00051 |
| 218002_s_at | *CXCL14* | chemokine (C-X-C motif) ligand 14 | 2.51 | 0.00183 |
| 225797_at | *MRPL54* | mitochondrial ribosomal protein L54 | 2.51 | 0.02095 |
| 209182_s_at | *C10orf10* | chromosome 10 open reading frame 10 | 2.5 | 0.00012 |
| 219203_at | *EMC9* | ER membrane protein complex subunit 9 | 2.5 | 0.00075 |
| 235912_at |  |  | 2.5 | 0.00143 |
| 223610_at | *SEMA5B* | sema domain, seven thrombospondin repeats (type 1 and type 1-like), transmembrane domain (TM) and short cytoplasmic domain, (semaphorin) 5B | 2.5 | 0.00789 |
| 241846_at | *HCG18* | HLA complex group 18 (non-protein coding) | 2.5 | 0.01095 |
| 202839_s_at | *NDUFB7* | NADH dehydrogenase (ubiquinone) 1 beta subcomplex, 7, 18kDa | 2.5 | 0.04988 |
| 203839_s_at | *TNK2* | tyrosine kinase, non-receptor, 2 | 2.49 | 0.00144 |
| 209312_x_at | *HLA-DRB1* | major histocompatibility complex, class II, DR beta 1; major histocompatibility complex, class II, DR beta 4; major histocompatibility complex, class II, DR beta 5; HLA class II histocompatibility antigen, DRB1-7 beta chain-like | 2.49 | 0.00201 |
| 213201_s_at | *TNNT1* | troponin T type 1 (skeletal, slow) | 2.49 | 0.00215 |
| 213988_s_at | *SAT1* | spermidine/spermine N1-acetyltransferase 1 | 2.49 | 0.00285 |
| 240665_at |  |  | 2.49 | 0.00302 |
| 206424_at | *CYP26A1* | cytochrome P450, family 26, subfamily A, polypeptide 1 | 2.49 | 0.00321 |
| 229340_at | *NKAPL* | NFKB activating protein-like | 2.49 | 0.00355 |
| 203108_at | *GPRC5A* | G protein-coupled receptor, family C, group 5, member A | 2.49 | 0.00586 |
| 235616_at | *TSHZ2* | teashirt zinc finger homeobox 2 | 2.49 | 0.00652 |
| 235672_at | *MAP6* | microtubule-associated protein 6 | 2.49 | 0.00799 |
| 217339_x_at | *CTAG1A* | cancer/testis antigen 1A; cancer/testis antigen 1B | 2.49 | 0.00918 |
| 232002_at |  |  | 2.49 | 0.01349 |
| 242479_s_at | *MCM4* | minichromosome maintenance complex component 4 | 2.49 | 0.01556 |
| 205081_at | *CRIP1* | cysteine-rich protein 1 (intestinal) | 2.49 | 0.02073 |
| 244509_at | *GPR155* | G protein-coupled receptor 155 | 2.48 | 5.8x10^-5^ |
| 205249_at | *EGR2* | early growth response 2 | 2.48 | 0.00055 |
| 211695_x_at | *MUC1* | mucin 1, cell surface associated | 2.48 | 0.00091 |
| 232968_at | *FANK1* | fibronectin type III and ankyrin repeat domains 1 | 2.48 | 0.00184 |
| 233457_at |  |  | 2.48 | 0.00255 |
| 220795_s_at | *BEGAIN* | brain-enriched guanylate kinase-associated | 2.48 | 0.0187 |
| 228866_at |  |  | 2.48 | 0.02229 |
| 209360_s_at |  |  | 2.47 | 1x10^-6^ |
| 213486_at | *COPG2IT1* | COPG2 imprinted transcript 1 (non-protein coding) | 2.47 | 0.00031 |
| 230267_at |  |  | 2.47 | 0.0012 |
| 241669_x_at | *PRKD2* | protein kinase D2 | 2.47 | 0.00149 |
| 234495_at | *KLK15* | kallikrein-related peptidase 15 | 2.47 | 0.0027 |
| 212496_s_at | *KDM4B* | lysine (K)-specific demethylase 4B | 2.47 | 0.00328 |
| 223454_at | *CXCL16* | chemokine (C-X-C motif) ligand 16 | 2.47 | 0.00329 |
| 239968_at | *LINC00313* | long intergenic non-protein coding RNA 313 | 2.47 | 0.00703 |
| 235419_at |  |  | 2.47 | 0.01019 |
| 202887_s_at | *DDIT4* | DNA-damage-inducible transcript 4 | 2.46 | 0.00048 |
| 203192_at | *ABCB6* | ATP-binding cassette, sub-family B (MDR/TAP), member 6 | 2.46 | 0.00089 |
| 206620_at | *GRAP* | GRB2-related adaptor protein | 2.46 | 0.00281 |
| 207987_s_at | *GNRH1* | gonadotropin-releasing hormone 1 (luteinizing-releasing hormone) | 2.46 | 0.00372 |
| 229390_at | *FAM26F* | family with sequence similarity 26, member F | 2.46 | 0.00769 |
| 206732_at | *SLITRK3* | SLIT and NTRK-like family, member 3 | 2.46 | 0.02109 |
| 219722_s_at | *GDPD3* | glycerophosphodiester phosphodiesterase domain containing 3 | 2.46 | 0.02247 |
| 212242_at | *TUBA4A* | tubulin, alpha 4a | 2.45 | 0.00021 |
| 228943_at | *MAP6* | microtubule-associated protein 6 | 2.45 | 0.00077 |
| 214471_x_at | *LHB* | luteinizing hormone beta polypeptide | 2.45 | 0.00114 |
| 1562529_s_at |  |  | 2.45 | 0.00154 |
| 209304_x_at | *GADD45B* | growth arrest and DNA-damage-inducible, beta | 2.45 | 0.00218 |
| 226677_at | *ZNF521* | zinc finger protein 521 | 2.45 | 0.00325 |
| 243297_at |  |  | 2.45 | 0.00373 |
| 214208_at | *KLHL35* | kelch-like family member 35 | 2.45 | 0.00569 |
| 213527_s_at | *ZNF688* | zinc finger protein 688 | 2.45 | 0.01652 |
| 202935_s_at | *SOX9* | SRY (sex determining region Y)-box 9 | 2.44 | 0.00021 |
| 1563606_a_at | *LOC286359* | uncharacterized LOC286359 | 2.44 | 0.00911 |
| 217616_at |  |  | 2.44 | 0.01199 |
| 236114_at |  |  | 2.44 | 0.03383 |
| 202524_s_at | *SPOCK2* | sparc/osteonectin, cwcv and kazal-like domains proteoglycan (testican) 2 | 2.43 | 0.00023 |
| 230531_at | *KCNC3* | potassium voltage-gated channel, Shaw-related subfamily, member 3 | 2.43 | 0.00039 |
| 59625_at | *NOL3* | nucleolar protein 3 (apoptosis repressor with CARD domain) | 2.43 | 0.00102 |
| 218977_s_at | *TRNAU1AP* | tRNA selenocysteine 1 associated protein 1 | 2.43 | 0.0039 |
| 220425_x_at | *ROPN1B* | rhophilin associated tail protein 1B | 2.43 | 0.00529 |
| 222072_at | *ADD3-AS1* | ADD3 antisense RNA 1 | 2.42 | 1.9x10^-5^ |
| 205774_at | *F12* | coagulation factor XII (Hageman factor) | 2.42 | 5.2x10^-5^ |
| 226870_at | *COMTD1* | catechol-O-methyltransferase domain containing 1 | 2.42 | 0.00185 |
| 238669_at | *PTGS1* | prostaglandin-endoperoxide synthase 1 (prostaglandin G/H synthase and cyclooxygenase) | 2.42 | 0.00254 |
| 230228_at | *SSC5D* | scavenger receptor cysteine rich domain containing (5 domains) | 2.42 | 0.00302 |
| 226452_at | *PDK1* | pyruvate dehydrogenase kinase, isozyme 1 | 2.42 | 0.00337 |
| 210454_s_at | *KCNJ6* | potassium inwardly-rectifying channel, subfamily J, member 6 | 2.42 | 0.00349 |
| 233128_at |  |  | 2.42 | 0.00398 |
| 234925_at | *LINC00917* | long intergenic non-protein coding RNA 917 | 2.42 | 0.00415 |
| 219348_at | *USE1* | unconventional SNARE in the ER 1 homolog (S. cerevisiae) | 2.42 | 0.01568 |
| 230143_at | *RNF165* | ring finger protein 165 | 2.42 | 0.01936 |
| 228690_s_at | *NDUFA11* | NADH dehydrogenase (ubiquinone) 1 alpha subcomplex, 11, 14.7kDa | 2.42 | 0.03348 |
| 239315_at | *FAM115C* | family with sequence similarity 115, member C; protein FAM115C-like | 2.41 | 0.00024 |
| 209732_at | *CLEC2B* | C-type lectin domain family 2, member B | 2.41 | 0.00042 |
| 205212_s_at | *ACAP1* | ArfGAP with coiled-coil, ankyrin repeat and PH domains 1 | 2.41 | 0.00058 |
| 239446_x_at | *DCBLD2* | discoidin, CUB and LCCL domain containing 2 | 2.41 | 0.00092 |
| 211911_x_at | *HLA-B* | major histocompatibility complex, class I, B | 2.41 | 0.00092 |
| 213940_s_at | *FNBP1* | formin binding protein 1 | 2.41 | 0.00196 |
| 208277_at | *PITX3* | paired-like homeodomain 3 | 2.41 | 0.00578 |
| 243893_at | *KCNC3* | potassium voltage-gated channel, Shaw-related subfamily, member 3 | 2.41 | 0.00682 |
| 207184_at | *SLC6A13* | solute carrier family 6 (neurotransmitter transporter, GABA), member 13 | 2.41 | 0.00806 |
| 224491_at | *APOL4* | apolipoprotein L, 4 | 2.41 | 0.02279 |
| 208608_s_at | *SNTB1* | syntrophin, beta 1 (dystrophin-associated protein A1, 59kDa, basic component 1) | 2.41 | 0.04774 |
| 218498_s_at | *ERO1L* | ERO1-like (S. cerevisiae) | 2.4 | 0.00017 |
| 201465_s_at | *JUN* | jun proto-oncogene | 2.4 | 0.00103 |
| 219694_at | *FAM105A* | family with sequence similarity 105, member A | 2.4 | 0.00491 |
| 234431_at | *GSN* | gelsolin | 2.4 | 0.00585 |
| 1564836_at |  |  | 2.4 | 0.00845 |
| 238916_at | *LINC00938* | long intergenic non-protein coding RNA 938 | 2.4 | 0.00876 |
| 235257_at | *ODF3B* | outer dense fiber of sperm tails 3B | 2.4 | 0.01271 |
| 1566232_at |  |  | 2.39 | 0.00192 |
| 220611_at | *DAB1* | Dab, reelin signal transducer, homolog 1 (Drosophila) | 2.39 | 0.0031 |
| 228738_at | *D2HGDH* | D-2-hydroxyglutarate dehydrogenase | 2.39 | 0.00581 |
| 238091_at |  |  | 2.39 | 0.01352 |
| 242872_at | *CIT* | citron (rho-interacting, serine/threonine kinase 21); microRNA 1178 | 2.39 | 0.01998 |
| 230674_at | *LGR4* | leucine-rich repeat containing G protein-coupled receptor 4 | 2.39 | 0.0263 |
| 212843_at | *NCAM1* | neural cell adhesion molecule 1 | 2.38 | 0.00021 |
| 213338_at | *TMEM158* | transmembrane protein 158 (gene/pseudogene) | 2.38 | 0.00027 |
| 209598_at | *PNMA2* | paraneoplastic Ma antigen 2 | 2.38 | 0.00109 |
| 230402_at | *DUSP15* | dual specificity phosphatase 15 | 2.38 | 0.00262 |
| 237425_at | *SORCS3-AS1* | SORCS3 antisense RNA 1 | 2.38 | 0.0034 |
| 228720_at | *SORCS2* | sortilin-related VPS10 domain containing receptor 2 | 2.38 | 0.00544 |
| 206777_s_at | *CRYBB2* | crystallin, beta B2; crystallin, beta B2 pseudogene 1 | 2.38 | 0.00618 |
| 1569263_at |  |  | 2.38 | 0.01535 |
| 223447_at | *REG4* | regenerating islet-derived family, member 4 | 2.38 | 0.0174 |
| 35617_at | *MAPK7* | mitogen-activated protein kinase 7 | 2.37 | 0.0001 |
| 207521_s_at | *ATP2A3* | ATPase, Ca++ transporting, ubiquitous | 2.37 | 0.00277 |
| 222508_s_at | *ARGLU1* | arginine and glutamate rich 1 | 2.37 | 0.003 |
| 226824_at | *CPXM2* | carboxypeptidase X (M14 family), member 2 | 2.37 | 0.00305 |
| 206782_s_at | *DNAJC4* | DnaJ (Hsp40) homolog, subfamily C, member 4 | 2.37 | 0.00351 |
| 205577_at | *PYGM* | phosphorylase, glycogen, muscle | 2.37 | 0.00494 |
| 235317_at |  |  | 2.37 | 0.00496 |
| 207756_at |  |  | 2.37 | 0.005 |
| 231982_at | *C19orf77* | chromosome 19 open reading frame 77 | 2.37 | 0.00651 |
| 218921_at | *SIGIRR* | single immunoglobulin and toll-interleukin 1 receptor (TIR) domain | 2.37 | 0.00703 |
| 211620_x_at |  |  | 2.37 | 0.01238 |
| 231242_at | *BHLHE41* | basic helix-loop-helix family, member e41 | 2.37 | 0.01324 |
| 224273_at | *C3orf20* | chromosome 3 open reading frame 20 | 2.37 | 0.01645 |
| 205902_at | *KCNN3* | potassium intermediate/small conductance calcium-activated channel, subfamily N, member 3 | 2.37 | 0.018 |
| 241525_at |  |  | 2.37 | 0.02632 |
| 1565525_a_at | *TCP11L2* | t-complex 11, testis-specific-like 2 | 2.37 | 0.03802 |
| 1557470_at | *SPATA13* | spermatogenesis associated 13 | 2.36 | 0.00027 |
| 1561375_at |  |  | 2.36 | 0.00036 |
| 213630_at | *NACAD* | NAC alpha domain containing | 2.36 | 0.00041 |
| 219315_s_at | *TMEM204* | transmembrane protein 204 | 2.36 | 0.00086 |
| 221013_s_at | *APOL2* | apolipoprotein L, 2 | 2.36 | 0.00297 |
| 209559_at | *HIP1R* | huntingtin interacting protein 1 related | 2.36 | 0.00451 |
| 219014_at | *PLAC8* | placenta-specific 8 | 2.36 | 0.00471 |
| 205603_s_at | *DIAPH2* | diaphanous-related formin 2 | 2.36 | 0.00538 |
| 208078_s_at | *SIK1* | salt-inducible kinase 1 | 2.36 | 0.00666 |
| 1569372_at |  |  | 2.36 | 0.00903 |
| 1559491_at |  |  | 2.36 | 0.02353 |
| 236562_at | *ZNF439* | zinc finger protein 439 | 2.35 | 0.00016 |
| 228284_at | *TLE1* | transducin-like enhancer of split 1 (E(sp1) homolog, Drosophila) | 2.35 | 0.00121 |
| 236266_at | *RORA* | RAR-related orphan receptor A | 2.35 | 0.00154 |
| 218687_s_at | *MUC13* | mucin 13, cell surface associated | 2.35 | 0.0018 |
| 213640_s_at | *LOX* | lysyl oxidase | 2.35 | 0.00208 |
| 225683_x_at | *PHPT1* | phosphohistidine phosphatase 1 | 2.35 | 0.00712 |
| 207353_s_at | *HMX1* | H6 family homeobox 1 | 2.35 | 0.00774 |
| 230301_at |  |  | 2.35 | 0.01577 |
| 237335_at | *ZP1* | zona pellucida glycoprotein 1 (sperm receptor) | 2.34 | 0.00127 |
| 222655_s_at | *IMPAD1* | inositol monophosphatase domain containing 1 | 2.34 | 0.00246 |
| 229047_at | *PLEKHB1* | pleckstrin homology domain containing, family B (evectins) member 1 | 2.34 | 0.003 |
| 211809_x_at | *COL13A1* | collagen, type XIII, alpha 1 | 2.34 | 0.00799 |
| 223929_s_at |  |  | 2.34 | 0.01251 |
| 206791_s_at |  |  | 2.34 | 0.01548 |
| 1570627_at | *TCEB3* | transcription elongation factor B (SIII), polypeptide 3 (110kDa, elongin A) | 2.34 | 0.02154 |
| 1553962_s_at | *RHOB* | ras homolog family member B | 2.34 | 0.02258 |
| 232342_at | *MTMR14* | myotubularin related protein 14 | 2.34 | 0.04279 |
| 200737_at | *PGK1* | phosphoglycerate kinase 1 | 2.33 | 0.00013 |
| 232134_at |  |  | 2.33 | 0.00039 |
| 242525_at | *SLC2A5* | solute carrier family 2 (facilitated glucose/fructose transporter), member 5 | 2.33 | 0.00143 |
| 216552_x_at | *KIR2DS4* | killer cell immunoglobulin-like receptor, two domains, short cytoplasmic tail, 4 | 2.33 | 0.00157 |
| 231243_s_at | *BHLHE41* | basic helix-loop-helix family, member e41 | 2.33 | 0.0041 |
| 241285_at |  |  | 2.33 | 0.00677 |
| 230839_at | *PRMT8* | protein arginine methyltransferase 8 | 2.33 | 0.00784 |
| 244583_at |  |  | 2.33 | 0.01565 |
| 212899_at | *CDK19* | cyclin-dependent kinase 19 | 2.32 | 1.6x10^-5^ |
| 219033_at | *PARP8* | poly (ADP-ribose) polymerase family, member 8 | 2.32 | 3.6x10^-5^ |
| 212703_at | *TLN2* | talin 2 | 2.32 | 0.00198 |
| 221497_x_at | *EGLN1* | egl nine homolog 1 (C. elegans) | 2.32 | 0.00206 |
| 225757_s_at | *CLMN* | calmin (calponin-like, transmembrane) | 2.32 | 0.00213 |
| 225839_at | *RBM33* | RNA binding motif protein 33 | 2.32 | 0.0022 |
| 222723_at | *VWA1* | von Willebrand factor A domain containing 1 | 2.32 | 0.00292 |
| 204756_at | *MAP2K5* | mitogen-activated protein kinase kinase 5 | 2.32 | 0.00975 |
| 230498_at | *MCHR1* | melanin-concentrating hormone receptor 1 | 2.32 | 0.01422 |
| 240669_at |  |  | 2.32 | 0.01563 |
| 1559991_s_at |  |  | 2.32 | 0.02866 |
| 230555_s_at | *MED30* | mediator complex subunit 30 | 2.32 | 0.036 |
| 208798_x_at | *GOLGA8A* | golgin A8 family, member A | 2.31 | 0.00037 |
| 208177_at | *SLC34A1* | solute carrier family 34 (sodium phosphate), member 1 | 2.31 | 0.0007 |
| 217691_x_at | *SLC16A3* | solute carrier family 16, member 3 (monocarboxylic acid transporter 4) | 2.31 | 0.00099 |
| 206396_at | *SLC1A1* | solute carrier family 1 (neuronal/epithelial high affinity glutamate transporter, system Xag), member 1 | 2.31 | 0.00286 |
| 207271_x_at | *SPAG11B* | sperm associated antigen 11B | 2.31 | 0.00291 |
| 244176_at |  |  | 2.31 | 0.00338 |
| 216093_at |  |  | 2.31 | 0.00414 |
| 235055_x_at | *MUC4* | mucin 4, cell surface associated | 2.31 | 0.00669 |
| 38710_at | *OTUB1* | OTU domain, ubiquitin aldehyde binding 1 | 2.31 | 0.01902 |
| 1553009_s_at | *REXO1L1* | REX1, RNA exonuclease 1 homolog (S. cerevisiae)-like 1 | 2.31 | 0.03228 |
| 234345_at |  |  | 2.31 | 0.03287 |
| 211102_s_at | *LILRA2* | leukocyte immunoglobulin-like receptor, subfamily A (with TM domain), member 2 | 2.3 | 0.00011 |
| 229898_at | *SNX33* | sorting nexin 33 | 2.3 | 0.00012 |
| 214775_at | *N4BP3* | NEDD4 binding protein 3 | 2.3 | 0.00024 |
| 243633_at |  |  | 2.3 | 0.00158 |
| 222124_at | *HIF3A* | hypoxia inducible factor 3, alpha subunit | 2.3 | 0.00277 |
| 219766_at | *B9D2* | B9 protein domain 2 | 2.3 | 0.00303 |
| 226462_at | *STXBP6* | syntaxin binding protein 6 (amisyn) | 2.3 | 0.00415 |
| 211700_s_at | *TRO* | trophinin | 2.3 | 0.00472 |
| 236884_at | *RIMKLA* | ribosomal modification protein rimK-like family member A | 2.3 | 0.00675 |
| 217728_at | *S100A6* | S100 calcium binding protein A6 | 2.3 | 0.00755 |
| 1552409_a_at | *ODF4* | outer dense fiber of sperm tails 4 | 2.3 | 0.00756 |
| 235377_at | *MLIP* | muscular LMNA-interacting protein | 2.3 | 0.00786 |
| 202030_at | *BCKDK* | branched chain ketoacid dehydrogenase kinase | 2.3 | 0.0093 |
| 221363_x_at | *GPR25* | G protein-coupled receptor 25 | 2.3 | 0.01129 |
| 205460_at | *NPAS2* | neuronal PAS domain protein 2 | 2.3 | 0.01698 |
| 217238_s_at | *ALDOB* | aldolase B, fructose-bisphosphate | 2.3 | 0.03531 |
| 243409_at | *FOXL1* | forkhead box L1 | 2.29 | 0.00019 |
| 204990_s_at | *ITGB4* | integrin, beta 4 | 2.29 | 0.00022 |
| 215715_at | *SLC6A2* | solute carrier family 6 (neurotransmitter transporter, noradrenalin), member 2 | 2.29 | 0.00024 |
| 240517_at | *CBS* | cystathionine-beta-synthase | 2.29 | 0.00073 |
| 237646_x_at | *PLEKHG5* | pleckstrin homology domain containing, family G (with RhoGef domain) member 5 | 2.29 | 0.00152 |
| 232290_at |  |  | 2.29 | 0.00203 |
| 222334_at | *C1orf186* | chromosome 1 open reading frame 186; uncharacterized LOC100505650 | 2.29 | 0.00301 |
| 208929_x_at | *RPL13* | ribosomal protein L13; small nucleolar RNA, C/D box 68 | 2.29 | 0.00456 |
| 238926_at |  |  | 2.29 | 0.02402 |
| 208179_x_at | *KIR2DL3* | killer cell immunoglobulin-like receptor, two domains, long cytoplasmic tail, 3 | 2.29 | 0.03011 |
| 244864_at |  |  | 2.28 | 6.8x10^-5^ |
| 243616_at |  |  | 2.28 | 0.00027 |
| 230368_at | *ERF* | Ets2 repressor factor | 2.28 | 0.00058 |
| 217032_at | *FOXD4* | forkhead box D4; forkhead box D4-like 1 | 2.28 | 0.00081 |
| 234359_at |  |  | 2.28 | 0.00105 |
| 231322_at |  |  | 2.28 | 0.00137 |
| 236848_s_at | *TEX13A* | testis expressed 13A | 2.28 | 0.00155 |
| 206811_at | *ADCY8* | adenylate cyclase 8 (brain) | 2.28 | 0.00208 |
| 215367_at | *KIAA1614* | KIAA1614 | 2.28 | 0.00216 |
| 205309_at | *SMPDL3B* | sphingomyelin phosphodiesterase, acid-like 3B | 2.28 | 0.00308 |
| 238042_at |  |  | 2.28 | 0.00322 |
| 223801_s_at | *APOL4* | apolipoprotein L, 4 | 2.28 | 0.00388 |
| 240377_at |  |  | 2.28 | 0.00409 |
| 200635_s_at | *PTPRF* | protein tyrosine phosphatase, receptor type, F | 2.28 | 0.00572 |
| 205103_at | *C1orf61* | chromosome 1 open reading frame 61 | 2.28 | 0.03269 |
| 215503_at | *SPINT3* | serine peptidase inhibitor, Kunitz type, 3 | 2.28 | 0.03278 |
| 33304_at | *ISG20* | interferon stimulated exonuclease gene 20kDa | 2.27 | 0.00026 |
| 235533_at | *COX19* | cytochrome c oxidase assembly homolog 19 (S. cerevisiae) | 2.27 | 0.00126 |
| 230341_x_at | *ADAMTS10* | ADAM metallopeptidase with thrombospondin type 1 motif, 10 | 2.27 | 0.00189 |
| 214781_at |  |  | 2.27 | 0.00196 |
| 222216_s_at | *MRPL17* | mitochondrial ribosomal protein L17 | 2.27 | 0.00203 |
| 210340_s_at | *CSF2RA* | colony stimulating factor 2 receptor, alpha, low-affinity (granulocyte-macrophage) | 2.27 | 0.00234 |
| 210461_s_at | *ABLIM1* | actin binding LIM protein 1 | 2.27 | 0.00276 |
| 234003_at | *ENOX2* | ecto-NOX disulfide-thiol exchanger 2 | 2.27 | 0.00308 |
| 215273_s_at | *TADA3* | transcriptional adaptor 3 | 2.27 | 0.00419 |
| 243749_s_at |  |  | 2.27 | 0.00488 |
| 238267_s_at |  |  | 2.27 | 0.00539 |
| 214676_x_at | *MUC3A* | mucin 3A, cell surface associated; mucin 3B, cell surface associated | 2.27 | 0.00601 |
| 206763_at | *FKBP6* | FK506 binding protein 6, 36kDa | 2.27 | 0.00642 |
| 202208_s_at | *ARL4C* | ADP-ribosylation factor-like 4C | 2.27 | 0.00657 |
| 205832_at | *CPA4* | carboxypeptidase A4 | 2.27 | 0.00819 |
| 209597_s_at | *PNMA2* | paraneoplastic Ma antigen 2 | 2.27 | 0.02025 |
| 223937_at | *FOXP1* | forkhead box P1 | 2.27 | 0.03774 |
| 221097_s_at | *KCNMB2* | potassium large conductance calcium-activated channel, subfamily M, beta member 2 | 2.27 | 0.03819 |
| 213942_at | *MEGF6* | multiple EGF-like-domains 6 | 2.26 | 6.4x10^-5^ |
| 219395_at | *ESRP2* | epithelial splicing regulatory protein 2 | 2.26 | 0.00037 |
| 204465_s_at | *INA* | internexin neuronal intermediate filament protein, alpha | 2.26 | 0.00059 |
| 230430_at | *ENTPD2* | ectonucleoside triphosphate diphosphohydrolase 2 | 2.26 | 0.0009 |
| 206404_at | *FGF9* | fibroblast growth factor 9 (glia-activating factor) | 2.26 | 0.00124 |
| 229282_at | *GATA6* | GATA binding protein 6 | 2.26 | 0.00168 |
| 211813_x_at | *DCN* | decorin | 2.26 | 0.00229 |
| 1562320_at | *NAV2-AS5* | NAV2 antisense RNA 5 | 2.26 | 0.00296 |
| 209302_at | *POLR2H* | polymerase (RNA) II (DNA directed) polypeptide H | 2.26 | 0.00362 |
| 235567_at | *RORA* | RAR-related orphan receptor A | 2.26 | 0.00423 |
| 229880_at |  |  | 2.26 | 0.00448 |
| 239643_at |  |  | 2.26 | 0.00453 |
| 206658_at | *UPK3B* | uroplakin 3B | 2.26 | 0.00513 |
| 237986_at |  |  | 2.26 | 0.00541 |
| 236482_at |  |  | 2.26 | 0.00569 |
| 204669_s_at | *RNF24* | ring finger protein 24 | 2.26 | 0.00648 |
| 202796_at | *SYNPO* | synaptopodin | 2.26 | 0.00786 |
| 243099_at | *NFAM1* | NFAT activating protein with ITAM motif 1 | 2.26 | 0.01301 |
| 240493_at |  |  | 2.26 | 0.01626 |
| 224972_at | *ROMO1* | reactive oxygen species modulator 1 | 2.26 | 0.01879 |
| 216010_x_at | *FUT3* | fucosyltransferase 3 (galactoside 3(4)-L-fucosyltransferase, Lewis blood group) | 2.26 | 0.03156 |
| 235252_at | *KSR1* | kinase suppressor of ras 1 | 2.25 | 0.00055 |
| 231963_at | *ANKRD33B* | ankyrin repeat domain 33B | 2.25 | 0.00063 |
| 223460_at | *CAMKK1* | calcium/calmodulin-dependent protein kinase kinase 1, alpha | 2.25 | 0.00097 |
| 203986_at | *FAM47E* | family with sequence similarity 47, member E; FAM47E-STBD1 readthrough; starch binding domain 1 | 2.25 | 0.001 |
| 1552296_at | *BEST4* | bestrophin 4 | 2.25 | 0.00118 |
| 210889_s_at | *FCGR2B* | Fc fragment of IgG, low affinity IIb, receptor (CD32) | 2.25 | 0.00245 |
| 230851_x_at | *C16orf13* | chromosome 16 open reading frame 13 | 2.25 | 0.00268 |
| 210792_x_at | *SIVA1* | SIVA1, apoptosis-inducing factor | 2.25 | 0.00268 |
| 224464_s_at | *NUDT22* | nudix (nucleoside diphosphate linked moiety X)-type motif 22 | 2.25 | 0.00301 |
| 223389_s_at | *ZNF581* | zinc finger protein 581 | 2.25 | 0.00351 |
| 210855_at | *GREB1* | growth regulation by estrogen in breast cancer 1 | 2.25 | 0.00383 |
| 209299_x_at | *PPIL2* | peptidylprolyl isomerase (cyclophilin)-like 2 | 2.25 | 0.00713 |
| 217112_at | *PDGFB* | platelet-derived growth factor beta polypeptide | 2.25 | 0.00942 |
| 214624_at | *UPK1A* | uroplakin 1A | 2.25 | 0.00968 |
| 226453_at | *RNASEH2C* | ribonuclease H2, subunit C | 2.25 | 0.01033 |
| 235911_at | *MFI2* | antigen p97 (melanoma associated) identified by monoclonal antibodies 133.2 and 96.5 | 2.25 | 0.01196 |
| 222045_s_at | *PCIF1* | PDX1 C-terminal inhibiting factor 1 | 2.25 | 0.01325 |
| 211025_x_at | *COX5B* | cytochrome c oxidase subunit Vb | 2.25 | 0.01906 |
| 244453_at | *ANKRD53* | ankyrin repeat domain 53 | 2.25 | 0.0285 |
| 210867_at | *CNOT4* | CCR4-NOT transcription complex, subunit 4 | 2.24 | 0.00045 |
| 224003_at |  |  | 2.24 | 0.00094 |
| 211286_x_at | *CSF2RA* | colony stimulating factor 2 receptor, alpha, low-affinity (granulocyte-macrophage) | 2.24 | 0.00114 |
| 206071_s_at | *EPHA3* | EPH receptor A3 | 2.24 | 0.00413 |
| 227185_at | *C1orf233* | chromosome 1 open reading frame 233 | 2.24 | 0.0046 |
| 208763_s_at | *TSC22D3* | TSC22 domain family, member 3 | 2.24 | 0.00461 |
| 203792_x_at | *PCGF2* | polycomb group ring finger 2 | 2.24 | 0.00483 |
| 206070_s_at | *EPHA3* | EPH receptor A3 | 2.24 | 0.01122 |
| 201452_at | *RHEB* | Ras homolog enriched in brain | 2.24 | 0.01129 |
| 203186_s_at | *S100A4* | S100 calcium binding protein A4 | 2.24 | 0.01234 |
| 228869_at | *SNX20* | sorting nexin 20 | 2.24 | 0.0182 |
| 237909_at | *ADAM6* | ADAM metallopeptidase domain 6, pseudogene | 2.24 | 0.02385 |
| 239990_at |  |  | 2.24 | 0.02904 |
| 1557689_at |  |  | 2.24 | 0.03947 |
| 229411_at | *PNCK* | pregnancy up-regulated non-ubiquitously expressed CaM kinase | 2.23 | 6.2x10^-5^ |
| 202023_at | *EFNA1* | ephrin-A1 | 2.23 | 0.00044 |
| 226571_s_at | *PTPRS* | protein tyrosine phosphatase, receptor type, S | 2.23 | 0.00196 |
| 227781_x_at | *FAM57B* | family with sequence similarity 57, member B; uncharacterized LOC100996332 | 2.23 | 0.00247 |
| 222857_s_at | *KCNMB4* | potassium large conductance calcium-activated channel, subfamily M, beta member 4 | 2.23 | 0.00328 |
| 215144_at |  |  | 2.23 | 0.00348 |
| 221886_at | *DENND2A* | DENN/MADD domain containing 2A | 2.23 | 0.00424 |
| 219966_x_at | *BANP* | BTG3 associated nuclear protein | 2.23 | 0.0048 |
| 216927_at | *MAU2* | MAU2 chromatid cohesion factor homolog (C. elegans) | 2.23 | 0.00702 |
| 216345_at | *ZSWIM8* | zinc finger, SWIM-type containing 8 | 2.23 | 0.00738 |
| 216949_s_at |  |  | 2.23 | 0.00954 |
| 228880_at | *NAT8L* | N-acetyltransferase 8-like (GCN5-related, putative) | 2.23 | 0.01006 |
| 219061_s_at | *LAGE3* | L antigen family, member 3 | 2.23 | 0.01739 |
| 217818_s_at | *ARPC4* | actin related protein 2/3 complex, subunit 4, 20kDa | 2.23 | 0.02025 |
| 217871_s_at | *MIF* | macrophage migration inhibitory factor (glycosylation-inhibiting factor) | 2.23 | 0.0285 |
| 204631_at | *MYH2* | myosin, heavy chain 2, skeletal muscle, adult | 2.22 | 2x10^-6^ |
| 236599_at |  |  | 2.22 | 0.0002 |
| 235245_at | *TMEM92* | transmembrane protein 92 | 2.22 | 0.00022 |
| 218533_s_at | *UCKL1* | uridine-cytidine kinase 1-like 1 | 2.22 | 0.00034 |
| 241038_at |  |  | 2.22 | 0.00162 |
| 208232_x_at | *NRG1* | neuregulin 1 | 2.22 | 0.00162 |
| 214977_at |  |  | 2.22 | 0.00416 |
| 219897_at | *RNF122* | ring finger protein 122 | 2.22 | 0.00423 |
| 221959_at | *FAM110B* | family with sequence similarity 110, member B | 2.22 | 0.0064 |
| 227352_at | *SWSAP1* | SWIM-type zinc finger 7 associated protein 1 | 2.22 | 0.01003 |
| 217278_x_at |  |  | 2.22 | 0.01023 |
| 1558540_s_at | *SLC2A11* | solute carrier family 2 (facilitated glucose transporter), member 11 | 2.22 | 0.0126 |
| 231233_at | *PCAT6* | prostate cancer associated transcript 6 (non-protein coding) | 2.21 | 0.0002 |
| 217674_at |  |  | 2.21 | 0.00038 |
| 243777_at | *RAB7L1* | RAB7, member RAS oncogene family-like 1 | 2.21 | 0.00094 |
| 201137_s_at | *HLA-DPB1* | major histocompatibility complex, class II, DP beta 1 | 2.21 | 0.00095 |
| 229583_at |  |  | 2.21 | 0.00143 |
| 231998_at | *SART1* | squamous cell carcinoma antigen recognized by T cells | 2.21 | 0.00233 |
| 218638_s_at |  |  | 2.21 | 0.00273 |
| 203641_s_at | *COBLL1* | cordon-bleu WH2 repeat protein-like 1 | 2.21 | 0.00298 |
| 202210_x_at | *GSK3A* | glycogen synthase kinase 3 alpha | 2.21 | 0.00338 |
| 202996_at | *POLD4* | polymerase (DNA-directed), delta 4, accessory subunit | 2.21 | 0.00464 |
| 1568706_s_at | *PP12719* | uncharacterized LOC100653022 | 2.21 | 0.0054 |
| 216706_x_at | *IGHD3-16* | immunoglobulin heavy diversity 3-16; NULL | 2.21 | 0.00556 |
| 219692_at | *KREMEN2* | kringle containing transmembrane protein 2 | 2.21 | 0.00622 |
| 204670_x_at | *HLA-DRB1* | major histocompatibility complex, class II, DR beta 1; major histocompatibility complex, class II, DR beta 4; HLA class II histocompatibility antigen, DRB1-7 beta chain-like | 2.21 | 0.00651 |
| 211457_at | *GABARAPL3* | GABA(A) receptors associated protein like 3, pseudogene | 2.21 | 0.00663 |
| 221525_at | *ZMIZ2* | zinc finger, MIZ-type containing 2 | 2.21 | 0.00975 |
| 214986_x_at | *PPIL2* | peptidylprolyl isomerase (cyclophilin)-like 2 | 2.21 | 0.01049 |
| 223923_at | *C21orf62* | chromosome 21 open reading frame 62 | 2.21 | 0.01125 |
| 223621_at | *PNMA3* | paraneoplastic Ma antigen 3 | 2.21 | 0.01457 |
| 1561048_at | *RARS2* | arginyl-tRNA synthetase 2, mitochondrial | 2.21 | 0.02268 |
| 1566115_at |  |  | 2.21 | 0.04495 |
| 212873_at | *HMHA1* | histocompatibility (minor) HA-1 | 2.2 | 6.7x10^-5^ |
| 213143_at | *C2orf72* | chromosome 2 open reading frame 72 | 2.2 | 0.00035 |
| 1562631_at | *TEX26-AS1* | TEX26 antisense RNA 1 | 2.2 | 0.00045 |
| 204748_at | *PTGS2* | prostaglandin-endoperoxide synthase 2 (prostaglandin G/H synthase and cyclooxygenase) | 2.2 | 0.00087 |
| 218223_s_at | *PLEKHO1* | pleckstrin homology domain containing, family O member 1 | 2.2 | 0.00198 |
| 206161_s_at | *SYT5* | synaptotagmin V | 2.2 | 0.00289 |
| 244491_at |  |  | 2.2 | 0.00299 |
| 234536_at | *SARDH* | sarcosine dehydrogenase | 2.2 | 0.00433 |
| 214851_at | *HNF4A* | hepatocyte nuclear factor 4, alpha | 2.2 | 0.00453 |
| 244060_at |  |  | 2.2 | 0.00619 |
| 203548_s_at | *LPL* | lipoprotein lipase | 2.2 | 0.0078 |
| 207306_at | *TCF15* | transcription factor 15 (basic helix-loop-helix) | 2.2 | 0.00972 |
| 213749_at | *MASP1* | mannan-binding lectin serine peptidase 1 (C4/C2 activating component of Ra-reactive factor) | 2.2 | 0.01061 |
| 240464_at |  |  | 2.2 | 0.01106 |
| 211486_s_at | *KCNQ2* | potassium voltage-gated channel, KQT-like subfamily, member 2 | 2.2 | 0.01546 |
| 206741_at | *SSUH2* | ssu-2 homolog (C. elegans) | 2.2 | 0.01977 |
| 1555985_at | *C17orf64* | chromosome 17 open reading frame 64 | 2.2 | 0.02333 |
| 212689_s_at | *KDM3A* | lysine (K)-specific demethylase 3A | 2.19 | 2.5x10^-5^ |
| 236571_at |  |  | 2.19 | 0.00011 |
| 230284_at | *MYOM3* | myomesin 3 | 2.19 | 0.00028 |
| 1560841_at |  |  | 2.19 | 0.00087 |
| 211692_s_at | *BBC3* | BCL2 binding component 3; microRNA 3190; microRNA 3191 | 2.19 | 0.00134 |
| 201271_s_at | *RALY* | RNA binding protein, autoantigenic (hnRNP-associated with lethal yellow homolog (mouse)) | 2.19 | 0.00158 |
| 202671_s_at | *PDXK* | pyridoxal (pyridoxine, vitamin B6) kinase | 2.19 | 0.00183 |
| 236118_at |  |  | 2.19 | 0.00259 |
| 244656_at | *RASL10B* | RAS-like, family 10, member B | 2.19 | 0.00327 |
| 231670_at |  |  | 2.19 | 0.00384 |
| 1556405_s_at |  |  | 2.19 | 0.00467 |
| 220765_s_at | *LIMS2* | LIM and senescent cell antigen-like domains 2 | 2.19 | 0.00659 |
| 244686_at | *TCOF1* | Treacher Collins-Franceschetti syndrome 1 | 2.19 | 0.00814 |
| 222020_s_at |  |  | 2.19 | 0.00988 |
| 237759_at | *CD48* | CD48 molecule | 2.19 | 0.01075 |
| 229631_at | *DNHD1* | dynein heavy chain domain 1 | 2.19 | 0.01133 |
| 231729_s_at | *CAPS* | calcyphosine | 2.19 | 0.01432 |
| 212283_at | *AGRN* | agrin | 2.19 | 0.01554 |
| 207641_at | *TNFRSF13B* | tumor necrosis factor receptor superfamily, member 13B | 2.19 | 0.02105 |
| 208797_s_at | *GOLGA8A* | golgin A8 family, member A | 2.19 | 0.02549 |
| 211660_at | *POU2F2* | POU class 2 homeobox 2 | 2.19 | 0.02782 |
| 215198_s_at | *CALD1* | caldesmon 1 | 2.19 | 0.02879 |
| 1553813_s_at | *TLE6* | transducin-like enhancer of split 6 (E(sp1) homolog, Drosophila) | 2.19 | 0.04517 |
| 200904_at | *HLA-E* | major histocompatibility complex, class I, E | 2.18 | 0.00017 |
| 214301_s_at | *DPYSL4* | dihydropyrimidinase-like 4 | 2.18 | 0.00023 |
| 215616_s_at | *KDM4B* | lysine (K)-specific demethylase 4B | 2.18 | 0.00038 |
| 229161_at |  |  | 2.18 | 0.00089 |
| 221713_s_at | *MAP6D1* | MAP6 domain containing 1 | 2.18 | 0.00185 |
| 231435_at | *C7orf34* | chromosome 7 open reading frame 34 | 2.18 | 0.00193 |
| 234958_at |  |  | 2.18 | 0.00213 |
| 239996_x_at | *ATP2A2* | ATPase, Ca++ transporting, cardiac muscle, slow twitch 2 | 2.18 | 0.00269 |
| 239581_at | *ARL10* | ADP-ribosylation factor-like 10 | 2.18 | 0.00289 |
| 237013_at |  |  | 2.18 | 0.00396 |
| 210507_s_at | *AVIL* | advillin | 2.18 | 0.00551 |
| 211181_x_at |  |  | 2.18 | 0.00591 |
| 210921_at |  |  | 2.18 | 0.0067 |
| 228350_at | *UNC13D* | unc-13 homolog D (C. elegans) | 2.18 | 0.00691 |
| 209347_s_at | *MAF* | v-maf musculoaponeurotic fibrosarcoma oncogene homolog (avian) | 2.18 | 0.00693 |
| 238541_at | *C21orf58* | chromosome 21 open reading frame 58 | 2.18 | 0.00775 |
| 233733_at |  |  | 2.18 | 0.00788 |
| 216388_s_at | *LTB4R* | leukotriene B4 receptor | 2.18 | 0.00843 |
| 211559_s_at | *CCNG2* | cyclin G2 | 2.18 | 0.01102 |
| 222164_at | *FGFR1* | fibroblast growth factor receptor 1 | 2.18 | 0.0139 |
| 230184_at |  |  | 2.18 | 0.01392 |
| 234250_at |  |  | 2.18 | 0.02071 |
| 1563897_at |  |  | 2.18 | 0.02337 |
| 1562655_at |  |  | 2.18 | 0.03558 |
| 208321_s_at | *CABP1* | calcium binding protein 1 | 2.17 | 1.5x10^-5^ |
| 223543_at | *PDZD4* | PDZ domain containing 4 | 2.17 | 0.0001 |
| 226869_at | *MEGF6* | multiple EGF-like-domains 6 | 2.17 | 0.00013 |
| 243769_at |  |  | 2.17 | 0.00043 |
| 225990_at | *BOC* | BOC cell adhesion associated, oncogene regulated | 2.17 | 0.00076 |
| 241346_at | *ARHGAP30* | Rho GTPase activating protein 30 | 2.17 | 0.00096 |
| 241327_at |  |  | 2.17 | 0.001 |
| 233756_at |  |  | 2.17 | 0.00176 |
| 206431_x_at | *TBC1D9B* | TBC1 domain family, member 9B (with GRAM domain) | 2.17 | 0.00224 |
| 235100_at |  |  | 2.17 | 0.00268 |
| 236744_at | *PHPT1* | phosphohistidine phosphatase 1 | 2.17 | 0.00349 |
| 230472_at | *IRX1* | iroquois homeobox 1 | 2.17 | 0.00465 |
| 235568_at | *C19orf59* | chromosome 19 open reading frame 59 | 2.17 | 0.00747 |
| 216271_x_at | *SYDE1* | synapse defective 1, Rho GTPase, homolog 1 (C. elegans) | 2.17 | 0.00797 |
| 239341_at |  |  | 2.17 | 0.0082 |
| 204802_at | *RRAD* | Ras-related associated with diabetes | 2.17 | 0.00943 |
| 235894_at |  |  | 2.17 | 0.01938 |
| 1558476_at | *BEND5* | BEN domain containing 5 | 2.16 | 0.00032 |
| 215844_at | *TNPO2* | transportin 2 | 2.16 | 0.001 |
| 205575_at | *C1QL1* | complement component 1, q subcomponent-like 1 | 2.16 | 0.00114 |
| 222831_at | *SAP30L* | SAP30-like | 2.16 | 0.00138 |
| 204298_s_at | *LOX* | lysyl oxidase | 2.16 | 0.00153 |
| 217407_x_at | *PPIL2* | peptidylprolyl isomerase (cyclophilin)-like 2 | 2.16 | 0.00222 |
| 204684_at | *NPTX1* | neuronal pentraxin I | 2.16 | 0.00261 |
| 236800_at | *UCMA* | upper zone of growth plate and cartilage matrix associated | 2.16 | 0.00278 |
| 220597_s_at | *ARL6IP4* | ADP-ribosylation-like factor 6 interacting protein 4 | 2.16 | 0.00279 |
| 206612_at | *CACNG1* | calcium channel, voltage-dependent, gamma subunit 1 | 2.16 | 0.00656 |
| 209495_at | *CEP250* | centrosomal protein 250kDa | 2.16 | 0.00709 |
| 215797_at | *TRAV8-3* | T cell receptor alpha variable 8-3 | 2.16 | 0.00721 |
| 1569745_at | *OSER1-AS1* | OSER1 antisense RNA 1 (head to head) | 2.16 | 0.00854 |
| 236732_at |  |  | 2.16 | 0.01089 |
| 202387_at | *BAG1* | BCL2-associated athanogene | 2.16 | 0.01462 |
| 1558532_at | *TPM1* | tropomyosin 1 (alpha) | 2.16 | 0.0151 |
| 234855_at | *ORF1* | uncharacterized protein, clone pT-Adv JuaX22 | 2.16 | 0.02026 |
| 1556161_a_at |  |  | 2.16 | 0.0218 |
| 204668_at | *RNF24* | ring finger protein 24 | 2.16 | 0.02214 |
| 239657_x_at | *FOXO6* | forkhead box O6; NULL | 2.16 | 0.02982 |
| 220708_at |  |  | 2.16 | 0.04763 |
| 216891_at |  |  | 2.15 | 0.00048 |
| 215869_at |  |  | 2.15 | 0.00105 |
| 205846_at | *PTPRB* | protein tyrosine phosphatase, receptor type, B | 2.15 | 0.00139 |
| 223129_x_at | *MYLIP* | myosin regulatory light chain interacting protein | 2.15 | 0.00164 |
| 210215_at | *TFR2* | transferrin receptor 2 | 2.15 | 0.00167 |
| 229190_at |  |  | 2.15 | 0.00189 |
| 212236_x_at | *JUP* | junction plakoglobin; keratin 17 | 2.15 | 0.00257 |
| 206410_at | *NR0B2* | nuclear receptor subfamily 0, group B, member 2 | 2.15 | 0.00292 |
| 238531_x_at |  |  | 2.15 | 0.00365 |
| 220677_s_at | *ADAMTS8* | ADAM metallopeptidase with thrombospondin type 1 motif, 8 | 2.15 | 0.00538 |
| 239419_at | *PTPRA* | protein tyrosine phosphatase, receptor type, A | 2.15 | 0.00806 |
| 202523_s_at | *SPOCK2* | sparc/osteonectin, cwcv and kazal-like domains proteoglycan (testican) 2 | 2.15 | 0.00842 |
| 220778_x_at | *SEMA6B* | sema domain, transmembrane domain (TM), and cytoplasmic domain, (semaphorin) 6B | 2.15 | 0.01218 |
| 211322_s_at | *SARDH* | sarcosine dehydrogenase | 2.15 | 0.01232 |
| 207751_at | *PRO2949* | uncharacterized protein PRO2949 | 2.15 | 0.01606 |
| 202934_at | *HK2* | hexokinase 2 | 2.14 | 0.00014 |
| 213664_at | *SLC1A1* | solute carrier family 1 (neuronal/epithelial high affinity glutamate transporter, system Xag), member 1 | 2.14 | 0.00019 |
| 227084_at | *DTNA* | dystrobrevin, alpha | 2.14 | 0.00024 |
| 1557080_s_at | *ITGBL1* | integrin, beta-like 1 (with EGF-like repeat domains) | 2.14 | 0.00026 |
| 204954_s_at | *DYRK1B* | dual-specificity tyrosine-(Y)-phosphorylation regulated kinase 1B | 2.14 | 0.00062 |
| 219862_s_at | *NARF* | nuclear prelamin A recognition factor | 2.14 | 0.0009 |
| 214592_s_at | *SNAPC5* | small nuclear RNA activating complex, polypeptide 5, 19kDa | 2.14 | 0.00176 |
| 205722_s_at | *GFRA2* | GDNF family receptor alpha 2 | 2.14 | 0.00197 |
| 235930_at | *KCNMB4* | potassium large conductance calcium-activated channel, subfamily M, beta member 4 | 2.14 | 0.00257 |
| 230374_at |  |  | 2.14 | 0.00326 |
| 216928_at | *TAL1* | T-cell acute lymphocytic leukemia 1 | 2.14 | 0.00363 |
| 241670_x_at |  |  | 2.14 | 0.00388 |
| 237371_at |  |  | 2.14 | 0.00397 |
| 202803_s_at | *ITGB2* | integrin, beta 2 (complement component 3 receptor 3 and 4 subunit) | 2.14 | 0.00435 |
| 1562914_a_at | *LINC00905* | long intergenic non-protein coding RNA 905 | 2.14 | 0.00689 |
| 244217_at |  |  | 2.14 | 0.00693 |
| 1565000_a_at | *TCP11L2* | t-complex 11, testis-specific-like 2 | 2.14 | 0.00764 |
| 209403_at |  |  | 2.14 | 0.00764 |
| 240229_at |  |  | 2.14 | 0.00983 |
| 231079_at |  |  | 2.14 | 0.01461 |
| 1557690_x_at |  |  | 2.14 | 0.02048 |
| 202343_x_at | *COX5B* | cytochrome c oxidase subunit Vb | 2.14 | 0.0308 |
| 219742_at | *PRR7* | proline rich 7 (synaptic) | 2.14 | 0.03403 |
| 1570645_at |  |  | 2.14 | 0.03692 |
| 206619_at | *DKK4* | dickkopf WNT signaling pathway inhibitor 4 | 2.14 | 0.03956 |
| 204845_s_at | *ENPEP* | glutamyl aminopeptidase (aminopeptidase A) | 2.13 | 1x10^-6^ |
| 230704_s_at | *ITGB4* | integrin, beta 4 | 2.13 | 0.00054 |
| 212054_x_at | *TBC1D9B* | TBC1 domain family, member 9B (with GRAM domain) | 2.13 | 0.00085 |
| 238423_at | *SYTL3* | synaptotagmin-like 3 | 2.13 | 0.00088 |
| 230221_at | *ABHD16A* | abhydrolase domain containing 16A | 2.13 | 0.00101 |
| 238445_x_at | *MGAT5B* | mannosyl (alpha-1,6-)-glycoprotein beta-1,6-N-acetyl-glucosaminyltransferase, isozyme B | 2.13 | 0.00123 |
| 221792_at | *RAB6B* | RAB6B, member RAS oncogene family | 2.13 | 0.00206 |
| 221866_at | *TFEB* | transcription factor EB | 2.13 | 0.00263 |
| 241890_at |  |  | 2.13 | 0.00282 |
| 1563541_at |  |  | 2.13 | 0.00324 |
| 1569006_at |  |  | 2.13 | 0.00361 |
| 234026_at |  |  | 2.13 | 0.00382 |
| 232337_at | *B3GNT7* | UDP-GlcNAc:betaGal beta-1,3-N-acetylglucosaminyltransferase 7 | 2.13 | 0.00398 |
| 214111_at | *OPCML* | opioid binding protein/cell adhesion molecule-like | 2.13 | 0.00427 |
| 203891_s_at | *DAPK3* | death-associated protein kinase 3; microRNA 637 | 2.13 | 0.00444 |
| 244104_at | *MGAT3* | mannosyl (beta-1,4-)-glycoprotein beta-1,4-N-acetylglucosaminyltransferase | 2.13 | 0.00459 |
| 231884_at | *CNTROB* | centrobin, centrosomal BRCA2 interacting protein | 2.13 | 0.00464 |
| 240538_at |  |  | 2.13 | 0.00552 |
| 206922_at | *VCY* | variable charge, Y-linked; variable charge, Y-linked 1B | 2.13 | 0.00561 |
| 216289_at | *GPR144* | G protein-coupled receptor 144 | 2.13 | 0.0062 |
| 1566916_at | *HPYR1* | Helicobacter pylori responsive 1 (non-protein coding) | 2.13 | 0.00706 |
| 220002_at | *KIF26B* | kinesin family member 26B | 2.13 | 0.01211 |
| 202328_s_at |  |  | 2.13 | 0.014 |
| 206215_at | *OPCML* | opioid binding protein/cell adhesion molecule-like | 2.13 | 0.01502 |
| 236598_at |  |  | 2.13 | 0.02478 |
| 211591_s_at | *PDE4A* | phosphodiesterase 4A, cAMP-specific | 2.13 | 0.03348 |
| 221793_at |  |  | 2.13 | 0.03386 |
| 231488_at | *OTP* | orthopedia homeobox | 2.12 | 0.00021 |
| 220435_at | *SLC30A10* | solute carrier family 30, member 10 | 2.12 | 0.00059 |
| 209466_x_at | *PTN* | pleiotrophin | 2.12 | 0.00088 |
| 202402_s_at | *CARS* | cysteinyl-tRNA synthetase | 2.12 | 0.00093 |
| 200832_s_at | *SCD* | stearoyl-CoA desaturase (delta-9-desaturase) | 2.12 | 0.00169 |
| 243497_at |  |  | 2.12 | 0.00243 |
| 221860_at | *HNRNPL* | heterogeneous nuclear ribonucleoprotein L | 2.12 | 0.00433 |
| 242119_at |  |  | 2.12 | 0.00444 |
| 220080_at | *FBXL8* | F-box and leucine-rich repeat protein 8 | 2.12 | 0.00687 |
| 205050_s_at | *MAPK8IP2* | mitogen-activated protein kinase 8 interacting protein 2 | 2.12 | 0.00854 |
| 210343_s_at | *SLC22A6* | solute carrier family 22 (organic anion transporter), member 6 | 2.12 | 0.00897 |
| 212938_at | *COL6A1* | collagen, type VI, alpha 1 | 2.12 | 0.01179 |
| 1558378_a_at | *AHNAK2* | AHNAK nucleoprotein 2 | 2.12 | 0.01784 |
| 228725_x_at | *PRMT2* | protein arginine methyltransferase 2 | 2.12 | 0.02716 |
| 214115_at | *VAMP5* | vesicle-associated membrane protein 5 | 2.12 | 0.02887 |
| 236993_at |  |  | 2.12 | 0.03038 |
| 209266_s_at | *SLC39A8* | solute carrier family 39 (zinc transporter), member 8 | 2.11 | 0.00021 |
| 202500_at | *DNAJB2* | DnaJ (Hsp40) homolog, subfamily B, member 2 | 2.11 | 0.00053 |
| 206338_at | *ELAVL3* | ELAV (embryonic lethal, abnormal vision, Drosophila)-like 3 (Hu antigen C) | 2.11 | 0.00101 |
| 242187_s_at | *TNRC18* | trinucleotide repeat containing 18 | 2.11 | 0.00105 |
| 238345_at | *SLC38A10* | solute carrier family 38, member 10 | 2.11 | 0.00106 |
| 214630_at | *CYP11B2* | cytochrome P450, family 11, subfamily B, polypeptide 2 | 2.11 | 0.00132 |
| 219155_at | *PITPNC1* | phosphatidylinositol transfer protein, cytoplasmic 1 | 2.11 | 0.00132 |
| 216929_x_at | *ABO* | ABO blood group (transferase A, alpha 1-3-N-acetylgalactosaminyltransferase; transferase B, alpha 1-3-galactosyltransferase) | 2.11 | 0.00141 |
| 227394_at | *NCAM1* | neural cell adhesion molecule 1 | 2.11 | 0.00265 |
| 212776_s_at | *OBSL1* | obscurin-like 1 | 2.11 | 0.00349 |
| 235734_at | *ARFGAP2* | ADP-ribosylation factor GTPase activating protein 2 | 2.11 | 0.00377 |
| 1570635_at |  |  | 2.11 | 0.00393 |
| 1556192_x_at |  |  | 2.11 | 0.00409 |
| 209912_s_at | *AP5Z1* | adaptor-related protein complex 5, zeta 1 subunit; microRNA 4656 | 2.11 | 0.00417 |
| 217509_x_at | *GRIK5* | glutamate receptor, ionotropic, kainate 5 | 2.11 | 0.00474 |
| 225146_at | *FAM219A* | family with sequence similarity 219, member A | 2.11 | 0.00521 |
| 211512_s_at | *OGFR* | opioid growth factor receptor | 2.11 | 0.00557 |
| 223828_s_at | *LGALS12* | lectin, galactoside-binding, soluble, 12 | 2.11 | 0.00617 |
| 218624_s_at | *MGC2752* | CENPB DNA-binding domains containing 1 pseudogene | 2.11 | 0.00629 |
| 216041_x_at | *GRN* | granulin | 2.11 | 0.0072 |
| 212859_x_at | *MT1E* | metallothionein 1E | 2.11 | 0.00734 |
| 208432_s_at | *CACNA1E* | calcium channel, voltage-dependent, R type, alpha 1E subunit | 2.11 | 0.00745 |
| 215778_x_at |  |  | 2.11 | 0.00889 |
| 228928_x_at | *BANP* | BTG3 associated nuclear protein | 2.11 | 0.0097 |
| 235002_at | *NUDT16* | nudix (nucleoside diphosphate linked moiety X)-type motif 16 | 2.11 | 0.01051 |
| 214304_x_at |  |  | 2.11 | 0.01067 |
| 205813_s_at | *MAT1A* | methionine adenosyltransferase I, alpha | 2.11 | 0.01311 |
| 238326_at | *ODF3B* | outer dense fiber of sperm tails 3B | 2.11 | 0.01313 |
| 244884_at |  |  | 2.11 | 0.01386 |
| 227999_at | *PWWP2B* | PWWP domain containing 2B | 2.11 | 0.01945 |
| 243180_at |  |  | 2.11 | 0.0236 |
| 228010_at | *PPP2R2C* | protein phosphatase 2, regulatory subunit B, gamma | 2.11 | 0.02485 |
| 219483_s_at | *PORCN* | porcupine homolog (Drosophila) | 2.11 | 0.02905 |
| 216916_s_at | *DLGAP2* | discs, large (Drosophila) homolog-associated protein 2 | 2.11 | 0.04592 |
| 240919_at |  |  | 2.1 | 0.00015 |
| 214973_x_at | *IGHD* | immunoglobulin heavy constant delta | 2.1 | 0.00063 |
| 215256_x_at | *ARHGAP33* | Rho GTPase activating protein 33 | 2.1 | 0.00173 |
| 221990_at | *PAX8* | paired box 8 | 2.1 | 0.00176 |
| 240147_at | *C7orf50* | chromosome 7 open reading frame 50 | 2.1 | 0.00212 |
| 205374_at | *SLN* | sarcolipin | 2.1 | 0.00236 |
| 244421_at |  |  | 2.1 | 0.00264 |
| 200823_x_at | *RPL29* | ribosomal protein L29 | 2.1 | 0.00412 |
| 214461_at | *LBP* | lipopolysaccharide binding protein | 2.1 | 0.00548 |
| 202997_s_at | *LOXL2* | lysyl oxidase-like 2 | 2.1 | 0.00664 |
| 205744_at | *DOC2A* | double C2-like domains, alpha | 2.1 | 0.00715 |
| 224497_x_at | *HSD17B14* | hydroxysteroid (17-beta) dehydrogenase 14 | 2.1 | 0.00755 |
| 213870_at | *COL11A2* | collagen, type XI, alpha 2 | 2.1 | 0.00836 |
| 216317_x_at | *RHCE* | Rh blood group, CcEe antigens | 2.1 | 0.00877 |
| 232105_at | *BLACAT1* | bladder cancer associated transcript 1 (non-protein coding) | 2.1 | 0.0133 |
| 206219_s_at | *VAV1* | vav 1 guanine nucleotide exchange factor | 2.1 | 0.01381 |
| 203473_at | *SLCO2B1* | solute carrier organic anion transporter family, member 2B1 | 2.1 | 0.02643 |
| 213566_at | *RNASE6* | ribonuclease, RNase A family, k6 | 2.1 | 0.03327 |
| 211706_s_at | *CDK19* | cyclin-dependent kinase 19 | 2.1 | 0.03491 |
| 225136_at | *PLEKHA2* | pleckstrin homology domain containing, family A (phosphoinositide binding specific) member 2 | 2.09 | 3.7x10^-5^ |
| 236749_at | *MNT* | MNT, MAX dimerization protein | 2.09 | 0.00071 |
| 239886_at |  |  | 2.09 | 0.00098 |
| 233186_s_at | *BANP* | BTG3 associated nuclear protein | 2.09 | 0.00158 |
| 241650_x_at | *HMCN2* | hemicentin 2; hemicentin-2-like | 2.09 | 0.00161 |
| 202002_at | *ACAA2* | acetyl-CoA acyltransferase 2 | 2.09 | 0.00175 |
| 89476_r_at | *NPEPL1* | aminopeptidase-like 1 | 2.09 | 0.00202 |
| 230212_at | *SPRY1* | sprouty homolog 1, antagonist of FGF signaling (Drosophila) | 2.09 | 0.00232 |
| 222059_at | *ZNF335* | zinc finger protein 335 | 2.09 | 0.00268 |
| 212803_at | *NAB2* | NGFI-A binding protein 2 (EGR1 binding protein 2) | 2.09 | 0.00308 |
| 211024_s_at | *NKX2-1* | NK2 homeobox 1 | 2.09 | 0.00316 |
| 240651_at |  |  | 2.09 | 0.00323 |
| 1569443_s_at |  |  | 2.09 | 0.00327 |
| 211605_s_at | *RARA* | retinoic acid receptor, alpha | 2.09 | 0.00346 |
| 205817_at | *SIX1* | SIX homeobox 1 | 2.09 | 0.00359 |
| 238082_at |  |  | 2.09 | 0.00392 |
| 220757_s_at | *MIR4746* | microRNA 4746; UBX domain protein 6 | 2.09 | 0.00434 |
| 228068_at | *GOLGA7B* | golgin A7 family, member B | 2.09 | 0.00446 |
| 204136_at | *COL7A1* | collagen, type VII, alpha 1 | 2.09 | 0.00455 |
| 240424_s_at |  |  | 2.09 | 0.00505 |
| 204311_at | *ATP1B2* | ATPase, Na+/K+ transporting, beta 2 polypeptide | 2.09 | 0.00607 |
| 243002_at |  |  | 2.09 | 0.00727 |
| 233802_at |  |  | 2.09 | 0.00865 |
| 210736_x_at | *DTNA* | dystrobrevin, alpha | 2.09 | 0.01235 |
| 202410_x_at | *IGF2* | insulin-like growth factor 2 (somatomedin A); INS-IGF2 readthrough | 2.09 | 0.01476 |
| 203761_at | *SLA* | Src-like-adaptor | 2.09 | 0.01569 |
| 210364_at | *SCN2B* | sodium channel, voltage-gated, type II, beta subunit | 2.09 | 0.01602 |
| 238271_x_at |  |  | 2.09 | 0.01673 |
| 1560848_at |  |  | 2.09 | 0.02267 |
| 1556456_at |  |  | 2.09 | 0.02356 |
| 1558649_at |  |  | 2.09 | 0.02529 |
| 236776_at |  |  | 2.09 | 0.03718 |
| 214797_s_at | *CDK18* | cyclin-dependent kinase 18 | 2.08 | 4.4x10^-5^ |
| 229913_at | *C7orf61* | chromosome 7 open reading frame 61 | 2.08 | 0.00026 |
| 241944_x_at |  |  | 2.08 | 0.00073 |
| 201752_s_at | *ADD3* | adducin 3 (gamma) | 2.08 | 0.00074 |
| 227044_at |  |  | 2.08 | 0.00111 |
| 234404_at | *CAND2* | cullin-associated and neddylation-dissociated 2 (putative) | 2.08 | 0.00117 |
| 202733_at | *P4HA2* | prolyl 4-hydroxylase, alpha polypeptide II | 2.08 | 0.00127 |
| 218840_s_at | *NADSYN1* | NAD synthetase 1 | 2.08 | 0.0018 |
| 221778_at | *JHDM1D* | jumonji C domain containing histone demethylase 1 homolog D (S. cerevisiae) | 2.08 | 0.00239 |
| 209565_at | *RNF113A* | ring finger protein 113A | 2.08 | 0.00277 |
| 230615_at | *DUOXA2* | dual oxidase maturation factor 2 | 2.08 | 0.00296 |
| 242039_at | *ARAP1* | ArfGAP with RhoGAP domain, ankyrin repeat and PH domain 1 | 2.08 | 0.00305 |
| 210350_x_at | *ING1* | inhibitor of growth family, member 1 | 2.08 | 0.00331 |
| 230423_at |  |  | 2.08 | 0.00351 |
| 204801_s_at | *DHRS12* | dehydrogenase/reductase (SDR family) member 12 | 2.08 | 0.00401 |
| 1554748_at | *CLCNKB* | chloride channel, voltage-sensitive Kb | 2.08 | 0.00429 |
| 237534_at |  |  | 2.08 | 0.0044 |
| 229612_at |  |  | 2.08 | 0.00493 |
| 205319_at | *PSCA* | prostate stem cell antigen | 2.08 | 0.00499 |
| 236091_at | *HMGB2* | high mobility group box 2 | 2.08 | 0.0053 |
| 210929_s_at | *AHSG* | alpha-2-HS-glycoprotein | 2.08 | 0.00589 |
| 208581_x_at | *MT1X* | metallothionein 1X | 2.08 | 0.00605 |
| 1556281_at |  |  | 2.08 | 0.00612 |
| 218216_x_at | *ARL6IP4* | ADP-ribosylation-like factor 6 interacting protein 4 | 2.08 | 0.00648 |
| 1566323_at | *MAF* | v-maf musculoaponeurotic fibrosarcoma oncogene homolog (avian) | 2.08 | 0.00778 |
| 230419_at | *FLJ37644* | uncharacterized LOC400618 | 2.08 | 0.00805 |
| 231692_at | *PIGG* | phosphatidylinositol glycan anchor biosynthesis, class G | 2.08 | 0.00918 |
| 240067_at |  |  | 2.08 | 0.00983 |
| 235976_at | *SLITRK6* | SLIT and NTRK-like family, member 6 | 2.08 | 0.01003 |
| 234505_at |  |  | 2.08 | 0.01101 |
| 240051_at | *TPD52L3* | tumor protein D52-like 3 | 2.08 | 0.01252 |
| 234919_s_at | *SNTG1* | syntrophin, gamma 1 | 2.08 | 0.01485 |
| 205485_at | *RYR1* | ryanodine receptor 1 (skeletal) | 2.08 | 0.01856 |
| 208105_at | *GIPR* | gastric inhibitory polypeptide receptor | 2.08 | 0.01965 |
| 225072_at | *ZCCHC3* | zinc finger, CCHC domain containing 3 | 2.08 | 0.02202 |
| 233060_at | *ZMIZ1* | zinc finger, MIZ-type containing 1 | 2.08 | 0.03768 |
| 220686_s_at | *PIWIL2* | piwi-like RNA-mediated gene silencing 2 | 2.08 | 0.04469 |
| 230675_at |  |  | 2.07 | 0.00007 |
| 238741_at | *FAM83A* | family with sequence similarity 83, member A | 2.07 | 0.00009 |
| 226792_s_at | *KIFC2* | kinesin family member C2 | 2.07 | 9.9x10^-5^ |
| 202772_at | *HMGCL* | 3-hydroxymethyl-3-methylglutaryl-CoA lyase | 2.07 | 0.00022 |
| 218898_at | *FAM57A* | family with sequence similarity 57, member A | 2.07 | 0.00035 |
| 236529_at | *SRCRB4D* | scavenger receptor cysteine rich domain containing, group B (4 domains) | 2.07 | 0.0009 |
| 232133_at | *ADAMTS10* | ADAM metallopeptidase with thrombospondin type 1 motif, 10 | 2.07 | 0.00114 |
| 228384_s_at | *PYROXD2* | pyridine nucleotide-disulphide oxidoreductase domain 2 | 2.07 | 0.00115 |
| 223326_s_at | *LOC80154* | golgin A2 pseudogene | 2.07 | 0.00142 |
| 228296_at | *YPEL1* | yippee-like 1 (Drosophila) | 2.07 | 0.00148 |
| 208584_at | *SNCG* | synuclein, gamma (breast cancer-specific protein 1) | 2.07 | 0.00162 |
| 234781_at |  |  | 2.07 | 0.0022 |
| 233525_s_at | *LINC00475* | long intergenic non-protein coding RNA 475 | 2.07 | 0.00233 |
| 206278_at | *PTAFR* | platelet-activating factor receptor | 2.07 | 0.00252 |
| 240828_at | *JARID2-AS1* | JARID2 antisense RNA 1 | 2.07 | 0.00255 |
| 205213_at | *ACAP1* | ArfGAP with coiled-coil, ankyrin repeat and PH domains 1 | 2.07 | 0.0027 |
| 205882_x_at | *ADD3* | adducin 3 (gamma) | 2.07 | 0.00273 |
| 233317_at | *CD9* | CD9 molecule | 2.07 | 0.00356 |
| 222888_at | *CCNJ* | cyclin J | 2.07 | 0.00435 |
| 222042_x_at | *MEX3D* | mex-3 homolog D (C. elegans) | 2.07 | 0.00452 |
| 216699_s_at | *KLK1* | kallikrein 1 | 2.07 | 0.00457 |
| 213767_at | *KSR1* | kinase suppressor of ras 1 | 2.07 | 0.00567 |
| 223631_s_at | *C19orf33* | chromosome 19 open reading frame 33 | 2.07 | 0.00638 |
| 242081_at | *ACAP1* | ArfGAP with coiled-coil, ankyrin repeat and PH domains 1 | 2.07 | 0.00655 |
| 241357_at | *MAPK15* | mitogen-activated protein kinase 15 | 2.07 | 0.00668 |
| 1554380_at | *NEK11* | NIMA-related kinase 11 | 2.07 | 0.00745 |
| 221680_s_at | *ETV7* | ets variant 7 | 2.07 | 0.0088 |
| 204850_s_at | *DCX* | doublecortin | 2.07 | 0.01095 |
| 220829_s_at | *B3GALT1* | UDP-Gal:betaGlcNAc beta 1,3-galactosyltransferase, polypeptide 1 | 2.07 | 0.01541 |
| 219438_at | *NKAIN1* | Na+/K+ transporting ATPase interacting 1 | 2.07 | 0.02235 |
| 224890_s_at | *LAMTOR4* | late endosomal/lysosomal adaptor, MAPK and MTOR activator 4 | 2.07 | 0.02737 |
| 229429_x_at | *LINC00623* | long intergenic non-protein coding RNA 623; uncharacterized LOC728875 | 2.07 | 0.03207 |
| 207299_s_at | *GRM1* | glutamate receptor, metabotropic 1 | 2.07 | 0.04018 |
| 217004_s_at | *MCF2* | MCF.2 cell line derived transforming sequence | 2.06 | 0.00043 |
| 208455_at | *PVRL1* | poliovirus receptor-related 1 (herpesvirus entry mediator C) | 2.06 | 0.00105 |
| 240349_at | *PRKAA2* | protein kinase, AMP-activated, alpha 2 catalytic subunit | 2.06 | 0.00106 |
| 231794_at | *CTLA4* | cytotoxic T-lymphocyte-associated protein 4 | 2.06 | 0.00131 |
| 216894_x_at | *CDKN1C* | cyclin-dependent kinase inhibitor 1C (p57, Kip2) | 2.06 | 0.00138 |
| 220319_s_at | *MYLIP* | myosin regulatory light chain interacting protein | 2.06 | 0.00144 |
| 1255_g_at | *GUCA1A* | guanylate cyclase activator 1A (retina) | 2.06 | 0.00146 |
| 233723_at |  |  | 2.06 | 0.00187 |
| 1558821_s_at | *AC083843.1* | NULL | 2.06 | 0.00189 |
| 203859_s_at | *PALM* | paralemmin | 2.06 | 0.00228 |
| 208215_x_at | *DRD4* | dopamine receptor D4 | 2.06 | 0.00342 |
| 228832_at | *FLJ20021* | uncharacterized LOC90024 | 2.06 | 0.00347 |
| 202498_s_at | *SLC2A3* | solute carrier family 2 (facilitated glucose transporter), member 3 | 2.06 | 0.00348 |
| 228114_x_at | *C16orf13* | chromosome 16 open reading frame 13 | 2.06 | 0.00376 |
| 203558_at | *CUL7* | cullin 7 | 2.06 | 0.0049 |
| 236959_s_at |  |  | 2.06 | 0.00515 |
| 1561453_at |  |  | 2.06 | 0.00536 |
| 221539_at | *EIF4EBP1* | eukaryotic translation initiation factor 4E binding protein 1 | 2.06 | 0.00735 |
| 1562722_at | *PRR20A* | proline rich 20A; proline rich 20B; proline rich 20C; proline rich 20D; proline rich 20E | 2.06 | 0.00781 |
| 240048_at | *STRC* | stereocilin | 2.06 | 0.00801 |
| 216320_x_at | *MST1* | macrophage stimulating 1 (hepatocyte growth factor-like) | 2.06 | 0.0082 |
| 215700_x_at | *CPNE6* | copine VI (neuronal) | 2.06 | 0.0108 |
| 244882_at | *TNRC18* | trinucleotide repeat containing 18 | 2.06 | 0.01675 |
| 236891_at |  |  | 2.06 | 0.01714 |
| 1556320_at | *STOML1* | stomatin (EPB72)-like 1 | 2.06 | 0.02222 |
| 228132_at | *ABLIM2* | actin binding LIM protein family, member 2 | 2.06 | 0.02699 |
| 219756_s_at | *POF1B* | premature ovarian failure, 1B | 2.06 | 0.0297 |
| 210155_at | *MYOC* | myocilin, trabecular meshwork inducible glucocorticoid response | 2.06 | 0.03031 |
| 225983_s_at | *VWA1* | von Willebrand factor A domain containing 1 | 2.06 | 0.03197 |
| 1557422_at |  |  | 2.06 | 0.03539 |
| 1562311_at |  |  | 2.06 | 0.03647 |
| 1560456_at | *PLIN5* | perilipin 5 | 2.06 | 0.03971 |
| 230616_at | *LAMB2P1* | laminin, beta 2 pseudogene 1 | 2.06 | 0.04822 |
| 211065_x_at | *PFKL* | phosphofructokinase, liver | 2.05 | 0.00024 |
| 220430_at | *FAM110D* | family with sequence similarity 110, member D | 2.05 | 0.00061 |
| 217040_x_at | *SOX15* | SRY (sex determining region Y)-box 15 | 2.05 | 0.00082 |
| 237046_x_at | *IL34* | interleukin 34 | 2.05 | 0.00117 |
| 220989_s_at | *AMN* | amnion associated transmembrane protein | 2.05 | 0.00137 |
| 228048_at | *ZNF503-AS2* | ZNF503 antisense RNA 2 | 2.05 | 0.00154 |
| 238629_x_at |  |  | 2.05 | 0.00173 |
| 232025_at | *SYT7* | synaptotagmin VII | 2.05 | 0.00238 |
| 208064_s_at | *ST8SIA3* | ST8 alpha-N-acetyl-neuraminide alpha-2,8-sialyltransferase 3 | 2.05 | 0.00248 |
| 235624_at | *HDLBP* | high density lipoprotein binding protein | 2.05 | 0.00265 |
| 226424_at | *CAPS* | calcyphosine | 2.05 | 0.00281 |
| 237000_at |  |  | 2.05 | 0.00332 |
| 236051_at |  |  | 2.05 | 0.0034 |
| 230707_at | *SORL1* | sortilin-related receptor, L(DLR class) A repeats containing | 2.05 | 0.00366 |
| 242658_at |  |  | 2.05 | 0.00367 |
| 240531_at |  |  | 2.05 | 0.00379 |
| 221968_s_at | *ZNF771* | zinc finger protein 771 | 2.05 | 0.00411 |
| 203592_s_at | *FSTL3* | follistatin-like 3 (secreted glycoprotein) | 2.05 | 0.00464 |
| 216206_x_at | *MAP2K7* | mitogen-activated protein kinase kinase 7 | 2.05 | 0.00556 |
| 229769_at | *TMEM242* | transmembrane protein 242 | 2.05 | 0.0061 |
| 208592_s_at | *CD1E* | CD1e molecule | 2.05 | 0.00621 |
| 1555049_at | *TSPEAR* | thrombospondin-type laminin G domain and EAR repeats | 2.05 | 0.00655 |
| 229761_at |  |  | 2.05 | 0.00664 |
| 1556916_a_at |  |  | 2.05 | 0.00728 |
| 214619_at | *CRHR1* | corticotropin releasing hormone receptor 1 | 2.05 | 0.00824 |
| 213711_at | *KRT81* | keratin 81 | 2.05 | 0.00882 |
| 208888_s_at | *NCOR2* | nuclear receptor corepressor 2 | 2.05 | 0.00916 |
| 202390_s_at | *HTT* | huntingtin | 2.05 | 0.01016 |
| 243905_at |  |  | 2.05 | 0.01243 |
| 229160_at | *MUM1L1* | melanoma associated antigen (mutated) 1-like 1 | 2.05 | 0.01306 |
| 1554285_at | *HAVCR2* | hepatitis A virus cellular receptor 2 | 2.05 | 0.01497 |
| 221900_at | *COL8A2* | collagen, type VIII, alpha 2 | 2.05 | 0.01602 |
| 229608_at | *FAM212B* | family with sequence similarity 212, member B | 2.05 | 0.02331 |
| 1564385_at |  |  | 2.05 | 0.03492 |
| 209870_s_at | *APBA2* | amyloid beta (A4) precursor protein-binding, family A, member 2 | 2.05 | 0.04841 |
| 234701_at | *ANKRD11* | ankyrin repeat domain 11 | 2.04 | 0.00015 |
| 241508_at |  |  | 2.04 | 0.00029 |
| 211632_at | *IGHD* | immunoglobulin heavy constant delta; immunoglobulin heavy constant gamma 1 (G1m marker); immunoglobulin heavy constant mu | 2.04 | 0.00051 |
| 204284_at | *PPP1R3C* | protein phosphatase 1, regulatory subunit 3C | 2.04 | 0.00052 |
| 211808_s_at | *CREBBP* | CREB binding protein | 2.04 | 0.00064 |
| 239920_at | *UBTF* | upstream binding transcription factor, RNA polymerase I | 2.04 | 0.00115 |
| 203778_at | *MANBA* | mannosidase, beta A, lysosomal | 2.04 | 0.0015 |
| 219523_s_at | *TENM3* | teneurin transmembrane protein 3 | 2.04 | 0.00177 |
| 213182_x_at | *CDKN1C* | cyclin-dependent kinase inhibitor 1C (p57, Kip2) | 2.04 | 0.00184 |
| 209890_at | *TSPAN5* | tetraspanin 5 | 2.04 | 0.00242 |
| 236673_at | *TIFAB* | TRAF-interacting protein with forkhead-associated domain, family member B | 2.04 | 0.00268 |
| 232470_at | *SIK1* | salt-inducible kinase 1 | 2.04 | 0.00286 |
| 1567058_at | *OR8G2* | olfactory receptor, family 8, subfamily G, member 2 | 2.04 | 0.00323 |
| 226667_x_at | *EPN1* | epsin 1 | 2.04 | 0.00338 |
| 243774_at | *MUC20* | mucin 20, cell surface associated | 2.04 | 0.00365 |
| 36084_at | *CUL7* | cullin 7 | 2.04 | 0.00385 |
| 230536_at | *PBX4* | pre-B-cell leukemia homeobox 4 | 2.04 | 0.004 |
| 242341_x_at | *GLYCTK* | glycerate kinase | 2.04 | 0.00522 |
| 211474_s_at | *SERPINB6* | serpin peptidase inhibitor, clade B (ovalbumin), member 6 | 2.04 | 0.00576 |
| 224268_x_at | *ZAN* | zonadhesin | 2.04 | 0.006 |
| 221091_at | *INSL5* | insulin-like 5 | 2.04 | 0.00667 |
| 211639_x_at | *IGH* | immunoglobulin heavy locus; immunoglobulin heavy constant alpha 1; immunoglobulin heavy constant alpha 2 (A2m marker); immunoglobulin heavy constant delta; immunoglobulin heavy constant gamma 1 (G1m marker); immunoglobulin heavy constant gamma 3 (G3m marker); immunoglobulin heavy constant gamma 4 (G4m marker); immunoglobulin heavy constant mu; immunoglobulin heavy variable 4-31 | 2.04 | 0.00723 |
| 238943_at | *FIBCD1* | fibrinogen C domain containing 1 | 2.04 | 0.00727 |
| 234031_at | *C20orf112* | chromosome 20 open reading frame 112 | 2.04 | 0.00857 |
| 1559641_at |  |  | 2.04 | 0.00906 |
| 219345_at | *BOLA1* | bolA homolog 1 (E. coli) | 2.04 | 0.01011 |
| 1565846_at |  |  | 2.04 | 0.0106 |
| 231050_at | *HRASLS5* | HRAS-like suppressor family, member 5 | 2.04 | 0.01413 |
| 236285_at | *KLHDC7B* | kelch domain containing 7B | 2.04 | 0.01427 |
| 219733_s_at | *SLC27A5* | solute carrier family 27 (fatty acid transporter), member 5 | 2.04 | 0.01593 |
| 206309_at | *LECT1* | leukocyte cell derived chemotaxin 1 | 2.04 | 0.01869 |
| 242860_at |  |  | 2.04 | 0.01891 |
| 222123_s_at | *HIF3A* | hypoxia inducible factor 3, alpha subunit | 2.04 | 0.02151 |
| 239263_at |  |  | 2.04 | 0.02654 |
| 210226_at | *NR4A1* | nuclear receptor subfamily 4, group A, member 1 | 2.04 | 0.04159 |
| 206486_at | *LAG3* | lymphocyte-activation gene 3 | 2.03 | 0.00011 |
| 203516_at | *SNTA1* | syntrophin, alpha 1 | 2.03 | 0.00039 |
| 217585_at | *NEBL* | nebulette | 2.03 | 0.00047 |
| 41386_i_at | *KDM6B* | lysine (K)-specific demethylase 6B | 2.03 | 0.00054 |
| 235845_at | *SP5* | Sp5 transcription factor | 2.03 | 0.00064 |
| 227263_at | *C8orf58* | chromosome 8 open reading frame 58 | 2.03 | 0.00082 |
| 227894_at | *WDR90* | WD repeat domain 90 | 2.03 | 0.00091 |
| 234881_at |  |  | 2.03 | 0.00098 |
| 213471_at | *NPHP4* | nephronophthisis 4 | 2.03 | 0.00178 |
| 228327_x_at | *MEIS3* | Meis homeobox 3 | 2.03 | 0.00181 |
| 219893_at | *CCDC71* | coiled-coil domain containing 71 | 2.03 | 0.00196 |
| 236681_at | *HOXD13* | homeobox D13 | 2.03 | 0.00205 |
| 203842_s_at | *MAPRE3* | microtubule-associated protein, RP/EB family, member 3 | 2.03 | 0.0022 |
| 243738_at | *NMNAT3* | nicotinamide nucleotide adenylyltransferase 3 | 2.03 | 0.0025 |
| 206384_at | *CACNG3* | calcium channel, voltage-dependent, gamma subunit 3 | 2.03 | 0.00268 |
| 203409_at | *DDB2* | damage-specific DNA binding protein 2, 48kDa | 2.03 | 0.00282 |
| 228008_at | *ARID3A* | AT rich interactive domain 3A (BRIGHT-like) | 2.03 | 0.00283 |
| 210715_s_at | *SPINT2* | serine peptidase inhibitor, Kunitz type, 2 | 2.03 | 0.00288 |
| 215488_at |  |  | 2.03 | 0.00315 |
| 241671_x_at | *LINC00340* | long intergenic non-protein coding RNA 340 | 2.03 | 0.00326 |
| 218707_at | *ZNF444* | zinc finger protein 444 | 2.03 | 0.00332 |
| 212680_x_at | *PPP1R14B* | protein phosphatase 1, regulatory (inhibitor) subunit 14B | 2.03 | 0.00353 |
| 233790_at |  |  | 2.03 | 0.00421 |
| 240720_at |  |  | 2.03 | 0.0051 |
| 228116_at |  |  | 2.03 | 0.0052 |
| 240042_at | *FIBCD1* | fibrinogen C domain containing 1 | 2.03 | 0.00544 |
| 214969_at | *MAP3K9* | mitogen-activated protein kinase kinase kinase 9 | 2.03 | 0.00552 |
| 229617_x_at | *AP2A1* | adaptor-related protein complex 2, alpha 1 subunit | 2.03 | 0.00569 |
| 207914_x_at | *EVX1* | even-skipped homeobox 1 | 2.03 | 0.00597 |
| 242415_at | *FBRSL1* | fibrosin-like 1 | 2.03 | 0.00708 |
| 205922_at | *VNN2* | vanin 2 | 2.03 | 0.00749 |
| 202847_at | *PCK2* | phosphoenolpyruvate carboxykinase 2 (mitochondrial) | 2.03 | 0.00783 |
| 1557621_at | *KCP* | kielin/chordin-like protein | 2.03 | 0.00808 |
| 230015_at | *PRCD* | progressive rod-cone degeneration | 2.03 | 0.00949 |
| 240198_at |  |  | 2.03 | 0.01007 |
| 238928_at |  |  | 2.03 | 0.01203 |
| 208085_s_at | *ARHGAP6* | Rho GTPase activating protein 6 | 2.03 | 0.01253 |
| 239139_at | *CPNE9* | copine family member IX | 2.03 | 0.01268 |
| 236080_at |  |  | 2.03 | 0.01285 |
| 208554_at | *POU4F3* | POU class 4 homeobox 3 | 2.03 | 0.01969 |
| 227483_s_at | *UNC93B1* | unc-93 homolog B1 (C. elegans) | 2.03 | 0.02297 |
| 229127_at | *JAM2* | junctional adhesion molecule 2 | 2.03 | 0.02671 |
| 1561538_at |  |  | 2.03 | 0.02678 |
| 219423_x_at | *TNFRSF25* | tumor necrosis factor receptor superfamily, member 25 | 2.03 | 0.03048 |
| 217022_s_at | *IGH* | immunoglobulin heavy locus; immunoglobulin heavy constant alpha 1; immunoglobulin heavy constant alpha 2 (A2m marker) | 2.03 | 0.03323 |
| 235083_at |  |  | 2.03 | 0.03478 |
| 236518_at | *KIAA1984* | KIAA1984 | 2.03 | 0.03598 |
| 1568894_at |  |  | 2.03 | 0.03756 |
| 243199_at |  |  | 2.02 | 0.00012 |
| 218951_s_at | *PLCXD1* | phosphatidylinositol-specific phospholipase C, X domain containing 1 | 2.02 | 0.00061 |
| 234021_at | *EML2* | echinoderm microtubule associated protein like 2 | 2.02 | 0.00097 |
| 207574_s_at | *GADD45B* | growth arrest and DNA-damage-inducible, beta | 2.02 | 0.00104 |
| 216940_x_at | *YBX1* | Y box binding protein 1 | 2.02 | 0.00117 |
| 207005_s_at | *BCL2* | B-cell CLL/lymphoma 2 | 2.02 | 0.00117 |
| 210132_at | *EFNA3* | ephrin-A3 | 2.02 | 0.0015 |
| 231250_at | *HOXB7* | NULL | 2.02 | 0.00175 |
| 240813_at |  |  | 2.02 | 0.00197 |
| 211143_x_at | *NR4A1* | nuclear receptor subfamily 4, group A, member 1 | 2.02 | 0.00211 |
| 231229_at | *HILS1* | histone linker H1 domain, spermatid-specific 1, pseudogene | 2.02 | 0.00247 |
| 205656_at | *PCDH17* | protocadherin 17 | 2.02 | 0.00289 |
| 206436_at | *MPPED1* | metallophosphoesterase domain containing 1 | 2.02 | 0.0033 |
| 1567277_at | *CTTN* | cortactin | 2.02 | 0.0047 |
| 202098_s_at | *PRMT2* | protein arginine methyltransferase 2 | 2.02 | 0.00474 |
| 241040_at |  |  | 2.02 | 0.0058 |
| 205128_x_at | *PTGS1* | prostaglandin-endoperoxide synthase 1 (prostaglandin G/H synthase and cyclooxygenase) | 2.02 | 0.00842 |
| 230114_at |  |  | 2.02 | 0.01047 |
| 1557807_a_at |  |  | 2.02 | 0.0137 |
| 231021_at | *SLC6A19* | solute carrier family 6 (neutral amino acid transporter), member 19 | 2.02 | 0.01428 |
| 231991_at | *CCM2L* | cerebral cavernous malformation 2-like | 2.02 | 0.01716 |
| 218958_at | *C19orf60* | chromosome 19 open reading frame 60 | 2.02 | 0.02185 |
| 211488_s_at | *ITGB8* | integrin, beta 8 | 2.02 | 0.02451 |
| 222217_s_at | *SLC27A3* | solute carrier family 27 (fatty acid transporter), member 3 | 2.02 | 0.02695 |
| 239798_at |  |  | 2.02 | 0.02722 |
| 217213_at | *SLC6A2* | solute carrier family 6 (neurotransmitter transporter, noradrenalin), member 2 | 2.02 | 0.03505 |
| 1552504_a_at | *BRSK1* | BR serine/threonine kinase 1 | 2.02 | 0.03655 |
| 240173_at |  |  | 2.02 | 0.04252 |
| 239474_at |  |  | 2.01 | 0.00035 |
| 238051_x_at | *PWWP2B* | PWWP domain containing 2B | 2.01 | 0.00042 |
| 224409_s_at | *TSSK6* | testis-specific serine kinase 6 | 2.01 | 0.00051 |
| 216499_at |  |  | 2.01 | 0.00058 |
| 235496_at | *HRCT1* | histidine rich carboxyl terminus 1 | 2.01 | 0.00059 |
| 238404_x_at | *SEZ6L2* | seizure related 6 homolog (mouse)-like 2 | 2.01 | 0.00133 |
| 241633_x_at |  |  | 2.01 | 0.00222 |
| 227806_at | *C16orf74* | chromosome 16 open reading frame 74 | 2.01 | 0.00231 |
| 229915_at | *FAM26F* | family with sequence similarity 26, member F | 2.01 | 0.00249 |
| 211323_s_at | *ITPR1* | inositol 1,4,5-trisphosphate receptor, type 1 | 2.01 | 0.00253 |
| 242478_at |  |  | 2.01 | 0.0031 |
| 206981_at | *SCN4A* | sodium channel, voltage-gated, type IV, alpha subunit | 2.01 | 0.00327 |
| 237429_at |  |  | 2.01 | 0.00332 |
| 240896_at |  |  | 2.01 | 0.00364 |
| 202570_s_at | *DLGAP4* | discs, large (Drosophila) homolog-associated protein 4 | 2.01 | 0.00377 |
| 224194_at | *FCRL2* | Fc receptor-like 2 | 2.01 | 0.00425 |
| 226872_at | *RFX2* | regulatory factor X, 2 (influences HLA class II expression) | 2.01 | 0.00428 |
| 214524_at | *GHRH* | growth hormone releasing hormone | 2.01 | 0.00448 |
| 239627_at |  |  | 2.01 | 0.00462 |
| 230963_at | *EMX2OS* | EMX2 opposite strand/antisense RNA | 2.01 | 0.00481 |
| 217207_s_at | *BTNL3* | butyrophilin-like 3 | 2.01 | 0.00491 |
| 220130_x_at | *LTB4R2* | leukotriene B4 receptor 2 | 2.01 | 0.00661 |
| 236496_at | *DEGS2* | delta(4)-desaturase, sphingolipid 2 | 2.01 | 0.00724 |
| 212734_x_at | *RPL13* | ribosomal protein L13; small nucleolar RNA, C/D box 68 | 2.01 | 0.01007 |
| 212312_at | *BCL2L1* | BCL2-like 1 | 2.01 | 0.01023 |
| 201792_at | *AEBP1* | AE binding protein 1 | 2.01 | 0.01028 |
| 1562078_at |  |  | 2.01 | 0.01051 |
| 231587_at | *APOC3* | apolipoprotein C-III | 2.01 | 0.01059 |
| 238109_at |  |  | 2.01 | 0.01106 |
| 1564122_at | *LINC00514* | long intergenic non-protein coding RNA 514 | 2.01 | 0.01183 |
| 1553424_at | *C12orf40* | chromosome 12 open reading frame 40 | 2.01 | 0.01303 |
| 1556606_at | *NAV2* | neuron navigator 2 | 2.01 | 0.0132 |
| 244655_at |  |  | 2.01 | 0.0153 |
| 238013_at | *PLEKHA2* | pleckstrin homology domain containing, family A (phosphoinositide binding specific) member 2 | 2.01 | 0.01617 |
| 1561849_at | *PKD1L2* | polycystic kidney disease 1-like 2 | 2.01 | 0.01665 |
| 209230_s_at | *NUPR1* | nuclear protein, transcriptional regulator, 1 | 2.01 | 0.01705 |
| 231153_at | *C16orf86* | chromosome 16 open reading frame 86 | 2.01 | 0.01923 |
| 238083_at | *PARP10* | poly (ADP-ribose) polymerase family, member 10 | 2.01 | 0.01988 |
| 221557_s_at | *LEF1* | lymphoid enhancer-binding factor 1 | 2.01 | 0.02053 |
| 243428_at | *KCNQ1OT1* | KCNQ1 opposite strand/antisense transcript 1 (non-protein coding) | 2.01 | 0.02425 |
| 71933_at | *WNT6* | wingless-type MMTV integration site family, member 6 | 2.01 | 0.02493 |
| 207530_s_at | *CDKN2B* | cyclin-dependent kinase inhibitor 2B (p15, inhibits CDK4) | 2.01 | 0.02683 |
| 235861_at |  |  | 2.01 | 0.02719 |
| 236274_at | *EIF3B* | eukaryotic translation initiation factor 3, subunit B | 2.01 | 0.03286 |
| 223767_at | *GPR84* | G protein-coupled receptor 84 | 2.01 | 0.03855 |
| 241003_at |  |  | 2 | 0.00027 |
| 240984_at |  |  | 2 | 0.00036 |
| 206128_at | *ADRA2C* | adrenoceptor alpha 2C | 2 | 0.00044 |
| 202482_x_at | *RANBP1* | RAN binding protein 1 | 2 | 0.00092 |
| 236845_at | *TRIM62* | tripartite motif containing 62 | 2 | 0.00098 |
| 233669_s_at | *TRIM54* | tripartite motif containing 54 | 2 | 0.00125 |
| 235107_at | *PIAS2* | protein inhibitor of activated STAT, 2 | 2 | 0.00162 |
| 233407_at |  |  | 2 | 0.00167 |
| 217014_s_at | *AZGP1* | alpha-2-glycoprotein 1, zinc-binding; alpha-2-glycoprotein 1, zinc-binding pseudogene 1 | 2 | 0.00172 |
| 222561_at | *LANCL2* | LanC lantibiotic synthetase component C-like 2 (bacterial) | 2 | 0.00179 |
| 236635_at | *ZNF667* | zinc finger protein 667 | 2 | 0.00181 |
| 223546_x_at | *LUC7L* | LUC7-like (S. cerevisiae) | 2 | 0.00195 |
| 231529_at | *MEG3* | maternally expressed 3 (non-protein coding) | 2 | 0.00242 |
| 236169_at |  |  | 2 | 0.00289 |
| 232878_at | *NR2F2-AS1* | NR2F2 antisense RNA 1 | 2 | 0.00309 |
| 237900_at | *KLHDC4* | kelch domain containing 4 | 2 | 0.00336 |
| 214957_at | *ACTL8* | actin-like 8 | 2 | 0.00362 |
| 206259_at | *PROC* | protein C (inactivator of coagulation factors Va and VIIIa) | 2 | 0.00366 |
| 205814_at | *GRM3* | glutamate receptor, metabotropic 3 | 2 | 0.00367 |
| 216905_s_at | *ST14* | suppression of tumorigenicity 14 (colon carcinoma) | 2 | 0.00426 |
| 244245_at | *ANKRD9* | ankyrin repeat domain 9 | 2 | 0.00431 |
| 239544_at |  |  | 2 | 0.00465 |
| 1557275_a_at | *TLCD2* | TLC domain containing 2 | 2 | 0.00505 |
| 230932_at | *RNF130* | NULL | 2 | 0.00619 |
| 239508_x_at | *CCDC108* | coiled-coil domain containing 108 | 2 | 0.00666 |
| 216485_s_at | *TPSAB1* | tryptase alpha/beta 1 | 2 | 0.00724 |
| 220316_at | *NPAS3* | neuronal PAS domain protein 3 | 2 | 0.00746 |
| 203926_x_at | *ATP5D* | ATP synthase, H+ transporting, mitochondrial F1 complex, delta subunit | 2 | 0.00746 |
| 1553633_s_at | *SLC9B1* | solute carrier family 9, subfamily B (NHA1, cation proton antiporter 1), member 1 | 2 | 0.00784 |
| 202977_s_at | *CREBZF* | CREB/ATF bZIP transcription factor | 2 | 0.00798 |
| 202592_at | *BLOC1S1* | biogenesis of lysosomal organelles complex-1, subunit 1 | 2 | 0.00825 |
| 224141_at | *FLJ38668* | uncharacterized LOC644903 | 2 | 0.00846 |
| 208038_at | *IL1RL2* | interleukin 1 receptor-like 2 | 2 | 0.00988 |
| 1552848_a_at | *PTCHD1* | patched domain containing 1 | 2 | 0.01117 |
| 203683_s_at | *VEGFB* | vascular endothelial growth factor B | 2 | 0.01156 |
| 227574_at | *OBSL1* | obscurin-like 1 | 2 | 0.01332 |
| 227825_at | *NAIF1* | nuclear apoptosis inducing factor 1 | 2 | 0.01375 |
| 233963_at | *H2BFXP* | H2B histone family, member X, pseudogene | 2 | 0.01404 |
| 1553137_s_at | *KLF11* | Kruppel-like factor 11 | 2 | 0.01449 |
| 233416_at |  |  | 2 | 0.0165 |
| 1564233_at | *FLJ33534* | uncharacterized LOC285150 | 2 | 0.01871 |
| 210587_at | *INHBE* | inhibin, beta E | 2 | 0.02019 |
| 204746_s_at | *PICK1* | protein interacting with PRKCA 1 | 2 | 0.02102 |
| 1569775_at | *RNF157* | ring finger protein 157 | 2 | 0.02174 |
| 244499_at | *THAP2* | THAP domain containing, apoptosis associated protein 2 | 2 | 0.02176 |
| 237732_at | *PRR9* | proline rich 9 | 2 | 0.02678 |
| 227371_at | *BAIAP2L1* | BAI1-associated protein 2-like 1 | 2 | 0.03087 |
| 230913_at |  |  | 2 | 0.0325 |
