# Supplementary material for: Transcriptomic Analysis of Human Astrocytes In Vitro Reveals Hypoxia-Induced Mitochondrial Dysfunction, Modulation of Metabolism, and Dysregulation of the Immune Response
Source: Int J Mol Sci. 2020 Oct 28;21(21):8028. doi: 10.3390/ijms21218028 (PMC7672558; doi:10.3390/ijms21218028)
Supplement: Supplementary file 1 [file ijms-21-08028-s001.zip › Supplementary Table 2.docx]

**Supplementary Table 2.** Genes downregulated in human astrocytes in response to hypoxia (FC≥2, p<0.05)

| **Transcript** | | | **Gene symbol** | | **Gene name** | **FC** | **p-value** |
| --- | --- | --- | --- | --- | --- | --- | --- |
| 201479_at | | | *DKC1* | dyskeratosis congenita 1, dyskerin; microRNA 644b; small nucleolar RNA, H/ACA box 56 | | -2 | 1.8x10^-5^ |
| 1555982_at | | | *ZFYVE16* | zinc finger, FYVE domain containing 16 | | -2 | 6.8x10^-5^ |
| 1569594_a_at | | | *NEMF* | nuclear export mediator factor | | -2 | 0.00017 |
| 205677_s_at | | | *DLEU1* | deleted in lymphocytic leukemia 1 (non-protein coding) | | -2 | 0.00031 |
| 223292_s_at | | | *MRPS15* | mitochondrial ribosomal protein S15 | | -2 | 0.00032 |
| 224826_at | | | *GPCPD1* | glycerophosphocholine phosphodiesterase GDE1 homolog (S. cerevisiae) | | -2 | 0.00061 |
| 228634_s_at | | | *YBX3* | Y box binding protein 3 | | -2 | 0.00074 |
| 236568_at | | |  |  | | -2 | 0.00096 |
| 226711_at | | | *FOXN2* | forkhead box N2 | | -2 | 0.00103 |
| 200740_s_at | | | *SUMO3* | small ubiquitin-like modifier 3 | | -2 | 0.00106 |
| 229061_s_at | | | *SLC25A13* | solute carrier family 25 (aspartate/glutamate carrier), member 13 | | -2 | 0.00115 |
| 204554_at | | | *PPP1R3D* | protein phosphatase 1, regulatory subunit 3D | | -2 | 0.00122 |
| 213360_s_at | | | *POM121* | POM121 transmembrane nucleoporin; POM121 transmembrane nucleoporin C | | -2 | 0.00156 |
| 209527_at | | | *EXOSC2* | exosome component 2 | | -2 | 0.00165 |
| 222991_s_at | | | *UBQLN1* | ubiquilin 1 | | -2 | 0.00188 |
| 203396_at | | | *PSMA4* | proteasome (prosome, macropain) subunit, alpha type, 4 | | -2 | 0.00234 |
| 222530_s_at | | | *MKKS* | McKusick-Kaufman syndrome | | -2 | 0.00239 |
| 228505_s_at | | | *TMEM170A* | transmembrane protein 170A | | -2 | 0.00288 |
| 213623_at | | | *KIF3A* | kinesin family member 3A | | -2 | 0.00328 |
| 202088_at | | | *SLC39A6* | solute carrier family 39 (zinc transporter), member 6 | | -2 | 0.00338 |
| 218331_s_at | | | *FAM208B* | family with sequence similarity 208, member B | | -2 | 0.00383 |
| 236300_at | | | *PDE3A* | phosphodiesterase 3A, cGMP-inhibited | | -2 | 0.0039 |
| 229856_s_at | | | *PITHD1* | PITH (C-terminal proteasome-interacting domain of thioredoxin-like) domain containing 1 | | -2 | 0.00414 |
| 213134_x_at | | | *BTG3* | BTG family, member 3 | | -2 | 0.00443 |
| 227997_at | | | *IL17RD* | interleukin 17 receptor D | | -2 | 0.00468 |
| 219367_s_at | | | *NRP2* | neuropilin 2 | | -2 | 0.00495 |
| 212793_at | | | *DAAM2* | dishevelled associated activator of morphogenesis 2 | | -2 | 0.00515 |
| 241861_at | | | *SYCP3* | synaptonemal complex protein 3 | | -2 | 0.00534 |
| 230773_at | | | *ZNF385D* | zinc finger protein 385D | | -2 | 0.00535 |
| 205162_at | | | *ERCC8* | excision repair cross-complementing rodent repair deficiency, complementation group 8 | | -2 | 0.00539 |
| 201994_at | | | *MORF4L2* | mortality factor 4 like 2 | | -2 | 0.00563 |
| 1562777_at | | | *ERV3-1* | endogenous retrovirus group 3, member 1 | | -2 | 0.00572 |
| 201458_s_at | | | *BUB3* | BUB3 mitotic checkpoint protein | | -2 | 0.00605 |
| 201661_s_at | | | *ACSL3* | acyl-CoA synthetase long-chain family member 3 | | -2 | 0.00632 |
| 224399_at | | | *PDCD1LG2* | programmed cell death 1 ligand 2 | | -2 | 0.00658 |
| 218289_s_at | | | *UBA5* | ubiquitin-like modifier activating enzyme 5 | | -2 | 0.00756 |
| 200080_s_at | | | *H3F3A* | H3 histone, family 3A; H3 histone, family 3A, pseudogene 4; H3 histone, family 3B (H3.3B) | | -2 | 0.00814 |
| 213304_at | | | *FAM179B* | family with sequence similarity 179, member B | | -2 | 0.00828 |
| 212287_at | | | *SUZ12* | SUZ12 polycomb repressive complex 2 subunit | | -2 | 0.00841 |
| 223486_at | | | *GTPBP8* | GTP-binding protein 8 (putative) | | -2 | 0.00904 |
| 209628_at | | | *NXT2* | nuclear transport factor 2-like export factor 2 | | -2 | 0.01108 |
| 212220_at | | | *PSME4* | proteasome (prosome, macropain) activator subunit 4 | | -2 | 0.0113 |
| 222889_at | | | *DCLRE1B* | DNA cross-link repair 1B | | -2 | 0.01435 |
| 205992_s_at | | | *IL15* | interleukin 15 | | -2 | 0.01442 |
| 1555725_a_at | | | *RGS5* | regulator of G-protein signaling 5 | | -2 | 0.01479 |
| 233490_at | | | *DCTN4* | dynactin 4 (p62) | | -2 | 0.01835 |
| 223661_at | | | *NUCKS1* | nuclear casein kinase and cyclin-dependent kinase substrate 1 | | -2 | 0.01995 |
| 233677_at | | |  |  | | -2 | 0.02041 |
| 220768_s_at | | | *CSNK1G3* | casein kinase 1, gamma 3 | | -2 | 0.0245 |
| 218979_at | | | *RMI1* | RecQ mediated genome instability 1 | | -2 | 0.02695 |
| 235196_at | | | *CDC73* | cell division cycle 73 | | -2 | 0.02917 |
| 203429_s_at | | | *SUCO* | SUN domain containing ossification factor | | -2 | 0.03003 |
| 1554155_at | | | *MCPH1* | microcephalin 1 | | -2 | 0.03059 |
| 222111_at | | | *FAM63B* | family with sequence similarity 63, member B | | -2 | 0.03354 |
| 228573_at | | | *ANTXR2* | anthrax toxin receptor 2 | | -2 | 0.03362 |
| 224901_at | | | *SCD5* | stearoyl-CoA desaturase 5 | | -2 | 0.03956 |
| 212911_at | | | *DNAJC16* | DnaJ (Hsp40) homolog, subfamily C, member 16 | | -2 | 0.03984 |
| 206765_at | | | *KCNJ2* | potassium inwardly-rectifying channel, subfamily J, member 2 | | -2 | 0.0416 |
| 223487_x_at | | | *GNB4* | guanine nucleotide binding protein (G protein), beta polypeptide 4 | | -2 | 0.04295 |
| 208754_s_at | | | *NAP1L1* | nucleosome assembly protein 1-like 1 | | -2 | 0.04664 |
| 241174_at | | | *AP4E1* | adaptor-related protein complex 4, epsilon 1 subunit | | -2 | 0.04705 |
| 201700_at | | | *CCND3* | cyclin D3 | | -2.01 | 1.8x10^-5^ |
| 233233_at | | | *RASSF3* | Ras association (RalGDS/AF-6) domain family member 3 | | -2.01 | 4.6x10^-5^ |
| 202613_at | | | *CTPS1* | CTP synthase 1 | | -2.01 | 0.0002 |
| 204252_at | | | *CDK2* | cyclin-dependent kinase 2 | | -2.01 | 0.00027 |
| 202900_s_at | | | *NUP88* | nucleoporin 88kDa | | -2.01 | 0.00028 |
| 213009_s_at | | | *TRIM37* | tripartite motif containing 37 | | -2.01 | 0.00039 |
| 229512_at | | | *FAM120C* | family with sequence similarity 120C | | -2.01 | 0.00082 |
| 1558762_a_at | | | *ZNF789* | zinc finger protein 789 | | -2.01 | 0.00092 |
| 209754_s_at | | | *TMPO* | thymopoietin | | -2.01 | 0.00117 |
| 222689_at | | | *ACER3* | alkaline ceramidase 3 | | -2.01 | 0.00129 |
| 214214_s_at | | | *C1QBP* | complement component 1, q subcomponent binding protein | | -2.01 | 0.00217 |
| 242155_x_at | | | *RFFL* | ring finger and FYVE-like domain containing E3 ubiquitin protein ligase | | -2.01 | 0.00275 |
| 221669_s_at | | | *ACAD8* | acyl-CoA dehydrogenase family, member 8 | | -2.01 | 0.00276 |
| 240044_x_at | | | *TNRC6B* | trinucleotide repeat containing 6B | | -2.01 | 0.00311 |
| 232341_x_at | | | *HABP4* | hyaluronan binding protein 4 | | -2.01 | 0.00324 |
| 235180_at | | | *STYX* | serine/threonine/tyrosine interacting protein | | -2.01 | 0.00349 |
| 230291_s_at | | |  |  | | -2.01 | 0.0035 |
| 1553979_at | | | *ZNF121* | zinc finger protein 121 | | -2.01 | 0.00362 |
| 224971_at | | | *C2orf15* | chromosome 2 open reading frame 15; mitochondrial ribosomal protein L30 | | -2.01 | 0.00391 |
| 209648_x_at | | | *SOCS5* | suppressor of cytokine signaling 5 | | -2.01 | 0.00392 |
| 203132_at | | | *RB1* | retinoblastoma 1 | | -2.01 | 0.00417 |
| 203711_s_at | | | *HIBCH* | 3-hydroxyisobutyryl-CoA hydrolase | | -2.01 | 0.00485 |
| 1554599_x_at | | |  |  | | -2.01 | 0.00549 |
| 232432_s_at | | | *SLC30A5* | solute carrier family 30 (zinc transporter), member 5 | | -2.01 | 0.00656 |
| 203989_x_at | | | *F2R* | coagulation factor II (thrombin) receptor | | -2.01 | 0.00707 |
| 204067_at | | | *SUOX* | sulfite oxidase | | -2.01 | 0.00741 |
| 202983_at | | | *HLTF* | helicase-like transcription factor | | -2.01 | 0.00742 |
| 212417_at | | | *SCAMP1* | secretory carrier membrane protein 1 | | -2.01 | 0.00776 |
| 1558028_x_at | | |  |  | | -2.01 | 0.00888 |
| 228115_at | | | *GAREM* | GRB2 associated, regulator of MAPK1 | | -2.01 | 0.00905 |
| 213133_s_at | | | *GCSH* | glycine cleavage system protein H (aminomethyl carrier); glycine cleavage system H protein, mitochondrial-like | | -2.01 | 0.00932 |
| 210907_s_at | | | *PDCD10* | programmed cell death 10 | | -2.01 | 0.01005 |
| 228249_at | | | *C11orf74* | chromosome 11 open reading frame 74 | | -2.01 | 0.01037 |
| 224764_at | | | *ARHGAP21* | Rho GTPase activating protein 21 | | -2.01 | 0.01287 |
| 211676_s_at | | | *IFNGR1* | interferon gamma receptor 1 | | -2.01 | 0.0144 |
| 204796_at | | | *EML1* | echinoderm microtubule associated protein like 1 | | -2.01 | 0.01506 |
| 217833_at | | | *SYNCRIP* | synaptotagmin binding, cytoplasmic RNA interacting protein | | -2.01 | 0.0177 |
| 243852_at | | | *LUC7L2* | LUC7-like 2 (S. cerevisiae) | | -2.01 | 0.01772 |
| 227433_at | | | *KIAA2018* | KIAA2018 | | -2.01 | 0.01888 |
| 229166_s_at | | | *GATAD2A* | GATA zinc finger domain containing 2A | | -2.01 | 0.01983 |
| 230891_at | | | *TUBE1* | tubulin, epsilon 1 | | -2.01 | 0.01986 |
| 209501_at | | | *CDR2* | cerebellar degeneration-related protein 2, 62kDa | | -2.01 | 0.02003 |
| 207305_s_at | | | *TRAPPC8* | trafficking protein particle complex 8 | | -2.01 | 0.02149 |
| 208644_at | | | *PARP1* | poly (ADP-ribose) polymerase 1 | | -2.01 | 0.02195 |
| 226574_at | | | *PSPC1* | paraspeckle component 1 | | -2.01 | 0.02346 |
| 224635_s_at | | | *BIRC6* | baculoviral IAP repeat containing 6 | | -2.01 | 0.02499 |
| 241245_at | | | *SRSF4* | serine/arginine-rich splicing factor 4 | | -2.01 | 0.02558 |
| 238592_at | | |  |  | | -2.01 | 0.02655 |
| 229500_at | | | *SLC30A9* | solute carrier family 30 (zinc transporter), member 9 | | -2.01 | 0.03003 |
| 206022_at | | | *NDP* | Norrie disease (pseudoglioma) | | -2.01 | 0.03342 |
| 228561_at | | | *CDC37L1* | cell division cycle 37-like 1 | | -2.01 | 0.03391 |
| 202261_at | | | *VPS72* | vacuolar protein sorting 72 homolog (S. cerevisiae) | | -2.01 | 0.03631 |
| 206247_at | | | *MICB* | MHC class I polypeptide-related sequence B | | -2.01 | 0.04164 |
| 218411_s_at | | | *MBIP* | MAP3K12 binding inhibitory protein 1 | | -2.01 | 0.04237 |
| 1569061_at | | | *IQGAP3* | IQ motif containing GTPase activating protein 3 | | -2.01 | 0.04362 |
| 233261_at | | | *EBF1* | early B-cell factor 1 | | -2.01 | 0.04877 |
| 216519_s_at | | | *PROSC* | proline synthetase co-transcribed homolog (bacterial) | | -2.02 | 1x10^-6^ |
| 201971_s_at | | | *ATP6V1A* | ATPase, H+ transporting, lysosomal 70kDa, V1 subunit A | | -2.02 | 0.00013 |
| 202883_s_at | | | *PPP2R1B* | protein phosphatase 2, regulatory subunit A, beta | | -2.02 | 0.00018 |
| 1554549_a_at | | | *WDR20* | WD repeat domain 20 | | -2.02 | 0.00023 |
| 200806_s_at | | | *HSPD1* | heat shock 60kDa protein 1 (chaperonin) | | -2.02 | 0.00096 |
| 205419_at | | | *GPR183* | G protein-coupled receptor 183 | | -2.02 | 0.00098 |
| 202743_at | | | *PIK3R3* | phosphoinositide-3-kinase, regulatory subunit 3 (gamma) | | -2.02 | 0.00117 |
| 234000_s_at | | | *PTPLAD1* | protein tyrosine phosphatase-like A domain containing 1 | | -2.02 | 0.00177 |
| 240013_at | | |  |  | | -2.02 | 0.00197 |
| 217879_at | | | *CDC27* | cell division cycle 27 | | -2.02 | 0.00237 |
| 242953_at | | | *ZNF234* | zinc finger protein 234 | | -2.02 | 0.00264 |
| 209481_at | | | *SNRK* | SNF related kinase | | -2.02 | 0.00308 |
| 203420_at | | | *FAM8A1* | family with sequence similarity 8, member A1 | | -2.02 | 0.00365 |
| 235101_at | | | *FNBP4* | formin binding protein 4 | | -2.02 | 0.00386 |
| 203250_at | | | *SCAF8* | SR-related CTD-associated factor 8 | | -2.02 | 0.00432 |
| 213857_s_at | | | *CD47* | CD47 molecule | | -2.02 | 0.00486 |
| 242828_at | | | *FIGN* | fidgetin | | -2.02 | 0.00488 |
| 238822_at | |  | |  | | -2.02 | 0.00494 |
| 60474_at | | *FERMT1* | | fermitin family member 1 | | -2.02 | 0.00515 |
| 235019_at | | *CPM* | | carboxypeptidase M | | -2.02 | 0.00533 |
| 200014_s_at | | *HNRNPC* | | heterogeneous nuclear ribonucleoprotein C (C1/C2) | | -2.02 | 0.0056 |
| 217837_s_at | *CHMP3* | | | charged multivesicular body protein 3; RNF103-CHMP3 readthrough | | -2.02 | 0.00593 |
| 237400_at |  | | |  | | -2.02 | 0.00607 |
| 218713_at | *NARG2* | | | NMDA receptor regulated 2 | | -2.02 | 0.00644 |
| 216996_s_at | *FASTKD2* | | | FAST kinase domains 2 | | -2.02 | 0.00696 |
| 211097_s_at | *PBX2* | | | pre-B-cell leukemia homeobox 2 | | -2.02 | 0.00701 |
| 229480_at | *MAGI2-AS3* | | | MAGI2 antisense RNA 3 | | -2.02 | 0.00725 |
| 225332_at | *OIP5-AS1* | | | OIP5 antisense RNA 1 | | -2.02 | 0.00746 |
| 222426_at | *MAPKAP1* | | | mitogen-activated protein kinase associated protein 1 | | -2.02 | 0.0079 |
| 229986_at |  | | |  | | -2.02 | 0.00849 |
| 225665_at | *ZAK* | | | sterile alpha motif and leucine zipper containing kinase AZK | | -2.02 | 0.00898 |
| 227236_at | *TSPAN2* | | | tetraspanin 2 | | -2.02 | 0.00933 |
| 227413_at | *UBLCP1* | | | ubiquitin-like domain containing CTD phosphatase 1 | | -2.02 | 0.01099 |
| 1553546_at |  | | |  | | -2.02 | 0.01238 |
| 209175_at | *SEC23IP* | | | SEC23 interacting protein | | -2.02 | 0.01246 |
| 219292_at | *THAP1* | | | THAP domain containing, apoptosis associated protein 1 | | -2.02 | 0.01317 |
| 219029_at | *C5orf28* | | | chromosome 5 open reading frame 28 | | -2.02 | 0.01324 |
| 227167_s_at | *RASSF3* | | | Ras association (RalGDS/AF-6) domain family member 3 | | -2.02 | 0.01465 |
| 212942_s_at | *KIAA1199* | | | KIAA1199 | | -2.02 | 0.01538 |
| 236233_at | *TRIM32* | | | tripartite motif containing 32 | | -2.02 | 0.01547 |
| 233228_at |  | | |  | | -2.02 | 0.01753 |
| 215820_x_at | *SNX13* | | | sorting nexin 13 | | -2.02 | 0.01858 |
| 221044_s_at | *TRIM34* | | | tripartite motif containing 34; TRIM6-TRIM34 readthrough | | -2.02 | 0.01954 |
| 243915_at |  | | |  | | -2.02 | 0.02132 |
| 208845_at | *VDAC3* | | | voltage-dependent anion channel 3 | | -2.02 | 0.02141 |
| 244455_at | *KCNT2* | | | potassium channel, subfamily T, member 2 | | -2.02 | 0.02371 |
| 204529_s_at | *TOX* | | | thymocyte selection-associated high mobility group box | | -2.02 | 0.02374 |
| 207507_s_at | *ATP5G3* | | | ATP synthase, H+ transporting, mitochondrial Fo complex, subunit C3 (subunit 9) | | -2.02 | 0.02477 |
| 1555284_at | *ALS2* | | | amyotrophic lateral sclerosis 2 (juvenile) | | -2.02 | 0.02745 |
| 223775_at | *HHIP* | | | hedgehog interacting protein | | -2.02 | 0.02772 |
| 221858_at | *TBC1D12* | | | TBC1 domain family, member 12 | | -2.02 | 0.0278 |
| 211931_s_at | *HNRNPA3* | | | heterogeneous nuclear ribonucleoprotein A3; heterogeneous nuclear ribonucleoprotein A3 pseudogene 1 | | -2.02 | 0.03057 |
| 218521_s_at | *UBE2W* | | | ubiquitin-conjugating enzyme E2W (putative) | | -2.02 | 0.03488 |
| 221745_at | *DCAF7* | | | DDB1 and CUL4 associated factor 7 | | -2.02 | 0.0359 |
| 223330_s_at | *SUGT1* | | | SGT1, suppressor of G2 allele of SKP1 (S. cerevisiae) | | -2.02 | 0.03666 |
| 206241_at | *KPNA5* | | | karyopherin alpha 5 (importin alpha 6) | | -2.02 | 0.03964 |
| 212950_at | *GPR116* | | | G protein-coupled receptor 116 | | -2.03 | 0.00017 |
| 201112_s_at | *CSE1L* | | | CSE1 chromosome segregation 1-like (yeast) | | -2.03 | 0.00049 |
| 244115_at | *FAM126A* | | | family with sequence similarity 126, member A | | -2.03 | 0.00066 |
| 209840_s_at | *LRRN3* | | | leucine rich repeat neuronal 3 | | -2.03 | 0.00072 |
| 219675_s_at | *UXS1* | | | UDP-glucuronate decarboxylase 1 | | -2.03 | 0.00106 |
| 227788_at | *USP13* | | | ubiquitin specific peptidase 13 (isopeptidase T-3) | | -2.03 | 0.00199 |
| 225447_at | *GPD2* | | | glycerol-3-phosphate dehydrogenase 2 (mitochondrial) | | -2.03 | 0.00208 |
| 206624_at | *USP9Y* | | | ubiquitin specific peptidase 9, Y-linked | | -2.03 | 0.00235 |
| 243857_at | *MORF4L2* | | | mortality factor 4 like 2 | | -2.03 | 0.00241 |
| 1565823_at | *SEPT7* | | | septin 7 | | -2.03 | 0.00265 |
| 1569022_a_at | *PIK3C2A* | | | phosphatidylinositol-4-phosphate 3-kinase, catalytic subunit type 2 alpha | | -2.03 | 0.00297 |
| 202703_at | *DUSP11* | | | dual specificity phosphatase 11 (RNA/RNP complex 1-interacting) | | -2.03 | 0.00326 |
| 1569894_at | *PPP2R3C* | | | protein phosphatase 2, regulatory subunit B'', gamma | | -2.03 | 0.00335 |
| 216841_s_at |  | | |  | | -2.03 | 0.00339 |
| 225049_at | *BLOC1S2* | | | biogenesis of lysosomal organelles complex-1, subunit 2 | | -2.03 | 0.00381 |
| 242283_at | *DNAH14* | | | dynein, axonemal, heavy chain 14 | | -2.03 | 0.00393 |
| 211337_s_at | *TUBGCP4* | | | tubulin, gamma complex associated protein 4 | | -2.03 | 0.00452 |
| 207361_at | *HBP1* | | | HMG-box transcription factor 1 | | -2.03 | 0.00466 |
| 200639_s_at | *YWHAZ* | | | tyrosine 3-monooxygenase/tryptophan 5-monooxygenase activation protein, zeta polypeptide | | -2.03 | 0.00472 |
| 235222_x_at | *XIAP* | | | X-linked inhibitor of apoptosis | | -2.03 | 0.00542 |
| 218047_at | *OSBPL9* | | | oxysterol binding protein-like 9 | | -2.03 | 0.00567 |
| 242233_at |  | | |  | | -2.03 | 0.00614 |
| 243366_s_at |  | | |  | | -2.03 | 0.00679 |
| 236115_at | *HTR7P1* | | | 5-hydroxytryptamine (serotonin) receptor 7 pseudogene 1 | | -2.03 | 0.00693 |
| 228443_s_at | *SETD8* | | | SET domain containing (lysine methyltransferase) 8 | | -2.03 | 0.00761 |
| 242100_at | *CHSY3* | | | chondroitin sulfate synthase 3 | | -2.03 | 0.00761 |
| 242526_at |  | | |  | | -2.03 | 0.0085 |
| 226816_s_at | *KIAA1143* | | | KIAA1143 | | -2.03 | 0.00966 |
| 1552970_s_at | *ZMYM6* | | | zinc finger, MYM-type 6 | | -2.03 | 0.00999 |
| 222589_at | *NLK* | | | nemo-like kinase | | -2.03 | 0.01085 |
| 1560318_at | *ARHGAP29* | | | Rho GTPase activating protein 29 | | -2.03 | 0.01175 |
| 212582_at | *OSBPL8* | | | oxysterol binding protein-like 8 | | -2.03 | 0.01215 |
| 209753_s_at | *TMPO* | | | thymopoietin | | -2.03 | 0.01231 |
| 241198_s_at | *C11orf70* | | | chromosome 11 open reading frame 70 | | -2.03 | 0.01498 |
| 225455_at | *TADA1* | | | transcriptional adaptor 1 | | -2.03 | 0.01542 |
| 229285_at | *RNASEL* | | | ribonuclease L (2',5'-oligoisoadenylate synthetase-dependent) | | -2.03 | 0.01624 |
| 241574_s_at | *IGF2BP1* | | | insulin-like growth factor 2 mRNA binding protein 1 | | -2.03 | 0.01634 |
| 235398_at | *ZNF805* | | | zinc finger protein 805 | | -2.03 | 0.02021 |
| 200624_s_at | *MATR3* | | | matrin 3; small nucleolar RNA host gene 4 (non-protein coding) | | -2.03 | 0.02069 |
| 226785_at | *ATP11C* | | | ATPase, class VI, type 11C | | -2.03 | 0.02162 |
| 210718_s_at | *ARL17A* | | | ADP-ribosylation factor-like 17A; ADP-ribosylation factor-like 17B; ADP-ribosylation factor-like protein 17-like | | -2.03 | 0.02285 |
| 1553725_s_at |  | | |  | | -2.03 | 0.02296 |
| 227121_at |  | | |  | | -2.03 | 0.02323 |
| 230669_at | *RASA2* | | | RAS p21 protein activator 2 | | -2.03 | 0.02371 |
| 212458_at | *SPRED2* | | | sprouty-related, EVH1 domain containing 2 | | -2.03 | 0.02438 |
| 217506_at |  | | |  | | -2.03 | 0.02546 |
| 205110_s_at | *FGF13* | | | fibroblast growth factor 13 | | -2.03 | 0.0297 |
| 230569_at | *KIAA1430* | | | KIAA1430 | | -2.03 | 0.02993 |
| 1553191_at | *DST* | | | dystonin | | -2.03 | 0.03437 |
| 212262_at | *QKI* | | | QKI, KH domain containing, RNA binding | | -2.03 | 0.03597 |
| 215629_s_at | *DLEU2* | | | deleted in lymphocytic leukemia 2 (non-protein coding); deleted in lymphocytic leukemia 2-like | | -2.03 | 0.04683 |
| 225275_at | *EDIL3* | | | EGF-like repeats and discoidin I-like domains 3 | | -2.03 | 0.04898 |
| 202170_s_at | *AASDHPPT* | | | aminoadipate-semialdehyde dehydrogenase-phosphopantetheinyl transferase | | -2.04 | 0.00002 |
| 201920_at | *SLC20A1* | | | solute carrier family 20 (phosphate transporter), member 1 | | -2.04 | 7.8x10^-5^ |
| 227315_x_at | *PROSC* | | | proline synthetase co-transcribed homolog (bacterial) | | -2.04 | 0.00025 |
| 225993_at | *EARS2* | | | glutamyl-tRNA synthetase 2, mitochondrial | | -2.04 | 0.00049 |
| 204339_s_at | *RGS4* | | | regulator of G-protein signaling 4 | | -2.04 | 0.00054 |
| 200713_s_at | *MAPRE1* | | | microtubule-associated protein, RP/EB family, member 1 | | -2.04 | 0.0006 |
| 225556_at | *VMA21* | | | VMA21 vacuolar H+-ATPase homolog (S. cerevisiae) | | -2.04 | 0.00094 |
| 228176_at | *S1PR3* | | | sphingosine-1-phosphate receptor 3 | | -2.04 | 0.00104 |
| 222556_at | *ALG5* | | | ALG5, dolichyl-phosphate beta-glucosyltransferase | | -2.04 | 0.00106 |
| 201847_at | *LIPA* | | | lipase A, lysosomal acid, cholesterol esterase | | -2.04 | 0.00142 |
| 214791_at | *SP140L* | | | SP140 nuclear body protein-like | | -2.04 | 0.00163 |
| 241330_x_at |  | | |  | | -2.04 | 0.00212 |
| 229757_at |  | | |  | | -2.04 | 0.00241 |
| 202070_s_at | *IDH3A* | | | isocitrate dehydrogenase 3 (NAD+) alpha | | -2.04 | 0.00249 |
| 222562_s_at | *TNKS2* | | | tankyrase, TRF1-interacting ankyrin-related ADP-ribose polymerase 2 | | -2.04 | 0.0029 |
| 227246_at | *PLRG1* | | | pleiotropic regulator 1 | | -2.04 | 0.00296 |
| 225343_at | *TMED8* | | | transmembrane emp24 protein transport domain containing 8 | | -2.04 | 0.003 |
| 235283_at | *INTS6* | | | integrator complex subunit 6 | | -2.04 | 0.00314 |
| 204603_at | *EXO1* | | | exonuclease 1 | | -2.04 | 0.00319 |
| 219581_at | *TSEN2* | | | tRNA splicing endonuclease 2 homolog (S. cerevisiae) | | -2.04 | 0.00398 |
| 218953_s_at | *PCYOX1L* | | | prenylcysteine oxidase 1 like | | -2.04 | 0.00416 |
| 219007_at | *NUP43* | | | nucleoporin 43kDa | | -2.04 | 0.00418 |
| 208515_at | *HIST1H2BM* | | | histone cluster 1, H2bm | | -2.04 | 0.005 |
| 1552486_s_at | *LACTB* | | | lactamase, beta | | -2.04 | 0.00534 |
| 243704_at | *GPATCH2* | | | G patch domain containing 2 | | -2.04 | 0.00587 |
| 232183_at | *SERAC1* | | | serine active site containing 1 | | -2.04 | 0.00707 |
| 228121_at | *TGFB2* | | | transforming growth factor, beta 2 | | -2.04 | 0.00766 |
| 1555858_at | *SETD5-AS1* | | | SETD5 antisense RNA 1 | | -2.04 | 0.00772 |
| 222590_s_at | *NLK* | | | nemo-like kinase | | -2.04 | 0.0095 |
| 231544_s_at | *POLR3G* | | | polymerase (RNA) III (DNA directed) polypeptide G (32kD) | | -2.04 | 0.00962 |
| 218968_s_at | *ZFP64* | | | ZFP64 zinc finger protein | | -2.04 | 0.00985 |
| 203767_s_at | *STS* | | | steroid sulfatase (microsomal), isozyme S | | -2.04 | 0.01049 |
| 1558487_a_at | *TMED4* | | | transmembrane emp24 protein transport domain containing 4 | | -2.04 | 0.01121 |
| 244132_x_at | *ZNF518A* | | | zinc finger protein 518A | | -2.04 | 0.0128 |
| 201912_s_at | *GSPT1* | | | G1 to S phase transition 1 | | -2.04 | 0.01477 |
| 221487_s_at | *ENSA* | | | endosulfine alpha | | -2.04 | 0.01735 |
| 203640_at | *MBNL2* | | | muscleblind-like splicing regulator 2 | | -2.04 | 0.01771 |
| 1556151_at | *ITFG1* | | | integrin alpha FG-GAP repeat containing 1 | | -2.04 | 0.01778 |
| 218911_at | *YEATS4* | | | YEATS domain containing 4 | | -2.04 | 0.02295 |
| 224778_s_at | *TAOK1* | | | TAO kinase 1 | | -2.04 | 0.02421 |
| 228099_at | *ZNF550* | | | zinc finger protein 550 | | -2.04 | 0.02764 |
| 237910_x_at | *FAM92A1* | | | family with sequence similarity 92, member A1 | | -2.04 | 0.02869 |
| 218129_s_at | *NFYB* | | | nuclear transcription factor Y, beta | | -2.04 | 0.02913 |
| 228603_at | *ACTR3* | | | ARP3 actin-related protein 3 homolog (yeast) | | -2.04 | 0.03231 |
| 212302_at | *RTF1* | | | Rtf1, Paf1/RNA polymerase II complex component, homolog (S. cerevisiae) | | -2.04 | 0.033 |
| 233019_at | *CNOT7* | | | CCR4-NOT transcription complex, subunit 7 | | -2.04 | 0.03361 |
| 224648_at | *GPBP1* | | | GC-rich promoter binding protein 1 | | -2.04 | 0.03403 |
| 223641_at |  | | |  | | -2.04 | 0.04429 |
| 225459_at | *AMOTL1* | | | angiomotin like 1 | | -2.05 | 0.00017 |
| 225182_at | *TMEM50B* | | | transmembrane protein 50B | | -2.05 | 0.00026 |
| 204812_at | *ZW10* | | | zw10 kinetochore protein | | -2.05 | 0.00036 |
| 211968_s_at | *HSP90AA1* | | | heat shock protein 90kDa alpha (cytosolic), class A member 1 | | -2.05 | 0.00055 |
| 214090_at | *PAWR* | | | PRKC, apoptosis, WT1, regulator | | -2.05 | 0.00076 |
| 201632_at | *EIF2B1* | | | eukaryotic translation initiation factor 2B, subunit 1 alpha, 26kDa | | -2.05 | 0.00076 |
| 227270_at | *FAM200B* | | | family with sequence similarity 200, member B | | -2.05 | 0.00108 |
| 211071_s_at | *MLLT11* | | | myeloid/lymphoid or mixed-lineage leukemia (trithorax homolog, Drosophila); translocated to, 11 | | -2.05 | 0.00125 |
| 1556886_a_at | *LAYN* | | | layilin | | -2.05 | 0.00127 |
| 208081_s_at | *ZNF442* | | | zinc finger protein 442 | | -2.05 | 0.00154 |
| 202054_s_at | *ALDH3A2* | | | aldehyde dehydrogenase 3 family, member A2 | | -2.05 | 0.00182 |
| 223396_at | *TMEM60* | | | transmembrane protein 60 | | -2.05 | 0.00183 |
| 64488_at | *IRGQ* | | | immunity-related GTPase family, Q | | -2.05 | 0.0019 |
| 228144_at | *ZNF300* | | | zinc finger protein 300 | | -2.05 | 0.00228 |
| 1562234_a_at | *NAV3* | | | neuron navigator 3 | | -2.05 | 0.00246 |
| 227443_at | *LURAP1L* | | | leucine rich adaptor protein 1-like | | -2.05 | 0.00259 |
| 1553743_at | *METTL21A* | | | methyltransferase like 21A | | -2.05 | 0.00265 |
| 203610_s_at | *TRIM38* | | | tripartite motif containing 38 | | -2.05 | 0.00287 |
| 229793_at | *ASAH2B* | | | N-acylsphingosine amidohydrolase (non-lysosomal ceramidase) 2B | | -2.05 | 0.0029 |
| 1554906_a_at | *MPHOSPH6* | | | M-phase phosphoprotein 6 | | -2.05 | 0.00296 |
| 219783_at | *SLC35F6* | | | solute carrier family 35, member F6 | | -2.05 | 0.00301 |
| 202668_at | *EFNB2* | | | ephrin-B2 | | -2.05 | 0.00308 |
| 230516_at | *MALSU1* | | | mitochondrial assembly of ribosomal large subunit 1 | | -2.05 | 0.00327 |
| 238722_x_at | *NAPEPLD* | | | N-acyl phosphatidylethanolamine phospholipase D | | -2.05 | 0.00397 |
| 226665_at | *AHSA2* | | | AHA1, activator of heat shock 90kDa protein ATPase homolog 2 (yeast) | | -2.05 | 0.0041 |
| 1563189_at |  | | |  | | -2.05 | 0.00514 |
| 211761_s_at | *CACYBP* | | | calcyclin binding protein | | -2.05 | 0.00515 |
| 235000_at | *LPP* | | | LIM domain containing preferred translocation partner in lipoma | | -2.05 | 0.00573 |
| 228341_at | *NUDT16* | | | nudix (nucleoside diphosphate linked moiety X)-type motif 16 | | -2.05 | 0.00602 |
| 1555785_a_at | *XRN1* | | | 5'-3' exoribonuclease 1 | | -2.05 | 0.0063 |
| 212031_at | *RBM25* | | | RNA binding motif protein 25 | | -2.05 | 0.0064 |
| 243630_at | *NDUFB1* | | | NADH dehydrogenase (ubiquinone) 1 beta subcomplex, 1, 7kDa | | -2.05 | 0.00683 |
| 216607_s_at | *CYP51A1* | | | cytochrome P450, family 51, subfamily A, polypeptide 1; leucine-rich repeats and death domain containing 1 | | -2.05 | 0.00732 |
| 205191_at | *RP2* | | | retinitis pigmentosa 2 (X-linked recessive) | | -2.05 | 0.00741 |
| 225897_at | *MARCKS* | | | myristoylated alanine-rich protein kinase C substrate | | -2.05 | 0.00741 |
| 224935_at | *EIF2S3* | | | eukaryotic translation initiation factor 2, subunit 3 gamma, 52kDa | | -2.05 | 0.00762 |
| 212397_at | *RDX* | | | radixin | | -2.05 | 0.00765 |
| 242918_at | *NASP* | | | nuclear autoantigenic sperm protein (histone-binding) | | -2.05 | 0.0088 |
| 212815_at | *ASCC3* | | | activating signal cointegrator 1 complex subunit 3 | | -2.05 | 0.0104 |
| 222182_s_at | *CNOT2* | | | CCR4-NOT transcription complex, subunit 2 | | -2.05 | 0.0106 |
| 1556499_s_at | *COL1A1* | | | collagen, type I, alpha 1 | | -2.05 | 0.01092 |
| 201432_at | *CAT* | | | catalase | | -2.05 | 0.0112 |
| 200690_at | *HSPA9* | | | heat shock 70kDa protein 9 (mortalin) | | -2.05 | 0.01154 |
| 222441_x_at | *SLMO2* | | | slowmo homolog 2 (Drosophila) | | -2.05 | 0.01172 |
| 212409_s_at | *TOR1AIP1* | | | torsin A interacting protein 1 | | -2.05 | 0.01352 |
| 1554411_at | *CTNNB1* | | | catenin (cadherin-associated protein), beta 1, 88kDa | | -2.05 | 0.01387 |
| 223616_at | *ZNF649* | | | zinc finger protein 649 | | -2.05 | 0.01488 |
| 202678_at | *GTF2A2* | | | general transcription factor IIA, 2, 12kDa | | -2.05 | 0.01554 |
| 221517_s_at | *MED17* | | | mediator complex subunit 17 | | -2.05 | 0.01615 |
| 213158_at |  | | |  | | -2.05 | 0.0165 |
| 202097_at | *NUP153* | | | nucleoporin 153kDa | | -2.05 | 0.0179 |
| 205761_s_at | *DUS4L* | | | dihydrouridine synthase 4-like (S. cerevisiae) | | -2.05 | 0.01849 |
| 236932_s_at | *GATAD2A* | | | GATA zinc finger domain containing 2A | | -2.05 | 0.0189 |
| 218469_at | *GREM1* | | | gremlin 1, DAN family BMP antagonist | | -2.05 | 0.01937 |
| 217881_s_at | *CDC27* | | | cell division cycle 27 | | -2.05 | 0.02099 |
| 206734_at | *JRKL* | | | jerky homolog-like (mouse) | | -2.05 | 0.02282 |
| 238469_at | *OGFRL1* | | | opioid growth factor receptor-like 1 | | -2.05 | 0.02314 |
| 227357_at | *TAB3* | | | TGF-beta activated kinase 1/MAP3K7 binding protein 3 | | -2.05 | 0.02864 |
| 204782_at |  | | |  | | -2.05 | 0.0299 |
| 204658_at | *TRA2A* | | | transformer 2 alpha homolog (Drosophila) | | -2.05 | 0.03109 |
| 239252_at | *COX7B* | | | cytochrome c oxidase subunit VIIb | | -2.05 | 0.0318 |
| 206581_at | *BNC1* | | | basonuclin 1 | | -2.05 | 0.03625 |
| 212530_at | *NEK7* | | | NIMA-related kinase 7 | | -2.05 | 0.0387 |
| 215450_at | *SNRPE* | | | small nuclear ribonucleoprotein polypeptide E | | -2.06 | 0.00015 |
| 230426_at | *DLD* | | | dihydrolipoamide dehydrogenase | | -2.06 | 0.0003 |
| 221542_s_at | *ERLIN2* | | | ER lipid raft associated 2 | | -2.06 | 0.0003 |
| 214211_at | *FTH1* | | | ferritin, heavy polypeptide 1 | | -2.06 | 0.00033 |
| 213813_x_at |  | | |  | | -2.06 | 0.00037 |
| 224634_at | *GPATCH4* | | | G patch domain containing 4 | | -2.06 | 0.00069 |
| 203060_s_at | *PAPSS2* | | | 3'-phosphoadenosine 5'-phosphosulfate synthase 2 | | -2.06 | 0.0015 |
| 221625_at |  | | |  | | -2.06 | 0.00192 |
| 204105_s_at | *NRCAM* | | | neuronal cell adhesion molecule | | -2.06 | 0.00194 |
| 213881_x_at | *SUMO2* | | | small ubiquitin-like modifier 2 | | -2.06 | 0.00247 |
| 201574_at | *ETF1* | | | eukaryotic translation termination factor 1 | | -2.06 | 0.00316 |
| 227227_at |  | | |  | | -2.06 | 0.0045 |
| 212477_at | *ACAP2* | | | ArfGAP with coiled-coil, ankyrin repeat and PH domains 2 | | -2.06 | 0.00494 |
| 217880_at | *CDC27* | | | cell division cycle 27 | | -2.06 | 0.00495 |
| 209434_s_at | *PPAT* | | | phosphoribosyl pyrophosphate amidotransferase | | -2.06 | 0.00655 |
| 230192_at | *TRIM13* | | | tripartite motif containing 13 | | -2.06 | 0.00665 |
| 204980_at | *CLOCK* | | | clock circadian regulator | | -2.06 | 0.0076 |
| 201238_s_at | *CAPZA2* | | | capping protein (actin filament) muscle Z-line, alpha 2 | | -2.06 | 0.00809 |
| 201370_s_at | *CUL3* | | | cullin 3 | | -2.06 | 0.00884 |
| 202964_s_at | *RFX5* | | | regulatory factor X, 5 (influences HLA class II expression) | | -2.06 | 0.0092 |
| 213291_s_at | *UBE3A* | | | ubiquitin protein ligase E3A | | -2.06 | 0.00936 |
| 218515_at | *PAXBP1* | | | PAX3 and PAX7 binding protein 1 | | -2.06 | 0.01029 |
| 207280_at | *RNF185-AS1* | | | RNF185 antisense RNA 1 | | -2.06 | 0.01135 |
| 236002_at |  | | |  | | -2.06 | 0.01204 |
| 240544_at |  | | |  | | -2.06 | 0.01406 |
| 213241_at | *PLXNC1* | | | plexin C1 | | -2.06 | 0.01432 |
| 212984_at | *ATF2* | | | activating transcription factor 2 | | -2.06 | 0.01466 |
| 212216_at | *PREPL* | | | prolyl endopeptidase-like | | -2.06 | 0.01635 |
| 209022_at | *STAG2* | | | stromal antigen 2 | | -2.06 | 0.01665 |
| 212193_s_at | *LARP1* | | | La ribonucleoprotein domain family, member 1 | | -2.06 | 0.01722 |
| 218544_s_at | *RCL1* | | | RNA terminal phosphate cyclase-like 1 | | -2.06 | 0.01794 |
| 201706_s_at | *PEX19* | | | peroxisomal biogenesis factor 19 | | -2.06 | 0.01841 |
| 231841_s_at | *KIAA1462* | | | KIAA1462 | | -2.06 | 0.01853 |
| 206059_at | *ZNF91* | | | zinc finger protein 91 | | -2.06 | 0.01967 |
| 200004_at | *EIF4G2* | | | eukaryotic translation initiation factor 4 gamma, 2 | | -2.06 | 0.02096 |
| 234947_s_at | *FAM204A* | | | family with sequence similarity 204, member A | | -2.06 | 0.03023 |
| 237994_at |  | | |  | | -2.06 | 0.03104 |
| 205121_at | *SGCB* | | | sarcoglycan, beta (43kDa dystrophin-associated glycoprotein) | | -2.06 | 0.03306 |
| 202611_s_at | *MED14* | | | mediator complex subunit 14 | | -2.06 | 0.0336 |
| 1569490_at | *FNDC3B* | | | fibronectin type III domain containing 3B | | -2.06 | 0.03786 |
| 238119_at |  | | |  | | -2.06 | 0.04374 |
| 238480_at | *TTC39C* | | | tetratricopeptide repeat domain 39C | | -2.07 | 2.2x10^-5^ |
| 238829_at |  | | |  | | -2.07 | 0.00036 |
| 230655_at |  | | |  | | -2.07 | 0.00043 |
| 228135_at | *C1orf52* | | | chromosome 1 open reading frame 52 | | -2.07 | 0.00074 |
| 215093_at | *NSDHL* | | | NAD(P) dependent steroid dehydrogenase-like | | -2.07 | 0.0014 |
| 201132_at | *HNRNPH2* | | | heterogeneous nuclear ribonucleoprotein H2 (H'); RPL36A-HNRNPH2 readthrough | | -2.07 | 0.00158 |
| 218796_at | *FERMT1* | | | fermitin family member 1 | | -2.07 | 0.00209 |
| 211999_at | *H3F3A* | | | H3 histone, family 3A; H3 histone, family 3B (H3.3B); microRNA 4738 | | -2.07 | 0.0023 |
| 226611_s_at | *CENPV* | | | centromere protein V | | -2.07 | 0.00236 |
| 242277_at |  | | |  | | -2.07 | 0.00252 |
| 201143_s_at | *EIF2S1* | | | eukaryotic translation initiation factor 2, subunit 1 alpha, 35kDa | | -2.07 | 0.00313 |
| 214077_x_at | *MEIS3P1* | | | Meis homeobox 3 pseudogene 1 | | -2.07 | 0.00371 |
| 227451_s_at | *MCUR1* | | | mitochondrial calcium uniporter regulator 1 | | -2.07 | 0.00383 |
| 203521_s_at | *ZNF318* | | | zinc finger protein 318 | | -2.07 | 0.00405 |
| 217809_at | *BZW2* | | | basic leucine zipper and W2 domains 2 | | -2.07 | 0.00418 |
| 226180_at | *WDR36* | | | WD repeat domain 36 | | -2.07 | 0.00437 |
| 218514_at | *SMG8* | | | smg-8 homolog, nonsense mediated mRNA decay factor (C. elegans) | | -2.07 | 0.00442 |
| 223250_at | *KLHL7* | | | kelch-like family member 7 | | -2.07 | 0.00558 |
| 213447_at | *IPW* | | | imprinted in Prader-Willi syndrome (non-protein coding) | | -2.07 | 0.00567 |
| 225186_at | *RAPH1* | | | Ras association (RalGDS/AF-6) and pleckstrin homology domains 1 | | -2.07 | 0.00589 |
| 222688_at | *ACER3* | | | alkaline ceramidase 3 | | -2.07 | 0.00663 |
| 215787_at | *ACTA2* | | | actin, alpha 2, smooth muscle, aorta | | -2.07 | 0.0072 |
| 214773_x_at | *TIPRL* | | | TIP41, TOR signaling pathway regulator-like (S. cerevisiae) | | -2.07 | 0.00888 |
| 218171_at | *VPS4B* | | | vacuolar protein sorting 4 homolog B (S. cerevisiae) | | -2.07 | 0.00914 |
| 214453_s_at | *IFI44* | | | interferon-induced protein 44 | | -2.07 | 0.01067 |
| 1570156_s_at | *FMN1* | | | formin 1 | | -2.07 | 0.01141 |
| 204391_x_at | *TRIM24* | | | tripartite motif containing 24 | | -2.07 | 0.01203 |
| 240066_at |  | | |  | | -2.07 | 0.01214 |
| 209323_at | *PRKRIR* | | | protein-kinase, interferon-inducible double stranded RNA dependent inhibitor, repressor of (P58 repressor) | | -2.07 | 0.0135 |
| 243071_at |  | | |  | | -2.07 | 0.01451 |
| 217106_x_at | *DIMT1* | | | DIM1 dimethyladenosine transferase 1 homolog (S. cerevisiae) | | -2.07 | 0.01553 |
| 227261_at | *KLF12* | | | Kruppel-like factor 12 | | -2.07 | 0.01874 |
| 233341_s_at | *POLR1B* | | | polymerase (RNA) I polypeptide B, 128kDa | | -2.07 | 0.01941 |
| 205739_x_at | *ZNF107* | | | zinc finger protein 107 | | -2.07 | 0.02038 |
| 212160_at | *XPOT* | | | exportin, tRNA | | -2.07 | 0.02273 |
| 201776_s_at | *EFCAB14* | | | EF-hand calcium binding domain 14 | | -2.07 | 0.02564 |
| 203705_s_at | *FZD7* | | | frizzled family receptor 7 | | -2.07 | 0.0268 |
| 242007_at |  | | |  | | -2.07 | 0.02731 |
| 227523_s_at | *PHF20L1* | | | PHD finger protein 20-like 1 | | -2.07 | 0.02888 |
| 219166_at | *DNAAF2* | | | dynein, axonemal, assembly factor 2 | | -2.07 | 0.02971 |
| 202171_at | *VEZF1* | | | vascular endothelial zinc finger 1 | | -2.07 | 0.03086 |
| 228941_at | *ALG10B* | | | ALG10B, alpha-1,2-glucosyltransferase | | -2.07 | 0.03117 |
| 219306_at | *KIF15* | | | kinesin family member 15 | | -2.07 | 0.03156 |
| 1555960_at | *HINT1* | | | histidine triad nucleotide binding protein 1 | | -2.07 | 0.0384 |
| 228916_at | *CWF19L2* | | | CWF19-like 2, cell cycle control (S. pombe) | | -2.07 | 0.03946 |
| 1552426_a_at | *TM2D3* | | | TM2 domain containing 3 | | -2.07 | 0.0488 |
| 209419_at | *THOC5* | | | THO complex 5 | | -2.07 | 0.04887 |
| 202269_x_at | *GBP1* | | | guanylate binding protein 1, interferon-inducible | | -2.08 | 0.00056 |
| 227244_s_at |  | | |  | | -2.08 | 0.00059 |
| 202850_at | *ABCD3* | | | ATP-binding cassette, sub-family D (ALD), member 3 | | -2.08 | 0.00101 |
| 1553570_x_at | *COX2* | | | cytochrome c oxidase subunit II; OAF homolog (Drosophila); transducin-like enhancer of split 1 (E(sp1) homolog, Drosophila) | | -2.08 | 0.00138 |
| 238056_at | *SDHC* | | | succinate dehydrogenase complex, subunit C, integral membrane protein, 15kDa | | -2.08 | 0.00176 |
| 212510_at | *GPD1L* | | | glycerol-3-phosphate dehydrogenase 1-like | | -2.08 | 0.00241 |
| 226498_at | *FLT1* | | | fms-related tyrosine kinase 1 | | -2.08 | 0.0031 |
| 217508_s_at | *C18orf25* | | | chromosome 18 open reading frame 25 | | -2.08 | 0.00321 |
| 220939_s_at | *DPP8* | | | dipeptidyl-peptidase 8 | | -2.08 | 0.00331 |
| 221952_x_at | *TRMT5* | | | tRNA methyltransferase 5 homolog (S. cerevisiae) | | -2.08 | 0.00356 |
| 221613_s_at | *ZFAND6* | | | zinc finger, AN1-type domain 6 | | -2.08 | 0.00452 |
| 226832_at | *RNF168* | | | ring finger protein 168, E3 ubiquitin protein ligase | | -2.08 | 0.00492 |
| 239537_at | *ST8SIA2* | | | ST8 alpha-N-acetyl-neuraminide alpha-2,8-sialyltransferase 2 | | -2.08 | 0.00525 |
| 218306_s_at | *HERC1* | | | HECT and RLD domain containing E3 ubiquitin protein ligase family member 1 | | -2.08 | 0.00674 |
| 201612_at | *ALDH9A1* | | | aldehyde dehydrogenase 9 family, member A1 | | -2.08 | 0.007 |
| 1557985_s_at | *CEP78* | | | centrosomal protein 78kDa | | -2.08 | 0.00837 |
| 1562738_a_at | *USP3-AS1* | | | USP3 antisense RNA 1 | | -2.08 | 0.00923 |
| 219347_at | *NUDT15* | | | nudix (nucleoside diphosphate linked moiety X)-type motif 15 | | -2.08 | 0.00995 |
| 40189_at | *SET* | | | SET nuclear oncogene | | -2.08 | 0.01001 |
| 222586_s_at | *OSBPL11* | | | oxysterol binding protein-like 11 | | -2.08 | 0.01139 |
| 227554_at | *MAGI2-AS3* | | | MAGI2 antisense RNA 3 | | -2.08 | 0.01141 |
| 218304_s_at | *OSBPL11* | | | oxysterol binding protein-like 11 | | -2.08 | 0.01143 |
| 1555929_s_at |  | | |  | | -2.08 | 0.01234 |
| 1552306_at | *ALG10* | | | ALG10, alpha-1,2-glucosyltransferase | | -2.08 | 0.01283 |
| 235766_x_at | *RAB27A* | | | RAB27A, member RAS oncogene family | | -2.08 | 0.01316 |
| 232489_at | *TRMT13* | | | tRNA methyltransferase 13 homolog (S. cerevisiae) | | -2.08 | 0.01476 |
| 218610_s_at | *CPPED1* | | | calcineurin-like phosphoesterase domain containing 1 | | -2.08 | 0.01613 |
| 1558292_s_at | *PIGW* | | | phosphatidylinositol glycan anchor biosynthesis, class W | | -2.08 | 0.01746 |
| 234987_at | *SAMHD1* | | | SAM domain and HD domain 1 | | -2.08 | 0.01844 |
| 1553694_a_at | *PIK3C2A* | | | phosphatidylinositol-4-phosphate 3-kinase, catalytic subunit type 2 alpha | | -2.08 | 0.02037 |
| 1558254_s_at | *SRPK2* | | | SRSF protein kinase 2 | | -2.08 | 0.02047 |
| 1552790_a_at | *SEC62* | | | SEC62 homolog (S. cerevisiae) | | -2.08 | 0.02233 |
| 238990_x_at | *TRIM61* | | | tripartite motif containing 61 | | -2.08 | 0.02708 |
| 212533_at | *WEE1* | | | WEE1 homolog (S. pombe) | | -2.08 | 0.0273 |
| 1554726_at | *ZNF655* | | | zinc finger protein 655 | | -2.08 | 0.0289 |
| 226225_at | *MCC* | | | mutated in colorectal cancers | | -2.08 | 0.03005 |
| 218461_at | *GPN3* | | | GPN-loop GTPase 3 | | -2.08 | 0.03397 |
| 203970_s_at | *PEX3* | | | peroxisomal biogenesis factor 3 | | -2.08 | 0.03818 |
| 202971_s_at | *DYRK2* | | | dual-specificity tyrosine-(Y)-phosphorylation regulated kinase 2 | | -2.08 | 0.04377 |
| 221634_at | *MGC2752* | | | CENPB DNA-binding domains containing 1 pseudogene; ribosomal protein L23a pseudogene 7 | | -2.08 | 0.04574 |
| 223113_at | *TMEM138* | | | transmembrane protein 138 | | -2.09 | 0.00019 |
| 226385_s_at | *MALSU1* | | | mitochondrial assembly of ribosomal large subunit 1 | | -2.09 | 0.00043 |
| 229144_at | *KAZN* | | | kazrin, periplakin interacting protein | | -2.09 | 0.00058 |
| 227658_s_at | *PLEKHA3* | | | pleckstrin homology domain containing, family A (phosphoinositide binding specific) member 3 | | -2.09 | 0.00071 |
| 219083_at | *SHQ1* | | | SHQ1, H/ACA ribonucleoprotein assembly factor | | -2.09 | 0.00072 |
| 230047_at | *ARHGAP42* | | | Rho GTPase activating protein 42 | | -2.09 | 0.00111 |
| 203598_s_at | *WBP4* | | | WW domain binding protein 4 | | -2.09 | 0.00243 |
| 224823_at | *MYLK* | | | myosin light chain kinase | | -2.09 | 0.00245 |
| 226509_at | *ZNF641* | | | zinc finger protein 641 | | -2.09 | 0.00294 |
| 227445_at | *ZNF689* | | | zinc finger protein 689 | | -2.09 | 0.00311 |
| 218595_s_at | *HEATR1* | | | HEAT repeat containing 1 | | -2.09 | 0.00517 |
| 237919_at | *RFFL* | | | ring finger and FYVE-like domain containing E3 ubiquitin protein ligase | | -2.09 | 0.00568 |
| 213425_at | *WNT5A* | | | wingless-type MMTV integration site family, member 5A | | -2.09 | 0.0062 |
| 243583_at |  | | |  | | -2.09 | 0.0065 |
| 210573_s_at |  | | |  | | -2.09 | 0.00673 |
| 225202_at | *RHOBTB3* | | | Rho-related BTB domain containing 3 | | -2.09 | 0.00776 |
| 243790_at | *ZNF585A* | | | zinc finger protein 585A | | -2.09 | 0.00803 |
| 212725_s_at | *TUG1* | | | taurine upregulated 1 (non-protein coding) | | -2.09 | 0.00911 |
| 222369_at | *NAA40* | | | N(alpha)-acetyltransferase 40, NatD catalytic subunit, homolog (S. cerevisiae) | | -2.09 | 0.00913 |
| 202506_at | *SSFA2* | | | sperm specific antigen 2 | | -2.09 | 0.00924 |
| 236207_at | *SSFA2* | | | sperm specific antigen 2 | | -2.09 | 0.00978 |
| 1556210_at |  | | |  | | -2.09 | 0.00985 |
| 205229_s_at | *COCH* | | | cochlin | | -2.09 | 0.00995 |
| 222824_at | *NUDT5* | | | nudix (nucleoside diphosphate linked moiety X)-type motif 5 | | -2.09 | 0.0103 |
| 214717_at | *PKI55* | | | DKFZp434H1419 | | -2.09 | 0.01056 |
| 212589_at | *RRAS2* | | | related RAS viral (r-ras) oncogene homolog 2 | | -2.09 | 0.01333 |
| 222444_at | *ARMCX3* | | | armadillo repeat containing, X-linked 3 | | -2.09 | 0.01515 |
| 212590_at | *RRAS2* | | | related RAS viral (r-ras) oncogene homolog 2 | | -2.09 | 0.01611 |
| 213754_s_at | *PAIP1* | | | poly(A) binding protein interacting protein 1 | | -2.09 | 0.01733 |
| 213251_at | *SMARCA5* | | | SWI/SNF related, matrix associated, actin dependent regulator of chromatin, subfamily a, member 5 | | -2.09 | 0.01844 |
| 226528_at | *MTX3* | | | metaxin 3 | | -2.09 | 0.01963 |
| 213598_at |  | | |  | | -2.09 | 0.02235 |
| 222393_s_at | *NAA50* | | | N(alpha)-acetyltransferase 50, NatE catalytic subunit | | -2.09 | 0.02398 |
| 219156_at | *SYNJ2BP* | | | synaptojanin 2 binding protein; SYNJ2BP-COX16 readthrough | | -2.09 | 0.02465 |
| 228046_at | *ZNF827* | | | zinc finger protein 827 | | -2.09 | 0.02517 |
| 208809_s_at | *C6orf62* | | | chromosome 6 open reading frame 62 | | -2.09 | 0.02641 |
| 1552307_a_at | *TTC39C* | | | tetratricopeptide repeat domain 39C | | -2.09 | 0.03334 |
| 222580_at |  | | |  | | -2.09 | 0.03713 |
| 222681_at | *POGLUT1* | | | protein O-glucosyltransferase 1 | | -2.09 | 0.03772 |
| 232044_at | *RBBP6* | | | retinoblastoma binding protein 6 | | -2.09 | 0.04491 |
| 239561_at |  | | |  | | -2.09 | 0.04566 |
| 202184_s_at | *NUP133* | | | nucleoporin 133kDa | | -2.1 | 0.00029 |
| 217601_at | *NUP188* | | | nucleoporin 188kDa | | -2.1 | 0.00042 |
| 216304_x_at | *YME1L1* | | | YME1-like 1 ATPase | | -2.1 | 0.00094 |
| 225888_at | *NAA25* | | | N(alpha)-acetyltransferase 25, NatB auxiliary subunit | | -2.1 | 0.00095 |
| 204780_s_at | *FAS* | | | Fas cell surface death receptor | | -2.1 | 0.00118 |
| 232771_at | *NRK* | | | Nik related kinase | | -2.1 | 0.00198 |
| 229422_at | *NRD1* | | | nardilysin (N-arginine dibasic convertase) | | -2.1 | 0.00267 |
| 226740_x_at |  | | |  | | -2.1 | 0.00273 |
| 225068_at | *KLHL12* | | | kelch-like family member 12 | | -2.1 | 0.0029 |
| 235711_at | *PURB* | | | purine-rich element binding protein B | | -2.1 | 0.00297 |
| 46167_at |  | | |  | | -2.1 | 0.0031 |
| 228161_at | *RAB32* | | | RAB32, member RAS oncogene family | | -2.1 | 0.00343 |
| 239678_at |  | | |  | | -2.1 | 0.00397 |
| 209248_at | *GHITM* | | | growth hormone inducible transmembrane protein | | -2.1 | 0.00427 |
| 226508_at | *PHC3* | | | polyhomeotic homolog 3 (Drosophila) | | -2.1 | 0.00466 |
| 222411_s_at | *SSR3* | | | signal sequence receptor, gamma (translocon-associated protein gamma) | | -2.1 | 0.00472 |
| 205214_at | *STK17B* | | | serine/threonine kinase 17b | | -2.1 | 0.0049 |
| 229865_at | *FNDC3B* | | | fibronectin type III domain containing 3B | | -2.1 | 0.00521 |
| 201842_s_at | *EFEMP1* | | | EGF containing fibulin-like extracellular matrix protein 1 | | -2.1 | 0.00726 |
| 209647_s_at | *SOCS5* | | | suppressor of cytokine signaling 5 | | -2.1 | 0.00734 |
| 218395_at | *ACTR6* | | | ARP6 actin-related protein 6 homolog (yeast) | | -2.1 | 0.00779 |
| 220250_at | *ZNF286A* | | | zinc finger protein 286A | | -2.1 | 0.00794 |
| 225045_at | *CCDC88A* | | | coiled-coil domain containing 88A | | -2.1 | 0.00798 |
| 230542_at | *ZNF597* | | | zinc finger protein 597 | | -2.1 | 0.00894 |
| 232566_at | *NOL6* | | | nucleolar protein 6 (RNA-associated) | | -2.1 | 0.00929 |
| 218748_s_at | *EXOC5* | | | exocyst complex component 5 | | -2.1 | 0.01056 |
| 209033_s_at | *DYRK1A* | | | dual-specificity tyrosine-(Y)-phosphorylation regulated kinase 1A | | -2.1 | 0.01068 |
| 219960_s_at | *UCHL5* | | | ubiquitin carboxyl-terminal hydrolase L5 | | -2.1 | 0.01104 |
| 226254_s_at | *KIAA1430* | | | KIAA1430 | | -2.1 | 0.01127 |
| 202660_at | *ITPR2* | | | inositol 1,4,5-trisphosphate receptor, type 2 | | -2.1 | 0.01564 |
| 216004_s_at | *PKNOX1* | | | PBX/knotted 1 homeobox 1 | | -2.1 | 0.01588 |
| 1556126_s_at |  | | |  | | -2.1 | 0.01618 |
| 227928_at | *PARPBP* | | | PARP1 binding protein | | -2.1 | 0.01673 |
| 203988_s_at | *FUT8* | | | fucosyltransferase 8 (alpha (1,6) fucosyltransferase) | | -2.1 | 0.01742 |
| 205393_s_at | *CHEK1* | | | checkpoint kinase 1 | | -2.1 | 0.01867 |
| 224373_s_at | *C10orf99* | | | chromosome 10 open reading frame 99; coiled-coil domain containing 104; heterogeneous nuclear ribonucleoprotein M; NADH dehydrogenase, subunit 4 (complex I) | | -2.1 | 0.02049 |
| 204024_at | *OSGIN2* | | | oxidative stress induced growth inhibitor family member 2 | | -2.1 | 0.02067 |
| 201017_at | *EIF1AX* | | | eukaryotic translation initiation factor 1A, X-linked; eukaryotic translation initiation factor 1A, X-chromosomal-like | | -2.1 | 0.02176 |
| 224655_at | *AK3* | | | adenylate kinase 3 | | -2.1 | 0.0241 |
| 238795_at | *FAM208B* | | | family with sequence similarity 208, member B | | -2.1 | 0.02492 |
| 229072_at | *RAB30* | | | RAB30, member RAS oncogene family | | -2.1 | 0.02594 |
| 228339_at | *ECSCR* | | | endothelial cell surface expressed chemotaxis and apoptosis regulator | | -2.1 | 0.04134 |
| 201860_s_at | *PLAT* | | | plasminogen activator, tissue | | -2.11 | 0.00022 |
| 225484_at | *CEP41* | | | centrosomal protein 41kDa | | -2.11 | 0.00155 |
| 1567219_at |  | | |  | | -2.11 | 0.00166 |
| 224714_at | *MKI67IP* | | | MKI67 (FHA domain) interacting nucleolar phosphoprotein | | -2.11 | 0.00178 |
| 218287_s_at | *AGO1* | | | argonaute RISC catalytic component 1 | | -2.11 | 0.00186 |
| 204531_s_at | *BRCA1* | | | breast cancer 1, early onset | | -2.11 | 0.0023 |
| 204285_s_at | *PMAIP1* | | | phorbol-12-myristate-13-acetate-induced protein 1 | | -2.11 | 0.00242 |
| 200783_s_at | *STMN1* | | | stathmin 1 | | -2.11 | 0.00243 |
| 201722_s_at | *GALNT1* | | | UDP-N-acetyl-alpha-D-galactosamine:polypeptide N-acetylgalactosaminyltransferase 1 (GalNAc-T1) | | -2.11 | 0.00339 |
| 1559449_a_at |  | | |  | | -2.11 | 0.00345 |
| 244552_at | *ZNF788* | | | zinc finger family member 788 | | -2.11 | 0.00366 |
| 213243_at | *VPS13B* | | | vacuolar protein sorting 13 homolog B (yeast) | | -2.11 | 0.00379 |
| 228825_at | *PTGR1* | | | prostaglandin reductase 1 | | -2.11 | 0.00459 |
| 221520_s_at | *CDCA8* | | | cell division cycle associated 8 | | -2.11 | 0.00477 |
| 239106_at | *CA5BP1* | | | carbonic anhydrase VB pseudogene 1 | | -2.11 | 0.00614 |
| 203566_s_at | *AGL* | | | amylo-alpha-1, 6-glucosidase, 4-alpha-glucanotransferase | | -2.11 | 0.00663 |
| 209624_s_at | *MCCC2* | | | methylcrotonoyl-CoA carboxylase 2 (beta) | | -2.11 | 0.00669 |
| 1555384_a_at | *LARP4* | | | La ribonucleoprotein domain family, member 4 | | -2.11 | 0.00772 |
| 209257_s_at | *SMC3* | | | structural maintenance of chromosomes 3 | | -2.11 | 0.00773 |
| 204866_at | *PHF16* | | | PHD finger protein 16 | | -2.11 | 0.0081 |
| 206942_s_at | *PMCH* | | | pro-melanin-concentrating hormone | | -2.11 | 0.00834 |
| 219099_at | *C12orf5* | | | chromosome 12 open reading frame 5 | | -2.11 | 0.00835 |
| 201218_at | *CTBP2* | | | C-terminal binding protein 2 | | -2.11 | 0.00869 |
| 205857_at | *SLC18A2* | | | solute carrier family 18 (vesicular monoamine), member 2 | | -2.11 | 0.00886 |
| 217758_s_at | *TM9SF3* | | | transmembrane 9 superfamily member 3 | | -2.11 | 0.00923 |
| 203739_at | *ZNF217* | | | zinc finger protein 217 | | -2.11 | 0.0094 |
| 228988_at | *ZNF711* | | | zinc finger protein 711 | | -2.11 | 0.01074 |
| 215570_s_at | *ZNF780A* | | | zinc finger protein 780A; zinc finger protein 780B | | -2.11 | 0.01089 |
| 225773_at | *RSPRY1* | | | ring finger and SPRY domain containing 1 | | -2.11 | 0.01115 |
| 226190_at | *MAP3K13* | | | mitogen-activated protein kinase kinase kinase 13 | | -2.11 | 0.01205 |
| 212322_at | *SGPL1* | | | sphingosine-1-phosphate lyase 1 | | -2.11 | 0.01246 |
| 219582_at | *OGFRL1* | | | opioid growth factor receptor-like 1 | | -2.11 | 0.0128 |
| 229043_at | *PAPD5* | | | PAP associated domain containing 5 | | -2.11 | 0.01294 |
| 212888_at | *DICER1* | | | dicer 1, ribonuclease type III | | -2.11 | 0.0134 |
| 200788_s_at | *PEA15* | | | phosphoprotein enriched in astrocytes 15 | | -2.11 | 0.01342 |
| 200776_s_at | *BZW1* | | | basic leucine zipper and W2 domains 1 | | -2.11 | 0.01473 |
| 1553994_at | *NT5E* | | | 5'-nucleotidase, ecto (CD73) | | -2.11 | 0.01474 |
| 214359_s_at | *HSP90AB1* | | | heat shock protein 90kDa alpha (cytosolic), class B member 1 | | -2.11 | 0.01656 |
| 214800_x_at | *BTF3* | | | basic transcription factor 3 | | -2.11 | 0.01694 |
| 226802_s_at | *LOC96610* | | | BMS1 homolog, ribosome assembly protein (yeast) pseudogene | | -2.11 | 0.01907 |
| 209274_s_at | *ISCA1* | | | iron-sulfur cluster assembly 1 homolog (S. cerevisiae) | | -2.11 | 0.02205 |
| 1555358_a_at | *ENTPD4* | | | ectonucleoside triphosphate diphosphohydrolase 4 | | -2.11 | 0.02763 |
| 212982_at | *ZDHHC17* | | | zinc finger, DHHC-type containing 17 | | -2.11 | 0.02943 |
| 224953_at | *YIPF5* | | | Yip1 domain family, member 5 | | -2.11 | 0.03336 |
| 242372_s_at | *MFSD4* | | | major facilitator superfamily domain containing 4 | | -2.11 | 0.03929 |
| 209404_s_at | *TMED7* | | | transmembrane emp24 protein transport domain containing 7; TMED7-TICAM2 readthrough | | -2.11 | 0.0437 |
| 205280_at | *GLRB* | | | glycine receptor, beta | | -2.11 | 0.0452 |
| 218967_s_at | *PTER* | | | phosphotriesterase related | | -2.11 | 0.04669 |
| 239526_x_at | *PTPN1* | | | protein tyrosine phosphatase, non-receptor type 1 | | -2.12 | 5.2x10^-5^ |
| 218442_at | *TTC4* | | | tetratricopeptide repeat domain 4 | | -2.12 | 0.00024 |
| 219654_at | *PTPLA* | | | protein tyrosine phosphatase-like (proline instead of catalytic arginine), member A | | -2.12 | 0.00041 |
| 201713_s_at | *RANBP2* | | | RAN binding protein 2 | | -2.12 | 0.00069 |
| 1554089_s_at | *SBDS* | | | Shwachman-Bodian-Diamond syndrome; Shwachman-Bodian-Diamond syndrome pseudogene 1 | | -2.12 | 0.00114 |
| 203487_s_at | *ARMC8* | | | armadillo repeat containing 8 | | -2.12 | 0.00118 |
| 223096_at | *NOP58* | | | NOP58 ribonucleoprotein | | -2.12 | 0.00126 |
| 41553_at | *OSGIN2* | | | oxidative stress induced growth inhibitor family member 2 | | -2.12 | 0.00157 |
| 231785_at | *NTF4* | | | neurotrophin 4 | | -2.12 | 0.00169 |
| 204835_at | *POLA1* | | | polymerase (DNA directed), alpha 1, catalytic subunit | | -2.12 | 0.00186 |
| 1565746_at |  | | |  | | -2.12 | 0.00188 |
| 230241_at | *TOR1AIP2* | | | torsin A interacting protein 2 | | -2.12 | 0.00246 |
| 225291_at | *PNPT1* | | | polyribonucleotide nucleotidyltransferase 1 | | -2.12 | 0.00247 |
| 224694_at | *ANTXR1* | | | anthrax toxin receptor 1 | | -2.12 | 0.00274 |
| 232165_at | *EPPK1* | | | epiplakin 1 | | -2.12 | 0.00274 |
| 209852_x_at | *PSME3* | | | proteasome (prosome, macropain) activator subunit 3 (PA28 gamma; Ki) | | -2.12 | 0.00279 |
| 241453_at | *PTK2* | | | protein tyrosine kinase 2 | | -2.12 | 0.00288 |
| 212202_s_at | *TMEM87A* | | | transmembrane protein 87A | | -2.12 | 0.00312 |
| 224410_s_at | *LMBR1* | | | limb region 1 homolog (mouse) | | -2.12 | 0.00379 |
| 209226_s_at | *TNPO1* | | | transportin 1 | | -2.12 | 0.00396 |
| 203706_s_at | *FZD7* | | | frizzled family receptor 7 | | -2.12 | 0.00662 |
| 220238_s_at | *KLHL7* | | | kelch-like family member 7 | | -2.12 | 0.00731 |
| 212112_s_at | *STX12* | | | syntaxin 12 | | -2.12 | 0.0075 |
| 214591_at | *KLHL4* | | | kelch-like family member 4 | | -2.12 | 0.0076 |
| 225093_at | *UTRN* | | | utrophin | | -2.12 | 0.00796 |
| 218196_at | *OSTM1* | | | osteopetrosis associated transmembrane protein 1 | | -2.12 | 0.00886 |
| 203651_at | *ZFYVE16* | | | zinc finger, FYVE domain containing 16 | | -2.12 | 0.00886 |
| 209341_s_at | *IKBKB* | | | inhibitor of kappa light polypeptide gene enhancer in B-cells, kinase beta | | -2.12 | 0.00992 |
| 202922_at | *GCLC* | | | glutamate-cysteine ligase, catalytic subunit | | -2.12 | 0.01016 |
| 225617_at | *ODF2* | | | outer dense fiber of sperm tails 2 | | -2.12 | 0.01053 |
| 1558759_s_at | *CLASP2* | | | cytoplasmic linker associated protein 2 | | -2.12 | 0.0131 |
| 1556082_a_at |  | | |  | | -2.12 | 0.01718 |
| 241827_at | *ZNF615* | | | zinc finger protein 615 | | -2.12 | 0.02055 |
| 218696_at | *EIF2AK3* | | | eukaryotic translation initiation factor 2-alpha kinase 3 | | -2.12 | 0.02266 |
| 214863_at |  | | |  | | -2.12 | 0.02368 |
| 220670_at |  | | |  | | -2.12 | 0.02492 |
| 243463_s_at | *RIT1* | | | Ras-like without CAAX 1 | | -2.12 | 0.02704 |
| 217027_x_at |  | | |  | | -2.12 | 0.02892 |
| 239973_at |  | | |  | | -2.12 | 0.03873 |
| 1557895_at | *FLJ35934* | | | FLJ35934 | | -2.12 | 0.04282 |
| 224407_s_at | *MST4* | | | serine/threonine protein kinase MST4 | | -2.13 | 6.72x10^-7^ |
| 238337_s_at | *DNAJC21* | | | DnaJ (Hsp40) homolog, subfamily C, member 21 | | -2.13 | 2.2x10^-5^ |
| 227847_at | *EPM2AIP1* | | | EPM2A (laforin) interacting protein 1 | | -2.13 | 5.4x10^-5^ |
| 216028_at |  | | |  | | -2.13 | 0.00021 |
| 228909_at |  | | |  | | -2.13 | 0.00022 |
| 221423_s_at | *YIPF5* | | | Yip1 domain family, member 5 | | -2.13 | 0.00083 |
| 227912_s_at | *EXOSC3* | | | exosome component 3 | | -2.13 | 0.00111 |
| 226538_at | *MAN2A1* | | | mannosidase, alpha, class 2A, member 1 | | -2.13 | 0.00216 |
| 218689_at | *FANCF* | | | Fanconi anemia, complementation group F | | -2.13 | 0.00329 |
| 211092_s_at | *NF2* | | | neurofibromin 2 (merlin) | | -2.13 | 0.00344 |
| 226660_at | *RPS6KB1* | | | ribosomal protein S6 kinase, 70kDa, polypeptide 1 | | -2.13 | 0.00399 |
| 204162_at | *NDC80* | | | NDC80 kinetochore complex component | | -2.13 | 0.00421 |
| 203939_at | *NT5E* | | | 5'-nucleotidase, ecto (CD73) | | -2.13 | 0.0043 |
| 214948_s_at | *TMF1* | | | TATA element modulatory factor 1 | | -2.13 | 0.00514 |
| 214427_at | *NOP2* | | | NOP2 nucleolar protein | | -2.13 | 0.00534 |
| 225222_at | *HIAT1* | | | hippocampus abundant transcript 1 | | -2.13 | 0.00577 |
| 235939_at |  | | |  | | -2.13 | 0.00637 |
| 242302_at | *PDS5B* | | | PDS5, regulator of cohesion maintenance, homolog B (S. cerevisiae) | | -2.13 | 0.00785 |
| 1556121_at | *NAP1L1* | | | nucleosome assembly protein 1-like 1 | | -2.13 | 0.009 |
| 221570_s_at | *METTL5* | | | methyltransferase like 5 | | -2.13 | 0.0096 |
| 226496_at | *ZCCHC7* | | | zinc finger, CCHC domain containing 7 | | -2.13 | 0.01099 |
| 234110_at |  | | |  | | -2.13 | 0.01401 |
| 204084_s_at | *CLN5* | | | ceroid-lipofuscinosis, neuronal 5 | | -2.13 | 0.01462 |
| 225720_at | *SYNPO2* | | | synaptopodin 2 | | -2.13 | 0.01516 |
| 203983_at | *TSNAX* | | | translin-associated factor X | | -2.13 | 0.01834 |
| 228530_at | *MZT1* | | | mitotic spindle organizing protein 1 | | -2.13 | 0.01898 |
| 235241_at | *SLC38A9* | | | solute carrier family 38, member 9 | | -2.13 | 0.01975 |
| 203404_at | *ARMCX2* | | | armadillo repeat containing, X-linked 2 | | -2.13 | 0.02061 |
| 226505_x_at | *USP32* | | | ubiquitin specific peptidase 32 | | -2.13 | 0.02087 |
| 226948_at | *RHBDD1* | | | rhomboid domain containing 1 | | -2.13 | 0.02321 |
| 204327_s_at | *ZNF202* | | | zinc finger protein 202 | | -2.13 | 0.0237 |
| 235629_at |  | | |  | | -2.13 | 0.02576 |
| 241780_at |  | | |  | | -2.13 | 0.02953 |
| 237408_at | *DCUN1D1* | | | DCN1, defective in cullin neddylation 1, domain containing 1 | | -2.13 | 0.02994 |
| 220201_at | *RC3H2* | | | ring finger and CCCH-type domains 2 | | -2.13 | 0.03419 |
| 219949_at | *LRRC2* | | | leucine rich repeat containing 2 | | -2.13 | 0.04765 |
| 227288_at | *SREK1IP1* | | | SREK1-interacting protein 1 | | -2.13 | 0.04966 |
| 222669_s_at | *SBDS* | | | Shwachman-Bodian-Diamond syndrome; Shwachman-Bodian-Diamond syndrome pseudogene 1 | | -2.14 | 1.89x10^-7^ |
| 238512_at |  | | |  | | -2.14 | 9.6x10^-5^ |
| 212020_s_at | *MKI67* | | | antigen identified by monoclonal antibody Ki-67 | | -2.14 | 0.00026 |
| 207098_s_at | *MFN1* | | | mitofusin 1 | | -2.14 | 0.00044 |
| 224888_at | *EPT1* | | | ethanolaminephosphotransferase 1 (CDP-ethanolamine-specific) | | -2.14 | 0.0011 |
| 201805_at | *PRKAG1* | | | protein kinase, AMP-activated, gamma 1 non-catalytic subunit | | -2.14 | 0.00171 |
| 204897_at | *PTGER4* | | | prostaglandin E receptor 4 (subtype EP4) | | -2.14 | 0.00195 |
| 204128_s_at | *RFC3* | | | replication factor C (activator 1) 3, 38kDa | | -2.14 | 0.00225 |
| 235689_at | *MTFMT* | | | mitochondrial methionyl-tRNA formyltransferase | | -2.14 | 0.00238 |
| 233608_at |  | | |  | | -2.14 | 0.00252 |
| 224829_at | *CPEB4* | | | cytoplasmic polyadenylation element binding protein 4 | | -2.14 | 0.0027 |
| 221750_at | *HMGCS1* | | | 3-hydroxy-3-methylglutaryl-CoA synthase 1 (soluble) | | -2.14 | 0.00297 |
| 233656_s_at | *VPS54* | | | vacuolar protein sorting 54 homolog (S. cerevisiae) | | -2.14 | 0.00338 |
| 210875_s_at |  | | |  | | -2.14 | 0.00374 |
| 204821_at | *BTN3A3* | | | butyrophilin, subfamily 3, member A3 | | -2.14 | 0.00389 |
| 225688_s_at | *PHLDB2* | | | pleckstrin homology-like domain, family B, member 2 | | -2.14 | 0.00389 |
| 201784_s_at | *C11orf58* | | | chromosome 11 open reading frame 58 | | -2.14 | 0.00406 |
| 202435_s_at | *CYP1B1* | | | cytochrome P450, family 1, subfamily B, polypeptide 1 | | -2.14 | 0.00476 |
| 226194_at | *CHAMP1* | | | chromosome alignment maintaining phosphoprotein 1 | | -2.14 | 0.00517 |
| 204356_at | *LIMK1* | | | LIM domain kinase 1 | | -2.14 | 0.00532 |
| 218716_x_at | *MTO1* | | | mitochondrial tRNA translation optimization 1 | | -2.14 | 0.00683 |
| 233375_at | *EFCAB2* | | | EF-hand calcium binding domain 2 | | -2.14 | 0.00715 |
| 221918_at | *CDK17* | | | cyclin-dependent kinase 17 | | -2.14 | 0.00791 |
| 235179_at | *ZNF641* | | | zinc finger protein 641 | | -2.14 | 0.00791 |
| 204011_at | *SPRY2* | | | sprouty homolog 2 (Drosophila) | | -2.14 | 0.00941 |
| 234915_s_at | *DENR* | | | density-regulated protein | | -2.14 | 0.00998 |
| 235142_at | *ZBTB8A* | | | zinc finger and BTB domain containing 8A | | -2.14 | 0.01092 |
| 212978_at | *LRRC8B* | | | leucine rich repeat containing 8 family, member B | | -2.14 | 0.014 |
| 209065_at | *UQCRB* | | | ubiquinol-cytochrome c reductase binding protein | | -2.14 | 0.0141 |
| 243176_at | *ARL5A* | | | ADP-ribosylation factor-like 5A | | -2.14 | 0.02057 |
| 224309_s_at | *SUGT1* | | | SGT1, suppressor of G2 allele of SKP1 (S. cerevisiae) | | -2.14 | 0.02208 |
| 225468_at | *PATL1* | | | protein associated with topoisomerase II homolog 1 (yeast) | | -2.14 | 0.02278 |
| 214658_at | *TMED7* | | | transmembrane emp24 protein transport domain containing 7; TMED7-TICAM2 readthrough | | -2.14 | 0.02582 |
| 211814_s_at | *CCNE2* | | | cyclin E2 | | -2.14 | 0.03079 |
| 1556964_s_at |  | | |  | | -2.14 | 0.03134 |
| 1556007_s_at |  | | |  | | -2.14 | 0.03201 |
| 230621_at | *IAH1* | | | isoamyl acetate-hydrolyzing esterase 1 homolog (S. cerevisiae) | | -2.14 | 0.03777 |
| 214709_s_at | *KTN1* | | | kinectin 1 (kinesin receptor) | | -2.14 | 0.04657 |
| 227481_at | *CNKSR3* | | | CNKSR family member 3 | | -2.15 | 8.6x10^-5^ |
| 205126_at | *VRK2* | | | vaccinia related kinase 2 | | -2.15 | 0.00058 |
| 201175_at | *TMX2* | | | thioredoxin-related transmembrane protein 2 | | -2.15 | 0.0009 |
| 206096_at | *ZNF35* | | | zinc finger protein 35 | | -2.15 | 0.00093 |
| 1555679_a_at | *RTN4IP1* | | | reticulon 4 interacting protein 1 | | -2.15 | 0.0011 |
| 225116_at | *HIPK2* | | | homeodomain interacting protein kinase 2 | | -2.15 | 0.0012 |
| 226775_at | *ENY2* | | | enhancer of yellow 2 homolog (Drosophila) | | -2.15 | 0.00139 |
| 214694_at | *MPRIP* | | | myosin phosphatase Rho interacting protein | | -2.15 | 0.00302 |
| 218527_at | *APTX* | | | aprataxin | | -2.15 | 0.00315 |
| 226758_at | *LUC7L2* | | | LUC7-like 2 (S. cerevisiae) | | -2.15 | 0.00345 |
| 231896_s_at | *DENR* | | | density-regulated protein | | -2.15 | 0.00468 |
| 220841_s_at | *AHI1* | | | Abelson helper integration site 1 | | -2.15 | 0.00494 |
| 203621_at | *NDUFB5* | | | NADH dehydrogenase (ubiquinone) 1 beta subcomplex, 5, 16kDa | | -2.15 | 0.00496 |
| 206025_s_at | *TNFAIP6* | | | tumor necrosis factor, alpha-induced protein 6 | | -2.15 | 0.00536 |
| 206254_at | *EGF* | | | epidermal growth factor | | -2.15 | 0.00586 |
| 1555996_s_at | *EIF4A2* | | | eukaryotic translation initiation factor 4A2; microRNA 1248; small nucleolar RNA, H/ACA box 4; small nucleolar RNA, H/ACA box 63; small nucleolar RNA, H/ACA box 81; small nucleolar RNA, C/D box 2 | | -2.15 | 0.0067 |
| 224446_at | *LLPH* | | | LLP homolog, long-term synaptic facilitation (Aplysia); protein LLP homolog | | -2.15 | 0.0071 |
| 224989_at |  | | |  | | -2.15 | 0.00715 |
| 219356_s_at | *CHMP5* | | | charged multivesicular body protein 5 | | -2.15 | 0.00725 |
| 212138_at | *PDS5A* | | | PDS5, regulator of cohesion maintenance, homolog A (S. cerevisiae) | | -2.15 | 0.0073 |
| 210544_s_at | *ALDH3A2* | | | aldehyde dehydrogenase 3 family, member A2 | | -2.15 | 0.00796 |
| 203338_at | *PPP2R5E* | | | protein phosphatase 2, regulatory subunit B', epsilon isoform | | -2.15 | 0.00807 |
| 1559156_at |  | | |  | | -2.15 | 0.00897 |
| 236204_at | *COPS8* | | | COP9 signalosome subunit 8 | | -2.15 | 0.00972 |
| 204185_x_at | *PPID* | | | peptidylprolyl isomerase D | | -2.15 | 0.0102 |
| 219161_s_at | *CKLF* | | | chemokine-like factor; CKLF-CMTM1 readthrough | | -2.15 | 0.01198 |
| 209459_s_at | *ABAT* | | | 4-aminobutyrate aminotransferase | | -2.15 | 0.01262 |
| 239897_at | *BCLAF1* | | | BCL2-associated transcription factor 1 | | -2.15 | 0.01413 |
| 1555125_at | *PAXBP1* | | | PAX3 and PAX7 binding protein 1 | | -2.15 | 0.01423 |
| 1553538_s_at | *COX1* | | | cytochrome c oxidase subunit I | | -2.15 | 0.0155 |
| 223596_at | *SLC12A6* | | | solute carrier family 12 (potassium/chloride transporters), member 6 | | -2.15 | 0.01669 |
| 214047_s_at | *MBD4* | | | methyl-CpG binding domain protein 4 | | -2.15 | 0.017 |
| 200073_s_at | *HNRNPD* | | | heterogeneous nuclear ribonucleoprotein D (AU-rich element RNA binding protein 1, 37kDa) | | -2.15 | 0.01772 |
| 228045_at |  | | |  | | -2.15 | 0.01836 |
| 228032_s_at | *DENND1B* | | | DENN/MADD domain containing 1B | | -2.15 | 0.02008 |
| 243613_at | *DENND5B* | | | DENN/MADD domain containing 5B | | -2.15 | 0.02327 |
| 232740_at | *MCM3AP-AS1* | | | MCM3AP antisense RNA 1 | | -2.15 | 0.02406 |
| 210407_at | *PPM1A* | | | protein phosphatase, Mg2+/Mn2+ dependent, 1A | | -2.15 | 0.02499 |
| 224619_at | *CASC4* | | | cancer susceptibility candidate 4 | | -2.15 | 0.02621 |
| 209523_at | *TAF2* | | | TAF2 RNA polymerase II, TATA box binding protein (TBP)-associated factor, 150kDa | | -2.15 | 0.02766 |
| 228289_at | *BRD7* | | | bromodomain containing 7 | | -2.15 | 0.03008 |
| 239151_at | *BMS1P1* | | | BMS1 pseudogene 1; BMS1 pseudogene 5; uncharacterized LOC100996797 | | -2.15 | 0.03061 |
| 211355_x_at | *LEPR* | | | leptin receptor | | -2.15 | 0.04112 |
| 210910_s_at | *POMZP3* | | | POM121 and ZP3 fusion | | -2.16 | 6x10^-6^ |
| 1552344_s_at | *CNOT7* | | | CCR4-NOT transcription complex, subunit 7 | | -2.16 | 0.00016 |
| 238706_at | *PAPD4* | | | PAP associated domain containing 4 | | -2.16 | 0.00077 |
| 232589_at |  | | |  | | -2.16 | 0.00094 |
| 1558369_at | *MPHOSPH9* | | | M-phase phosphoprotein 9 | | -2.16 | 0.00101 |
| 232379_at | *SKIL* | | | SKI-like oncogene | | -2.16 | 0.0012 |
| 221541_at | *CRISPLD2* | | | cysteine-rich secretory protein LCCL domain containing 2 | | -2.16 | 0.0014 |
| 222404_x_at | *PTPLAD1* | | | protein tyrosine phosphatase-like A domain containing 1 | | -2.16 | 0.0015 |
| 1556153_s_at |  | | |  | | -2.16 | 0.00175 |
| 225745_at | *LRP6* | | | low density lipoprotein receptor-related protein 6 | | -2.16 | 0.0031 |
| 222132_s_at | *AGK* | | | acylglycerol kinase | | -2.16 | 0.00326 |
| 219588_s_at | *NCAPG2* | | | non-SMC condensin II complex, subunit G2 | | -2.16 | 0.00335 |
| 206061_s_at | *DICER1* | | | dicer 1, ribonuclease type III | | -2.16 | 0.00374 |
| 221895_at | *MOSPD2* | | | motile sperm domain containing 2 | | -2.16 | 0.0038 |
| 222845_x_at | *TMBIM4* | | | transmembrane BAX inhibitor motif containing 4 | | -2.16 | 0.00403 |
| 211935_at | *ARL6IP1* | | | ADP-ribosylation factor-like 6 interacting protein 1 | | -2.16 | 0.00409 |
| 202579_x_at | *HMGN4* | | | high mobility group nucleosomal binding domain 4 | | -2.16 | 0.00421 |
| 217542_at | *MDM2* | | | MDM2 oncogene, E3 ubiquitin protein ligase | | -2.16 | 0.00432 |
| 215936_s_at | *KIAA1033* | | | KIAA1033 | | -2.16 | 0.00501 |
| 231199_at |  | | |  | | -2.16 | 0.006 |
| 231863_at | *ING3* | | | inhibitor of growth family, member 3 | | -2.16 | 0.00653 |
| 202786_at | *STK39* | | | serine threonine kinase 39 | | -2.16 | 0.00659 |
| 235603_at | *HNRNPU* | | | heterogeneous nuclear ribonucleoprotein U (scaffold attachment factor A) | | -2.16 | 0.00709 |
| 220987_s_at | *AKIP1* | | | A kinase (PRKA) interacting protein 1; NUAK family, SNF1-like kinase, 2 | | -2.16 | 0.00858 |
| 217452_s_at | *B3GALT2* | | | UDP-Gal:betaGlcNAc beta 1,3-galactosyltransferase, polypeptide 2 | | -2.16 | 0.00948 |
| 217916_s_at | *FAM49B* | | | family with sequence similarity 49, member B | | -2.16 | 0.00971 |
| 229284_at | *MAT2B* | | | methionine adenosyltransferase II, beta | | -2.16 | 0.01097 |
| 217097_s_at | *PHTF2* | | | putative homeodomain transcription factor 2 | | -2.16 | 0.01107 |
| 201687_s_at | *API5* | | | apoptosis inhibitor 5 | | -2.16 | 0.01137 |
| 226338_at | *TMEM55A* | | | transmembrane protein 55A | | -2.16 | 0.0117 |
| 243495_s_at | *ZNF652* | | | zinc finger protein 652 | | -2.16 | 0.01248 |
| 227569_at | *LNX2* | | | ligand of numb-protein X 2 | | -2.16 | 0.01501 |
| 226353_at | *SPPL2A* | | | signal peptide peptidase like 2A | | -2.16 | 0.01589 |
| 1568955_at | *SRGAP2* | | | SLIT-ROBO Rho GTPase activating protein 2; SLIT-ROBO Rho GTPase activating protein 2C; SLIT-ROBO Rho GTPase activating protein 2D | | -2.16 | 0.01681 |
| 238435_at |  | | |  | | -2.16 | 0.01758 |
| 212211_at | *ANKRD17* | | | ankyrin repeat domain 17 | | -2.16 | 0.01909 |
| 204075_s_at | *CEP104* | | | centrosomal protein 104kDa | | -2.16 | 0.01925 |
| 201312_s_at | *SH3BGRL* | | | SH3 domain binding glutamic acid-rich protein like | | -2.16 | 0.02462 |
| 228776_at | *GJC1* | | | gap junction protein, gamma 1, 45kDa | | -2.16 | 0.02527 |
| 228991_at | *CDK13* | | | cyclin-dependent kinase 13 | | -2.16 | 0.03278 |
| 204739_at | *CENPC1* | | | centromere protein C 1 | | -2.16 | 0.03456 |
| 214984_at |  | | |  | | -2.16 | 0.03818 |
| 226326_at | *PCGF5* | | | polycomb group ring finger 5 | | -2.16 | 0.044 |
| 220615_s_at | *FAR2* | | | fatty acyl CoA reductase 2 | | -2.17 | 0.0005 |
| 211984_at | *CALM1* | | | calmodulin 1 (phosphorylase kinase, delta); calmodulin 2 (phosphorylase kinase, delta); calmodulin 3 (phosphorylase kinase, delta) | | -2.17 | 0.00148 |
| 201375_s_at | *PPP2CB* | | | protein phosphatase 2, catalytic subunit, beta isozyme | | -2.17 | 0.0015 |
| 201103_x_at |  | | |  | | -2.17 | 0.0016 |
| 227948_at | *FGD4* | | | FYVE, RhoGEF and PH domain containing 4 | | -2.17 | 0.00177 |
| 213020_at | *GOSR1* | | | golgi SNAP receptor complex member 1 | | -2.17 | 0.00205 |
| 200993_at | *IPO7* | | | importin 7 | | -2.17 | 0.00355 |
| 217997_at | *PHLDA1* | | | pleckstrin homology-like domain, family A, member 1 | | -2.17 | 0.00361 |
| 210024_s_at | *UBE2E3* | | | ubiquitin-conjugating enzyme E2E 3 | | -2.17 | 0.0037 |
| 206874_s_at | *SLK* | | | STE20-like kinase | | -2.17 | 0.00535 |
| 217941_s_at | *ERBB2IP* | | | erbb2 interacting protein | | -2.17 | 0.00547 |
| 233230_s_at | *SLAIN2* | | | SLAIN motif family, member 2 | | -2.17 | 0.0056 |
| 1566256_s_at | *GPR180* | | | G protein-coupled receptor 180 | | -2.17 | 0.00579 |
| 228378_at | *C12orf29* | | | chromosome 12 open reading frame 29 | | -2.17 | 0.00616 |
| 206456_at | *GABRA5* | | | gamma-aminobutyric acid (GABA) A receptor, alpha 5 | | -2.17 | 0.00619 |
| 223620_at | *GPR34* | | | G protein-coupled receptor 34 | | -2.17 | 0.00624 |
| 218889_at | *NOC3L* | | | nucleolar complex associated 3 homolog (S. cerevisiae) | | -2.17 | 0.00666 |
| 202547_s_at | *ARHGEF7* | | | Rho guanine nucleotide exchange factor (GEF) 7 | | -2.17 | 0.00816 |
| 212008_at | *UBXN4* | | | UBX domain protein 4 | | -2.17 | 0.00974 |
| 224628_at | *ERLEC1* | | | endoplasmic reticulum lectin 1 | | -2.17 | 0.00998 |
| 213742_at | *SRSF11* | | | serine/arginine-rich splicing factor 11 | | -2.17 | 0.01033 |
| 221825_at | *ANGEL2* | | | angel homolog 2 (Drosophila) | | -2.17 | 0.01231 |
| 1563039_at | *LARGE-AS1* | | | LARGE antisense RNA 1 | | -2.17 | 0.01314 |
| 213410_at | *C10orf137* | | | chromosome 10 open reading frame 137 | | -2.17 | 0.01397 |
| 204566_at | *PPM1D* | | | protein phosphatase, Mg2+/Mn2+ dependent, 1D | | -2.17 | 0.01538 |
| 203804_s_at | *LUC7L3* | | | LUC7-like 3 (S. cerevisiae) | | -2.17 | 0.01766 |
| 221428_s_at | *TBL1XR1* | | | transducin (beta)-like 1 X-linked receptor 1 | | -2.17 | 0.02463 |
| 218583_s_at | *DCUN1D1* | | | DCN1, defective in cullin neddylation 1, domain containing 1 | | -2.17 | 0.02623 |
| 206500_s_at | *MIS18BP1* | | | MIS18 binding protein 1 | | -2.17 | 0.02913 |
| 214774_x_at | *TOX3* | | | TOX high mobility group box family member 3 | | -2.17 | 0.03007 |
| 223608_at | *EFCAB2* | | | EF-hand calcium binding domain 2 | | -2.17 | 0.04592 |
| 221326_s_at | *TUBD1* | | | tubulin, delta 1 | | -2.18 | 0.0004 |
| 200848_at | *AHCYL1* | | | adenosylhomocysteinase-like 1 | | -2.18 | 0.00063 |
| 202280_at |  | | |  | | -2.18 | 0.00069 |
| 212121_at | *TCTN3* | | | tectonic family member 3 | | -2.18 | 0.00103 |
| 225083_at | *GTF3C6* | | | general transcription factor IIIC, polypeptide 6, alpha 35kDa | | -2.18 | 0.00122 |
| 216305_s_at | *GCFC2* | | | GC-rich sequence DNA-binding factor 2 | | -2.18 | 0.00132 |
| 201567_s_at | *GOLGA4* | | | golgin A4 | | -2.18 | 0.00209 |
| 238821_at | *CSTF2* | | | cleavage stimulation factor, 3' pre-RNA, subunit 2, 64kDa | | -2.18 | 0.00248 |
| 225195_at | *DPH3* | | | DPH3, KTI11 homolog (S. cerevisiae) | | -2.18 | 0.00253 |
| 228201_at | *ARL13B* | | | ADP-ribosylation factor-like 13B | | -2.18 | 0.00278 |
| 229454_at | *BCLAF1* | | | BCL2-associated transcription factor 1 | | -2.18 | 0.00403 |
| 210757_x_at | *DAB2* | | | Dab, mitogen-responsive phosphoprotein, homolog 2 (Drosophila) | | -2.18 | 0.00412 |
| 226615_at | *XPR1* | | | xenotropic and polytropic retrovirus receptor 1 | | -2.18 | 0.00419 |
| 212605_s_at |  | | |  | | -2.18 | 0.00423 |
| 239001_at | *MGST1* | | | microsomal glutathione S-transferase 1 | | -2.18 | 0.0048 |
| 224872_at | *DIP2B* | | | DIP2 disco-interacting protein 2 homolog B (Drosophila) | | -2.18 | 0.00494 |
| 200027_at | *NARS* | | | asparaginyl-tRNA synthetase | | -2.18 | 0.00533 |
| 226181_at | *TUBE1* | | | tubulin, epsilon 1 | | -2.18 | 0.00583 |
| 226993_at | *TRIP12* | | | NULL | | -2.18 | 0.0069 |
| 238889_at | *AGBL5* | | | ATP/GTP binding protein-like 5 | | -2.18 | 0.00699 |
| 208840_s_at | *G3BP2* | | | GTPase activating protein (SH3 domain) binding protein 2 | | -2.18 | 0.00726 |
| 232115_at | *SLC39A3* | | | solute carrier family 39 (zinc transporter), member 3 | | -2.18 | 0.00837 |
| 218212_s_at | *MOCS2* | | | molybdenum cofactor synthesis 2 | | -2.18 | 0.00953 |
| 202939_at | *ZMPSTE24* | | | zinc metallopeptidase STE24 | | -2.18 | 0.01078 |
| 200902_at | *Sep-15* | | | 15 kDa selenoprotein | | -2.18 | 0.01411 |
| 203392_s_at | *CTBP1* | | | C-terminal binding protein 1 | | -2.18 | 0.01423 |
| 226965_at | *DENND6A* | | | DENN/MADD domain containing 6A | | -2.18 | 0.01427 |
| 218700_s_at | *RAB7L1* | | | RAB7, member RAS oncogene family-like 1 | | -2.18 | 0.01458 |
| 235159_at |  | | |  | | -2.18 | 0.01462 |
| 227568_at | *HECTD2* | | | HECT domain containing E3 ubiquitin protein ligase 2 | | -2.18 | 0.01874 |
| 212060_at | *U2SURP* | | | U2 snRNP-associated SURP domain containing | | -2.18 | 0.02054 |
| 203182_s_at | *SRPK2* | | | SRSF protein kinase 2 | | -2.18 | 0.02519 |
| 202351_at | *ITGAV* | | | integrin, alpha V | | -2.18 | 0.03163 |
| 209330_s_at | *HNRNPD* | | | heterogeneous nuclear ribonucleoprotein D (AU-rich element RNA binding protein 1, 37kDa) | | -2.18 | 0.04252 |
| 203846_at | *TRIM32* | | | tripartite motif containing 32 | | -2.19 | 0.00041 |
| 227224_at | *RALGPS2* | | | Ral GEF with PH domain and SH3 binding motif 2 | | -2.19 | 0.00061 |
| 236939_at | *PTPLAD2* | | | protein tyrosine phosphatase-like A domain containing 2 | | -2.19 | 0.00113 |
| 219204_s_at | *SRR* | | | serine racemase | | -2.19 | 0.00159 |
| 203200_s_at | *MTRR* | | | 5-methyltetrahydrofolate-homocysteine methyltransferase reductase | | -2.19 | 0.00186 |
| 210824_at |  | | |  | | -2.19 | 0.00187 |
| 214281_s_at | *RCHY1* | | | ring finger and CHY zinc finger domain containing 1, E3 ubiquitin protein ligase | | -2.19 | 0.00211 |
| 220741_s_at | *PPA2* | | | pyrophosphatase (inorganic) 2 | | -2.19 | 0.00224 |
| 209161_at | *PRPF4* | | | PRP4 pre-mRNA processing factor 4 homolog (yeast) | | -2.19 | 0.00392 |
| 232075_at | *WDR61* | | | WD repeat domain 61 | | -2.19 | 0.00503 |
| 201129_at | *SRSF7* | | | serine/arginine-rich splicing factor 7 | | -2.19 | 0.00514 |
| 219303_at | *RNF219* | | | ring finger protein 219 | | -2.19 | 0.00534 |
| 203629_s_at | *COG5* | | | component of oligomeric golgi complex 5 | | -2.19 | 0.00588 |
| 1568777_at | *EML5* | | | echinoderm microtubule associated protein like 5 | | -2.19 | 0.00699 |
| 222537_s_at | *CDC42SE1* | | | CDC42 small effector 1 | | -2.19 | 0.00824 |
| 225113_at | *AGPS* | | | alkylglycerone phosphate synthase | | -2.19 | 0.00936 |
| 226421_at | *AMMECR1* | | | Alport syndrome, mental retardation, midface hypoplasia and elliptocytosis chromosomal region gene 1 | | -2.19 | 0.00978 |
| 225127_at | *TMEM181* | | | transmembrane protein 181 | | -2.19 | 0.01072 |
| 242273_at |  | | |  | | -2.19 | 0.01129 |
| 218350_s_at | *GMNN* | | | geminin, DNA replication inhibitor | | -2.19 | 0.01373 |
| 221550_at | *COX15* | | | cytochrome c oxidase assembly homolog 15 (yeast) | | -2.19 | 0.01514 |
| 208927_at | *SPOP* | | | speckle-type POZ protein | | -2.19 | 0.01709 |
| 217620_s_at | *PIK3CB* | | | phosphatidylinositol-4,5-bisphosphate 3-kinase, catalytic subunit beta | | -2.19 | 0.02111 |
| 213765_at | *MFAP5* | | | microfibrillar associated protein 5 | | -2.19 | 0.02611 |
| 1558233_s_at | *ATF1* | | | activating transcription factor 1 | | -2.19 | 0.02614 |
| 210286_s_at | *SLC4A7* | | | solute carrier family 4, sodium bicarbonate cotransporter, member 7 | | -2.19 | 0.02689 |
| 212927_at | *SMC5* | | | structural maintenance of chromosomes 5 | | -2.19 | 0.02881 |
| 229027_at | *PPM1A* | | | protein phosphatase, Mg2+/Mn2+ dependent, 1A | | -2.19 | 0.03106 |
| 218948_at | *QRSL1* | | | glutaminyl-tRNA synthase (glutamine-hydrolyzing)-like 1 | | -2.19 | 0.03421 |
| 204376_at | *VPRBP* | | | Vpr (HIV-1) binding protein | | -2.19 | 0.03459 |
| 201669_s_at | *MARCKS* | | | myristoylated alanine-rich protein kinase C substrate | | -2.19 | 0.0346 |
| 222235_s_at | *CSGALNACT2* | | | chondroitin sulfate N-acetylgalactosaminyltransferase 2 | | -2.19 | 0.03553 |
| 217731_s_at | *ITM2B* | | | integral membrane protein 2B | | -2.19 | 0.04335 |
| 229109_s_at | *BLVRA* | | | biliverdin reductase A | | -2.2 | 0.00021 |
| 1554873_at | *CSPP1* | | | centrosome and spindle pole associated protein 1 | | -2.2 | 0.00037 |
| 225087_at | *FOPNL* | | | FGFR1OP N-terminal like | | -2.2 | 0.00063 |
| 1559052_s_at | *PAK2* | | | p21 protein (Cdc42/Rac)-activated kinase 2 | | -2.2 | 0.00118 |
| 235345_at | *RUFY2* | | | RUN and FYVE domain containing 2 | | -2.2 | 0.00161 |
| 1559914_at |  | | |  | | -2.2 | 0.00163 |
| 242981_at |  | | |  | | -2.2 | 0.002 |
| 226853_at | *BMP2K* | | | BMP2 inducible kinase | | -2.2 | 0.00258 |
| 225297_at | *HAUS1* | | | HAUS augmin-like complex, subunit 1 | | -2.2 | 0.00261 |
| 225348_at |  | | |  | | -2.2 | 0.00311 |
| 218854_at | *DSE* | | | dermatan sulfate epimerase | | -2.2 | 0.00314 |
| 229622_at | *FAM132B* | | | family with sequence similarity 132, member B | | -2.2 | 0.00364 |
| 235964_x_at | *SAMHD1* | | | SAM domain and HD domain 1 | | -2.2 | 0.00422 |
| 213129_s_at | *GCSH* | | | glycine cleavage system protein H (aminomethyl carrier); glycine cleavage system H protein, mitochondrial-like | | -2.2 | 0.00435 |
| 205084_at | *BCAP29* | | | B-cell receptor-associated protein 29 | | -2.2 | 0.0044 |
| 200594_x_at | *HNRNPU* | | | heterogeneous nuclear ribonucleoprotein U (scaffold attachment factor A) | | -2.2 | 0.00548 |
| 213111_at | *PIKFYVE* | | | phosphoinositide kinase, FYVE finger containing | | -2.2 | 0.00625 |
| 225662_at | *ZAK* | | | sterile alpha motif and leucine zipper containing kinase AZK | | -2.2 | 0.00709 |
| 227551_at | *ABHD17B* | | | abhydrolase domain containing 17B | | -2.2 | 0.00852 |
| 230561_s_at | *KANSL1L* | | | KAT8 regulatory NSL complex subunit 1-like | | -2.2 | 0.00891 |
| 1569827_at | *ATG7* | | | autophagy related 7 | | -2.2 | 0.00917 |
| 201773_at | *ADNP* | | | activity-dependent neuroprotector homeobox | | -2.2 | 0.01011 |
| 1557360_at | *LRPPRC* | | | leucine-rich pentatricopeptide repeat containing | | -2.2 | 0.01121 |
| 213911_s_at | *H2AFZ* | | | H2A histone family, member Z | | -2.2 | 0.01266 |
| 217577_at |  | | |  | | -2.2 | 0.01292 |
| 232857_at | *POLR3H* | | | polymerase (RNA) III (DNA directed) polypeptide H (22.9kD) | | -2.2 | 0.01382 |
| 210748_at |  | | |  | | -2.2 | 0.01726 |
| 1569362_at | *ALCAM* | | | activated leukocyte cell adhesion molecule | | -2.2 | 0.01944 |
| 236235_at | *ITCH* | | | itchy E3 ubiquitin protein ligase | | -2.2 | 0.02003 |
| 1555836_at | *POLR2B* | | | polymerase (RNA) II (DNA directed) polypeptide B, 140kDa | | -2.2 | 0.02053 |
| 243908_at |  | | |  | | -2.2 | 0.02129 |
| 201449_at | *TIA1* | | | TIA1 cytotoxic granule-associated RNA binding protein | | -2.2 | 0.02259 |
| 222607_s_at | *DIS3* | | | DIS3 mitotic control homolog (S. cerevisiae) | | -2.2 | 0.02353 |
| 240370_at |  | | |  | | -2.2 | 0.02423 |
| 215758_x_at | *ZNF93* | | | zinc finger protein 93 | | -2.2 | 0.02634 |
| 212122_at | *RHOQ* | | | ras homolog family member Q | | -2.2 | 0.02891 |
| 1565633_at |  | | |  | | -2.2 | 0.04477 |
| 203545_at | *ALG8* | | | ALG8, alpha-1,3-glucosyltransferase | | -2.21 | 0.00028 |
| 221547_at | *PRPF18* | | | pre-mRNA processing factor 18 | | -2.21 | 0.00031 |
| 213982_s_at | *RABGAP1L* | | | RAB GTPase activating protein 1-like | | -2.21 | 0.00046 |
| 209699_x_at | *AKR1C2* | | | aldo-keto reductase family 1, member C2; aldo-keto reductase family 1 member C2-like | | -2.21 | 0.00051 |
| 243405_at |  | | |  | | -2.21 | 0.00086 |
| 204207_s_at | *RNGTT* | | | RNA guanylyltransferase and 5'-phosphatase | | -2.21 | 0.001 |
| 206809_s_at | *HNRNPA3* | | | heterogeneous nuclear ribonucleoprotein A3; heterogeneous nuclear ribonucleoprotein A3 pseudogene 1 | | -2.21 | 0.00108 |
| 206948_at | *NEU3* | | | sialidase 3 (membrane sialidase) | | -2.21 | 0.00133 |
| 213051_at | *ZC3HAV1* | | | zinc finger CCCH-type, antiviral 1 | | -2.21 | 0.00153 |
| 234874_at | *ATE1* | | | arginyltransferase 1 | | -2.21 | 0.00157 |
| 205453_at | *HOXB2* | | | homeobox B2 | | -2.21 | 0.00195 |
| 203688_at | *PKD2* | | | polycystic kidney disease 2 (autosomal dominant) | | -2.21 | 0.00303 |
| 228011_at | *FAM92A1* | | | family with sequence similarity 92, member A1 | | -2.21 | 0.00344 |
| 220060_s_at | *PARPBP* | | | PARP1 binding protein | | -2.21 | 0.00406 |
| 213118_at | *UHRF1BP1L* | | | UHRF1 binding protein 1-like | | -2.21 | 0.00514 |
| 203359_s_at | *GJA9-MYCBP* | | | GJA9-MYCBP readthrough; c-myc binding protein | | -2.21 | 0.00535 |
| 224760_at | *SP1* | | | Sp1 transcription factor | | -2.21 | 0.00553 |
| 242560_at | *FANCD2* | | | Fanconi anemia, complementation group D2 | | -2.21 | 0.00653 |
| 201760_s_at | *WSB2* | | | WD repeat and SOCS box containing 2 | | -2.21 | 0.00679 |
| 1554482_a_at | *SAR1B* | | | SAR1 homolog B (S. cerevisiae) | | -2.21 | 0.00717 |
| 202429_s_at | *PPP3CA* | | | protein phosphatase 3, catalytic subunit, alpha isozyme | | -2.21 | 0.00733 |
| 201393_s_at | *IGF2R* | | | insulin-like growth factor 2 receptor | | -2.21 | 0.00781 |
| 228254_at | *STAM2* | | | signal transducing adaptor molecule (SH3 domain and ITAM motif) 2 | | -2.21 | 0.00913 |
| 1568813_at |  | | |  | | -2.21 | 0.00985 |
| 224881_at | *VKORC1L1* | | | vitamin K epoxide reductase complex, subunit 1-like 1 | | -2.21 | 0.01156 |
| 1554450_s_at | *MIER3* | | | mesoderm induction early response 1, family member 3 | | -2.21 | 0.0118 |
| 228208_x_at | *ZNF354C* | | | zinc finger protein 354C | | -2.21 | 0.01234 |
| 226290_at | *BDP1* | | | B double prime 1, subunit of RNA polymerase III transcription initiation factor IIIB | | -2.21 | 0.01283 |
| 203284_s_at | *HS2ST1* | | | heparan sulfate 2-O-sulfotransferase 1 | | -2.21 | 0.01284 |
| 222014_x_at | *MTO1* | | | mitochondrial tRNA translation optimization 1 | | -2.21 | 0.0132 |
| 1559284_at |  | | |  | | -2.21 | 0.01438 |
| 230413_s_at |  | | |  | | -2.21 | 0.01472 |
| 231920_s_at | *CSNK1G1* | | | casein kinase 1, gamma 1 | | -2.21 | 0.01622 |
| 207941_s_at | *RBM39* | | | RNA binding motif protein 39 | | -2.21 | 0.0178 |
| 227623_at | *CACNA2D1* | | | calcium channel, voltage-dependent, alpha 2/delta subunit 1 | | -2.21 | 0.01985 |
| 236989_at |  | | |  | | -2.21 | 0.02039 |
| 221039_s_at | *ASAP1* | | | ArfGAP with SH3 domain, ankyrin repeat and PH domain 1 | | -2.21 | 0.02158 |
| 238035_at | *SP3* | | | Sp3 transcription factor | | -2.21 | 0.02608 |
| 216221_s_at | *PUM2* | | | pumilio homolog 2 (Drosophila) | | -2.21 | 0.02799 |
| 1564381_s_at |  | | |  | | -2.21 | 0.02837 |
| 219649_at | *ALG6* | | | ALG6, alpha-1,3-glucosyltransferase | | -2.21 | 0.03107 |
| 209259_s_at | *SMC3* | | | structural maintenance of chromosomes 3 | | -2.21 | 0.03282 |
| 229551_x_at | *ZNF367* | | | zinc finger protein 367 | | -2.21 | 0.03881 |
| 214119_s_at | *FKBP1A* | | | FK506 binding protein 1A, 12kDa | | -2.21 | 0.04024 |
| 216961_s_at | *RPAIN* | | | RPA interacting protein | | -2.21 | 0.04558 |
| 201756_at | *RPA2* | | | replication protein A2, 32kDa | | -2.22 | 1.77x10^-7^ |
| 203116_s_at | *FECH* | | | ferrochelatase | | -2.22 | 0.00037 |
| 202780_at | *OXCT1* | | | 3-oxoacid CoA transferase 1 | | -2.22 | 0.00134 |
| 210054_at | *HAUS3* | | | HAUS augmin-like complex, subunit 3 | | -2.22 | 0.00214 |
| 1560715_at | *P4HA3* | | | prolyl 4-hydroxylase, alpha polypeptide III | | -2.22 | 0.0023 |
| 204151_x_at | *AKR1C1* | | | aldo-keto reductase family 1, member C1 | | -2.22 | 0.00243 |
| 225397_at | *C15orf57* | | | chromosome 15 open reading frame 57 | | -2.22 | 0.00291 |
| 220199_s_at | *AIDA* | | | axin interactor, dorsalization associated | | -2.22 | 0.00296 |
| 224561_s_at | *MORF4L1* | | | mortality factor 4 like 1 | | -2.22 | 0.0032 |
| 210117_at | *SPAG1* | | | sperm associated antigen 1 | | -2.22 | 0.00339 |
| 212033_at | *RBM25* | | | RNA binding motif protein 25 | | -2.22 | 0.00341 |
| 202538_s_at | *CHMP2B* | | | charged multivesicular body protein 2B | | -2.22 | 0.0042 |
| 213262_at | *SACS* | | | spastic ataxia of Charlevoix-Saguenay (sacsin) | | -2.22 | 0.00443 |
| 217724_at | *SERBP1* | | | SERPINE1 mRNA binding protein 1 | | -2.22 | 0.00446 |
| 211684_s_at | *DYNC1I2* | | | dynein, cytoplasmic 1, intermediate chain 2 | | -2.22 | 0.00516 |
| 213005_s_at | *KANK1* | | | KN motif and ankyrin repeat domains 1 | | -2.22 | 0.00555 |
| 229450_at | *IFIT3* | | | interferon-induced protein with tetratricopeptide repeats 3 | | -2.22 | 0.00604 |
| 203525_s_at | *APC* | | | adenomatous polyposis coli | | -2.22 | 0.0061 |
| 238615_at | *ERLIN2* | | | ER lipid raft associated 2 | | -2.22 | 0.00628 |
| 201146_at | *NFE2L2* | | | nuclear factor (erythroid-derived 2)-like 2 | | -2.22 | 0.00861 |
| 201634_s_at | *CYB5B* | | | cytochrome b5 type B (outer mitochondrial membrane) | | -2.22 | 0.01072 |
| 203312_x_at | *ARF6* | | | ADP-ribosylation factor 6 | | -2.22 | 0.01132 |
| 209898_x_at | *ITSN2* | | | intersectin 2 | | -2.22 | 0.01141 |
| 239108_at | *FAR2* | | | fatty acyl CoA reductase 2 | | -2.22 | 0.01166 |
| 206157_at | *PTX3* | | | pentraxin 3, long | | -2.22 | 0.0117 |
| 217870_s_at | *CMPK1* | | | cytidine monophosphate (UMP-CMP) kinase 1, cytosolic | | -2.22 | 0.01177 |
| 229712_at | *SNAPC3* | | | small nuclear RNA activating complex, polypeptide 3, 50kDa | | -2.22 | 0.01513 |
| 225265_at | *RBMS1* | | | RNA binding motif, single stranded interacting protein 1 | | -2.22 | 0.01548 |
| 226069_at | *PRICKLE1* | | | prickle homolog 1 (Drosophila) | | -2.22 | 0.01775 |
| 242963_at | *SGMS2* | | | sphingomyelin synthase 2 | | -2.22 | 0.01927 |
| 227020_at | *YPEL2* | | | yippee-like 2 (Drosophila) | | -2.22 | 0.02388 |
| 239100_x_at | *PCNX* | | | pecanex homolog (Drosophila) | | -2.22 | 0.02719 |
| 212779_at | *KIAA1109* | | | KIAA1109 | | -2.22 | 0.02736 |
| 235512_at | *CDKL1* | | | cyclin-dependent kinase-like 1 (CDC2-related kinase) | | -2.22 | 0.03191 |
| 203608_at | *ALDH5A1* | | | aldehyde dehydrogenase 5 family, member A1 | | -2.22 | 0.03561 |
| 1558522_at |  | | |  | | -2.22 | 0.03854 |
| 218197_s_at | *OXR1* | | | oxidation resistance 1 | | -2.22 | 0.03992 |
| 222627_at | *VPS54* | | | vacuolar protein sorting 54 homolog (S. cerevisiae) | | -2.22 | 0.04029 |
| 1556144_at | *DHX30* | | | DEAH (Asp-Glu-Ala-His) box polypeptide 30 | | -2.22 | 0.04322 |
| 239014_at | *CCAR1* | | | cell division cycle and apoptosis regulator 1 | | -2.22 | 0.04383 |
| 204341_at | *TRIM16* | | | tripartite motif containing 16 | | -2.23 | 1.8x10^-5^ |
| 205135_s_at | *NUFIP1* | | | nuclear fragile X mental retardation protein interacting protein 1 | | -2.23 | 0.00013 |
| 220153_at | *ENTPD7* | | | ectonucleoside triphosphate diphosphohydrolase 7 | | -2.23 | 0.00031 |
| 209921_at | *SLC7A11* | | | solute carrier family 7 (anionic amino acid transporter light chain, xc- system), member 11 | | -2.23 | 0.00069 |
| 205351_at | *GGCX* | | | gamma-glutamyl carboxylase | | -2.23 | 0.00092 |
| 1568012_at | *CLIP1* | | | CAP-GLY domain containing linker protein 1 | | -2.23 | 0.00135 |
| 212624_s_at | *CHN1* | | | chimerin 1 | | -2.23 | 0.00162 |
| 210275_s_at | *ZFAND5* | | | zinc finger, AN1-type domain 5 | | -2.23 | 0.00258 |
| 202203_s_at | *AMFR* | | | autocrine motility factor receptor, E3 ubiquitin protein ligase | | -2.23 | 0.00314 |
| 202695_s_at | *STK17A* | | | serine/threonine kinase 17a | | -2.23 | 0.00431 |
| 229881_at | *KLF12* | | | Kruppel-like factor 12 | | -2.23 | 0.00433 |
| 238516_at | *BMPR2* | | | bone morphogenetic protein receptor, type II (serine/threonine kinase) | | -2.23 | 0.0044 |
| 230147_at | *F2RL2* | | | coagulation factor II (thrombin) receptor-like 2 | | -2.23 | 0.00491 |
| 235341_at | *DNAJC3* | | | DnaJ (Hsp40) homolog, subfamily C, member 3 | | -2.23 | 0.00509 |
| 203856_at | *VRK1* | | | vaccinia related kinase 1 | | -2.23 | 0.00639 |
| 221816_s_at | *PHF11* | | | PHD finger protein 11 | | -2.23 | 0.00656 |
| 202930_s_at | *SUCLA2* | | | succinate-CoA ligase, ADP-forming, beta subunit | | -2.23 | 0.00773 |
| 219353_at | *NHLRC2* | | | NHL repeat containing 2 | | -2.23 | 0.00845 |
| 218013_x_at | *DCTN4* | | | dynactin 4 (p62) | | -2.23 | 0.00881 |
| 231689_at | *TRPM7* | | | transient receptor potential cation channel, subfamily M, member 7 | | -2.23 | 0.00921 |
| 226255_at | *ZBTB33* | | | zinc finger and BTB domain containing 33 | | -2.23 | 0.01038 |
| 201273_s_at | *SRP9* | | | signal recognition particle 9kDa | | -2.23 | 0.01039 |
| 216933_x_at | *APC* | | | adenomatous polyposis coli | | -2.23 | 0.0111 |
| 218924_s_at | *CTBS* | | | chitobiase, di-N-acetyl- | | -2.23 | 0.01113 |
| 226312_at | *RICTOR* | | | RPTOR independent companion of MTOR, complex 2 | | -2.23 | 0.01188 |
| 218663_at | *NCAPG* | | | non-SMC condensin I complex, subunit G | | -2.23 | 0.01266 |
| 204160_s_at | *ENPP4* | | | ectonucleotide pyrophosphatase/phosphodiesterase 4 (putative) | | -2.23 | 0.01276 |
| 222777_s_at | *WHSC1* | | | Wolf-Hirschhorn syndrome candidate 1 | | -2.23 | 0.01385 |
| 231894_at | *SARS* | | | seryl-tRNA synthetase | | -2.23 | 0.01459 |
| 208669_s_at | *EID1* | | | EP300 interacting inhibitor of differentiation 1 | | -2.23 | 0.01465 |
| 229495_at | *PM20D2* | | | peptidase M20 domain containing 2 | | -2.23 | 0.015 |
| 221865_at | *C9orf91* | | | chromosome 9 open reading frame 91 | | -2.23 | 0.01765 |
| 235706_at | *CPM* | | | carboxypeptidase M | | -2.23 | 0.01793 |
| 201855_s_at | *ATMIN* | | | ATM interactor | | -2.23 | 0.01819 |
| 1555562_a_at | *ZCCHC7* | | | zinc finger, CCHC domain containing 7 | | -2.23 | 0.02114 |
| 222843_at | *FIGNL1* | | | fidgetin-like 1 | | -2.23 | 0.02136 |
| 205234_at | *SLC16A4* | | | solute carrier family 16, member 4 (monocarboxylic acid transporter 5) | | -2.23 | 0.0248 |
| 205132_at | *ACTC1* | | | actin, alpha, cardiac muscle 1 | | -2.23 | 0.02565 |
| 228069_at | *MTFR2* | | | mitochondrial fission regulator 2 | | -2.23 | 0.02672 |
| 231130_at | *FKBP7* | | | FK506 binding protein 7 | | -2.23 | 0.02877 |
| 239578_at |  | | |  | | -2.23 | 0.03082 |
| 230352_at | *PRPS2* | | | phosphoribosyl pyrophosphate synthetase 2 | | -2.23 | 0.03178 |
| 201237_at | *CAPZA2* | | | capping protein (actin filament) muscle Z-line, alpha 2 | | -2.23 | 0.03202 |
| 241260_at |  | | |  | | -2.23 | 0.03558 |
| 235518_at | *SLC8A1* | | | solute carrier family 8 (sodium/calcium exchanger), member 1 | | -2.23 | 0.03605 |
| 237512_at | *NAV2-AS2* | | | NAV2 antisense RNA 2; NULL | | -2.23 | 0.03828 |
| 226083_at | *TMEM70* | | | transmembrane protein 70 | | -2.23 | 0.03884 |
| 202843_at | *DNAJB9* | | | DnaJ (Hsp40) homolog, subfamily B, member 9 | | -2.23 | 0.04858 |
| 226041_at | *NAPEPLD* | | | N-acyl phosphatidylethanolamine phospholipase D | | -2.23 | 0.04884 |
| 235223_at | *RRP36* | | | ribosomal RNA processing 36 homolog (S. cerevisiae) | | -2.24 | 0.00068 |
| 1562253_at | *SLC7A11-AS1* | | | SLC7A11 antisense RNA 1 | | -2.24 | 0.00072 |
| 239630_at |  | | |  | | -2.24 | 0.00083 |
| 226163_at | *ZBTB9* | | | zinc finger and BTB domain containing 9 | | -2.24 | 0.00092 |
| 203374_s_at | *TPP2* | | | tripeptidyl peptidase II | | -2.24 | 0.00119 |
| 234464_s_at | *EME1* | | | essential meiotic endonuclease 1 homolog 1 (S. pombe) | | -2.24 | 0.00189 |
| 202114_at | *SNX2* | | | sorting nexin 2 | | -2.24 | 0.00312 |
| 225975_at | *PCDH18* | | | protocadherin 18 | | -2.24 | 0.00338 |
| 226521_s_at | *FAM175A* | | | family with sequence similarity 175, member A | | -2.24 | 0.00361 |
| 242898_at | *EIF2AK2* | | | eukaryotic translation initiation factor 2-alpha kinase 2 | | -2.24 | 0.00365 |
| 221038_at |  | | |  | | -2.24 | 0.00404 |
| 212310_at | *MIA3* | | | melanoma inhibitory activity family, member 3 | | -2.24 | 0.00428 |
| 227375_at | *ANKRD13C* | | | ankyrin repeat domain 13C | | -2.24 | 0.00456 |
| 212221_x_at | *IDS* | | | iduronate 2-sulfatase | | -2.24 | 0.00456 |
| 1568781_at |  | | |  | | -2.24 | 0.0051 |
| 225180_at | *TTC14* | | | tetratricopeptide repeat domain 14 | | -2.24 | 0.00578 |
| 244334_at | *TRAM1L1* | | | translocation associated membrane protein 1-like 1 | | -2.24 | 0.00635 |
| 201991_s_at | *KIF5B* | | | kinesin family member 5B | | -2.24 | 0.00677 |
| 241775_at |  | | |  | | -2.24 | 0.00686 |
| 222894_x_at | *NDUFAF5* | | | NADH dehydrogenase (ubiquinone) complex I, assembly factor 5 | | -2.24 | 0.00707 |
| 212887_at | *SEC23A* | | | Sec23 homolog A (S. cerevisiae) | | -2.24 | 0.0071 |
| 226556_at | *MAP3K13* | | | mitogen-activated protein kinase kinase kinase 13 | | -2.24 | 0.00731 |
| 225976_at | *BTF3L4* | | | basic transcription factor 3-like 4 | | -2.24 | 0.00766 |
| 227708_at | *EEF1A1* | | | eukaryotic translation elongation factor 1 alpha 1 | | -2.24 | 0.00784 |
| 224700_at | *STT3B* | | | STT3B, subunit of the oligosaccharyltransferase complex (catalytic) | | -2.24 | 0.00811 |
| 217813_s_at | *SPIN1* | | | spindlin 1 | | -2.24 | 0.00825 |
| 201626_at | *INSIG1* | | | insulin induced gene 1 | | -2.24 | 0.00934 |
| 229751_s_at | *PUS7L* | | | pseudouridylate synthase 7 homolog (S. cerevisiae)-like | | -2.24 | 0.01183 |
| 224786_at | *SCOC* | | | short coiled-coil protein | | -2.24 | 0.0129 |
| 219334_s_at | *NABP1* | | | nucleic acid binding protein 1 | | -2.24 | 0.01403 |
| 218067_s_at | *ARGLU1* | | | arginine and glutamate rich 1 | | -2.24 | 0.01617 |
| 219917_at | *ZCCHC4* | | | zinc finger, CCHC domain containing 4 | | -2.24 | 0.0169 |
| 220083_x_at | *UCHL5* | | | ubiquitin carboxyl-terminal hydrolase L5 | | -2.24 | 0.01995 |
| 1570622_at |  | | |  | | -2.24 | 0.0214 |
| 221516_s_at | *SMCR7L* | | | Smith-Magenis syndrome chromosome region, candidate 7-like | | -2.25 | 9.8x10^-5^ |
| 225300_at | *KNSTRN* | | | kinetochore-localized astrin/SPAG5 binding protein | | -2.25 | 0.00011 |
| 201243_s_at | *ATP1B1* | | | ATPase, Na+/K+ transporting, beta 1 polypeptide | | -2.25 | 0.00109 |
| 213702_x_at | *ASAH1* | | | N-acylsphingosine amidohydrolase (acid ceramidase) 1 | | -2.25 | 0.00133 |
| 239768_x_at | *GPATCH2* | | | G patch domain containing 2 | | -2.25 | 0.00137 |
| 225331_at | *CCDC50* | | | coiled-coil domain containing 50 | | -2.25 | 0.00141 |
| 224994_at | *CAMK2D* | | | calcium/calmodulin-dependent protein kinase II delta | | -2.25 | 0.00141 |
| 239231_at | *ZNF101* | | | zinc finger protein 101 | | -2.25 | 0.00172 |
| 204759_at | *RCBTB2* | | | regulator of chromosome condensation (RCC1) and BTB (POZ) domain containing protein 2 | | -2.25 | 0.00186 |
| 225551_at | *CNST* | | | consortin, connexin sorting protein | | -2.25 | 0.0021 |
| 226269_at | *GDAP1* | | | ganglioside induced differentiation associated protein 1 | | -2.25 | 0.00212 |
| 211744_s_at | *CD58* | | | CD58 molecule | | -2.25 | 0.00263 |
| 212957_s_at |  | | |  | | -2.25 | 0.00274 |
| 227931_at | *INO80D* | | | INO80 complex subunit D | | -2.25 | 0.00287 |
| 205835_s_at | *YTHDC2* | | | YTH domain containing 2 | | -2.25 | 0.00294 |
| 217600_at | *SCUBE3* | | | signal peptide, CUB domain, EGF-like 3 | | -2.25 | 0.00317 |
| 201153_s_at | *MBNL1* | | | muscleblind-like splicing regulator 1 | | -2.25 | 0.00331 |
| 219555_s_at | *CENPN* | | | centromere protein N | | -2.25 | 0.00368 |
| 202396_at | *TCERG1* | | | transcription elongation regulator 1 | | -2.25 | 0.00392 |
| 201302_at | *ANXA4* | | | annexin A4 | | -2.25 | 0.00414 |
| 202691_at | *SNRPD1* | | | small nuclear ribonucleoprotein D1 polypeptide 16kDa | | -2.25 | 0.00426 |
| 220820_at |  | | |  | | -2.25 | 0.00432 |
| 1559121_s_at |  | | |  | | -2.25 | 0.00433 |
| 235457_at | *MAML2* | | | mastermind-like 2 (Drosophila) | | -2.25 | 0.00442 |
| 1553928_at | *ELMOD2* | | | ELMO/CED-12 domain containing 2 | | -2.25 | 0.00506 |
| 243540_at |  | | |  | | -2.25 | 0.00511 |
| 228992_at | *MED28* | | | mediator complex subunit 28 | | -2.25 | 0.00581 |
| 217544_at |  | | |  | | -2.25 | 0.00583 |
| 1554883_a_at | *ERCC8* | | | excision repair cross-complementing rodent repair deficiency, complementation group 8 | | -2.25 | 0.00607 |
| 225895_at | *SYNPO2* | | | synaptopodin 2 | | -2.25 | 0.00708 |
| 201733_at | *CLCN3* | | | chloride channel, voltage-sensitive 3 | | -2.25 | 0.00901 |
| 232618_at | *TXLNG2P* | | | taxilin gamma 2, pseudogene | | -2.25 | 0.01007 |
| 208715_at | *TMCO1* | | | transmembrane and coiled-coil domains 1 | | -2.25 | 0.01032 |
| 205340_at | *ZBTB24* | | | zinc finger and BTB domain containing 24 | | -2.25 | 0.01083 |
| 224250_s_at | *SECISBP2* | | | SECIS binding protein 2 | | -2.25 | 0.01388 |
| 53202_at | *C7orf25* | | | chromosome 7 open reading frame 25; proteasome (prosome, macropain) subunit, alpha type, 2 | | -2.25 | 0.01421 |
| 202606_s_at | *TLK1* | | | tousled-like kinase 1 | | -2.25 | 0.01443 |
| 227935_s_at | *PCGF5* | | | polycomb group ring finger 5 | | -2.25 | 0.01775 |
| 229588_at | *DNAJC10* | | | DnaJ (Hsp40) homolog, subfamily C, member 10 | | -2.25 | 0.01878 |
| 234488_s_at | *GMCL1* | | | germ cell-less, spermatogenesis associated 1; germ cell-less, spermatogenesis associated 1 pseudogene 1 | | -2.25 | 0.01879 |
| 200037_s_at | *CBX3* | | | chromobox homolog 3 | | -2.25 | 0.02039 |
| 225691_at | *CDK12* | | | cyclin-dependent kinase 12 | | -2.25 | 0.02264 |
| 235170_at | *ZNF92* | | | zinc finger protein 92 | | -2.25 | 0.02321 |
| 204435_at | *NUPL1* | | | nucleoporin like 1 | | -2.25 | 0.02379 |
| 219487_at | *BBS10* | | | Bardet-Biedl syndrome 10 | | -2.25 | 0.0345 |
| 235061_at | *PPM1K* | | | protein phosphatase, Mg2+/Mn2+ dependent, 1K | | -2.25 | 0.0355 |
| 1555137_a_at | *FGD6* | | | FYVE, RhoGEF and PH domain containing 6 | | -2.25 | 0.03933 |
| 227988_s_at | *VPS13A* | | | vacuolar protein sorting 13 homolog A (S. cerevisiae) | | -2.25 | 0.04473 |
| 224831_at | *CPEB4* | | | cytoplasmic polyadenylation element binding protein 4 | | -2.25 | 0.04545 |
| 224632_at | *GPATCH4* | | | G patch domain containing 4 | | -2.26 | 2.4x10^-5^ |
| 223481_s_at | *MRPL47* | | | mitochondrial ribosomal protein L47 | | -2.26 | 0.00003 |
| 204059_s_at | *ME1* | | | malic enzyme 1, NADP(+)-dependent, cytosolic | | -2.26 | 0.00011 |
| 221540_x_at | *GTF2H2* | | | general transcription factor IIH, polypeptide 2, 44kDa | | -2.26 | 0.00022 |
| 1566166_at |  | | |  | | -2.26 | 0.0007 |
| 226688_at | *TCAIM* | | | T cell activation inhibitor, mitochondrial | | -2.26 | 0.00076 |
| 222793_at | *DDX58* | | | DEAD (Asp-Glu-Ala-Asp) box polypeptide 58 | | -2.26 | 0.00103 |
| 219022_at | *C12orf43* | | | chromosome 12 open reading frame 43 | | -2.26 | 0.00277 |
| 239488_at | *PPM1L* | | | protein phosphatase, Mg2+/Mn2+ dependent, 1L | | -2.26 | 0.00281 |
| 234541_s_at | *ARHGEF12* | | | Rho guanine nucleotide exchange factor (GEF) 12 | | -2.26 | 0.00308 |
| 1554292_a_at | *UHRF1BP1L* | | | UHRF1 binding protein 1-like | | -2.26 | 0.00321 |
| 203896_s_at | *PLCB4* | | | phospholipase C, beta 4 | | -2.26 | 0.00385 |
| 222980_at | *RAB10* | | | RAB10, member RAS oncogene family | | -2.26 | 0.00416 |
| 211749_s_at | *VAMP3* | | | vesicle-associated membrane protein 3 | | -2.26 | 0.00506 |
| 224581_s_at | *NUCKS1* | | | nuclear casein kinase and cyclin-dependent kinase substrate 1 | | -2.26 | 0.00642 |
| 238762_at | *MTHFD2L* | | | methylenetetrahydrofolate dehydrogenase (NADP+ dependent) 2-like | | -2.26 | 0.00659 |
| 218656_s_at | *LHFP* | | | lipoma HMGIC fusion partner | | -2.26 | 0.00666 |
| 230724_s_at | *C11orf57* | | | chromosome 11 open reading frame 57 | | -2.26 | 0.00729 |
| 203007_x_at | *LYPLA1* | | | lysophospholipase I | | -2.26 | 0.00749 |
| 1564053_a_at | *YTHDF3* | | | YTH domain family, member 3 | | -2.26 | 0.00883 |
| 210211_s_at | *HSP90AA1* | | | heat shock protein 90kDa alpha (cytosolic), class A member 1 | | -2.26 | 0.00954 |
| 207590_s_at | *CENPI* | | | centromere protein I | | -2.26 | 0.00971 |
| 208273_at | *ZNF670* | | | zinc finger protein 670; ZNF670-ZNF695 readthrough; zinc finger protein 695 | | -2.26 | 0.01075 |
| 203968_s_at | *CDC6* | | | cell division cycle 6 | | -2.26 | 0.01136 |
| 223511_at | *SPRTN* | | | SprT-like N-terminal domain | | -2.26 | 0.0126 |
| 210878_s_at | *KDM3B* | | | lysine (K)-specific demethylase 3B | | -2.26 | 0.01484 |
| 211038_s_at | *CROCCP2* | | | ciliary rootlet coiled-coil, rootletin pseudogene 2 | | -2.26 | 0.01507 |
| 237864_at |  | | |  | | -2.26 | 0.01534 |
| 1554455_at | *LINS* | | | lines homolog (Drosophila) | | -2.26 | 0.01742 |
| 231098_at |  | | |  | | -2.26 | 0.01903 |
| 1566084_at |  | | |  | | -2.26 | 0.02203 |
| 230082_at |  | | |  | | -2.26 | 0.02316 |
| 225702_at | *C8orf76* | | | chromosome 8 open reading frame 76 | | -2.26 | 0.02566 |
| 242170_at |  | | |  | | -2.26 | 0.03265 |
| 213017_at | *ABHD3* | | | abhydrolase domain containing 3 | | -2.26 | 0.04368 |
| 224523_s_at | *CMSS1* | | | cms1 ribosomal small subunit homolog (yeast) | | -2.27 | 4.7x10^-5^ |
| 211375_s_at | *ILF3* | | | interleukin enhancer binding factor 3, 90kDa | | -2.27 | 0.00042 |
| 235511_at |  | | |  | | -2.27 | 0.00046 |
| 226384_at | *PPAPDC1B* | | | phosphatidic acid phosphatase type 2 domain containing 1B | | -2.27 | 0.00046 |
| 202289_s_at | *TACC2* | | | transforming, acidic coiled-coil containing protein 2 | | -2.27 | 0.00061 |
| 235003_at | *UHMK1* | | | U2AF homology motif (UHM) kinase 1 | | -2.27 | 0.00075 |
| 209003_at | *SLC25A11* | | | solute carrier family 25 (mitochondrial carrier; oxoglutarate carrier), member 11 | | -2.27 | 0.0008 |
| 201381_x_at | *CACYBP* | | | calcyclin binding protein | | -2.27 | 0.00105 |
| 204517_at | *PPIC* | | | peptidylprolyl isomerase C (cyclophilin C) | | -2.27 | 0.00132 |
| 207012_at | *MMP16* | | | matrix metallopeptidase 16 (membrane-inserted) | | -2.27 | 0.00135 |
| 229501_s_at | *USP8* | | | ubiquitin specific peptidase 8 | | -2.27 | 0.0015 |
| 233042_at |  | | |  | | -2.27 | 0.00151 |
| 222399_s_at | *TM9SF3* | | | transmembrane 9 superfamily member 3 | | -2.27 | 0.00209 |
| 209939_x_at | *CFLAR* | | | CASP8 and FADD-like apoptosis regulator | | -2.27 | 0.00234 |
| 201317_s_at | *PSMA2* | | | proteasome (prosome, macropain) subunit, alpha type, 2 | | -2.27 | 0.00305 |
| 224662_at | *KIF5B* | | | kinesin family member 5B | | -2.27 | 0.00325 |
| 214739_at | *LRCH3* | | | leucine-rich repeats and calponin homology (CH) domain containing 3 | | -2.27 | 0.00338 |
| 215719_x_at | *FAS* | | | Fas cell surface death receptor | | -2.27 | 0.00344 |
| 226750_at | *LARP1B* | | | La ribonucleoprotein domain family, member 1B | | -2.27 | 0.00344 |
| 239843_at | *RIT1* | | | Ras-like without CAAX 1 | | -2.27 | 0.00377 |
| 233999_s_at | *TTC26* | | | tetratricopeptide repeat domain 26 | | -2.27 | 0.00485 |
| 222752_s_at | *TMEM206* | | | transmembrane protein 206 | | -2.27 | 0.0049 |
| 209766_at | *PRDX3* | | | peroxiredoxin 3 | | -2.27 | 0.00592 |
| 207071_s_at | *ACO1* | | | aconitase 1, soluble | | -2.27 | 0.00657 |
| 219886_at | *CEP97* | | | centrosomal protein 97kDa | | -2.27 | 0.0073 |
| 212850_s_at | *LRP4* | | | low density lipoprotein receptor-related protein 4 | | -2.27 | 0.00748 |
| 201811_x_at | *SH3BP5* | | | SH3-domain binding protein 5 (BTK-associated) | | -2.27 | 0.00771 |
| 225017_at | *CCDC14* | | | coiled-coil domain containing 14 | | -2.27 | 0.00786 |
| 209657_s_at | *HSF2* | | | heat shock transcription factor 2 | | -2.27 | 0.00799 |
| 221482_s_at | *ARPP19* | | | cAMP-regulated phosphoprotein, 19kDa | | -2.27 | 0.00802 |
| 225456_at | *MED1* | | | mediator complex subunit 1 | | -2.27 | 0.00875 |
| 238660_at | *WDFY3* | | | WD repeat and FYVE domain containing 3 | | -2.27 | 0.00936 |
| 209337_at | *PSIP1* | | | PC4 and SFRS1 interacting protein 1 | | -2.27 | 0.01029 |
| 213222_at | *PLCB1* | | | phospholipase C, beta 1 (phosphoinositide-specific) | | -2.27 | 0.0119 |
| 218244_at | *NOL8* | | | nucleolar protein 8 | | -2.27 | 0.01279 |
| 1555978_s_at | *MYL12A* | | | myosin, light chain 12A, regulatory, non-sarcomeric | | -2.27 | 0.01364 |
| 1557302_at | *ZNF585B* | | | zinc finger protein 585B | | -2.27 | 0.01426 |
| 219875_s_at | *DESI2* | | | desumoylating isopeptidase 2 | | -2.27 | 0.01895 |
| 200914_x_at | *KTN1* | | | kinectin 1 (kinesin receptor) | | -2.27 | 0.01955 |
| 202362_at | *RAP1A* | | | RAP1A, member of RAS oncogene family | | -2.27 | 0.02304 |
| 208407_s_at | *CTNND1* | | | catenin (cadherin-associated protein), delta 1; TMX2-CTNND1 readthrough (NMD candidate) | | -2.27 | 0.02326 |
| 201445_at | *CNN3* | | | calponin 3, acidic | | -2.27 | 0.02549 |
| 224802_at | *NDFIP2* | | | Nedd4 family interacting protein 2 | | -2.27 | 0.03817 |
| 238410_x_at |  | | |  | | -2.28 | 0.0003 |
| 210621_s_at | *RASA1* | | | RAS p21 protein activator (GTPase activating protein) 1 | | -2.28 | 0.00065 |
| 226603_at | *SAMD9L* | | | sterile alpha motif domain containing 9-like | | -2.28 | 0.00111 |
| 205521_at | *EXOG* | | | endo/exonuclease (5'-3'), endonuclease G-like | | -2.28 | 0.00128 |
| 235768_at | *SH3RF2* | | | SH3 domain containing ring finger 2 | | -2.28 | 0.00175 |
| 1561033_at |  | | |  | | -2.28 | 0.00221 |
| 233472_at | *TCP11L1* | | | t-complex 11, testis-specific-like 1 | | -2.28 | 0.00252 |
| 211971_s_at | *LRPPRC* | | | leucine-rich pentatricopeptide repeat containing | | -2.28 | 0.00308 |
| 229371_at |  | | |  | | -2.28 | 0.00397 |
| 208103_s_at | *ANP32E* | | | acidic (leucine-rich) nuclear phosphoprotein 32 family, member E | | -2.28 | 0.00436 |
| 230775_s_at | *SPG20* | | | spastic paraplegia 20 (Troyer syndrome) | | -2.28 | 0.00516 |
| 1553315_at | *SLFNL1* | | | schlafen-like 1 | | -2.28 | 0.00526 |
| 201653_at | *CNIH* | | | cornichon homolog (Drosophila) | | -2.28 | 0.00654 |
| 203465_at | *MRPL19* | | | mitochondrial ribosomal protein L19 | | -2.28 | 0.00751 |
| 226511_at | *DCAF10* | | | DDB1 and CUL4 associated factor 10 | | -2.28 | 0.00847 |
| 229235_at | *NFATC2IP* | | | nuclear factor of activated T-cells, cytoplasmic, calcineurin-dependent 2 interacting protein | | -2.28 | 0.00931 |
| 212536_at | *ATP11B* | | | ATPase, class VI, type 11B | | -2.28 | 0.01228 |
| 203324_s_at | *CAV2* | | | caveolin 2 | | -2.28 | 0.01236 |
| 211069_s_at | *SUMO1* | | | small ubiquitin-like modifier 1 | | -2.28 | 0.01499 |
| 1566984_at |  | | |  | | -2.28 | 0.01512 |
| 209297_at | *ITSN1* | | | intersectin 1 (SH3 domain protein) | | -2.28 | 0.01599 |
| 222465_at | *RSL24D1* | | | ribosomal L24 domain containing 1 | | -2.28 | 0.01601 |
| 235879_at |  | | |  | | -2.28 | 0.0174 |
| 235520_at | *ZNF280C* | | | zinc finger protein 280C | | -2.28 | 0.01744 |
| 226381_at |  | | |  | | -2.28 | 0.01971 |
| 1556216_s_at |  | | |  | | -2.28 | 0.02356 |
| 235078_at |  | | |  | | -2.28 | 0.02563 |
| 217445_s_at | *GART* | | | phosphoribosylglycinamide formyltransferase, phosphoribosylglycinamide synthetase, phosphoribosylaminoimidazole synthetase | | -2.28 | 0.02604 |
| 1569025_s_at | *FAM13A* | | | family with sequence similarity 13, member A | | -2.28 | 0.02608 |
| 225501_at | *PHF6* | | | PHD finger protein 6 | | -2.28 | 0.03102 |
| 209717_at | *EVI5* | | | ecotropic viral integration site 5 | | -2.28 | 0.03188 |
| 218259_at | *MKL2* | | | MKL/myocardin-like 2 | | -2.28 | 0.03471 |
| 213404_s_at | *RHEB* | | | Ras homolog enriched in brain | | -2.28 | 0.04365 |
| 220721_at | *ZNF614* | | | zinc finger protein 614 | | -2.28 | 0.04436 |
| 225279_s_at | *C3orf17* | | | chromosome 3 open reading frame 17 | | -2.28 | 0.04578 |
| 228937_at | *LACC1* | | | laccase (multicopper oxidoreductase) domain containing 1 | | -2.28 | 0.04909 |
| 226771_at | *ATP8B2* | | | ATPase, aminophospholipid transporter, class I, type 8B, member 2 | | -2.29 | 3.5x10^-5^ |
| 203947_at | *CSTF3* | | | cleavage stimulation factor, 3' pre-RNA, subunit 3, 77kDa | | -2.29 | 0.00017 |
| 238542_at | *ULBP2* | | | UL16 binding protein 2 | | -2.29 | 0.00053 |
| 209712_at | *SLC35D1* | | | solute carrier family 35 (UDP-glucuronic acid/UDP-N-acetylgalactosamine dual transporter), member D1 | | -2.29 | 0.00096 |
| 225260_s_at | *MRPL32* | | | mitochondrial ribosomal protein L32 | | -2.29 | 0.00117 |
| 1566482_at |  | | |  | | -2.29 | 0.00216 |
| 1556060_a_at | *ZNF451* | | | zinc finger protein 451 | | -2.29 | 0.00226 |
| 236196_at |  | | |  | | -2.29 | 0.0032 |
| 229097_at | *DIAPH3* | | | diaphanous homolog 3 (Drosophila) | | -2.29 | 0.00323 |
| 209841_s_at | *LRRN3* | | | leucine rich repeat neuronal 3 | | -2.29 | 0.00342 |
| 201118_at | *PGD* | | | phosphogluconate dehydrogenase | | -2.29 | 0.00412 |
| 223754_at | *C2orf88* | | | chromosome 2 open reading frame 88 | | -2.29 | 0.00524 |
| 201925_s_at | *CD55* | | | CD55 molecule, decay accelerating factor for complement (Cromer blood group) | | -2.29 | 0.00544 |
| 226391_at |  | | |  | | -2.29 | 0.00578 |
| 224978_s_at | *USP36* | | | ubiquitin specific peptidase 36 | | -2.29 | 0.00696 |
| 226341_at |  | | |  | | -2.29 | 0.0085 |
| 239447_at | *TRA2B* | | | transformer 2 beta homolog (Drosophila) | | -2.29 | 0.00956 |
| 225988_at | *HERC4* | | | HECT and RLD domain containing E3 ubiquitin protein ligase 4 | | -2.29 | 0.01029 |
| 229354_at | *AHRR* | | | aryl-hydrocarbon receptor repressor | | -2.29 | 0.01096 |
| 209967_s_at | *CREM* | | | cAMP responsive element modulator | | -2.29 | 0.01379 |
| 204422_s_at | *FGF2* | | | fibroblast growth factor 2 (basic) | | -2.29 | 0.01565 |
| 212398_at | *RDX* | | | radixin | | -2.29 | 0.01625 |
| 244324_at | *C18orf54* | | | chromosome 18 open reading frame 54 | | -2.29 | 0.01824 |
| 205990_s_at | *WNT5A* | | | wingless-type MMTV integration site family, member 5A | | -2.29 | 0.01838 |
| 230260_s_at |  | | |  | | -2.29 | 0.02092 |
| 200726_at | *PPP1CC* | | | protein phosphatase 1, catalytic subunit, gamma isozyme | | -2.29 | 0.0221 |
| 212628_at | *PKN2* | | | protein kinase N2 | | -2.29 | 0.02378 |
| 212435_at | *TRIM33* | | | tripartite motif containing 33 | | -2.29 | 0.02397 |
| 209704_at | *MTF2* | | | metal response element binding transcription factor 2 | | -2.29 | 0.025 |
| 227921_at |  | | |  | | -2.29 | 0.02559 |
| 213572_s_at | *SERPINB1* | | | serpin peptidase inhibitor, clade B (ovalbumin), member 1 | | -2.29 | 0.02822 |
| 202146_at | *IFRD1* | | | interferon-related developmental regulator 1 | | -2.29 | 0.02985 |
| 226366_at | *SHPRH* | | | SNF2 histone linker PHD RING helicase, E3 ubiquitin protein ligase | | -2.29 | 0.03296 |
| 238940_at | *KLF12* | | | Kruppel-like factor 12 | | -2.29 | 0.03706 |
| 1559966_a_at | *ZFHX4-AS1* | | | ZFHX4 antisense RNA 1 | | -2.29 | 0.03767 |
| 218109_s_at | *MFSD1* | | | major facilitator superfamily domain containing 1 | | -2.3 | 0.00106 |
| 230400_s_at |  | | |  | | -2.3 | 0.00144 |
| 226400_at | *CDC42* | | | cell division cycle 42 | | -2.3 | 0.00156 |
| 223707_at | *RPL27A* | | | ribosomal protein L27a; small nucleolar RNA, H/ACA box 3 | | -2.3 | 0.00272 |
| 224840_at | *FKBP5* | | | FK506 binding protein 5 | | -2.3 | 0.00286 |
| 225640_at | *LOC100506710* | | | endogenous Bornavirus-like nucleoprotein 2 pseudogene | | -2.3 | 0.00369 |
| 202759_s_at | *AKAP2* | | | A kinase (PRKA) anchor protein 2; PALM2-AKAP2 readthrough | | -2.3 | 0.00374 |
| 200945_s_at | *SEC31A* | | | SEC31 homolog A (S. cerevisiae) | | -2.3 | 0.00435 |
| 214649_s_at | *MTMR2* | | | myotubularin related protein 2 | | -2.3 | 0.00475 |
| 213599_at | *OIP5* | | | Opa interacting protein 5 | | -2.3 | 0.00496 |
| 200057_s_at | *NONO* | | | non-POU domain containing, octamer-binding | | -2.3 | 0.00557 |
| 225578_at | *MZT1* | | | mitotic spindle organizing protein 1 | | -2.3 | 0.0059 |
| 226782_at | *SLC25A30* | | | solute carrier family 25, member 30 | | -2.3 | 0.00606 |
| 203835_at | *LRRC32* | | | leucine rich repeat containing 32 | | -2.3 | 0.00729 |
| 222654_at | *IMPAD1* | | | inositol monophosphatase domain containing 1 | | -2.3 | 0.0076 |
| 211090_s_at | *PRPF4B* | | | PRP4 pre-mRNA processing factor 4 homolog B (yeast) | | -2.3 | 0.00824 |
| 216966_at | *ITGA2B* | | | integrin, alpha 2b (platelet glycoprotein IIb of IIb/IIIa complex, antigen CD41) | | -2.3 | 0.00874 |
| 40612_at | *DOPEY1* | | | dopey family member 1 | | -2.3 | 0.00958 |
| 203883_s_at | *RAB11FIP2* | | | RAB11 family interacting protein 2 (class I) | | -2.3 | 0.01042 |
| 225589_at | *SH3RF1* | | | SH3 domain containing ring finger 1 | | -2.3 | 0.01104 |
| 223675_s_at | *VEZT* | | | vezatin, adherens junctions transmembrane protein | | -2.3 | 0.01152 |
| 232238_at | *ASPM* | | | asp (abnormal spindle) homolog, microcephaly associated (Drosophila) | | -2.3 | 0.01181 |
| 1566472_s_at | *LOC100996506* | | | all-trans-retinol 13,14-reductase-like; retinol saturase (all-trans-retinol 13,14-reductase) | | -2.3 | 0.01549 |
| 204841_s_at | *EEA1* | | | early endosome antigen 1 | | -2.3 | 0.0162 |
| 230875_s_at | *ATP11A* | | | ATPase, class VI, type 11A | | -2.3 | 0.02948 |
| 225774_at | *RSPRY1* | | | ring finger and SPRY domain containing 1 | | -2.3 | 0.02968 |
| 1556622_s_at |  | | |  | | -2.3 | 0.03317 |
| 234854_at | *MDN1* | | | MDN1, midasin homolog (yeast) | | -2.31 | 3.9x10^-5^ |
| 220941_s_at | *C21orf91* | | | chromosome 21 open reading frame 91 | | -2.31 | 6.9x10^-5^ |
| 222262_s_at | *ETNK1* | | | ethanolamine kinase 1 | | -2.31 | 0.00074 |
| 225424_at | *GPAM* | | | glycerol-3-phosphate acyltransferase, mitochondrial | | -2.31 | 0.00085 |
| 214313_s_at | *EIF5B* | | | eukaryotic translation initiation factor 5B | | -2.31 | 0.00086 |
| 236918_s_at | *LRRC34* | | | leucine rich repeat containing 34 | | -2.31 | 0.00091 |
| 212246_at | *MCFD2* | | | multiple coagulation factor deficiency 2 | | -2.31 | 0.00121 |
| 201209_at | *HDAC1* | | | histone deacetylase 1 | | -2.31 | 0.00126 |
| 231815_at | *PHF12* | | | PHD finger protein 12 | | -2.31 | 0.0019 |
| 234111_at |  | | |  | | -2.31 | 0.00337 |
| 240204_at | *SNRPN* | | | small nuclear ribonucleoprotein polypeptide N | | -2.31 | 0.00389 |
| 242654_at | *FANCC* | | | Fanconi anemia, complementation group C | | -2.31 | 0.00435 |
| 240172_at | *ERGIC2* | | | ERGIC and golgi 2 | | -2.31 | 0.00479 |
| 209786_at | *HMGN4* | | | high mobility group nucleosomal binding domain 4 | | -2.31 | 0.00524 |
| 222039_at | *KIF18B* | | | kinesin family member 18B | | -2.31 | 0.00538 |
| 226449_at | *CEP120* | | | centrosomal protein 120kDa | | -2.31 | 0.00548 |
| 219885_at | *SLFN12* | | | schlafen family member 12 | | -2.31 | 0.00557 |
| 209362_at | *MED21* | | | mediator complex subunit 21 | | -2.31 | 0.00649 |
| 229558_at | *KNOP1* | | | lysine-rich nucleolar protein 1 | | -2.31 | 0.00824 |
| 218549_s_at | *RMDN1* | | | regulator of microtubule dynamics 1 | | -2.31 | 0.00863 |
| 203736_s_at | *PPFIBP1* | | | PTPRF interacting protein, binding protein 1 (liprin beta 1) | | -2.31 | 0.00889 |
| 209909_s_at | *TGFB2* | | | transforming growth factor, beta 2 | | -2.31 | 0.00909 |
| 223758_s_at | *GTF2H2* | | | general transcription factor IIH, polypeptide 2, 44kDa | | -2.31 | 0.00911 |
| 238510_at | *ZNF720* | | | zinc finger protein 720 | | -2.31 | 0.00956 |
| 1554640_at | *PALM2* | | | paralemmin 2 | | -2.31 | 0.00964 |
| 211063_s_at | *NCK1* | | | NCK adaptor protein 1 | | -2.31 | 0.01105 |
| 209388_at | *PAPOLA* | | | poly(A) polymerase alpha | | -2.31 | 0.01262 |
| 222275_at | *MRPS30* | | | mitochondrial ribosomal protein S30 | | -2.31 | 0.01331 |
| 232681_at |  | | |  | | -2.31 | 0.01552 |
| 225612_s_at | *B3GNT5* | | | UDP-GlcNAc:betaGal beta-1,3-N-acetylglucosaminyltransferase 5 | | -2.31 | 0.02168 |
| 224754_at | *SP1* | | | Sp1 transcription factor | | -2.31 | 0.02169 |
| 217862_at | *PIAS1* | | | protein inhibitor of activated STAT, 1 | | -2.31 | 0.02401 |
| 202653_s_at | *Mar-07* | | | membrane-associated ring finger (C3HC4) 7, E3 ubiquitin protein ligase | | -2.31 | 0.02487 |
| 228357_at | *UNK* | | | unkempt homolog (Drosophila) | | -2.31 | 0.02602 |
| 207147_at | *DLX2* | | | distal-less homeobox 2 | | -2.31 | 0.03273 |
| 226321_at | *LYSMD3* | | | LysM, putative peptidoglycan-binding, domain containing 3 | | -2.31 | 0.04214 |
| 225707_at | *ARL6IP6* | | | ADP-ribosylation-like factor 6 interacting protein 6 | | -2.31 | 0.04604 |
| 212637_s_at | *WWP1* | | | WW domain containing E3 ubiquitin protein ligase 1 | | -2.31 | 0.0481 |
| 218087_s_at | *SORBS1* | | | sorbin and SH3 domain containing 1 | | -2.31 | 0.04873 |
| 203831_at | *R3HDM2* | | | R3H domain containing 2 | | -2.31 | 0.04944 |
| 229070_at | *ADTRP* | | | androgen-dependent TFPI-regulating protein | | -2.32 | 2.4x10^-5^ |
| 232298_at | *MBNL1-AS1* | | | MBNL1 antisense RNA 1 | | -2.32 | 0.00027 |
| 203695_s_at | *DFNA5* | | | deafness, autosomal dominant 5 | | -2.32 | 0.00093 |
| 204093_at | *CCNH* | | | cyclin H | | -2.32 | 0.00126 |
| 225019_at | *CAMK2D* | | | calcium/calmodulin-dependent protein kinase II delta | | -2.32 | 0.00155 |
| 1554029_a_at | *TTC37* | | | tetratricopeptide repeat domain 37 | | -2.32 | 0.00189 |
| 1563351_at |  | | |  | | -2.32 | 0.00219 |
| 202026_at | *SDHD* | | | succinate dehydrogenase complex, subunit D, integral membrane protein | | -2.32 | 0.00257 |
| 228390_at | *RAB30* | | | RAB30, member RAS oncogene family | | -2.32 | 0.00315 |
| 203351_s_at | *ORC4* | | | origin recognition complex, subunit 4 | | -2.32 | 0.00442 |
| 212990_at | *SYNJ1* | | | synaptojanin 1 | | -2.32 | 0.00498 |
| 225210_s_at | *FAM103A1* | | | family with sequence similarity 103, member A1 | | -2.32 | 0.00842 |
| 211758_x_at | *TXNDC9* | | | thioredoxin domain containing 9 | | -2.32 | 0.00893 |
| 236202_at |  | | |  | | -2.32 | 0.00899 |
| 208986_at | *TCF12* | | | transcription factor 12 | | -2.32 | 0.00982 |
| 208654_s_at | *CD164* | | | CD164 molecule, sialomucin | | -2.32 | 0.01281 |
| 239848_at | *EIF3M* | | | eukaryotic translation initiation factor 3, subunit M | | -2.32 | 0.01715 |
| 227012_at | *SLC25A40* | | | solute carrier family 25, member 40 | | -2.32 | 0.02009 |
| 1552862_at | *RUSC1-AS1* | | | RUSC1 antisense RNA 1 | | -2.32 | 0.02255 |
| 200934_at | *DEK* | | | DEK oncogene | | -2.32 | 0.0227 |
| 241700_at | *ZFHX4* | | | zinc finger homeobox 4 | | -2.32 | 0.0237 |
| 242555_at | *C16orf87* | | | chromosome 16 open reading frame 87 | | -2.32 | 0.02709 |
| 225755_at | *KLHDC8B* | | | kelch domain containing 8B | | -2.33 | 0.00011 |
| 218140_x_at | *SRPRB* | | | signal recognition particle receptor, B subunit | | -2.33 | 0.00039 |
| 233809_at | *HYPK* | | | huntingtin interacting protein K | | -2.33 | 0.00058 |
| 222051_s_at |  | | |  | | -2.33 | 0.0007 |
| 212880_at | *WDR7* | | | WD repeat domain 7 | | -2.33 | 0.00092 |
| 225092_at | *RABEP1* | | | rabaptin, RAB GTPase binding effector protein 1 | | -2.33 | 0.00109 |
| 225173_at | *ARHGAP18* | | | Rho GTPase activating protein 18 | | -2.33 | 0.00119 |
| 203740_at | *MPHOSPH6* | | | M-phase phosphoprotein 6 | | -2.33 | 0.00147 |
| 228760_at | *SRSF8* | | | serine/arginine-rich splicing factor 8 | | -2.33 | 0.00157 |
| 213741_s_at | *KPNA1* | | | karyopherin alpha 1 (importin alpha 5) | | -2.33 | 0.0017 |
| 226154_at | *DNM1L* | | | dynamin 1-like | | -2.33 | 0.00192 |
| 225310_at | *RBMX* | | | RNA binding motif protein, X-linked; small nucleolar RNA, C/D box 61 | | -2.33 | 0.00209 |
| 202126_at | *PRPF4B* | | | PRP4 pre-mRNA processing factor 4 homolog B (yeast) | | -2.33 | 0.00213 |
| 211081_s_at | *MAP4K5* | | | mitogen-activated protein kinase kinase kinase kinase 5 | | -2.33 | 0.00407 |
| 230779_at | *TNRC6B* | | | trinucleotide repeat containing 6B | | -2.33 | 0.00414 |
| 1053_at | *RFC2* | | | replication factor C (activator 1) 2, 40kDa | | -2.33 | 0.00495 |
| 226867_at | *DENND4C* | | | DENN/MADD domain containing 4C | | -2.33 | 0.00502 |
| 218379_at | *RBM7* | | | RNA binding motif protein 7 | | -2.33 | 0.00553 |
| 218131_s_at | *GATAD2A* | | | GATA zinc finger domain containing 2A | | -2.33 | 0.00591 |
| 233496_s_at | *CFL2* | | | cofilin 2 (muscle) | | -2.33 | 0.00623 |
| 222434_at | *ENAH* | | | enabled homolog (Drosophila) | | -2.33 | 0.00784 |
| 209935_at | *ATP2C1* | | | ATPase, Ca++ transporting, type 2C, member 1 | | -2.33 | 0.0081 |
| 236935_at | *PTPN4* | | | protein tyrosine phosphatase, non-receptor type 4 (megakaryocyte) | | -2.33 | 0.01053 |
| 233093_s_at | *BIRC6* | | | baculoviral IAP repeat containing 6 | | -2.33 | 0.01177 |
| 1560383_at |  | | |  | | -2.33 | 0.01202 |
| 200891_s_at | *SSR1* | | | signal sequence receptor, alpha | | -2.33 | 0.01247 |
| 1556175_at | *MTSS1L* | | | metastasis suppressor 1-like | | -2.33 | 0.01416 |
| 205770_at | *GSR* | | | glutathione reductase | | -2.33 | 0.01422 |
| 225512_at | *ZBTB38* | | | zinc finger and BTB domain containing 38 | | -2.33 | 0.01439 |
| 222550_at | *ARMC1* | | | armadillo repeat containing 1 | | -2.33 | 0.01483 |
| 204386_s_at | *MRP63* | | | mitochondrial ribosomal protein 63 | | -2.33 | 0.01501 |
| 211423_s_at | *SC5D* | | | sterol-C5-desaturase | | -2.33 | 0.01759 |
| 205217_at | *TIMM8A* | | | translocase of inner mitochondrial membrane 8 homolog A (yeast) | | -2.33 | 0.01775 |
| 1553274_a_at | *SNRNP48* | | | small nuclear ribonucleoprotein 48kDa (U11/U12) | | -2.33 | 0.01825 |
| 218297_at | *FAM188A* | | | family with sequence similarity 188, member A | | -2.33 | 0.01879 |
| 202536_at | *CHMP2B* | | | charged multivesicular body protein 2B | | -2.33 | 0.01998 |
| 227637_at | *TFCP2* | | | transcription factor CP2 | | -2.33 | 0.02345 |
| 235303_at | *TRMT10B* | | | tRNA methyltransferase 10 homolog B (S. cerevisiae) | | -2.33 | 0.02429 |
| 236694_at | *TXLNG2P* | | | taxilin gamma 2, pseudogene | | -2.33 | 0.02469 |
| 232060_at | *ROR1* | | | receptor tyrosine kinase-like orphan receptor 1 | | -2.33 | 0.02525 |
| 238983_at | *NSUN7* | | | NOP2/Sun domain family, member 7 | | -2.33 | 0.02943 |
| 202007_at | *NID1* | | | nidogen 1 | | -2.33 | 0.03199 |
| 229228_at | *CREB5* | | | cAMP responsive element binding protein 5; uncharacterized LOC401317 | | -2.33 | 0.03266 |
| 225706_at | *GLCCI1* | | | glucocorticoid induced transcript 1 | | -2.33 | 0.03284 |
| 216623_x_at | *TOX3* | | | TOX high mobility group box family member 3 | | -2.33 | 0.03332 |
| 205426_s_at | *HIP1* | | | huntingtin interacting protein 1 | | -2.34 | 4.7x10^-5^ |
| 236429_at | *ZNF83* | | | zinc finger protein 83 | | -2.34 | 0.00019 |
| 235050_at | *SLC2A12* | | | solute carrier family 2 (facilitated glucose transporter), member 12 | | -2.34 | 0.00021 |
| 222606_at | *ZWILCH* | | | zwilch kinetochore protein | | -2.34 | 0.00024 |
| 226094_at | *PIK3C2A* | | | phosphatidylinositol-4-phosphate 3-kinase, catalytic subunit type 2 alpha | | -2.34 | 0.00051 |
| 222684_s_at | *NOL10* | | | nucleolar protein 10 | | -2.34 | 0.00092 |
| 231321_s_at | *ACER3* | | | alkaline ceramidase 3 | | -2.34 | 0.001 |
| 208766_s_at | *HNRNPR* | | | heterogeneous nuclear ribonucleoprotein R | | -2.34 | 0.0013 |
| 232198_at |  | | |  | | -2.34 | 0.00131 |
| 218772_x_at | *TMEM38B* | | | transmembrane protein 38B | | -2.34 | 0.00297 |
| 225493_at | *CCNT1* | | | cyclin T1 | | -2.34 | 0.00335 |
| 226874_at | *KLHL8* | | | kelch-like family member 8 | | -2.34 | 0.00415 |
| 215391_at | *MAP1A* | | | microtubule-associated protein 1A | | -2.34 | 0.00457 |
| 209433_s_at | *PPAT* | | | phosphoribosyl pyrophosphate amidotransferase | | -2.34 | 0.00495 |
| 235045_at | *RBM7* | | | RNA binding motif protein 7 | | -2.34 | 0.00505 |
| 200854_at | *NCOR1* | | | nuclear receptor corepressor 1 | | -2.34 | 0.00563 |
| 236834_at | *SCFD2* | | | sec1 family domain containing 2 | | -2.34 | 0.00843 |
| 215948_x_at | *ZMYM5* | | | zinc finger, MYM-type 5 | | -2.34 | 0.00973 |
| 235653_s_at | *THAP6* | | | THAP domain containing 6 | | -2.34 | 0.00974 |
| 1566201_at |  | | |  | | -2.34 | 0.00975 |
| 230141_at | *ARID4A* | | | AT rich interactive domain 4A (RBP1-like) | | -2.34 | 0.00991 |
| 201916_s_at | *SEC63* | | | SEC63 homolog (S. cerevisiae) | | -2.34 | 0.01096 |
| 1557239_at | *BBX* | | | bobby sox homolog (Drosophila) | | -2.34 | 0.01447 |
| 236251_at |  | | |  | | -2.34 | 0.01562 |
| 204962_s_at | *CENPA* | | | centromere protein A; solute carrier family 35, member F6 | | -2.34 | 0.01637 |
| 222807_at | *C11orf30* | | | chromosome 11 open reading frame 30 | | -2.34 | 0.02174 |
| 225710_at | *GNB4* | | | guanine nucleotide binding protein (G protein), beta polypeptide 4 | | -2.34 | 0.02266 |
| 230679_at | *DCAF10* | | | DDB1 and CUL4 associated factor 10 | | -2.34 | 0.0231 |
| 226289_at | *CAPRIN1* | | | cell cycle associated protein 1 | | -2.34 | 0.02396 |
| 235919_at | *CEP78* | | | centrosomal protein 78kDa | | -2.34 | 0.02506 |
| 201421_s_at | *WDR77* | | | WD repeat domain 77 | | -2.35 | 0.0001 |
| 1564521_x_at | *PRMT5* | | | protein arginine methyltransferase 5 | | -2.35 | 0.00011 |
| 226637_at | *UBE2H* | | | ubiquitin-conjugating enzyme E2H | | -2.35 | 0.00044 |
| 236219_at | *SLC35G1* | | | solute carrier family 35, member G1 | | -2.35 | 0.0008 |
| 1554250_s_at | *TRIM73* | | | tripartite motif containing 73 | | -2.35 | 0.00091 |
| 218947_s_at | *MTPAP* | | | mitochondrial poly(A) polymerase | | -2.35 | 0.00131 |
| 213506_at | *F2RL1* | | | coagulation factor II (thrombin) receptor-like 1 | | -2.35 | 0.00135 |
| 213750_at | *RSL1D1* | | | ribosomal L1 domain containing 1 | | -2.35 | 0.00136 |
| 229846_s_at | *MAPKAP1* | | | mitogen-activated protein kinase associated protein 1 | | -2.35 | 0.00178 |
| 201457_x_at | *BUB3* | | | BUB3 mitotic checkpoint protein | | -2.35 | 0.00206 |
| 215704_at | *FLG* | | | filaggrin | | -2.35 | 0.00249 |
| 225236_at | *RBM18* | | | RNA binding motif protein 18 | | -2.35 | 0.00269 |
| 222482_at | *SSBP3* | | | single stranded DNA binding protein 3 | | -2.35 | 0.0041 |
| 209705_at | *MTF2* | | | metal response element binding transcription factor 2 | | -2.35 | 0.00471 |
| 231676_s_at |  | | |  | | -2.35 | 0.00496 |
| 212296_at | *PSMD14* | | | proteasome (prosome, macropain) 26S subunit, non-ATPase, 14 | | -2.35 | 0.00508 |
| 206261_at | *ZNF239* | | | zinc finger protein 239 | | -2.35 | 0.00596 |
| 1558956_s_at | *IFT80* | | | intraflagellar transport 80 homolog (Chlamydomonas) | | -2.35 | 0.00629 |
| 232231_at | *RUNX2* | | | runt-related transcription factor 2 | | -2.35 | 0.00643 |
| 222604_at | *GTF3C3* | | | general transcription factor IIIC, polypeptide 3, 102kDa | | -2.35 | 0.00722 |
| 217134_at | *MTAP* | | | methylthioadenosine phosphorylase | | -2.35 | 0.00811 |
| 224253_at | *EXOC5* | | | exocyst complex component 5 | | -2.35 | 0.00998 |
| 244779_at | *ZDHHC2* | | | zinc finger, DHHC-type containing 2 | | -2.35 | 0.01021 |
| 227029_at | *FAM177A1* | | | family with sequence similarity 177, member A1 | | -2.35 | 0.01262 |
| 205263_at | *BCL10* | | | B-cell CLL/lymphoma 10 | | -2.35 | 0.01437 |
| 201443_s_at | *ATP6AP2* | | | ATPase, H+ transporting, lysosomal accessory protein 2 | | -2.35 | 0.02127 |
| 238458_at | *MICU3* | | | mitochondrial calcium uptake family, member 3 | | -2.35 | 0.03013 |
| 242056_at | *TRIM45* | | | tripartite motif containing 45 | | -2.36 | 0.00018 |
| 221485_at | *B4GALT5* | | | UDP-Gal:betaGlcNAc beta 1,4- galactosyltransferase, polypeptide 5 | | -2.36 | 0.0005 |
| 219174_at | *IFT74* | | | intraflagellar transport 74 homolog (Chlamydomonas) | | -2.36 | 0.00079 |
| 213685_at |  | | |  | | -2.36 | 0.00083 |
| 219479_at | *KDELC1* | | | KDEL (Lys-Asp-Glu-Leu) containing 1 | | -2.36 | 0.0013 |
| 205809_s_at | *WASL* | | | Wiskott-Aldrich syndrome-like | | -2.36 | 0.00155 |
| 241933_at | *QRSL1* | | | glutaminyl-tRNA synthase (glutamine-hydrolyzing)-like 1 | | -2.36 | 0.00225 |
| 220173_at | *CCDC176* | | | coiled-coil domain containing 176 | | -2.36 | 0.00235 |
| 231832_at | *GALNT4* | | | UDP-N-acetyl-alpha-D-galactosamine:polypeptide N-acetylgalactosaminyltransferase 4 (GalNAc-T4); POC1B-GALNT4 readthrough | | -2.36 | 0.00235 |
| 213233_s_at | *KLHL9* | | | kelch-like family member 9 | | -2.36 | 0.00262 |
| 230494_at | *SLC20A1* | | | solute carrier family 20 (phosphate transporter), member 1 | | -2.36 | 0.00418 |
| 221685_s_at | *SPDL1* | | | spindle apparatus coiled-coil protein 1 | | -2.36 | 0.0043 |
| 223155_at | *HDHD2* | | | haloacid dehalogenase-like hydrolase domain containing 2 | | -2.36 | 0.00435 |
| 201225_s_at | *SRRM1* | | | serine/arginine repetitive matrix 1 | | -2.36 | 0.00512 |
| 217426_at |  | | |  | | -2.36 | 0.00601 |
| 202051_s_at | *ZMYM4* | | | zinc finger, MYM-type 4 | | -2.36 | 0.00681 |
| 227696_at |  | | |  | | -2.36 | 0.00706 |
| 212857_x_at | *SUB1* | | | SUB1 homolog (S. cerevisiae) | | -2.36 | 0.00843 |
| 209332_s_at | *MAX* | | | MYC associated factor X | | -2.36 | 0.01102 |
| 1555573_at | *TTC40* | | | tetratricopeptide repeat domain 40 | | -2.36 | 0.01348 |
| 200864_s_at | *RAB11A* | | | RAB11A, member RAS oncogene family | | -2.36 | 0.01418 |
| 235264_at | *HCFC2* | | | host cell factor C2 | | -2.36 | 0.01478 |
| 201943_s_at | *CPD* | | | carboxypeptidase D | | -2.36 | 0.01781 |
| 235103_at | *MAN2A1* | | | mannosidase, alpha, class 2A, member 1 | | -2.36 | 0.01877 |
| 212297_at | *ATP13A3* | | | ATPase type 13A3 | | -2.36 | 0.01912 |
| 218050_at | *UFM1* | | | ubiquitin-fold modifier 1 | | -2.36 | 0.02199 |
| 204646_at | *DPYD* | | | dihydropyrimidine dehydrogenase | | -2.36 | 0.02596 |
| 212918_at | *RECQL* | | | RecQ protein-like (DNA helicase Q1-like) | | -2.36 | 0.0276 |
| 227942_s_at | *CRIPT* | | | cysteine-rich PDZ-binding protein | | -2.36 | 0.03063 |
| 243804_at | *MTMR7* | | | myotubularin related protein 7 | | -2.36 | 0.03387 |
| 228423_at | *MAP9* | | | microtubule-associated protein 9 | | -2.36 | 0.04316 |
| 203563_at | *AFAP1* | | | actin filament associated protein 1 | | -2.36 | 0.04434 |
| 219972_s_at | *PCNXL4* | | | pecanex-like 4 (Drosophila) | | -2.36 | 0.04584 |
| 203868_s_at | *VCAM1* | | | vascular cell adhesion molecule 1 | | -2.37 | 0.00047 |
| 205884_at | *ITGA4* | | | integrin, alpha 4 (antigen CD49D, alpha 4 subunit of VLA-4 receptor) | | -2.37 | 0.0012 |
| 237706_at | *STXBP4* | | | syntaxin binding protein 4 | | -2.37 | 0.00147 |
| 235253_at | *RAD1* | | | RAD1 homolog (S. pombe) | | -2.37 | 0.0019 |
| 218607_s_at | *SDAD1* | | | SDA1 domain containing 1 | | -2.37 | 0.00257 |
| 219335_at | *ARMCX5* | | | armadillo repeat containing, X-linked 5 | | -2.37 | 0.00293 |
| 225086_at | *FAM98B* | | | family with sequence similarity 98, member B | | -2.37 | 0.00357 |
| 219921_s_at | *DOCK5* | | | dedicator of cytokinesis 5 | | -2.37 | 0.00449 |
| 200071_at | *SMNDC1* | | | survival motor neuron domain containing 1 | | -2.37 | 0.00462 |
| 1566538_at | *MAGI1* | | | membrane associated guanylate kinase, WW and PDZ domain containing 1 | | -2.37 | 0.00537 |
| 222685_at | *HAUS6* | | | HAUS augmin-like complex, subunit 6 | | -2.37 | 0.00575 |
| 222036_s_at | *MCM4* | | | minichromosome maintenance complex component 4 | | -2.37 | 0.00654 |
| 211760_s_at | *VAMP4* | | | vesicle-associated membrane protein 4 | | -2.37 | 0.0066 |
| 225399_at | *TSEN15* | | | tRNA splicing endonuclease 15 homolog (S. cerevisiae) | | -2.37 | 0.00884 |
| 232506_s_at | *C15orf41* | | | chromosome 15 open reading frame 41 | | -2.37 | 0.01195 |
| 202104_s_at | *SPG7* | | | spastic paraplegia 7 (pure and complicated autosomal recessive) | | -2.37 | 0.0145 |
| 224934_at | *YIPF5* | | | Yip1 domain family, member 5 | | -2.37 | 0.01471 |
| 203218_at | *MAPK9* | | | mitogen-activated protein kinase 9 | | -2.37 | 0.01485 |
| 230618_s_at |  | | |  | | -2.37 | 0.01486 |
| 201253_s_at | *CDIPT* | | | CDP-diacylglycerol--inositol 3-phosphatidyltransferase | | -2.37 | 0.01487 |
| 224641_at | *FYTTD1* | | | forty-two-three domain containing 1 | | -2.37 | 0.01744 |
| 1555989_at | *DAAM1* | | | dishevelled associated activator of morphogenesis 1 | | -2.37 | 0.01824 |
| 210406_s_at | *RAB6A* | | | RAB6A, member RAS oncogene family; RAB6C, member RAS oncogene family; RAB6C-like | | -2.37 | 0.02343 |
| 236402_at | *BRAF* | | | v-raf murine sarcoma viral oncogene homolog B1 | | -2.37 | 0.02524 |
| 218268_at | *TBC1D15* | | | TBC1 domain family, member 15 | | -2.37 | 0.03856 |
| 209098_s_at | *JAG1* | | | jagged 1 | | -2.37 | 0.04328 |
| 230091_at |  | | |  | | -2.37 | 0.04482 |
| 205966_at | *TAF13* | | | TAF13 RNA polymerase II, TATA box binding protein (TBP)-associated factor, 18kDa | | -2.38 | 0.00044 |
| 202053_s_at | *ALDH3A2* | | | aldehyde dehydrogenase 3 family, member A2 | | -2.38 | 0.0005 |
| 212061_at | *U2SURP* | | | U2 snRNP-associated SURP domain containing | | -2.38 | 0.00085 |
| 213344_s_at | *H2AFX* | | | H2A histone family, member X | | -2.38 | 0.00094 |
| 227340_s_at | *RGMB* | | | RGM domain family, member B | | -2.38 | 0.00209 |
| 201410_at | *PLEKHB2* | | | pleckstrin homology domain containing, family B (evectins) member 2 | | -2.38 | 0.0029 |
| 238427_at | *GRPEL2* | | | GrpE-like 2, mitochondrial (E. coli) | | -2.38 | 0.00309 |
| 223176_at | *KCTD20* | | | potassium channel tetramerisation domain containing 20 | | -2.38 | 0.0034 |
| 229083_at |  | | |  | | -2.38 | 0.00392 |
| 223361_at | *ABRACL* | | | ABRA C-terminal like | | -2.38 | 0.00406 |
| 219200_at | *FASTKD3* | | | FAST kinase domains 3 | | -2.38 | 0.00583 |
| 228496_s_at | *CRIM1* | | | cysteine rich transmembrane BMP regulator 1 (chordin-like) | | -2.38 | 0.00609 |
| 228563_at | *GJC1* | | | gap junction protein, gamma 1, 45kDa | | -2.38 | 0.00615 |
| 242580_at |  | | |  | | -2.38 | 0.00687 |
| 213027_at | *TROVE2* | | | TROVE domain family, member 2 | | -2.38 | 0.00784 |
| 200606_at | *DSP* | | | desmoplakin | | -2.38 | 0.00799 |
| 229442_at | *C18orf54* | | | chromosome 18 open reading frame 54 | | -2.38 | 0.00837 |
| 1555945_s_at | *FAM120A* | | | family with sequence similarity 120A | | -2.38 | 0.00858 |
| 204068_at | *STK3* | | | serine/threonine kinase 3 | | -2.38 | 0.00929 |
| 205743_at | *STAC* | | | SH3 and cysteine rich domain | | -2.38 | 0.00984 |
| 215646_s_at | *VCAN* | | | versican | | -2.38 | 0.01118 |
| 219151_s_at | *RABL2A* | | | RAB, member of RAS oncogene family-like 2A; RAB, member of RAS oncogene family-like 2B | | -2.38 | 0.01162 |
| 235125_x_at | *FAM73A* | | | family with sequence similarity 73, member A | | -2.38 | 0.01566 |
| 200733_s_at | *PTP4A1* | | | protein tyrosine phosphatase type IVA, member 1 | | -2.38 | 0.01592 |
| 203820_s_at | *IGF2BP3* | | | insulin-like growth factor 2 mRNA binding protein 3 | | -2.38 | 0.01824 |
| 218043_s_at | *AZI2* | | | 5-azacytidine induced 2 | | -2.38 | 0.02015 |
| 230176_at | *UEVLD* | | | UEV and lactate/malate dehyrogenase domains | | -2.38 | 0.02022 |
| 223559_s_at | *INIP* | | | INTS3 and NABP interacting protein | | -2.38 | 0.02709 |
| 235521_at | *HOXA3* | | | homeobox A3 | | -2.38 | 0.03223 |
| 202375_at | *SEC24D* | | | SEC24 family, member D (S. cerevisiae) | | -2.38 | 0.03465 |
| 240592_at | *LCORL* | | | ligand dependent nuclear receptor corepressor-like | | -2.38 | 0.03877 |
| 226810_at | *OGFRL1* | | | opioid growth factor receptor-like 1 | | -2.38 | 0.04244 |
| 203046_s_at | *TIMELESS* | | | timeless circadian clock | | -2.39 | 0.00049 |
| 227646_at | *EBF1* | | | early B-cell factor 1 | | -2.39 | 0.00116 |
| 213561_at | *MCM9* | | | minichromosome maintenance complex component 9 | | -2.39 | 0.00166 |
| 200729_s_at | *ACTR2* | | | ARP2 actin-related protein 2 homolog (yeast) | | -2.39 | 0.0029 |
| 211580_s_at | *PIK3R3* | | | phosphoinositide-3-kinase, regulatory subunit 3 (gamma) | | -2.39 | 0.00321 |
| 226056_at | *ARHGAP31* | | | Rho GTPase activating protein 31 | | -2.39 | 0.00347 |
| 218082_s_at | *UBP1* | | | upstream binding protein 1 (LBP-1a) | | -2.39 | 0.00398 |
| 202874_s_at | *ATP6V1C1* | | | ATPase, H+ transporting, lysosomal 42kDa, V1 subunit C1 | | -2.39 | 0.00616 |
| 220947_s_at | *TBC1D10B* | | | TBC1 domain family, member 10B | | -2.39 | 0.00737 |
| 208838_at | *CAND1* | | | cullin-associated and neddylation-dissociated 1 | | -2.39 | 0.00852 |
| 202823_at | *TCEB1* | | | transcription elongation factor B (SIII), polypeptide 1 (15kDa, elongin C) | | -2.39 | 0.00886 |
| 218826_at | *SLC35F2* | | | solute carrier family 35, member F2 | | -2.39 | 0.00912 |
| 225782_at | *MSRB3* | | | methionine sulfoxide reductase B3 | | -2.39 | 0.01126 |
| 225619_at | *SLAIN1* | | | SLAIN motif family, member 1 | | -2.39 | 0.01151 |
| 220682_s_at |  | | |  | | -2.39 | 0.01296 |
| 213287_s_at | *KRT10* | | | keratin 10 | | -2.39 | 0.01809 |
| 242924_at |  | | |  | | -2.39 | 0.01868 |
| 238317_x_at |  | | |  | | -2.39 | 0.02029 |
| 208896_at | *DDX18* | | | DEAD (Asp-Glu-Ala-Asp) box polypeptide 18 | | -2.39 | 0.03792 |
| 213734_at | *RFC5* | | | replication factor C (activator 1) 5, 36.5kDa | | -2.4 | 0.00039 |
| 218859_s_at | *ESF1* | | | ESF1, nucleolar pre-rRNA processing protein, homolog (S. cerevisiae) | | -2.4 | 0.00083 |
| 205133_s_at | *HSPE1* | | | heat shock 10kDa protein 1 (chaperonin 10) | | -2.4 | 0.00088 |
| 203011_at | *IMPA1* | | | inositol(myo)-1(or 4)-monophosphatase 1 | | -2.4 | 0.00088 |
| 207153_s_at | *GLMN* | | | glomulin, FKBP associated protein | | -2.4 | 0.00106 |
| 225686_at | *SKA2* | | | spindle and kinetochore associated complex subunit 2 | | -2.4 | 0.00118 |
| 1552572_a_at | *MIPOL1* | | | mirror-image polydactyly 1 | | -2.4 | 0.00122 |
| 233035_at |  | | |  | | -2.4 | 0.00129 |
| 222623_s_at | *ZNF639* | | | zinc finger protein 639 | | -2.4 | 0.00205 |
| 227369_at | *SERBP1* | | | SERPINE1 mRNA binding protein 1 | | -2.4 | 0.0033 |
| 209218_at | *SQLE* | | | squalene epoxidase | | -2.4 | 0.00355 |
| 209658_at | *CDC16* | | | cell division cycle 16 | | -2.4 | 0.00376 |
| 213025_at | *THUMPD1* | | | THUMP domain containing 1 | | -2.4 | 0.00586 |
| 238662_at | *DPH6* | | | DPH6 homolog (S. cerevisiae) | | -2.4 | 0.0059 |
| 61732_r_at | *IFT74* | | | intraflagellar transport 74 homolog (Chlamydomonas) | | -2.4 | 0.00679 |
| 203531_at | *CUL5* | | | cullin 5 | | -2.4 | 0.00724 |
| 201472_at | *VBP1* | | | von Hippel-Lindau binding protein 1 | | -2.4 | 0.00834 |
| 225231_at | *CBL* | | | Cbl proto-oncogene, E3 ubiquitin protein ligase | | -2.4 | 0.00865 |
| 208780_x_at | *VAPA* | | | VAMP (vesicle-associated membrane protein)-associated protein A, 33kDa | | -2.4 | 0.00966 |
| 208753_s_at | *NAP1L1* | | | nucleosome assembly protein 1-like 1 | | -2.4 | 0.01143 |
| 221474_at | *MYL12B* | | | myosin, light chain 12B, regulatory | | -2.4 | 0.01295 |
| 1555561_a_at | *UGGT2* | | | UDP-glucose glycoprotein glucosyltransferase 2 | | -2.4 | 0.01483 |
| 209894_at | *LEPR* | | | leptin receptor | | -2.4 | 0.01679 |
| 232587_at | *EML4* | | | echinoderm microtubule associated protein like 4 | | -2.4 | 0.01839 |
| 238681_at | *GDPD1* | | | glycerophosphodiester phosphodiesterase domain containing 1 | | -2.4 | 0.02251 |
| 223958_s_at | *DNAL1* | | | dynein, axonemal, light chain 1 | | -2.4 | 0.02559 |
| 202654_x_at | *Mar-07* | | | membrane-associated ring finger (C3HC4) 7, E3 ubiquitin protein ligase | | -2.4 | 0.02812 |
| 231966_at | *PPP1R9A* | | | protein phosphatase 1, regulatory subunit 9A | | -2.4 | 0.04472 |
| 202048_s_at | *CBX6* | | | chromobox homolog 6 | | -2.41 | 6.4x10^-5^ |
| 1559889_at |  | | |  | | -2.41 | 0.00068 |
| 201519_at | *TOMM70A* | | | translocase of outer mitochondrial membrane 70 homolog A (S. cerevisiae) | | -2.41 | 0.00077 |
| 225073_at | *PPHLN1* | | | periphilin 1 | | -2.41 | 0.00088 |
| 226719_at | *DERL2* | | | derlin 2 | | -2.41 | 0.00098 |
| 235051_at | *CCDC50* | | | coiled-coil domain containing 50 | | -2.41 | 0.00114 |
| 212222_at | *PSME4* | | | proteasome (prosome, macropain) activator subunit 4 | | -2.41 | 0.00121 |
| 202491_s_at | *IKBKAP* | | | inhibitor of kappa light polypeptide gene enhancer in B-cells, kinase complex-associated protein | | -2.41 | 0.00128 |
| 203745_at | *HCCS* | | | holocytochrome c synthase | | -2.41 | 0.00176 |
| 223136_at | *AIG1* | | | androgen-induced 1 | | -2.41 | 0.00182 |
| 225460_at | *SEC22C* | | | SEC22 vesicle trafficking protein homolog C (S. cerevisiae) | | -2.41 | 0.00184 |
| 211597_s_at | *HOPX* | | | HOP homeobox | | -2.41 | 0.00228 |
| 214484_s_at | *SIGMAR1* | | | sigma non-opioid intracellular receptor 1 | | -2.41 | 0.00309 |
| 232985_s_at | *DPPA4* | | | developmental pluripotency associated 4 | | -2.41 | 0.00382 |
| 203529_at | *PPP6C* | | | protein phosphatase 6, catalytic subunit | | -2.41 | 0.00422 |
| 242300_at |  | | |  | | -2.41 | 0.00431 |
| 87100_at | *ABHD2* | | | abhydrolase domain containing 2 | | -2.41 | 0.00546 |
| 218422_s_at | *RBM26* | | | RNA binding motif protein 26 | | -2.41 | 0.00585 |
| 229167_at | *PURA* | | | purine-rich element binding protein A | | -2.41 | 0.00863 |
| 226223_at | *PAWR* | | | PRKC, apoptosis, WT1, regulator | | -2.41 | 0.011 |
| 214794_at | *PA2G4* | | | proliferation-associated 2G4, 38kDa | | -2.41 | 0.01112 |
| 202213_s_at | *CUL4B* | | | cullin 4B | | -2.41 | 0.01172 |
| 204672_s_at | *ANKRD6* | | | ankyrin repeat domain 6 | | -2.41 | 0.01277 |
| 212229_s_at | *FBXO21* | | | F-box protein 21 | | -2.41 | 0.01479 |
| 215664_s_at | *EPHA5* | | | EPH receptor A5 | | -2.41 | 0.01582 |
| 226649_at | *PANK1* | | | pantothenate kinase 1 | | -2.41 | 0.01904 |
| 1556009_at | *PEX13* | | | peroxisomal biogenesis factor 13 | | -2.41 | 0.02565 |
| 210567_s_at | *SKP2* | | | S-phase kinase-associated protein 2, E3 ubiquitin protein ligase | | -2.41 | 0.02577 |
| 238421_at | *RC3H2* | | | ring finger and CCCH-type domains 2 | | -2.41 | 0.03028 |
| 235256_s_at | *GALM* | | | galactose mutarotase (aldose 1-epimerase) | | -2.41 | 0.03498 |
| 210098_s_at |  | | |  | | -2.42 | 0.00017 |
| 200870_at | *STRAP* | | | serine/threonine kinase receptor associated protein | | -2.42 | 0.00126 |
| 212640_at | *PTPLB* | | | protein tyrosine phosphatase-like (proline instead of catalytic arginine), member b | | -2.42 | 0.00136 |
| 209817_at | *PPP3CB* | | | protein phosphatase 3, catalytic subunit, beta isozyme | | -2.42 | 0.00175 |
| 223625_at | *FAM126A* | | | family with sequence similarity 126, member A | | -2.42 | 0.00202 |
| 236350_at |  | | |  | | -2.42 | 0.0023 |
| 217963_s_at | *NGFRAP1* | | | nerve growth factor receptor (TNFRSF16) associated protein 1 | | -2.42 | 0.00231 |
| 1555560_at | *UGGT2* | | | UDP-glucose glycoprotein glucosyltransferase 2 | | -2.42 | 0.00298 |
| 226752_at | *FAM174A* | | | family with sequence similarity 174, member A | | -2.42 | 0.00309 |
| 225726_s_at | *PLEKHH1* | | | pleckstrin homology domain containing, family H (with MyTH4 domain) member 1 | | -2.42 | 0.00337 |
| 201128_s_at | *ACLY* | | | ATP citrate lyase | | -2.42 | 0.00347 |
| 202277_at | *SPTLC1* | | | serine palmitoyltransferase, long chain base subunit 1 | | -2.42 | 0.00364 |
| 225469_at | *LYRM5* | | | LYR motif containing 5 | | -2.42 | 0.00433 |
| 218195_at | *C6orf211* | | | chromosome 6 open reading frame 211 | | -2.42 | 0.0056 |
| 225255_at | *MRPL35* | | | mitochondrial ribosomal protein L35 | | -2.42 | 0.00834 |
| 219030_at | *TPRKB* | | | TP53RK binding protein | | -2.42 | 0.00858 |
| 236752_at |  | | |  | | -2.42 | 0.01291 |
| 226884_at | *LRRN1* | | | leucine rich repeat neuronal 1 | | -2.42 | 0.01586 |
| 218884_s_at | *GUF1* | | | GUF1 GTPase homolog (S. cerevisiae) | | -2.42 | 0.01735 |
| 217299_s_at | *NBN* | | | nibrin | | -2.42 | 0.01802 |
| 1556348_at | *HEATR1* | | | HEAT repeat containing 1 | | -2.42 | 0.02077 |
| 224862_at | *GNAQ* | | | guanine nucleotide binding protein (G protein), q polypeptide | | -2.42 | 0.02083 |
| 202820_at | *AHR* | | | aryl hydrocarbon receptor | | -2.42 | 0.03063 |
| 230788_at | *GCNT2* | | | glucosaminyl (N-acetyl) transferase 2, I-branching enzyme (I blood group) | | -2.42 | 0.04059 |
| 218157_x_at | *CDC42SE1* | | | CDC42 small effector 1 | | -2.43 | 0.00035 |
| 209526_s_at | *HDGFRP3* | | | hepatoma-derived growth factor, related protein 3 | | -2.43 | 0.00077 |
| 202976_s_at | *RHOBTB3* | | | Rho-related BTB domain containing 3 | | -2.43 | 0.00106 |
| 235931_at | *METTL21A* | | | methyltransferase like 21A | | -2.43 | 0.00112 |
| 230893_at | *DNAJC21* | | | DnaJ (Hsp40) homolog, subfamily C, member 21 | | -2.43 | 0.00131 |
| 203699_s_at | *DIO2* | | | deiodinase, iodothyronine, type II | | -2.43 | 0.00146 |
| 225724_at | *FLJ31306* | | | uncharacterized LOC379025 | | -2.43 | 0.00212 |
| 1554273_a_at | *ERAP2* | | | endoplasmic reticulum aminopeptidase 2 | | -2.43 | 0.00217 |
| 209094_at | *DDAH1* | | | dimethylarginine dimethylaminohydrolase 1 | | -2.43 | 0.00232 |
| 237158_s_at | *MPHOSPH9* | | | M-phase phosphoprotein 9 | | -2.43 | 0.00434 |
| 217776_at | *RDH11* | | | retinol dehydrogenase 11 (all-trans/9-cis/11-cis) | | -2.43 | 0.00456 |
| 222770_s_at | *GUF1* | | | GUF1 GTPase homolog (S. cerevisiae) | | -2.43 | 0.00526 |
| 206943_at | *TGFBR1* | | | transforming growth factor, beta receptor 1 | | -2.43 | 0.00597 |
| 209525_at | *HDGFRP3* | | | hepatoma-derived growth factor, related protein 3 | | -2.43 | 0.00745 |
| 229614_at | *ZNF320* | | | zinc finger protein 320 | | -2.43 | 0.00753 |
| 210716_s_at | *CLIP1* | | | CAP-GLY domain containing linker protein 1 | | -2.43 | 0.00754 |
| 1567457_at | *RAC1* | | | ras-related C3 botulinum toxin substrate 1 (rho family, small GTP binding protein Rac1) | | -2.43 | 0.00855 |
| 1555202_a_at | *RPRD1A* | | | regulation of nuclear pre-mRNA domain containing 1A | | -2.43 | 0.00893 |
| 201031_s_at | *HNRNPH1* | | | heterogeneous nuclear ribonucleoprotein H1 (H) | | -2.43 | 0.01015 |
| 1569194_at | *ZNF789* | | | zinc finger protein 789 | | -2.43 | 0.01015 |
| 1564010_at | *CAST* | | | calpastatin | | -2.43 | 0.01127 |
| 235287_at | *CDK6* | | | cyclin-dependent kinase 6 | | -2.43 | 0.01276 |
| 218323_at | *RHOT1* | | | ras homolog family member T1 | | -2.43 | 0.01428 |
| 220172_at | *DCAF17* | | | DDB1 and CUL4 associated factor 17 | | -2.43 | 0.01437 |
| 212959_s_at | *GNPTAB* | | | N-acetylglucosamine-1-phosphate transferase, alpha and beta subunits | | -2.43 | 0.01784 |
| 239262_at |  | | |  | | -2.43 | 0.0312 |
| 222805_at | *MANEA* | | | mannosidase, endo-alpha | | -2.43 | 0.04137 |
| 243733_at |  | | |  | | -2.43 | 0.04425 |
| 224037_at | *SDAD1* | | | SDA1 domain containing 1 | | -2.44 | 0.00026 |
| 201266_at | *TXNRD1* | | | thioredoxin reductase 1 | | -2.44 | 0.00081 |
| 224964_s_at | *GNG2* | | | guanine nucleotide binding protein (G protein), gamma 2 | | -2.44 | 0.00084 |
| 221276_s_at | *SYNC* | | | syncoilin, intermediate filament protein | | -2.44 | 0.00104 |
| 208910_s_at | *C1QBP* | | | complement component 1, q subcomponent binding protein | | -2.44 | 0.00114 |
| 222233_s_at | *DCLRE1C* | | | DNA cross-link repair 1C | | -2.44 | 0.00193 |
| 231240_at | *DIO2* | | | deiodinase, iodothyronine, type II | | -2.44 | 0.00205 |
| 209533_s_at | *PLAA* | | | phospholipase A2-activating protein | | -2.44 | 0.00489 |
| 223599_at | *TRIM6* | | | tripartite motif containing 6 | | -2.44 | 0.0055 |
| 202677_at | *RASA1* | | | RAS p21 protein activator (GTPase activating protein) 1 | | -2.44 | 0.0067 |
| 215617_at | *SPATS2L* | | | spermatogenesis associated, serine-rich 2-like | | -2.44 | 0.00725 |
| 219605_at | *ZNF3* | | | zinc finger protein 3 | | -2.44 | 0.00771 |
| 1554471_a_at | *ANKRD13C* | | | ankyrin repeat domain 13C | | -2.44 | 0.00963 |
| 217800_s_at | *NDFIP1* | | | Nedd4 family interacting protein 1 | | -2.44 | 0.01554 |
| 208691_at | *TFRC* | | | transferrin receptor (p90, CD71) | | -2.44 | 0.01592 |
| 203086_at | *KIF2A* | | | kinesin heavy chain member 2A | | -2.44 | 0.01599 |
| 238007_at | *ZNF271* | | | zinc finger protein 271 | | -2.44 | 0.01685 |
| 223301_s_at | *CCDC82* | | | coiled-coil domain containing 82 | | -2.44 | 0.01844 |
| 220241_at | *TMCO3* | | | transmembrane and coiled-coil domains 3 | | -2.44 | 0.01907 |
| 228418_at | *EXOC5* | | | exocyst complex component 5 | | -2.44 | 0.02134 |
| 202552_s_at | *CRIM1* | | | cysteine rich transmembrane BMP regulator 1 (chordin-like) | | -2.44 | 0.02858 |
| 37943_at | *ZFYVE26* | | | zinc finger, FYVE domain containing 26 | | -2.44 | 0.03474 |
| 229858_at |  | | |  | | -2.44 | 0.03776 |
| 214751_at | *ZNF468* | | | zinc finger protein 468 | | -2.44 | 0.03855 |
| 221935_s_at | *EOGT* | | | EGF domain-specific O-linked N-acetylglucosamine (GlcNAc) transferase | | -2.44 | 0.04242 |
| 234032_at |  | | |  | | -2.44 | 0.04296 |
| 213212_x_at | *GOLGA6L10* | | | golgin A6 family-like 10; golgin A6 family-like 3; golgin A6 family-like 4; golgin A6 family-like 5 (pseudogene); golgin A6 family-like 9 | | -2.44 | 0.04786 |
| 203199_s_at | *MTRR* | | | 5-methyltetrahydrofolate-homocysteine methyltransferase reductase | | -2.45 | 0.0001 |
| 212514_x_at | *DDX3X* | | | DEAD (Asp-Glu-Ala-Asp) box polypeptide 3, X-linked | | -2.45 | 0.00025 |
| 219006_at | *NDUFAF4* | | | NADH dehydrogenase (ubiquinone) complex I, assembly factor 4 | | -2.45 | 0.00064 |
| 202085_at | *TJP2* | | | tight junction protein 2 | | -2.45 | 0.00163 |
| 218303_x_at | *KRCC1* | | | lysine-rich coiled-coil 1 | | -2.45 | 0.00313 |
| 216942_s_at | *CD58* | | | CD58 molecule | | -2.45 | 0.0041 |
| 226933_s_at | *ID4* | | | inhibitor of DNA binding 4, dominant negative helix-loop-helix protein | | -2.45 | 0.00426 |
| 1552370_at | *C4orf33* | | | chromosome 4 open reading frame 33 | | -2.45 | 0.00542 |
| 225402_at | *TP53RK* | | | TP53 regulating kinase | | -2.45 | 0.00559 |
| 219758_at | *TTC26* | | | tetratricopeptide repeat domain 26 | | -2.45 | 0.00635 |
| 226784_at | *TWISTNB* | | | TWIST neighbor | | -2.45 | 0.00694 |
| 212623_at | *TMEM41B* | | | transmembrane protein 41B | | -2.45 | 0.00768 |
| 238243_at |  | | |  | | -2.45 | 0.00778 |
| 228336_at | *PWWP2A* | | | PWWP domain containing 2A | | -2.45 | 0.0113 |
| 223144_s_at | *AKIRIN2* | | | akirin 2 | | -2.45 | 0.01494 |
| 223037_at | *PDZD11* | | | PDZ domain containing 11 | | -2.45 | 0.0152 |
| 226831_at | *SLC25A46* | | | solute carrier family 25, member 46 | | -2.45 | 0.02147 |
| 227198_at | *AFF3* | | | AF4/FMR2 family, member 3 | | -2.45 | 0.02174 |
| 222473_s_at | *ERBB2IP* | | | erbb2 interacting protein | | -2.45 | 0.02722 |
| 227692_at | *GNAI1* | | | guanine nucleotide binding protein (G protein), alpha inhibiting activity polypeptide 1 | | -2.45 | 0.03214 |
| 224734_at | *HMGB1* | | | high mobility group box 1 | | -2.45 | 0.03358 |
| 1558249_s_at | *STX16* | | | syntaxin 16 | | -2.45 | 0.03386 |
| 1556678_a_at |  | | |  | | -2.45 | 0.04279 |
| 218511_s_at | *PNPO* | | | pyridoxamine 5'-phosphate oxidase | | -2.46 | 0.00029 |
| 205284_at | *URB2* | | | URB2 ribosome biogenesis 2 homolog (S. cerevisiae) | | -2.46 | 0.00031 |
| 232164_s_at | *EPPK1* | | | epiplakin 1 | | -2.46 | 0.00168 |
| 205532_s_at | *CDH6* | | | cadherin 6, type 2, K-cadherin (fetal kidney) | | -2.46 | 0.0017 |
| 230232_at | *SEL1L3* | | | sel-1 suppressor of lin-12-like 3 (C. elegans) | | -2.46 | 0.00295 |
| 204234_s_at | *ZNF195* | | | zinc finger protein 195 | | -2.46 | 0.00494 |
| 235292_at | *FLJ32255* | | | uncharacterized LOC643977 | | -2.46 | 0.00508 |
| 201021_s_at | *DSTN* | | | destrin (actin depolymerizing factor) | | -2.46 | 0.00742 |
| 228128_x_at | *PAPPA* | | | pregnancy-associated plasma protein A, pappalysin 1 | | -2.46 | 0.00854 |
| 231839_at | *PDE12* | | | phosphodiesterase 12 | | -2.46 | 0.00922 |
| 225011_at | *PRKAR2A* | | | protein kinase, cAMP-dependent, regulatory, type II, alpha | | -2.46 | 0.01019 |
| 1557684_at | *ZNF286A* | | | zinc finger protein 286A | | -2.46 | 0.01033 |
| 201042_at | *TGM2* | | | transglutaminase 2 (C polypeptide, protein-glutamine-gamma-glutamyltransferase) | | -2.46 | 0.01078 |
| 212905_at | *CSTF2T* | | | cleavage stimulation factor, 3' pre-RNA, subunit 2, 64kDa, tau variant | | -2.46 | 0.01119 |
| 239835_at | *KBTBD8* | | | kelch repeat and BTB (POZ) domain containing 8 | | -2.46 | 0.01186 |
| 203603_s_at | *ZEB2* | | | zinc finger E-box binding homeobox 2 | | -2.46 | 0.01216 |
| 1560384_a_at |  | | |  | | -2.46 | 0.01754 |
| 201334_s_at | *ARHGEF12* | | | Rho guanine nucleotide exchange factor (GEF) 12 | | -2.46 | 0.01948 |
| 228478_at |  | | |  | | -2.46 | 0.02263 |
| 205091_x_at | *RECQL* | | | RecQ protein-like (DNA helicase Q1-like) | | -2.46 | 0.02341 |
| 1558605_at |  | | |  | | -2.46 | 0.02505 |
| 1553955_at | *PPP1R21* | | | protein phosphatase 1, regulatory subunit 21 | | -2.46 | 0.02599 |
| 222637_at | *COMMD10* | | | COMM domain containing 10 | | -2.46 | 0.028 |
| 223642_at | *ZIC2* | | | Zic family member 2 | | -2.46 | 0.03111 |
| 225663_at | *ACBD5* | | | acyl-CoA binding domain containing 5 | | -2.46 | 0.04307 |
| 201298_s_at | *MOB1A* | | | MOB kinase activator 1A | | -2.47 | 0.00038 |
| 200807_s_at | *HSPD1* | | | heat shock 60kDa protein 1 (chaperonin) | | -2.47 | 0.0004 |
| 220260_at | *TBC1D19* | | | TBC1 domain family, member 19 | | -2.47 | 0.00044 |
| 205836_s_at | *YTHDC2* | | | YTH domain containing 2 | | -2.47 | 0.0008 |
| 225106_s_at | *OGFOD1* | | | 2-oxoglutarate and iron-dependent oxygenase domain containing 1 | | -2.47 | 0.00086 |
| 231810_at | *BRI3BP* | | | BRI3 binding protein | | -2.47 | 0.00093 |
| 218273_s_at | *PDP1* | | | pyruvate dehyrogenase phosphatase catalytic subunit 1 | | -2.47 | 0.001 |
| 214959_s_at | *API5* | | | apoptosis inhibitor 5 | | -2.47 | 0.00136 |
| 229393_at | *L3MBTL3* | | | l(3)mbt-like 3 (Drosophila) | | -2.47 | 0.00143 |
| 236692_at |  | | |  | | -2.47 | 0.00181 |
| 217725_x_at | *SERBP1* | | | SERPINE1 mRNA binding protein 1 | | -2.47 | 0.00245 |
| 213612_x_at |  | | |  | | -2.47 | 0.00385 |
| 1555144_at | *ARL17A* | | | ADP-ribosylation factor-like 17A; ADP-ribosylation factor-like 17B | | -2.47 | 0.00447 |
| 1553764_a_at | *AJUBA* | | | ajuba LIM protein | | -2.47 | 0.00464 |
| 213024_at | *TMF1* | | | TATA element modulatory factor 1 | | -2.47 | 0.00522 |
| 201091_s_at | *CBX3* | | | chromobox homolog 3 | | -2.47 | 0.0058 |
| 225806_at | *AJUBA* | | | ajuba LIM protein | | -2.47 | 0.00611 |
| 32088_at | *BLZF1* | | | basic leucine zipper nuclear factor 1 | | -2.47 | 0.007 |
| 227364_at |  | | |  | | -2.47 | 0.00789 |
| 208651_x_at | *CD24* | | | CD24 molecule | | -2.47 | 0.00849 |
| 230806_s_at | *FAM65A* | | | family with sequence similarity 65, member A | | -2.47 | 0.00893 |
| 227808_at | *DNAJC15* | | | DnaJ (Hsp40) homolog, subfamily C, member 15 | | -2.47 | 0.01418 |
| 222924_at | *SLMAP* | | | sarcolemma associated protein | | -2.47 | 0.01672 |
| 213093_at | *PRKCA* | | | protein kinase C, alpha | | -2.47 | 0.02305 |
| 233898_s_at | *FGFR1OP2* | | | FGFR1 oncogene partner 2 | | -2.47 | 0.03007 |
| 219696_at | *DENND1B* | | | DENN/MADD domain containing 1B | | -2.47 | 0.03243 |
| 203852_s_at | *SMN1* | | | survival of motor neuron 1, telomeric; survival of motor neuron 2, centromeric | | -2.47 | 0.03415 |
| 208924_at | *RNF11* | | | ring finger protein 11 | | -2.47 | 0.03754 |
| 218875_s_at | *FBXO5* | | | F-box protein 5 | | -2.47 | 0.03924 |
| 211686_s_at | *MAK16* | | | MAK16 homolog (S. cerevisiae) | | -2.48 | 4.4x10^-5^ |
| 213903_s_at | *RQCD1* | | | RCD1 required for cell differentiation1 homolog (S. pombe) | | -2.48 | 0.00012 |
| 235291_s_at | *FLJ32255* | | | uncharacterized LOC643977 | | -2.48 | 0.00013 |
| 213186_at | *DZIP3* | | | DAZ interacting zinc finger protein 3 | | -2.48 | 0.00043 |
| 238465_at | *SETD9* | | | SET domain containing 9 | | -2.48 | 0.00046 |
| 202658_at |  | | |  | | -2.48 | 0.00059 |
| 1555351_s_at | *PPHLN1* | | | periphilin 1 | | -2.48 | 0.00112 |
| 203124_s_at | *SLC11A2* | | | solute carrier family 11 (proton-coupled divalent metal ion transporters), member 2 | | -2.48 | 0.00139 |
| 215505_s_at | *STRN3* | | | striatin, calmodulin binding protein 3 | | -2.48 | 0.0018 |
| 205794_s_at | *NOVA1* | | | neuro-oncological ventral antigen 1 | | -2.48 | 0.0024 |
| 1553588_at | *ND3* | | | NADH dehydrogenase, subunit 3 (complex I); SH3-domain kinase binding protein 1 | | -2.48 | 0.00257 |
| 202797_at | *SACM1L* | | | SAC1 suppressor of actin mutations 1-like (yeast) | | -2.48 | 0.00316 |
| 227559_at | *NDUFAF4* | | | NADH dehydrogenase (ubiquinone) complex I, assembly factor 4 | | -2.48 | 0.00414 |
| 201766_at | *ELAC2* | | | elaC ribonuclease Z 2 | | -2.48 | 0.00462 |
| 228181_at | *SLC30A1* | | | solute carrier family 30 (zinc transporter), member 1 | | -2.48 | 0.00462 |
| 218737_at | *SBNO1* | | | strawberry notch homolog 1 (Drosophila) | | -2.48 | 0.00463 |
| 38241_at | *BTN3A3* | | | butyrophilin, subfamily 3, member A3 | | -2.48 | 0.00479 |
| 230032_at | *OSGEPL1* | | | O-sialoglycoprotein endopeptidase-like 1 | | -2.48 | 0.00501 |
| 213077_at | *YTHDC2* | | | YTH domain containing 2 | | -2.48 | 0.00512 |
| 227748_at | *RBMXL1* | | | RNA binding motif protein, X-linked-like 1 | | -2.48 | 0.00699 |
| 222371_at |  | | |  | | -2.48 | 0.01036 |
| 223021_x_at | *VTA1* | | | Vps20-associated 1 homolog (S. cerevisiae) | | -2.48 | 0.01065 |
| 208319_s_at | *RBM3* | | | RNA binding motif (RNP1, RRM) protein 3 | | -2.48 | 0.01151 |
| 227647_at | *KCNE3* | | | potassium voltage-gated channel, Isk-related family, member 3 | | -2.48 | 0.01199 |
| 226479_at | *KBTBD6* | | | kelch repeat and BTB (POZ) domain containing 6 | | -2.48 | 0.01216 |
| 227356_at |  | | |  | | -2.48 | 0.01292 |
| 203987_at | *FZD6* | | | frizzled family receptor 6 | | -2.48 | 0.015 |
| 213510_x_at | *USP32P2* | | | ubiquitin specific peptidase 32 pseudogene 2 | | -2.48 | 0.01518 |
| 238002_at | *GOLIM4* | | | golgi integral membrane protein 4 | | -2.48 | 0.01915 |
| 218170_at | *ISOC1* | | | isochorismatase domain containing 1 | | -2.48 | 0.03001 |
| 219819_s_at | *MRPS28* | | | mitochondrial ribosomal protein S28 | | -2.49 | 0.00076 |
| 226085_at | *CBX5* | | | chromobox homolog 5 | | -2.49 | 0.00138 |
| 209895_at | *PTPN11* | | | protein tyrosine phosphatase, non-receptor type 11 | | -2.49 | 0.00144 |
| 219481_at | *TTC13* | | | tetratricopeptide repeat domain 13 | | -2.49 | 0.0019 |
| 200880_at | *DNAJA1* | | | DnaJ (Hsp40) homolog, subfamily A, member 1 | | -2.49 | 0.00233 |
| 212839_s_at | *TROVE2* | | | TROVE domain family, member 2 | | -2.49 | 0.00243 |
| 202713_s_at | *KIAA0391* | | | KIAA0391 | | -2.49 | 0.00244 |
| 213619_at |  | | |  | | -2.49 | 0.00289 |
| 223716_s_at | *ZRANB2* | | | zinc finger, RAN-binding domain containing 2 | | -2.49 | 0.00358 |
| 209609_s_at | *MRPL9* | | | mitochondrial ribosomal protein L9 | | -2.49 | 0.00358 |
| 217828_at | *SLTM* | | | SAFB-like, transcription modulator | | -2.49 | 0.00451 |
| 222872_x_at | *NABP1* | | | nucleic acid binding protein 1 | | -2.49 | 0.00485 |
| 209798_at | *NPAT* | | | nuclear protein, ataxia-telangiectasia locus | | -2.49 | 0.0064 |
| 203855_at | *WDR47* | | | WD repeat domain 47 | | -2.49 | 0.00668 |
| 222209_s_at | *TMEM135* | | | transmembrane protein 135 | | -2.49 | 0.0076 |
| 219913_s_at | *CRNKL1* | | | crooked neck pre-mRNA splicing factor-like 1 (Drosophila) | | -2.49 | 0.00804 |
| 34031_i_at | *KRIT1* | | | KRIT1, ankyrin repeat containing | | -2.49 | 0.0083 |
| 235072_s_at | *KIF13A* | | | kinesin family member 13A | | -2.49 | 0.01095 |
| 223651_x_at | *CDC23* | | | cell division cycle 23 | | -2.49 | 0.01098 |
| 236175_at | *TRIM55* | | | tripartite motif containing 55 | | -2.49 | 0.01598 |
| 210432_s_at | *SCN3A* | | | sodium channel, voltage-gated, type III, alpha subunit | | -2.49 | 0.03833 |
| 230009_at | *FAM118B* | | | family with sequence similarity 118, member B | | -2.5 | 0.00039 |
| 1567213_at | *PNN* | | | pinin, desmosome associated protein | | -2.5 | 0.00041 |
| 222488_s_at | *DCTN4* | | | dynactin 4 (p62) | | -2.5 | 0.00457 |
| 228479_at | *SOAT1* | | | sterol O-acyltransferase 1 | | -2.5 | 0.00485 |
| 225945_at | *ZNF655* | | | zinc finger protein 655 | | -2.5 | 0.00537 |
| 208843_s_at | *GORASP2* | | | golgi reassembly stacking protein 2, 55kDa | | -2.5 | 0.00568 |
| 203406_at | *MFAP1* | | | microfibrillar-associated protein 1 | | -2.5 | 0.00628 |
| 203830_at | *C17orf75* | | | chromosome 17 open reading frame 75 | | -2.5 | 0.00904 |
| 223099_s_at | *LONP2* | | | lon peptidase 2, peroxisomal | | -2.5 | 0.00977 |
| 235360_at | *PLEKHM3* | | | pleckstrin homology domain containing, family M, member 3 | | -2.5 | 0.00994 |
| 224630_at | *ERLEC1* | | | endoplasmic reticulum lectin 1 | | -2.5 | 0.00998 |
| 1558699_a_at | *HERPUD2* | | | HERPUD family member 2 | | -2.5 | 0.01073 |
| 1559656_a_at | *MAMDC2-AS1* | | | MAMDC2 antisense RNA 1 | | -2.5 | 0.01191 |
| 1560038_at |  | | | uncharacterized LOC100506071 | | -2.5 | 0.0131 |
| 217956_s_at | *ENOPH1* | | | enolase-phosphatase 1 | | -2.5 | 0.01356 |
| 204201_s_at | *PTPN13* | | | protein tyrosine phosphatase, non-receptor type 13 (APO-1/CD95 (Fas)-associated phosphatase) | | -2.5 | 0.014 |
| 227220_at | *NFXL1* | | | nuclear transcription factor, X-box binding-like 1 | | -2.5 | 0.01442 |
| 243423_at | *TNIP1* | | | TNFAIP3 interacting protein 1 | | -2.5 | 0.01634 |
| 207405_s_at | *RAD17* | | | RAD17 homolog (S. pombe) | | -2.5 | 0.0172 |
| 236215_at |  | | |  | | -2.5 | 0.01897 |
| 216914_at | *CDC25C* | | | cell division cycle 25C | | -2.5 | 0.01935 |
| 203874_s_at | *SMARCA1* | | | SWI/SNF related, matrix associated, actin dependent regulator of chromatin, subfamily a, member 1 | | -2.5 | 0.02501 |
| 242228_at |  | | |  | | -2.5 | 0.02504 |
| 227157_at | *CCDC111* | | | coiled-coil domain containing 111 | | -2.5 | 0.02634 |
| 202704_at | *TOB1* | | | transducer of ERBB2, 1 | | -2.5 | 0.04175 |
| 225252_at | *SRXN1* | | | sulfiredoxin 1 | | -2.51 | 4.8x10^-5^ |
| 219778_at | *ZFPM2* | | | zinc finger protein, FOG family member 2 | | -2.51 | 7.8x10^-5^ |
| 225567_at |  | | |  | | -2.51 | 0.00124 |
| 1559275_x_at | *ZNF732* | | | zinc finger protein 732 | | -2.51 | 0.00134 |
| 206554_x_at | *SETMAR* | | | SET domain and mariner transposase fusion gene | | -2.51 | 0.00163 |
| 224016_at | *HIPK2* | | | homeodomain interacting protein kinase 2 | | -2.51 | 0.00188 |
| 224938_at | *NUFIP2* | | | nuclear fragile X mental retardation protein interacting protein 2 | | -2.51 | 0.00206 |
| 212580_at | *CAST* | | | calpastatin | | -2.51 | 0.00207 |
| 214011_s_at | *NOP16* | | | NOP16 nucleolar protein | | -2.51 | 0.00294 |
| 209780_at | *PHTF2* | | | putative homeodomain transcription factor 2 | | -2.51 | 0.00331 |
| 55692_at | *ELMO2* | | | engulfment and cell motility 2 | | -2.51 | 0.00333 |
| 235620_x_at | *ZMYM5* | | | zinc finger, MYM-type 5 | | -2.51 | 0.00444 |
| 238011_at |  | | |  | | -2.51 | 0.00535 |
| 224320_s_at | *MCM8* | | | minichromosome maintenance complex component 8 | | -2.51 | 0.00637 |
| 218545_at | *CCDC91* | | | coiled-coil domain containing 91 | | -2.51 | 0.00734 |
| 212842_x_at | *RGPD3* | | | RANBP2-like and GRIP domain containing 3; RANBP2-like and GRIP domain containing 4; RANBP2-like and GRIP domain containing 5; RANBP2-like and GRIP domain containing 6; RANBP2-like and GRIP domain containing 8 | | -2.51 | 0.00781 |
| 211352_s_at | *NCOA3* | | | nuclear receptor coactivator 3 | | -2.51 | 0.00812 |
| 204409_s_at | *EIF1AY* | | | eukaryotic translation initiation factor 1A, Y-linked | | -2.51 | 0.00861 |
| 232125_at |  | | |  | | -2.51 | 0.0097 |
| 1554915_a_at | *PDE12* | | | phosphodiesterase 12 | | -2.51 | 0.01132 |
| 242059_at |  | | |  | | -2.51 | 0.01201 |
| 205053_at | *PRIM1* | | | primase, DNA, polypeptide 1 (49kDa) | | -2.51 | 0.01267 |
| 244050_at | *PTPLAD2* | | | protein tyrosine phosphatase-like A domain containing 2 | | -2.51 | 0.01386 |
| 219637_at | *ARMC9* | | | armadillo repeat containing 9 | | -2.51 | 0.01553 |
| 224368_s_at | *NDRG3* | | | NDRG family member 3 | | -2.51 | 0.01889 |
| 212320_at | *TUBB* | | | tubulin, beta class I | | -2.51 | 0.01913 |
| 1561180_at |  | | |  | | -2.51 | 0.02264 |
| 228767_at |  | | |  | | -2.51 | 0.02387 |
| 1560648_s_at | *TSPYL1* | | | TSPY-like 1 | | -2.51 | 0.02648 |
| 216632_at | *NAV3* | | | neuron navigator 3 | | -2.51 | 0.03817 |
| 210473_s_at | *GPR125* | | | G protein-coupled receptor 125 | | -2.52 | 0.00035 |
| 206029_at | *ANKRD1* | | | ankyrin repeat domain 1 (cardiac muscle) | | -2.52 | 0.00079 |
| 217921_at | *MAN1A2* | | | mannosidase, alpha, class 1A, member 2 | | -2.52 | 0.00231 |
| 232090_at | *DNM3OS* | | | DNM3 opposite strand/antisense RNA | | -2.52 | 0.00314 |
| 226092_at | *MPP5* | | | membrane protein, palmitoylated 5 (MAGUK p55 subfamily member 5) | | -2.52 | 0.00421 |
| 217820_s_at | *ENAH* | | | enabled homolog (Drosophila) | | -2.52 | 0.00435 |
| 229460_at | *FAM126B* | | | family with sequence similarity 126, member B | | -2.52 | 0.00489 |
| 233085_s_at | *NABP1* | | | nucleic acid binding protein 1 | | -2.52 | 0.00514 |
| 202630_at | *APPBP2* | | | amyloid beta precursor protein (cytoplasmic tail) binding protein 2 | | -2.52 | 0.0075 |
| 203139_at | *DAPK1* | | | death-associated protein kinase 1 | | -2.52 | 0.00882 |
| 203397_s_at | *GALNT3* | | | UDP-N-acetyl-alpha-D-galactosamine:polypeptide N-acetylgalactosaminyltransferase 3 (GalNAc-T3) | | -2.52 | 0.00923 |
| 205618_at | *PRRG1* | | | proline rich Gla (G-carboxyglutamic acid) 1 | | -2.52 | 0.01231 |
| 228597_at | *MIS18A* | | | MIS18 kinetochore protein homolog A (S. pombe) | | -2.52 | 0.01711 |
| 206613_s_at | *TAF1A* | | | TATA box binding protein (TBP)-associated factor, RNA polymerase I, A, 48kDa | | -2.52 | 0.02002 |
| 227636_at | *THAP5* | | | THAP domain containing 5 | | -2.52 | 0.02437 |
| 225655_at | *UHRF1* | | | ubiquitin-like with PHD and ring finger domains 1 | | -2.53 | 0.00016 |
| 200052_s_at | *ILF2* | | | interleukin enhancer binding factor 2 | | -2.53 | 0.00026 |
| 222250_s_at | *INTS7* | | | integrator complex subunit 7 | | -2.53 | 0.00038 |
| 203964_at | *NMI* | | | N-myc (and STAT) interactor | | -2.53 | 0.00063 |
| 218819_at | *INTS6* | | | integrator complex subunit 6 | | -2.53 | 0.00102 |
| 204141_at | *TUBB2A* | | | tubulin, beta 2A class IIa | | -2.53 | 0.00195 |
| 242618_at | *HCG18* | | | HLA complex group 18 (non-protein coding) | | -2.53 | 0.00231 |
| 214710_s_at | *CCNB1* | | | cyclin B1 | | -2.53 | 0.00232 |
| 241925_x_at | *SLC16A7* | | | solute carrier family 16, member 7 (monocarboxylic acid transporter 2) | | -2.53 | 0.00256 |
| 200918_s_at | *SRPR* | | | signal recognition particle receptor (docking protein) | | -2.53 | 0.00296 |
| 223418_x_at | *ANKRD13C* | | | ankyrin repeat domain 13C | | -2.53 | 0.00332 |
| 202829_s_at | *VAMP7* | | | vesicle-associated membrane protein 7 | | -2.53 | 0.0036 |
| 215438_x_at | *GSPT1* | | | G1 to S phase transition 1 | | -2.53 | 0.00416 |
| 217812_at | *YTHDF2* | | | YTH domain family, member 2 | | -2.53 | 0.00436 |
| 231899_at | *ZC3H12C* | | | zinc finger CCCH-type containing 12C | | -2.53 | 0.00512 |
| 1557984_s_at | *RPAP3* | | | RNA polymerase II associated protein 3 | | -2.53 | 0.00731 |
| 207213_s_at | *USP2* | | | ubiquitin specific peptidase 2 | | -2.53 | 0.00733 |
| 202059_s_at | *KPNA1* | | | karyopherin alpha 1 (importin alpha 5) | | -2.53 | 0.00745 |
| 200718_s_at | *SKP1* | | | S-phase kinase-associated protein 1 | | -2.53 | 0.00786 |
| 222316_at |  | | |  | | -2.53 | 0.00949 |
| 205703_at |  | | |  | | -2.53 | 0.01124 |
| 217645_at | *COX16* | | | COX16 cytochrome c oxidase assembly homolog (S. cerevisiae); SYNJ2BP-COX16 readthrough | | -2.53 | 0.01282 |
| 223520_s_at | *KIF13A* | | | kinesin family member 13A | | -2.53 | 0.02054 |
| 227761_at | *MYO5A* | | | myosin VA (heavy chain 12, myoxin) | | -2.53 | 0.0227 |
| 219743_at | *HEY2* | | | hairy/enhancer-of-split related with YRPW motif 2 | | -2.53 | 0.02624 |
| 219274_at | *TSPAN12* | | | tetraspanin 12 | | -2.53 | 0.03897 |
| 205609_at | *ANGPT1* | | | angiopoietin 1 | | -2.54 | 8.7x10^-5^ |
| 226233_at | *B3GALNT2* | | | beta-1,3-N-acetylgalactosaminyltransferase 2 | | -2.54 | 0.00074 |
| 212148_at | *PBX1* | | | pre-B-cell leukemia homeobox 1 | | -2.54 | 0.00116 |
| 1555973_at |  | | |  | | -2.54 | 0.00116 |
| 205885_s_at | *ITGA4* | | | integrin, alpha 4 (antigen CD49D, alpha 4 subunit of VLA-4 receptor) | | -2.54 | 0.0014 |
| 213449_at | *POP1* | | | processing of precursor 1, ribonuclease P/MRP subunit (S. cerevisiae) | | -2.54 | 0.00148 |
| 202020_s_at | *LANCL1* | | | LanC lantibiotic synthetase component C-like 1 (bacterial) | | -2.54 | 0.00175 |
| 204873_at | *PEX1* | | | peroxisomal biogenesis factor 1 | | -2.54 | 0.00185 |
| 201663_s_at | *SMC4* | | | structural maintenance of chromosomes 4 | | -2.54 | 0.00188 |
| 212314_at | *SEL1L3* | | | sel-1 suppressor of lin-12-like 3 (C. elegans) | | -2.54 | 0.00212 |
| 235189_at | *NARG2* | | | NMDA receptor regulated 2 | | -2.54 | 0.0024 |
| 203203_s_at | *KRR1* | | | KRR1, small subunit (SSU) processome component, homolog (yeast) | | -2.54 | 0.00446 |
| 218791_s_at | *KATNBL1* | | | katanin p80 subunit B-like 1 | | -2.54 | 0.00493 |
| 205080_at | *RARB* | | | retinoic acid receptor, beta | | -2.54 | 0.00493 |
| 205533_s_at | *CDH6* | | | cadherin 6, type 2, K-cadherin (fetal kidney) | | -2.54 | 0.00558 |
| 235453_at | *TOR1AIP2* | | | torsin A interacting protein 2 | | -2.54 | 0.00613 |
| 235296_at | *EIF5A2* | | | eukaryotic translation initiation factor 5A2 | | -2.54 | 0.00686 |
| 221492_s_at | *ATG3* | | | autophagy related 3 | | -2.54 | 0.00841 |
| 225679_at | *NAA30* | | | N(alpha)-acetyltransferase 30, NatC catalytic subunit | | -2.54 | 0.00845 |
| 217777_s_at | *PTPLAD1* | | | protein tyrosine phosphatase-like A domain containing 1 | | -2.54 | 0.00848 |
| 1559343_at |  | | |  | | -2.54 | 0.00921 |
| 203077_s_at | *SMAD2* | | | SMAD family member 2 | | -2.54 | 0.00975 |
| 229333_at |  | | |  | | -2.54 | 0.01149 |
| 219178_at | *QTRTD1* | | | queuine tRNA-ribosyltransferase domain containing 1 | | -2.54 | 0.01287 |
| 223238_s_at | *PBRM1* | | | polybromo 1 | | -2.54 | 0.0145 |
| 239859_x_at | *ATP5S* | | | ATP synthase, H+ transporting, mitochondrial Fo complex, subunit s (factor B) | | -2.54 | 0.01573 |
| 234154_at |  | | |  | | -2.54 | 0.02944 |
| 210320_s_at | *DDX52* | | | DEAD (Asp-Glu-Ala-Asp) box polypeptide 52 | | -2.55 | 0.00056 |
| 228490_at | *ABHD2* | | | abhydrolase domain containing 2 | | -2.55 | 0.00095 |
| 1568720_at | *ZNF506* | | | zinc finger protein 506 | | -2.55 | 0.00114 |
| 203502_at | *BPGM* | | | 2,3-bisphosphoglycerate mutase | | -2.55 | 0.00355 |
| 213102_at | *ACTR3* | | | ARP3 actin-related protein 3 homolog (yeast) | | -2.55 | 0.00428 |
| 230708_at | *PRICKLE1* | | | prickle homolog 1 (Drosophila) | | -2.55 | 0.00439 |
| 201074_at | *SMARCC1* | | | SWI/SNF related, matrix associated, actin dependent regulator of chromatin, subfamily c, member 1 | | -2.55 | 0.00525 |
| 1556097_at | *HOMER2* | | | homer homolog 2 (Drosophila) | | -2.55 | 0.00676 |
| 204675_at | *SRD5A1* | | | steroid-5-alpha-reductase, alpha polypeptide 1 (3-oxo-5 alpha-steroid delta 4-dehydrogenase alpha 1) | | -2.55 | 0.00834 |
| 200056_s_at | *C1D* | | | C1D nuclear receptor corepressor | | -2.55 | 0.00859 |
| 206158_s_at | *CNBP* | | | CCHC-type zinc finger, nucleic acid binding protein | | -2.55 | 0.00962 |
| 225084_at | *EXOC5* | | | exocyst complex component 5 | | -2.55 | 0.01108 |
| 203260_at | *HDDC2* | | | HD domain containing 2 | | -2.55 | 0.01152 |
| 213578_at | *BMPR1A* | | | bone morphogenetic protein receptor, type IA | | -2.55 | 0.01948 |
| 221152_at | *COL8A1* | | | collagen, type VIII, alpha 1 | | -2.55 | 0.0213 |
| 235093_at | *PEX13* | | | peroxisomal biogenesis factor 13 | | -2.55 | 0.02458 |
| 213000_at | *MORC3* | | | MORC family CW-type zinc finger 3 | | -2.55 | 0.04242 |
| 221090_s_at | *OGFOD1* | | | 2-oxoglutarate and iron-dependent oxygenase domain containing 1 | | -2.56 | 0.00071 |
| 221622_s_at | *TMEM126B* | | | transmembrane protein 126B | | -2.56 | 0.00086 |
| 216379_x_at | *CD24* | | | CD24 molecule | | -2.56 | 0.00149 |
| 205541_s_at | *GSPT2* | | | G1 to S phase transition 2 | | -2.56 | 0.00188 |
| 227036_at | *RASAL2* | | | RAS protein activator like 2 | | -2.56 | 0.00204 |
| 203427_at | *ASF1A* | | | anti-silencing function 1A histone chaperone | | -2.56 | 0.00213 |
| 243529_at | *MARS2* | | | methionyl-tRNA synthetase 2, mitochondrial | | -2.56 | 0.00322 |
| 228155_at | *FAM213A* | | | family with sequence similarity 213, member A | | -2.56 | 0.00453 |
| 207688_s_at |  | | |  | | -2.56 | 0.00611 |
| 212043_at | *TGOLN2* | | | trans-golgi network protein 2 | | -2.56 | 0.00672 |
| 218381_s_at | *U2AF2* | | | U2 small nuclear RNA auxiliary factor 2 | | -2.56 | 0.00864 |
| 241887_at | *UBE2W* | | | ubiquitin-conjugating enzyme E2W (putative) | | -2.56 | 0.02058 |
| 224873_s_at | *MRPS25* | | | mitochondrial ribosomal protein S25 | | -2.56 | 0.02333 |
| 226635_at |  | | |  | | -2.56 | 0.02999 |
| 228242_at | *N4BP2* | | | NEDD4 binding protein 2 | | -2.56 | 0.03094 |
| 213224_s_at | *BBIP1* | | | BBSome interacting protein 1 | | -2.56 | 0.0374 |
| 213984_at | *PDS5A* | | | PDS5, regulator of cohesion maintenance, homolog A (S. cerevisiae) | | -2.56 | 0.03767 |
| 212835_at | *FAM175B* | | | family with sequence similarity 175, member B | | -2.57 | 2.1x10^-5^ |
| 201197_at | *AMD1* | | | adenosylmethionine decarboxylase 1 | | -2.57 | 0.00131 |
| 218817_at | *SPCS3* | | | signal peptidase complex subunit 3 homolog (S. cerevisiae) | | -2.57 | 0.00148 |
| 230468_s_at | *C1orf56* | | | chromosome 1 open reading frame 56 | | -2.57 | 0.00153 |
| 229744_at | *SSFA2* | | | sperm specific antigen 2 | | -2.57 | 0.00173 |
| 201724_s_at | *GALNT1* | | | UDP-N-acetyl-alpha-D-galactosamine:polypeptide N-acetylgalactosaminyltransferase 1 (GalNAc-T1) | | -2.57 | 0.00236 |
| 204463_s_at | *EDNRA* | | | endothelin receptor type A | | -2.57 | 0.00237 |
| 225473_at | *SOGA1* | | | suppressor of glucose, autophagy associated 1 | | -2.57 | 0.00266 |
| 201487_at | *CTSC* | | | cathepsin C | | -2.57 | 0.00373 |
| 235141_at | *MARVELD2* | | | MARVEL domain containing 2 | | -2.57 | 0.00485 |
| 223071_at | *IER3IP1* | | | immediate early response 3 interacting protein 1 | | -2.57 | 0.00606 |
| 220346_at | *MTHFD2L* | | | methylenetetrahydrofolate dehydrogenase (NADP+ dependent) 2-like | | -2.57 | 0.00697 |
| 215983_s_at | *UBXN8* | | | UBX domain protein 8 | | -2.57 | 0.00969 |
| 1555971_s_at | *FBXO28* | | | F-box protein 28 | | -2.57 | 0.01066 |
| 243303_at |  | | |  | | -2.57 | 0.01112 |
| 226501_at | *XPNPEP3* | | | X-prolyl aminopeptidase (aminopeptidase P) 3, putative | | -2.57 | 0.0132 |
| 214587_at | *COL8A1* | | | collagen, type VIII, alpha 1 | | -2.57 | 0.02229 |
| 207703_at | *NLGN4Y* | | | neuroligin 4, Y-linked | | -2.57 | 0.02583 |
| 235549_at | *RNF144B* | | | ring finger protein 144B | | -2.57 | 0.03221 |
| 235036_at | *LIX1L* | | | Lix1 homolog (mouse)-like; LIX1-like protein-like | | -2.57 | 0.03235 |
| 239486_at |  | | |  | | -2.58 | 0.00035 |
| 201821_s_at | *TIMM17A* | | | translocase of inner mitochondrial membrane 17 homolog A (yeast) | | -2.58 | 0.00161 |
| 203418_at | *CCNA2* | | | cyclin A2 | | -2.58 | 0.00171 |
| 223595_at | *TMEM133* | | | transmembrane protein 133 | | -2.58 | 0.00249 |
| 232515_at |  | | |  | | -2.58 | 0.00296 |
| 217786_at | *PRMT5* | | | protein arginine methyltransferase 5 | | -2.58 | 0.00304 |
| 230175_s_at | *DCBLD2* | | | discoidin, CUB and LCCL domain containing 2 | | -2.58 | 0.00366 |
| 231984_at | *MTAP* | | | methylthioadenosine phosphorylase | | -2.58 | 0.00371 |
| 212149_at | *EFR3A* | | | EFR3 homolog A (S. cerevisiae) | | -2.58 | 0.00429 |
| 218782_s_at | *ATAD2* | | | ATPase family, AAA domain containing 2 | | -2.58 | 0.0048 |
| 228407_at | *SCUBE3* | | | signal peptide, CUB domain, EGF-like 3 | | -2.58 | 0.00531 |
| 218568_at | *AGK* | | | acylglycerol kinase | | -2.58 | 0.00644 |
| 202754_at | *R3HDM1* | | | R3H domain containing 1 | | -2.58 | 0.00742 |
| 203156_at | *AKAP11* | | | A kinase (PRKA) anchor protein 11 | | -2.58 | 0.0084 |
| 223180_s_at | *TIMM21* | | | translocase of inner mitochondrial membrane 21 homolog (yeast) | | -2.58 | 0.00843 |
| 226525_at | *STK17B* | | | serine/threonine kinase 17b | | -2.58 | 0.0092 |
| 221963_x_at | *ZNF587B* | | | zinc finger protein 587B | | -2.58 | 0.0092 |
| 227542_at | *SOCS6* | | | suppressor of cytokine signaling 6 | | -2.58 | 0.01191 |
| 227004_at |  | | |  | | -2.58 | 0.01223 |
| 202413_s_at | *USP1* | | | ubiquitin specific peptidase 1 | | -2.58 | 0.01301 |
| 227754_at |  | | |  | | -2.58 | 0.01305 |
| 208738_x_at | *SUMO2* | | | small ubiquitin-like modifier 2 | | -2.58 | 0.01533 |
| 221788_at | *PGM3* | | | phosphoglucomutase 3 | | -2.58 | 0.01611 |
| 239138_at | *NAA50* | | | N(alpha)-acetyltransferase 50, NatE catalytic subunit | | -2.58 | 0.01733 |
| 206364_at | *KIF14* | | | kinesin family member 14 | | -2.58 | 0.02203 |
| 227973_at | *C2orf69* | | | chromosome 2 open reading frame 69 | | -2.58 | 0.03787 |
| 214820_at | *BRWD1* | | | bromodomain and WD repeat domain containing 1 | | -2.58 | 0.03981 |
| 226898_s_at | *SFPQ* | | | splicing factor proline/glutamine-rich | | -2.59 | 0.0001 |
| 203596_s_at | *IFIT5* | | | interferon-induced protein with tetratricopeptide repeats 5 | | -2.59 | 0.00014 |
| 210154_at | *ME2* | | | malic enzyme 2, NAD(+)-dependent, mitochondrial | | -2.59 | 0.00019 |
| 217578_at |  | | |  | | -2.59 | 0.00045 |
| 231064_s_at | *NUP50* | | | nucleoporin 50kDa | | -2.59 | 0.00076 |
| 1558691_a_at | *DOCK4* | | | dedicator of cytokinesis 4 | | -2.59 | 0.00078 |
| 202199_s_at | *SRPK1* | | | SRSF protein kinase 1 | | -2.59 | 0.00103 |
| 225581_s_at | *MRPL50* | | | mitochondrial ribosomal protein L50 | | -2.59 | 0.00304 |
| 212799_at | *STX6* | | | syntaxin 6 | | -2.59 | 0.00448 |
| 200033_at | *DDX5* | | | DEAD (Asp-Glu-Ala-Asp) box helicase 5; microRNA 3064; microRNA 5047 | | -2.59 | 0.00497 |
| 222815_at | *RLIM* | | | ring finger protein, LIM domain interacting | | -2.59 | 0.00519 |
| 210076_x_at | *SERBP1* | | | SERPINE1 mRNA binding protein 1 | | -2.59 | 0.00645 |
| 242623_x_at | *PSMG4* | | | proteasome (prosome, macropain) assembly chaperone 4 | | -2.59 | 0.00694 |
| 204299_at |  | | |  | | -2.59 | 0.007 |
| 226568_at | *FAM102B* | | | family with sequence similarity 102, member B | | -2.59 | 0.00936 |
| 225426_at | *PPP6C* | | | protein phosphatase 6, catalytic subunit | | -2.59 | 0.00988 |
| 1560144_at |  | | |  | | -2.59 | 0.01011 |
| 224731_at | *HMGB1* | | | high mobility group box 1 | | -2.59 | 0.01337 |
| 235384_at | *NUDT19* | | | nudix (nucleoside diphosphate linked moiety X)-type motif 19 | | -2.59 | 0.01489 |
| 223035_s_at | *FARSB* | | | phenylalanyl-tRNA synthetase, beta subunit | | -2.6 | 0.00045 |
| 208767_s_at | *LAPTM4B* | | | lysosomal protein transmembrane 4 beta | | -2.6 | 0.00083 |
| 212145_at | *MRPS27* | | | mitochondrial ribosomal protein S27 | | -2.6 | 0.00117 |
| 219074_at | *TMEM184C* | | | transmembrane protein 184C | | -2.6 | 0.00218 |
| 227310_at | *ADSS* | | | adenylosuccinate synthase | | -2.6 | 0.00248 |
| 212956_at | *TBC1D9* | | | TBC1 domain family, member 9 (with GRAM domain) | | -2.6 | 0.00298 |
| 222774_s_at | *NETO2* | | | neuropilin (NRP) and tolloid (TLL)-like 2 | | -2.6 | 0.00318 |
| 212930_at | *ATP2B1* | | | ATPase, Ca++ transporting, plasma membrane 1 | | -2.6 | 0.00482 |
| 1555974_a_at |  | | |  | | -2.6 | 0.00634 |
| 219276_x_at | *CAAP1* | | | caspase activity and apoptosis inhibitor 1 | | -2.6 | 0.01315 |
| 208724_s_at | *RAB1A* | | | RAB1A, member RAS oncogene family | | -2.6 | 0.01555 |
| 212572_at | *STK38L* | | | serine/threonine kinase 38 like | | -2.6 | 0.01716 |
| 201202_at | *PCNA* | | | proliferating cell nuclear antigen | | -2.6 | 0.01743 |
| 228152_s_at | *DDX60L* | | | DEAD (Asp-Glu-Ala-Asp) box polypeptide 60-like | | -2.6 | 0.03116 |
| 1554016_a_at | *USB1* | | | U6 snRNA biogenesis 1 | | -2.61 | 0.00031 |
| 208844_at | *VDAC3* | | | voltage-dependent anion channel 3 | | -2.61 | 0.00039 |
| 223275_at | *PRMT6* | | | protein arginine methyltransferase 6 | | -2.61 | 0.0015 |
| 212438_at | *SNRNP27* | | | small nuclear ribonucleoprotein 27kDa (U4/U6.U5) | | -2.61 | 0.0018 |
| 214161_at | *OSGIN2* | | | oxidative stress induced growth inhibitor family member 2 | | -2.61 | 0.00266 |
| 225088_at | *FOPNL* | | | FGFR1OP N-terminal like | | -2.61 | 0.00324 |
| 223667_at | *FKBP7* | | | FK506 binding protein 7 | | -2.61 | 0.00833 |
| 232715_at |  | | |  | | -2.61 | 0.00841 |
| 1569706_at | *MYSM1* | | | Myb-like, SWIRM and MPN domains 1 | | -2.61 | 0.01257 |
| 235906_at | *TAF10* | | | TAF10 RNA polymerase II, TATA box binding protein (TBP)-associated factor, 30kDa | | -2.61 | 0.01451 |
| 218311_at | *MAP4K3* | | | mitogen-activated protein kinase kinase kinase kinase 3 | | -2.61 | 0.01517 |
| 211946_s_at | *PRRC2C* | | | proline-rich coiled-coil 2C | | -2.61 | 0.01553 |
| 217975_at | *WBP5* | | | WW domain binding protein 5 | | -2.61 | 0.01993 |
| 228468_at | *MASTL* | | | microtubule associated serine/threonine kinase-like | | -2.61 | 0.02001 |
| 224844_at | *SLAIN2* | | | SLAIN motif family, member 2 | | -2.61 | 0.02228 |
| 228408_s_at | *SDAD1* | | | SDA1 domain containing 1 | | -2.62 | 0.00119 |
| 222761_at | *BIVM* | | | basic, immunoglobulin-like variable motif containing | | -2.62 | 0.00178 |
| 212643_at | *MAPK1IP1L* | | | mitogen-activated protein kinase 1 interacting protein 1-like | | -2.62 | 0.00294 |
| 205173_x_at | *CD58* | | | CD58 molecule | | -2.62 | 0.00494 |
| 228913_at | *TPT1-AS1* | | | TPT1 antisense RNA 1 | | -2.62 | 0.00573 |
| 202903_at | *LSM5* | | | LSM5 homolog, U6 small nuclear RNA associated (S. cerevisiae) | | -2.62 | 0.00606 |
| 213628_at | *CLCC1* | | | chloride channel CLIC-like 1 | | -2.62 | 0.00621 |
| 204439_at | *IFI44L* | | | interferon-induced protein 44-like | | -2.62 | 0.00624 |
| 226604_at | *TMTC3* | | | transmembrane and tetratricopeptide repeat containing 3 | | -2.62 | 0.00649 |
| 203449_s_at | *TERF1* | | | telomeric repeat binding factor (NIMA-interacting) 1 | | -2.62 | 0.00879 |
| 215096_s_at | *ESD* | | | esterase D | | -2.62 | 0.00884 |
| 222478_at | *VPS36* | | | vacuolar protein sorting 36 homolog (S. cerevisiae) | | -2.62 | 0.0095 |
| 212615_at | *CHD9* | | | chromodomain helicase DNA binding protein 9 | | -2.62 | 0.01431 |
| 222438_at | *MED4* | | | mediator complex subunit 4 | | -2.62 | 0.02007 |
| 213168_at | *SP3* | | | Sp3 transcription factor | | -2.62 | 0.02198 |
| 208995_s_at | *PPIG* | | | peptidylprolyl isomerase G (cyclophilin G) | | -2.62 | 0.03121 |
| 203875_at | *SMARCA1* | | | SWI/SNF related, matrix associated, actin dependent regulator of chromatin, subfamily a, member 1 | | -2.62 | 0.03245 |
| 221840_at | *PTPRE* | | | protein tyrosine phosphatase, receptor type, E | | -2.63 | 0.00025 |
| 201193_at | *IDH1* | | | isocitrate dehydrogenase 1 (NADP+), soluble | | -2.63 | 0.00046 |
| 229800_at | *DCLK1* | | | doublecortin-like kinase 1 | | -2.63 | 0.00067 |
| 220115_s_at | *CDH10* | | | cadherin 10, type 2 (T2-cadherin) | | -2.63 | 0.00115 |
| 212943_at | *C2CD5* | | | C2 calcium-dependent domain containing 5 | | -2.63 | 0.00405 |
| 1554178_a_at | *FAM126B* | | | family with sequence similarity 126, member B | | -2.63 | 0.00415 |
| 209142_s_at | *UBE2G1* | | | ubiquitin-conjugating enzyme E2G 1 | | -2.63 | 0.00467 |
| 202872_at | *ATP6V1C1* | | | ATPase, H+ transporting, lysosomal 42kDa, V1 subunit C1 | | -2.63 | 0.00475 |
| 237411_at | *ADAMTS6* | | | ADAM metallopeptidase with thrombospondin type 1 motif, 6 | | -2.63 | 0.00524 |
| 213743_at | *CCNT2* | | | cyclin T2 | | -2.63 | 0.00678 |
| 32069_at | *N4BP1* | | | NEDD4 binding protein 1 | | -2.63 | 0.00757 |
| 212440_at | *SNRNP27* | | | small nuclear ribonucleoprotein 27kDa (U4/U6.U5) | | -2.63 | 0.00778 |
| 225666_at | *TMTC4* | | | transmembrane and tetratricopeptide repeat containing 4 | | -2.63 | 0.00915 |
| 226360_at | *ZNRF3* | | | zinc and ring finger 3 | | -2.63 | 0.01004 |
| 235311_at | *FKBP14* | | | FK506 binding protein 14, 22 kDa | | -2.63 | 0.01946 |
| 200995_at | *IPO7* | | | importin 7 | | -2.63 | 0.04579 |
| 228661_s_at |  | | |  | | -2.64 | 0.00021 |
| 201992_s_at | *KIF5B* | | | kinesin family member 5B | | -2.64 | 0.00046 |
| 228955_at | *LRP8* | | | low density lipoprotein receptor-related protein 8, apolipoprotein e receptor | | -2.64 | 0.00049 |
| 200011_s_at | *ARF3* | | | ADP-ribosylation factor 3 | | -2.64 | 0.00149 |
| 243948_at |  | | |  | | -2.64 | 0.00167 |
| 202918_s_at | *HSPE1-MOB4* | | | HSPE1-MOB4 readthrough; MOB family member 4, phocein | | -2.64 | 0.00202 |
| 235113_at | *LRR1* | | | leucine rich repeat protein 1 | | -2.64 | 0.00244 |
| 213610_s_at | *KLHL23* | | | kelch-like family member 23; PHOSPHO2-KLHL23 readthrough | | -2.64 | 0.00248 |
| 219813_at | *LATS1* | | | large tumor suppressor kinase 1 | | -2.64 | 0.00463 |
| 230127_at |  | | |  | | -2.64 | 0.00545 |
| 208732_at | *RAB2A* | | | RAB2A, member RAS oncogene family | | -2.64 | 0.00624 |
| 225225_at | *OIP5-AS1* | | | OIP5 antisense RNA 1 | | -2.64 | 0.00742 |
| 223455_at | *TCHP* | | | trichoplein, keratin filament binding | | -2.64 | 0.00911 |
| 201857_at | *ZFR* | | | zinc finger RNA binding protein | | -2.64 | 0.01098 |
| 218256_s_at | *NUP54* | | | nucleoporin 54kDa | | -2.64 | 0.01452 |
| 1554095_at | *RBM33* | | | RNA binding motif protein 33 | | -2.64 | 0.01693 |
| 218871_x_at | *CSGALNACT2* | | | chondroitin sulfate N-acetylgalactosaminyltransferase 2 | | -2.64 | 0.01977 |
| 202127_at | *PRPF4B* | | | PRP4 pre-mRNA processing factor 4 homolog B (yeast) | | -2.64 | 0.02056 |
| 200605_s_at | *PRKAR1A* | | | protein kinase, cAMP-dependent, regulatory, type I, alpha | | -2.64 | 0.02726 |
| 225181_at | *ARID1B* | | | AT rich interactive domain 1B (SWI1-like) | | -2.64 | 0.02796 |
| 201930_at | *MCM6* | | | minichromosome maintenance complex component 6 | | -2.65 | 4.7x10^-5^ |
| 229589_x_at | *BIVM* | | | basic, immunoglobulin-like variable motif containing | | -2.65 | 0.00062 |
| 218229_s_at | *POGK* | | | pogo transposable element with KRAB domain | | -2.65 | 0.00097 |
| 228805_at | *SIMC1* | | | SUMO-interacting motifs containing 1 | | -2.65 | 0.0014 |
| 218090_s_at | *WDR11* | | | WD repeat domain 11 | | -2.65 | 0.00255 |
| 231990_at | *USP15* | | | ubiquitin specific peptidase 15 | | -2.65 | 0.00308 |
| 1556583_a_at | *SLC8A1* | | | solute carrier family 8 (sodium/calcium exchanger), member 1 | | -2.65 | 0.00337 |
| 200630_x_at | *SET* | | | SET nuclear oncogene | | -2.65 | 0.00339 |
| 222728_s_at | *MIR1304* | | | microRNA 1304; small nucleolar RNA, H/ACA box 1; small nucleolar RNA, H/ACA box 18; small nucleolar RNA, H/ACA box 32; small nucleolar RNA, H/ACA box 40; small nucleolar RNA, H/ACA box 8; small nucleolar RNA, C/D box 5; TATA box binding protein (TBP)-associated factor, RNA polymerase I, D, 41kDa | | -2.65 | 0.0036 |
| 201963_at | *ACSL1* | | | acyl-CoA synthetase long-chain family member 1 | | -2.65 | 0.00401 |
| 208673_s_at | *SRSF3* | | | serine/arginine-rich splicing factor 3 | | -2.65 | 0.00404 |
| 221923_s_at | *NPM1* | | | nucleophosmin (nucleolar phosphoprotein B23, numatrin) | | -2.65 | 0.0056 |
| 227462_at | *ERAP2* | | | endoplasmic reticulum aminopeptidase 2 | | -2.65 | 0.00647 |
| 221502_at | *KPNA3* | | | karyopherin alpha 3 (importin alpha 4) | | -2.65 | 0.00677 |
| 203494_s_at | *CEP57* | | | centrosomal protein 57kDa | | -2.65 | 0.00682 |
| 218940_at | *METTL21D* | | | methyltransferase like 21D | | -2.65 | 0.00692 |
| 223404_s_at | *TRMT1L* | | | tRNA methyltransferase 1 homolog (S. cerevisiae)-like | | -2.65 | 0.00727 |
| 223359_s_at | *PDE7A* | | | phosphodiesterase 7A | | -2.65 | 0.00737 |
| 225356_at |  | | |  | | -2.65 | 0.00765 |
| 217743_s_at | *TMEM30A* | | | transmembrane protein 30A | | -2.65 | 0.00875 |
| 202254_at | *SIPA1L1* | | | signal-induced proliferation-associated 1 like 1 | | -2.65 | 0.00896 |
| 207338_s_at | *ZNF200* | | | zinc finger protein 200 | | -2.65 | 0.01026 |
| 213391_at | *DPY19L4* | | | dpy-19-like 4 (C. elegans) | | -2.65 | 0.01081 |
| 229843_at | *RMDN1* | | | regulator of microtubule dynamics 1 | | -2.65 | 0.01429 |
| 230165_at | *SGOL2* | | | shugoshin-like 2 (S. pombe) | | -2.65 | 0.0149 |
| 235698_at | *ZFP90* | | | ZFP90 zinc finger protein | | -2.65 | 0.01624 |
| 201110_s_at | *THBS1* | | | thrombospondin 1 | | -2.65 | 0.01751 |
| 239825_at |  | | |  | | -2.65 | 0.03985 |
| 212633_at | *UFL1* | | | UFM1-specific ligase 1 | | -2.65 | 0.04109 |
| 204464_s_at | *EDNRA* | | | endothelin receptor type A | | -2.66 | 0.00069 |
| 211762_s_at | *KPNA2* | | | karyopherin alpha 2 (RAG cohort 1, importin alpha 1) | | -2.66 | 0.00093 |
| 225308_s_at | *TANC1* | | | tetratricopeptide repeat, ankyrin repeat and coiled-coil containing 1 | | -2.66 | 0.00143 |
| 203335_at | *PHYH* | | | phytanoyl-CoA 2-hydroxylase | | -2.66 | 0.0016 |
| 1552312_a_at | *MFAP3* | | | microfibrillar-associated protein 3 | | -2.66 | 0.00216 |
| 215498_s_at |  | | |  | | -2.66 | 0.00221 |
| 1552485_at | *LACTB* | | | lactamase, beta | | -2.66 | 0.00231 |
| 201023_at | *TAF7* | | | TAF7 RNA polymerase II, TATA box binding protein (TBP)-associated factor, 55kDa | | -2.66 | 0.004 |
| 208905_at | *CYCS* | | | cytochrome c, somatic | | -2.66 | 0.00407 |
| 224812_at | *HIBADH* | | | 3-hydroxyisobutyrate dehydrogenase | | -2.66 | 0.00444 |
| 218374_s_at | *C12orf4* | | | chromosome 12 open reading frame 4 | | -2.66 | 0.00666 |
| 217465_at | *NCKAP1* | | | NCK-associated protein 1 | | -2.66 | 0.00711 |
| 225462_at | *TMEM128* | | | transmembrane protein 128 | | -2.66 | 0.00755 |
| 209484_s_at | *NSL1* | | | NSL1, MIND kinetochore complex component, homolog (S. cerevisiae) | | -2.66 | 0.00975 |
| 204666_s_at | *SIKE1* | | | suppressor of IKBKE 1 | | -2.66 | 0.01024 |
| 211698_at | *EID1* | | | EP300 interacting inhibitor of differentiation 1 | | -2.66 | 0.01276 |
| 227351_at | *C16orf52* | | | chromosome 16 open reading frame 52; uncharacterized LOC101060634 | | -2.66 | 0.01667 |
| 226484_at | *ZBTB47* | | | zinc finger and BTB domain containing 47 | | -2.66 | 0.01849 |
| 1557667_at |  | | |  | | -2.66 | 0.01864 |
| 1566987_s_at | *LINC00674* | | | long intergenic non-protein coding RNA 674 | | -2.66 | 0.01901 |
| 203614_at | *UTP14C* | | | UTP14, U3 small nucleolar ribonucleoprotein, homolog C (yeast) | | -2.66 | 0.03077 |
| 239102_s_at |  | | |  | | -2.66 | 0.03249 |
| 232256_s_at |  | | |  | | -2.66 | 0.03952 |
| 1558801_at |  | | |  | | -2.66 | 0.04947 |
| 1555845_at |  | | |  | | -2.66 | 0.04965 |
| 208849_at |  | | |  | | -2.67 | 0.00018 |
| 201554_x_at | *GYG1* | | | glycogenin 1 | | -2.67 | 0.00151 |
| 204210_s_at | *PCYT1A* | | | phosphate cytidylyltransferase 1, choline, alpha | | -2.67 | 0.00176 |
| 219399_at | *LIN7C* | | | lin-7 homolog C (C. elegans) | | -2.67 | 0.00182 |
| 202582_s_at | *RANBP9* | | | RAN binding protein 9 | | -2.67 | 0.00284 |
| 226742_at | *SAR1B* | | | SAR1 homolog B (S. cerevisiae) | | -2.67 | 0.00294 |
| 200751_s_at | *HNRNPC* | | | heterogeneous nuclear ribonucleoprotein C (C1/C2) | | -2.67 | 0.0034 |
| 233485_at |  | | |  | | -2.67 | 0.00536 |
| 204709_s_at | *KIF23* | | | kinesin family member 23 | | -2.67 | 0.00656 |
| 206695_x_at | *ZNF43* | | | zinc finger protein 43 | | -2.67 | 0.00929 |
| 234759_at |  | | |  | | -2.67 | 0.01144 |
| 213852_at | *RBM8A* | | | RNA binding motif protein 8A | | -2.67 | 0.01245 |
| 231902_at | *ZNF827* | | | zinc finger protein 827 | | -2.67 | 0.01297 |
| 1555618_s_at | *SAE1* | | | SUMO1 activating enzyme subunit 1 | | -2.67 | 0.01465 |
| 229886_at | *C5orf34* | | | chromosome 5 open reading frame 34 | | -2.67 | 0.01707 |
| 244688_at |  | | |  | | -2.67 | 0.01996 |
| 218929_at | *CDKN2AIP* | | | CDKN2A interacting protein | | -2.67 | 0.02125 |
| 201872_s_at | *ABCE1* | | | ATP-binding cassette, sub-family E (OABP), member 1 | | -2.68 | 5.8x10^-5^ |
| 226392_at | *RASA2* | | | RAS p21 protein activator 2 | | -2.68 | 0.00146 |
| 1558675_s_at | *NEMF* | | | nuclear export mediator factor | | -2.68 | 0.0029 |
| 1555910_at | *PTCD2* | | | pentatricopeptide repeat domain 2 | | -2.68 | 0.0032 |
| 1566342_at | *SOD2* | | | superoxide dismutase 2, mitochondrial | | -2.68 | 0.00339 |
| 235615_at | *PGGT1B* | | | protein geranylgeranyltransferase type I, beta subunit | | -2.68 | 0.00508 |
| 213019_at | *RANBP6* | | | RAN binding protein 6 | | -2.68 | 0.00547 |
| 225539_at | *ZBTB21* | | | zinc finger and BTB domain containing 21 | | -2.68 | 0.00564 |
| 227312_at | *SNTB2* | | | syntrophin, beta 2 (dystrophin-associated protein A1, 59kDa, basic component 2) | | -2.68 | 0.0067 |
| 1554661_s_at | *CNST* | | | consortin, connexin sorting protein | | -2.68 | 0.0076 |
| 202664_at | *WIPF1* | | | WAS/WASL interacting protein family, member 1 | | -2.68 | 0.00928 |
| 244246_at | *MIPOL1* | | | mirror-image polydactyly 1 | | -2.68 | 0.01745 |
| 1553111_a_at | *KBTBD6* | | | kelch repeat and BTB (POZ) domain containing 6 | | -2.68 | 0.01783 |
| 1554930_a_at | *FUT8* | | | fucosyltransferase 8 (alpha (1,6) fucosyltransferase) | | -2.68 | 0.0191 |
| 235747_at | *LINC00849* | | | long intergenic non-protein coding RNA 849 | | -2.68 | 0.01976 |
| 222552_at | *GOLT1B* | | | golgi transport 1B | | -2.68 | 0.02038 |
| 201657_at | *ARL1* | | | ADP-ribosylation factor-like 1 | | -2.68 | 0.02746 |
| 231239_at |  | | |  | | -2.69 | 0.00011 |
| 218115_at | *ASF1B* | | | anti-silencing function 1B histone chaperone | | -2.69 | 0.00122 |
| 220353_at | *FAM86C1* | | | family with sequence similarity 86, member C1 | | -2.69 | 0.002 |
| 202647_s_at | *NRAS* | | | neuroblastoma RAS viral (v-ras) oncogene homolog | | -2.69 | 0.00226 |
| 201769_at | *CLINT1* | | | clathrin interactor 1 | | -2.69 | 0.00279 |
| 238935_at | *RPS27L* | | | ribosomal protein S27-like | | -2.69 | 0.00328 |
| 209096_at | *UBE2V2* | | | ubiquitin-conjugating enzyme E2 variant 2 | | -2.69 | 0.00444 |
| 218615_s_at | *TMEM39A* | | | transmembrane protein 39A | | -2.69 | 0.00453 |
| 211764_s_at | *UBE2D1* | | | ubiquitin-conjugating enzyme E2D 1 | | -2.69 | 0.00571 |
| 243000_at | *CDK6* | | | cyclin-dependent kinase 6 | | -2.69 | 0.00586 |
| 234332_at | *NUB1* | | | negative regulator of ubiquitin-like proteins 1 | | -2.69 | 0.00662 |
| 1552739_s_at | *ST7L* | | | suppression of tumorigenicity 7 like | | -2.69 | 0.00713 |
| 225687_at | *FAM83D* | | | family with sequence similarity 83, member D | | -2.69 | 0.01025 |
| 207029_at | *KITLG* | | | KIT ligand | | -2.69 | 0.01083 |
| 229025_s_at | *IMMP1L* | | | IMP1 inner mitochondrial membrane peptidase-like (S. cerevisiae) | | -2.69 | 0.01196 |
| 225970_at | *DDHD1* | | | DDHD domain containing 1 | | -2.69 | 0.01334 |
| 1558373_s_at |  | | |  | | -2.69 | 0.01629 |
| 214193_s_at | *DIEXF* | | | digestive organ expansion factor homolog (zebrafish) | | -2.69 | 0.01791 |
| 202933_s_at | *YES1* | | | v-yes-1 Yamaguchi sarcoma viral oncogene homolog 1 | | -2.69 | 0.02573 |
| 207738_s_at | *NCKAP1* | | | NCK-associated protein 1 | | -2.69 | 0.02761 |
| 1559601_at | *KIAA2018* | | | KIAA2018 | | -2.69 | 0.04896 |
| 217919_s_at | *MRPL42* | | | mitochondrial ribosomal protein L42 | | -2.7 | 0.00036 |
| 215171_s_at | *TIMM17A* | | | translocase of inner mitochondrial membrane 17 homolog A (yeast) | | -2.7 | 0.00066 |
| 204244_s_at | *DBF4* | | | DBF4 homolog (S. cerevisiae) | | -2.7 | 0.0011 |
| 205621_at | *ALKBH1* | | | alkB, alkylation repair homolog 1 (E. coli) | | -2.7 | 0.00243 |
| 221559_s_at | *MIS12* | | | MIS12, MIND kinetochore complex component, homolog (S. pombe) | | -2.7 | 0.00382 |
| 227531_at | *CLOCK* | | | clock circadian regulator | | -2.7 | 0.00409 |
| 236377_at | *TMEM132D* | | | transmembrane protein 132D | | -2.7 | 0.00447 |
| 228583_at | *LIN52* | | | lin-52 homolog (C. elegans) | | -2.7 | 0.00533 |
| 208801_at | *SRP72* | | | signal recognition particle 72kDa | | -2.7 | 0.00656 |
| 238462_at | *UBASH3B* | | | ubiquitin associated and SH3 domain containing B | | -2.7 | 0.00662 |
| 218098_at | *ARFGEF2* | | | ADP-ribosylation factor guanine nucleotide-exchange factor 2 (brefeldin A-inhibited) | | -2.7 | 0.01017 |
| 238756_at | *GAS2L3* | | | growth arrest-specific 2 like 3 | | -2.7 | 0.01027 |
| 231895_at | *SASS6* | | | spindle assembly 6 homolog (C. elegans) | | -2.7 | 0.01066 |
| 203356_at | *CAPN7* | | | calpain 7 | | -2.7 | 0.01344 |
| 1552487_a_at | *BNC1* | | | basonuclin 1 | | -2.7 | 0.01345 |
| 1558807_at | *ATAD2B* | | | ATPase family, AAA domain containing 2B | | -2.7 | 0.01518 |
| 222985_at | *YWHAG* | | | tyrosine 3-monooxygenase/tryptophan 5-monooxygenase activation protein, gamma polypeptide | | -2.7 | 0.01975 |
| 200969_at | *SERP1* | | | stress-associated endoplasmic reticulum protein 1 | | -2.7 | 0.02366 |
| 200603_at | *PRKAR1A* | | | protein kinase, cAMP-dependent, regulatory, type I, alpha | | -2.7 | 0.02422 |
| 224219_s_at | *TRPC4* | | | transient receptor potential cation channel, subfamily C, member 4 | | -2.71 | 7.6x10^-5^ |
| 221821_s_at | *KANSL2* | | | KAT8 regulatory NSL complex subunit 2 | | -2.71 | 0.001 |
| 242470_at | *EID2B* | | | EP300 interacting inhibitor of differentiation 2B | | -2.71 | 0.00319 |
| 227927_at |  | | |  | | -2.71 | 0.00339 |
| 218468_s_at | *GREM1* | | | gremlin 1, DAN family BMP antagonist | | -2.71 | 0.00433 |
| 212196_at | *IL6ST* | | | interleukin 6 signal transducer (gp130, oncostatin M receptor) | | -2.71 | 0.00473 |
| 215509_s_at | *BUB1* | | | BUB1 mitotic checkpoint serine/threonine kinase | | -2.71 | 0.00474 |
| 1555790_a_at | *TMEM192* | | | transmembrane protein 192; zinc finger protein 320 | | -2.71 | 0.00556 |
| 223491_at | *COMMD2* | | | COMM domain containing 2 | | -2.71 | 0.007 |
| 221773_at | *ELK3* | | | ELK3, ETS-domain protein (SRF accessory protein 2) | | -2.71 | 0.00855 |
| 218085_at | *CHMP5* | | | charged multivesicular body protein 5 | | -2.71 | 0.00949 |
| 221891_x_at | *HSPA8* | | | heat shock 70kDa protein 8; small nucleolar RNA, C/D box 14C; small nucleolar RNA, C/D box 14D | | -2.71 | 0.00998 |
| 1559151_at |  | | |  | | -2.71 | 0.01089 |
| 228789_at | *MTMR6* | | | myotubularin related protein 6 | | -2.71 | 0.01239 |
| 204523_at | *ZNF140* | | | zinc finger protein 140 | | -2.71 | 0.01241 |
| 222455_s_at | *PARVA* | | | parvin, alpha | | -2.71 | 0.01576 |
| 1560116_a_at | *NEDD1* | | | neural precursor cell expressed, developmentally down-regulated 1 | | -2.71 | 0.01745 |
| 204186_s_at | *PPID* | | | peptidylprolyl isomerase D | | -2.71 | 0.01933 |
| 226686_at | *CISD2* | | | CDGSH iron sulfur domain 2 | | -2.71 | 0.02125 |
| 214716_at | *BMP2K* | | | BMP2 inducible kinase | | -2.71 | 0.02271 |
| 212855_at | *DCUN1D4* | | | DCN1, defective in cullin neddylation 1, domain containing 4 | | -2.71 | 0.02406 |
| 212681_at | *EPB41L3* | | | erythrocyte membrane protein band 4.1-like 3 | | -2.71 | 0.03233 |
| 242346_x_at | *CTC-260E6.4* | | | NULL | | -2.71 | 0.04549 |
| 218877_s_at | *TRMT11* | | | tRNA methyltransferase 11 homolog (S. cerevisiae) | | -2.72 | 0.00022 |
| 219055_at | *SRBD1* | | | S1 RNA binding domain 1 | | -2.72 | 0.00062 |
| 224740_at | *SMIM15* | | | small integral membrane protein 15 | | -2.72 | 0.00062 |
| 222940_at | *SULT1E1* | | | sulfotransferase family 1E, estrogen-preferring, member 1 | | -2.72 | 0.00121 |
| 221733_s_at | *GPATCH4* | | | G patch domain containing 4 | | -2.72 | 0.00176 |
| 219134_at | *ELTD1* | | | EGF, latrophilin and seven transmembrane domain containing 1 | | -2.72 | 0.00214 |
| 226922_at | *RANBP2* | | | RAN binding protein 2 | | -2.72 | 0.00244 |
| 238057_at | *USP45* | | | ubiquitin specific peptidase 45 | | -2.72 | 0.00328 |
| 201595_s_at | *ZC3H15* | | | zinc finger CCCH-type containing 15 | | -2.72 | 0.00382 |
| 224941_at | *PAPPA* | | | pregnancy-associated plasma protein A, pappalysin 1 | | -2.72 | 0.00664 |
| 205074_at | *SLC22A5* | | | solute carrier family 22 (organic cation/carnitine transporter), member 5 | | -2.72 | 0.00773 |
| 205250_s_at | *CEP290* | | | centrosomal protein 290kDa | | -2.72 | 0.0082 |
| 229232_at | *LRRC57* | | | leucine rich repeat containing 57 | | -2.72 | 0.01044 |
| 203196_at | *ABCC4* | | | ATP-binding cassette, sub-family C (CFTR/MRP), member 4 | | -2.72 | 0.01133 |
| 203869_at | *USP46* | | | ubiquitin specific peptidase 46 | | -2.72 | 0.01151 |
| 224200_s_at | *RAD18* | | | RAD18 homolog (S. cerevisiae) | | -2.72 | 0.01287 |
| 232184_at | *ALS2* | | | amyotrophic lateral sclerosis 2 (juvenile) | | -2.72 | 0.01454 |
| 1566603_s_at | *RPUSD3* | | | RNA pseudouridylate synthase domain containing 3 | | -2.72 | 0.01554 |
| 225731_at | *ANKRD50* | | | ankyrin repeat domain 50 | | -2.72 | 0.01692 |
| 206458_s_at | *WNT2B* | | | wingless-type MMTV integration site family, member 2B | | -2.72 | 0.0194 |
| 215596_s_at | *LTN1* | | | listerin E3 ubiquitin protein ligase 1 | | -2.72 | 0.02265 |
| 229081_at | *SLC25A13* | | | solute carrier family 25 (aspartate/glutamate carrier), member 13 | | -2.73 | 0.00037 |
| 204977_at | *DDX10* | | | DEAD (Asp-Glu-Ala-Asp) box polypeptide 10 | | -2.73 | 0.00039 |
| 218658_s_at | *ACTR8* | | | ARP8 actin-related protein 8 homolog (yeast) | | -2.73 | 0.00114 |
| 212926_at | *SMC5* | | | structural maintenance of chromosomes 5 | | -2.73 | 0.00136 |
| 1558653_at | *MLK7-AS1* | | | MLK7 antisense RNA 1 | | -2.73 | 0.00276 |
| 222283_at | *ZNF480* | | | zinc finger protein 480 | | -2.73 | 0.00314 |
| 236974_at |  | | |  | | -2.73 | 0.00348 |
| 226294_x_at | *FAM91A1* | | | family with sequence similarity 91, member A1 | | -2.73 | 0.0037 |
| 210005_at | *GART* | | | phosphoribosylglycinamide formyltransferase, phosphoribosylglycinamide synthetase, phosphoribosylaminoimidazole synthetase | | -2.73 | 0.00372 |
| 235198_at | *OSTM1* | | | osteopetrosis associated transmembrane protein 1 | | -2.73 | 0.00549 |
| 224958_at | *NUFIP2* | | | nuclear fragile X mental retardation protein interacting protein 2 | | -2.73 | 0.00578 |
| 239170_at | *ACTR3* | | | ARP3 actin-related protein 3 homolog (yeast) | | -2.73 | 0.00621 |
| 210807_s_at | *SLC16A7* | | | solute carrier family 16, member 7 (monocarboxylic acid transporter 2) | | -2.73 | 0.00647 |
| 212585_at | *OSBPL8* | | | oxysterol binding protein-like 8 | | -2.73 | 0.00666 |
| 1567575_at |  | | |  | | -2.73 | 0.00685 |
| 204527_at | *MYO5A* | | | myosin VA (heavy chain 12, myoxin) | | -2.73 | 0.00866 |
| 213302_at | *PFAS* | | | phosphoribosylformylglycinamidine synthase | | -2.73 | 0.00886 |
| 218564_at | *RFWD3* | | | ring finger and WD repeat domain 3 | | -2.73 | 0.01169 |
| 210078_s_at | *KCNAB1* | | | potassium voltage-gated channel, shaker-related subfamily, beta member 1 | | -2.73 | 0.01532 |
| 1555653_at |  | | |  | | -2.73 | 0.02088 |
| 1554934_at | *RCBTB1* | | | regulator of chromosome condensation (RCC1) and BTB (POZ) domain containing protein 1 | | -2.73 | 0.02194 |
| 203910_at | *ARHGAP29* | | | Rho GTPase activating protein 29 | | -2.73 | 0.02284 |
| 203552_at | *MAP4K5* | | | mitogen-activated protein kinase kinase kinase kinase 5 | | -2.73 | 0.03028 |
| 1558142_at | *TNRC6B* | | | trinucleotide repeat containing 6B | | -2.73 | 0.03269 |
| 203328_x_at | *IDE* | | | insulin-degrading enzyme | | -2.74 | 0.00034 |
| 229181_s_at | *HAUS2* | | | HAUS augmin-like complex, subunit 2 | | -2.74 | 0.00148 |
| 209070_s_at | *RGS5* | | | regulator of G-protein signaling 5 | | -2.74 | 0.00153 |
| 1567527_at |  | | |  | | -2.74 | 0.00158 |
| 227926_s_at | *NBPF8P* | | | neuroblastoma breakpoint family, member 8, pseudogene; NULL | | -2.74 | 0.00179 |
| 228092_at | *CREM* | | | cAMP responsive element modulator | | -2.74 | 0.00211 |
| 204258_at | *CHD1* | | | chromodomain helicase DNA binding protein 1 | | -2.74 | 0.00303 |
| 210559_s_at | *CDK1* | | | cyclin-dependent kinase 1 | | -2.74 | 0.00354 |
| 221509_at | *DENR* | | | density-regulated protein | | -2.74 | 0.00544 |
| 205283_at | *FKTN* | | | fukutin | | -2.74 | 0.00652 |
| 1552619_a_at | *ANLN* | | | anillin, actin binding protein | | -2.74 | 0.00691 |
| 207219_at | *ZFP69B* | | | ZFP69 zinc finger protein B | | -2.74 | 0.00982 |
| 221543_s_at | *ERLIN2* | | | ER lipid raft associated 2 | | -2.74 | 0.01053 |
| 214315_x_at | *CALR* | | | calreticulin | | -2.74 | 0.01071 |
| 221193_s_at | *ZCCHC10* | | | zinc finger, CCHC domain containing 10 | | -2.74 | 0.01107 |
| 232020_at | *SMURF2* | | | SMAD specific E3 ubiquitin protein ligase 2 | | -2.74 | 0.01425 |
| 224436_s_at | *NIPSNAP3A* | | | nipsnap homolog 3A (C. elegans) | | -2.74 | 0.0187 |
| 232964_at | *SPDYE1* | | | speedy/RINGO cell cycle regulator family member E1; speedy/RINGO cell cycle regulator family member E5 | | -2.74 | 0.02075 |
| 1560474_at |  | | |  | | -2.74 | 0.02149 |
| 225227_at | *SKIL* | | | SKI-like oncogene | | -2.74 | 0.02323 |
| 222407_s_at | *ZNF106* | | | zinc finger protein 106 | | -2.74 | 0.03096 |
| 218888_s_at | *NETO2* | | | neuropilin (NRP) and tolloid (TLL)-like 2 | | -2.75 | 0.00083 |
| 211953_s_at | *IPO5* | | | importin 5 | | -2.75 | 0.00163 |
| 223434_at | *GBP3* | | | guanylate binding protein 3 | | -2.75 | 0.00274 |
| 212946_at | *VWA8* | | | von Willebrand factor A domain containing 8 | | -2.75 | 0.00548 |
| 1552675_at | *DNAJB7* | | | DnaJ (Hsp40) homolog, subfamily B, member 7 | | -2.75 | 0.00786 |
| 226169_at | *SBF2* | | | SET binding factor 2 | | -2.75 | 0.01197 |
| 212364_at | *MYO1B* | | | myosin IB | | -2.75 | 0.01449 |
| 236327_at |  | | |  | | -2.75 | 0.01529 |
| 225919_s_at | *C9orf72* | | | chromosome 9 open reading frame 72 | | -2.75 | 0.01606 |
| 227350_at | *HELLS* | | | helicase, lymphoid-specific | | -2.75 | 0.01672 |
| 227856_at | *C4orf32* | | | chromosome 4 open reading frame 32 | | -2.75 | 0.02063 |
| 238970_at |  | | |  | | -2.75 | 0.04089 |
| 1558686_at |  | | |  | | -2.75 | 0.04291 |
| 211089_s_at | *NEK3* | | | NIMA-related kinase 3 | | -2.76 | 0.00008 |
| 212787_at | *YLPM1* | | | YLP motif containing 1 | | -2.76 | 0.00143 |
| 220301_at | *CCDC102B* | | | coiled-coil domain containing 102B | | -2.76 | 0.00204 |
| 212833_at | *SLC25A46* | | | solute carrier family 25, member 46 | | -2.76 | 0.0024 |
| 1561042_at |  | | |  | | -2.76 | 0.00257 |
| 240948_at |  | | |  | | -2.76 | 0.00407 |
| 218470_at | *YARS2* | | | tyrosyl-tRNA synthetase 2, mitochondrial | | -2.76 | 0.00415 |
| 208751_at | *NAPA* | | | N-ethylmaleimide-sensitive factor attachment protein, alpha | | -2.76 | 0.00558 |
| 200774_at | *FAM120A* | | | family with sequence similarity 120A | | -2.76 | 0.00659 |
| 1558080_s_at | *DNAJC3* | | | DnaJ (Hsp40) homolog, subfamily C, member 3 | | -2.76 | 0.00683 |
| 212037_at | *PNN* | | | pinin, desmosome associated protein | | -2.76 | 0.00723 |
| 220955_x_at | *RAB23* | | | RAB23, member RAS oncogene family | | -2.76 | 0.00727 |
| 1563796_s_at | *EARS2* | | | glutamyl-tRNA synthetase 2, mitochondrial | | -2.76 | 0.00979 |
| 220014_at | *PRR16* | | | proline rich 16 | | -2.76 | 0.01227 |
| 208047_s_at | *NAB1* | | | NGFI-A binding protein 1 (EGR1 binding protein 1) | | -2.76 | 0.01512 |
| 234212_at | *ACTR2* | | | ARP2 actin-related protein 2 homolog (yeast) | | -2.76 | 0.01642 |
| 242727_at | *ARL5B* | | | ADP-ribosylation factor-like 5B | | -2.76 | 0.01932 |
| 203543_s_at | *KLF9* | | | Kruppel-like factor 9 | | -2.76 | 0.0259 |
| 206710_s_at | *EPB41L3* | | | erythrocyte membrane protein band 4.1-like 3 | | -2.76 | 0.03038 |
| 241374_at | *TMEM39A* | | | transmembrane protein 39A | | -2.77 | 0.00012 |
| 224705_s_at | *TNRC6A* | | | trinucleotide repeat containing 6A | | -2.77 | 0.00174 |
| 222754_at | *TRNT1* | | | tRNA nucleotidyl transferase, CCA-adding, 1 | | -2.77 | 0.00319 |
| 201450_s_at | *TIA1* | | | TIA1 cytotoxic granule-associated RNA binding protein | | -2.77 | 0.00376 |
| 225452_at | *MED1* | | | mediator complex subunit 1 | | -2.77 | 0.00404 |
| 1555127_at | *MOCS1* | | | molybdenum cofactor synthesis 1 | | -2.77 | 0.00441 |
| 221931_s_at | *SEH1L* | | | SEH1-like (S. cerevisiae) | | -2.77 | 0.00563 |
| 200602_at | *APP* | | | amyloid beta (A4) precursor protein | | -2.77 | 0.01084 |
| 206205_at | *MPHOSPH9* | | | M-phase phosphoprotein 9 | | -2.77 | 0.01097 |
| 212919_at | *DCP2* | | | decapping mRNA 2 | | -2.77 | 0.01439 |
| 212635_at | *TNPO1* | | | transportin 1 | | -2.77 | 0.01482 |
| 217196_s_at | *CAMSAP2* | | | calmodulin regulated spectrin-associated protein family, member 2 | | -2.77 | 0.01558 |
| 227776_at | *ACER3* | | | alkaline ceramidase 3 | | -2.77 | 0.02 |
| 236044_at | *PPAPDC1A* | | | phosphatidic acid phosphatase type 2 domain containing 1A | | -2.78 | 0.00044 |
| 212751_at | *UBE2N* | | | ubiquitin-conjugating enzyme E2N | | -2.78 | 0.00147 |
| 234580_at | *TMEM106A* | | | transmembrane protein 106A | | -2.78 | 0.00336 |
| 227133_at | *FAM199X* | | | family with sequence similarity 199, X-linked | | -2.78 | 0.0035 |
| 210970_s_at | *IBTK* | | | inhibitor of Bruton agammaglobulinemia tyrosine kinase | | -2.78 | 0.00549 |
| 208800_at | *SRP72* | | | signal recognition particle 72kDa | | -2.78 | 0.00589 |
| 216060_s_at | *DAAM1* | | | dishevelled associated activator of morphogenesis 1 | | -2.78 | 0.00635 |
| 221493_at | *TSPYL1* | | | TSPY-like 1 | | -2.78 | 0.00709 |
| 226120_at | *TTC8* | | | tetratricopeptide repeat domain 8 | | -2.78 | 0.00946 |
| 224974_at | *SUDS3* | | | suppressor of defective silencing 3 homolog (S. cerevisiae) | | -2.78 | 0.00961 |
| 201582_at | *SEC23B* | | | Sec23 homolog B (S. cerevisiae) | | -2.78 | 0.01131 |
| 215751_at |  | | |  | | -2.78 | 0.01135 |
| 235944_at | *HMCN1* | | | hemicentin 1 | | -2.78 | 0.01206 |
| 212519_at | *UBE2E1* | | | ubiquitin-conjugating enzyme E2E 1 | | -2.78 | 0.01249 |
| 242836_at |  | | |  | | -2.78 | 0.021 |
| 1556497_a_at |  | | |  | | -2.78 | 0.02185 |
| 223204_at | *FAM198B* | | | family with sequence similarity 198, member B | | -2.78 | 0.02419 |
| 1554619_at | *MBLAC2* | | | metallo-beta-lactamase domain containing 2 | | -2.78 | 0.02838 |
| 212123_at | *TCTN3* | | | tectonic family member 3 | | -2.79 | 7.9x10^-5^ |
| 223463_at | *RAB23* | | | RAB23, member RAS oncogene family | | -2.79 | 0.0011 |
| 230149_at |  | | |  | | -2.79 | 0.00197 |
| 205282_at | *LRP8* | | | low density lipoprotein receptor-related protein 8, apolipoprotein e receptor | | -2.79 | 0.00316 |
| 231041_at | *POLR1E* | | | polymerase (RNA) I polypeptide E, 53kDa | | -2.79 | 0.00439 |
| 226276_at | *TMEM167A* | | | transmembrane protein 167A | | -2.79 | 0.00733 |
| 241866_at | *SLC16A7* | | | solute carrier family 16, member 7 (monocarboxylic acid transporter 2) | | -2.79 | 0.00929 |
| 235609_at | *BRIP1* | | | BRCA1 interacting protein C-terminal helicase 1 | | -2.79 | 0.01066 |
| 214440_at | *NAT1* | | | N-acetyltransferase 1 (arylamine N-acetyltransferase) | | -2.79 | 0.01159 |
| 205190_at | *PLS1* | | | plastin 1 | | -2.79 | 0.0176 |
| 227399_at | *VGLL3* | | | vestigial like 3 (Drosophila) | | -2.79 | 0.02649 |
| 218128_at | *NFYB* | | | nuclear transcription factor Y, beta | | -2.79 | 0.03135 |
| 1559515_at |  | | |  | | -2.79 | 0.03262 |
| 240170_at |  | | |  | | -2.79 | 0.04015 |
| 213959_s_at | *RPGRIP1L* | | | RPGRIP1-like | | -2.8 | 0.00034 |
| 201695_s_at | *PNP* | | | purine nucleoside phosphorylase | | -2.8 | 0.00036 |
| 204283_at | *FARS2* | | | phenylalanyl-tRNA synthetase 2, mitochondrial | | -2.8 | 0.00068 |
| 213310_at | *CTA-204B4.6* | | | NULL | | -2.8 | 0.00125 |
| 224720_at | *MIB1* | | | mindbomb E3 ubiquitin protein ligase 1 | | -2.8 | 0.00331 |
| 235333_at | *B4GALT6* | | | UDP-Gal:betaGlcNAc beta 1,4- galactosyltransferase, polypeptide 6 | | -2.8 | 0.0036 |
| 222549_at | *CLDN1* | | | claudin 1 | | -2.8 | 0.00374 |
| 1567576_at |  | | |  | | -2.8 | 0.0047 |
| 228062_at | *NAP1L5* | | | nucleosome assembly protein 1-like 5 | | -2.8 | 0.005 |
| 212648_at | *DHX29* | | | DEAH (Asp-Glu-Ala-His) box polypeptide 29 | | -2.8 | 0.00699 |
| 201929_s_at | *PKP4* | | | plakophilin 4 | | -2.8 | 0.00855 |
| 204507_s_at | *PPP3R1* | | | protein phosphatase 3, regulatory subunit B, alpha | | -2.8 | 0.01125 |
| 218981_at | *ACN9* | | | ACN9 homolog (S. cerevisiae) | | -2.8 | 0.01273 |
| 238735_at |  | | |  | | -2.8 | 0.01503 |
| 236535_at | *SMC6* | | | structural maintenance of chromosomes 6 | | -2.8 | 0.01778 |
| 214954_at | *SUSD5* | | | sushi domain containing 5 | | -2.8 | 0.01803 |
| 214277_at | *COX11* | | | cytochrome c oxidase assembly homolog 11 (yeast) | | -2.8 | 0.02146 |
| 228248_at | *RICTOR* | | | RPTOR independent companion of MTOR, complex 2 | | -2.8 | 0.0258 |
| 201304_at | *NDUFA5* | | | NADH dehydrogenase (ubiquinone) 1 alpha subcomplex, 5, 13kDa | | -2.8 | 0.03034 |
| 229541_at |  | | |  | | -2.8 | 0.03629 |
| 226630_at | *MIS18BP1* | | | MIS18 binding protein 1 | | -2.8 | 0.03718 |
| 208650_s_at | *CD24* | | | CD24 molecule | | -2.81 | 0.00049 |
| 200799_at | *HSPA1A* | | | heat shock 70kDa protein 1A; heat shock 70kDa protein 1B | | -2.81 | 0.00084 |
| 212996_s_at | *URB1* | | | URB1 ribosome biogenesis 1 homolog (S. cerevisiae) | | -2.81 | 0.00091 |
| 224963_at | *SLC26A2* | | | solute carrier family 26 (sulfate transporter), member 2 | | -2.81 | 0.00095 |
| 242317_at | *HIGD1A* | | | HIG1 hypoxia inducible domain family, member 1A | | -2.81 | 0.0027 |
| 230493_at | *SHISA2* | | | shisa homolog 2 (Xenopus laevis) | | -2.81 | 0.00357 |
| 226337_at | *GORAB* | | | golgin, RAB6-interacting | | -2.81 | 0.00456 |
| 222273_at | *PAPOLG* | | | poly(A) polymerase gamma | | -2.81 | 0.00478 |
| 232825_s_at | *DSEL* | | | dermatan sulfate epimerase-like | | -2.81 | 0.00529 |
| 220037_s_at | *LYVE1* | | | lymphatic vessel endothelial hyaluronan receptor 1 | | -2.81 | 0.00597 |
| 222958_s_at | *DEPDC1* | | | DEP domain containing 1 | | -2.81 | 0.00674 |
| 231495_at |  | | |  | | -2.81 | 0.00709 |
| 222656_at | *UBE2W* | | | ubiquitin-conjugating enzyme E2W (putative) | | -2.81 | 0.00798 |
| 220386_s_at | *EML4* | | | echinoderm microtubule associated protein like 4 | | -2.81 | 0.01758 |
| 232752_at | *LOXL1-AS1* | | | LOXL1 antisense RNA 1 | | -2.81 | 0.01808 |
| 1555780_a_at | *RHEB* | | | Ras homolog enriched in brain | | -2.81 | 0.03183 |
| 220103_s_at | *MRPS18C* | | | mitochondrial ribosomal protein S18C | | -2.81 | 0.03923 |
| 210519_s_at | *NQO1* | | | NAD(P)H dehydrogenase, quinone 1 | | -2.82 | 1.2x10^-5^ |
| 230138_at | *NCS1* | | | neuronal calcium sensor 1 | | -2.82 | 3.2x10^-5^ |
| 219569_s_at | *SLC35G2* | | | solute carrier family 35, member G2 | | -2.82 | 0.00051 |
| 209300_s_at | *NECAP1* | | | NECAP endocytosis associated 1 | | -2.82 | 0.00073 |
| 205867_at | *PTPN11* | | | protein tyrosine phosphatase, non-receptor type 11 | | -2.82 | 0.00105 |
| 213899_at | *METAP2* | | | methionyl aminopeptidase 2 | | -2.82 | 0.00159 |
| 217752_s_at | *CNDP2* | | | CNDP dipeptidase 2 (metallopeptidase M20 family) | | -2.82 | 0.00218 |
| 239761_at | *GCNT1* | | | glucosaminyl (N-acetyl) transferase 1, core 2 | | -2.82 | 0.00275 |
| 209630_s_at | *FBXW2* | | | F-box and WD repeat domain containing 2 | | -2.82 | 0.00304 |
| 1559062_at |  | | |  | | -2.82 | 0.0058 |
| 202346_at | *UBE2K* | | | ubiquitin-conjugating enzyme E2K | | -2.82 | 0.00666 |
| 214830_at | *SLC38A6* | | | solute carrier family 38, member 6 | | -2.82 | 0.00717 |
| 243835_at | *ZDHHC21* | | | zinc finger, DHHC-type containing 21 | | -2.82 | 0.00886 |
| 244414_at |  | | |  | | -2.82 | 0.01082 |
| 202502_at | *ACADM* | | | acyl-CoA dehydrogenase, C-4 to C-12 straight chain | | -2.82 | 0.01154 |
| 227798_at | *SMAD1* | | | SMAD family member 1 | | -2.82 | 0.01257 |
| 239218_at | *PDE1C* | | | phosphodiesterase 1C, calmodulin-dependent 70kDa | | -2.82 | 0.01488 |
| 201830_s_at | *NET1* | | | neuroepithelial cell transforming 1 | | -2.82 | 0.02861 |
| 212989_at | *SGMS1* | | | sphingomyelin synthase 1 | | -2.82 | 0.03723 |
| 220703_at | *IDI2-AS1* | | | IDI2 antisense RNA 1 | | -2.83 | 0.00033 |
| 229428_at | *TIMM23* | | | translocase of inner mitochondrial membrane 23 homolog (yeast) | | -2.83 | 0.00037 |
| 242474_s_at | *VMA21* | | | VMA21 vacuolar H+-ATPase homolog (S. cerevisiae) | | -2.83 | 0.00083 |
| 234074_at |  | | |  | | -2.83 | 0.00237 |
| 213372_at | *PAQR3* | | | progestin and adipoQ receptor family member III | | -2.83 | 0.00287 |
| 225340_s_at | *CAPRIN1* | | | cell cycle associated protein 1 | | -2.83 | 0.00398 |
| 224654_at | *DDX21* | | | DEAD (Asp-Glu-Ala-Asp) box helicase 21 | | -2.83 | 0.00414 |
| 221749_at | *YTHDF3* | | | YTH domain family, member 3 | | -2.83 | 0.00643 |
| 218396_at | *VPS13C* | | | vacuolar protein sorting 13 homolog C (S. cerevisiae) | | -2.83 | 0.007 |
| 235321_at | *NDUFS1* | | | NADH dehydrogenase (ubiquinone) Fe-S protein 1, 75kDa (NADH-coenzyme Q reductase) | | -2.83 | 0.00719 |
| 223336_s_at | *RAB18* | | | RAB18, member RAS oncogene family | | -2.83 | 0.00787 |
| 201589_at | *SMC1A* | | | structural maintenance of chromosomes 1A | | -2.83 | 0.0087 |
| 205394_at | *CHEK1* | | | checkpoint kinase 1 | | -2.83 | 0.01491 |
| 1555948_s_at | *FAM120A* | | | family with sequence similarity 120A | | -2.83 | 0.01582 |
| 228620_at |  | | |  | | -2.83 | 0.02452 |
| 203310_at | *STXBP3* | | | syntaxin binding protein 3 | | -2.83 | 0.03698 |
| 225060_at | *LRP11* | | | low density lipoprotein receptor-related protein 11 | | -2.84 | 0.00097 |
| 224779_s_at | *FAM96A* | | | family with sequence similarity 96, member A | | -2.84 | 0.00112 |
| 227274_at | *SYNJ2BP* | | | synaptojanin 2 binding protein | | -2.84 | 0.00168 |
| 225346_at | *MTERFD3* | | | MTERF domain containing 3 | | -2.84 | 0.00176 |
| 202541_at | *AIMP1* | | | aminoacyl tRNA synthetase complex-interacting multifunctional protein 1 | | -2.84 | 0.00284 |
| 212553_at | *RPRD2* | | | regulation of nuclear pre-mRNA domain containing 2 | | -2.84 | 0.00339 |
| 222239_s_at | *INTS6* | | | integrator complex subunit 6 | | -2.84 | 0.00483 |
| 201151_s_at | *MBNL1* | | | muscleblind-like splicing regulator 1 | | -2.84 | 0.0059 |
| 221229_s_at | *TRMT61B* | | | tRNA methyltransferase 61 homolog B (S. cerevisiae) | | -2.84 | 0.00965 |
| 213064_at | *ZC3H14* | | | zinc finger CCCH-type containing 14 | | -2.84 | 0.0123 |
| 209974_s_at | *BUB3* | | | BUB3 mitotic checkpoint protein | | -2.84 | 0.01261 |
| 222309_at |  | | |  | | -2.84 | 0.01294 |
| 213110_s_at | *COL4A5* | | | collagen, type IV, alpha 5 | | -2.84 | 0.01339 |
| 238752_at | *GPLD1* | | | glycosylphosphatidylinositol specific phospholipase D1 | | -2.84 | 0.02068 |
| 211379_x_at | *B3GALNT1* | | | beta-1,3-N-acetylgalactosaminyltransferase 1 (globoside blood group) | | -2.84 | 0.0479 |
| 221995_s_at |  | | |  | | -2.85 | 0.00039 |
| 229236_s_at | *SFXN4* | | | sideroflexin 4 | | -2.85 | 0.00154 |
| 235759_at |  | | |  | | -2.85 | 0.00194 |
| 221158_at | *PAXBP1* | | | PAX3 and PAX7 binding protein 1 | | -2.85 | 0.00222 |
| 209653_at | *KPNA4* | | | karyopherin alpha 4 (importin alpha 3) | | -2.85 | 0.00235 |
| 225880_at | *TOR1AIP2* | | | torsin A interacting protein 2 | | -2.85 | 0.00286 |
| 228605_at | *UBXN2A* | | | UBX domain protein 2A | | -2.85 | 0.00396 |
| 216322_at | *CD58* | | | CD58 molecule | | -2.85 | 0.00435 |
| 226628_at | *THOC2* | | | THO complex 2 | | -2.85 | 0.00466 |
| 224587_at | *SUB1* | | | SUB1 homolog (S. cerevisiae) | | -2.85 | 0.00477 |
| 222808_at | *ALG13* | | | ALG13, UDP-N-acetylglucosaminyltransferase subunit | | -2.85 | 0.00761 |
| 223261_at | *POLK* | | | polymerase (DNA directed) kappa | | -2.85 | 0.00794 |
| 228363_at | *XIAP* | | | X-linked inhibitor of apoptosis | | -2.85 | 0.00813 |
| 204049_s_at | *PHACTR2* | | | phosphatase and actin regulator 2 | | -2.85 | 0.00876 |
| 222990_at | *UBQLN1* | | | ubiquilin 1 | | -2.85 | 0.00905 |
| 244703_x_at | *IPO9* | | | importin 9 | | -2.85 | 0.00971 |
| 230263_s_at | *DOCK5* | | | dedicator of cytokinesis 5 | | -2.85 | 0.01103 |
| 204457_s_at | *GAS1* | | | growth arrest-specific 1 | | -2.85 | 0.01143 |
| 1553551_s_at | *ND2* | | | MTND2 | | -2.85 | 0.01336 |
| 229174_at | *C3orf38* | | | chromosome 3 open reading frame 38 | | -2.85 | 0.01539 |
| 206295_at | *IL18* | | | interleukin 18 (interferon-gamma-inducing factor) | | -2.85 | 0.01774 |
| 224876_at | *C5orf24* | | | chromosome 5 open reading frame 24 | | -2.85 | 0.01811 |
| 217853_at | *TNS3* | | | tensin 3 | | -2.86 | 0.00165 |
| 225521_at | *ANAPC7* | | | anaphase promoting complex subunit 7 | | -2.86 | 0.00204 |
| 228293_at | *DEPDC7* | | | DEP domain containing 7 | | -2.86 | 0.00254 |
| 204005_s_at | *PAWR* | | | PRKC, apoptosis, WT1, regulator | | -2.86 | 0.0026 |
| 209141_at | *UBE2G1* | | | ubiquitin-conjugating enzyme E2G 1 | | -2.86 | 0.00267 |
| 218458_at | *GMCL1* | | | germ cell-less, spermatogenesis associated 1 | | -2.86 | 0.00463 |
| 215245_x_at | *FMR1* | | | fragile X mental retardation 1 | | -2.86 | 0.00881 |
| 201358_s_at | *COPB1* | | | coatomer protein complex, subunit beta 1 | | -2.86 | 0.00979 |
| 225599_s_at | *TRIQK* | | | triple QxxK/R motif containing | | -2.86 | 0.01163 |
| 209130_at | *SNAP23* | | | synaptosomal-associated protein, 23kDa | | -2.86 | 0.01497 |
| 203253_s_at | *PPIP5K2* | | | diphosphoinositol pentakisphosphate kinase 2 | | -2.86 | 0.01776 |
| 221751_at | *PANK3* | | | pantothenate kinase 3 | | -2.86 | 0.02162 |
| 227772_at | *LATS1* | | | large tumor suppressor kinase 1 | | -2.86 | 0.02944 |
| 242074_at |  | | |  | | -2.86 | 0.03976 |
| 209124_at | *MYD88* | | | myeloid differentiation primary response 88 | | -2.87 | 6.6x10^-5^ |
| 201477_s_at | *RRM1* | | | ribonucleotide reductase M1 | | -2.87 | 0.00035 |
| 212248_at | *MTDH* | | | metadherin | | -2.87 | 0.00105 |
| 225475_at | *MIER1* | | | mesoderm induction early response 1 homolog (Xenopus laevis) | | -2.87 | 0.00471 |
| 242826_at |  | | |  | | -2.87 | 0.00502 |
| 204094_s_at | *TSC22D2* | | | TSC22 domain family, member 2 | | -2.87 | 0.00543 |
| 218712_at | *C1orf109* | | | chromosome 1 open reading frame 109 | | -2.87 | 0.00853 |
| 204847_at | *ZBTB11* | | | zinc finger and BTB domain containing 11 | | -2.87 | 0.0093 |
| 204410_at | *EIF1AY* | | | eukaryotic translation initiation factor 1A, Y-linked | | -2.87 | 0.01081 |
| 214835_s_at | *SUCLG2* | | | succinate-CoA ligase, GDP-forming, beta subunit | | -2.87 | 0.01441 |
| 210904_s_at | *IL13RA1* | | | interleukin 13 receptor, alpha 1 | | -2.87 | 0.01556 |
| 1565886_at |  | | |  | | -2.87 | 0.01569 |
| 218236_s_at | *PRKD3* | | | protein kinase D3 | | -2.87 | 0.02913 |
| 212281_s_at | *TMEM97* | | | transmembrane protein 97 | | -2.88 | 0.00029 |
| 202741_at | *PRKACB* | | | protein kinase, cAMP-dependent, catalytic, beta | | -2.88 | 0.0008 |
| 213282_at | *APOOL* | | | apolipoprotein O-like | | -2.88 | 0.00137 |
| 1564911_at | *SNHG4* | | | small nucleolar RNA host gene 4 (non-protein coding) | | -2.88 | 0.00162 |
| 222700_at | *ATL2* | | | atlastin GTPase 2 | | -2.88 | 0.0029 |
| 218326_s_at | *LGR4* | | | leucine-rich repeat containing G protein-coupled receptor 4 | | -2.88 | 0.0065 |
| 228202_at |  | | |  | | -2.88 | 0.00753 |
| 218598_at | *RINT1* | | | RAD50 interactor 1 | | -2.88 | 0.00776 |
| 202932_at | *YES1* | | | v-yes-1 Yamaguchi sarcoma viral oncogene homolog 1 | | -2.88 | 0.00808 |
| 235318_at | *FBN1* | | | fibrillin 1 | | -2.88 | 0.00952 |
| 200821_at | *LAMP2* | | | lysosomal-associated membrane protein 2 | | -2.88 | 0.00978 |
| 211575_s_at | *UBE3A* | | | ubiquitin protein ligase E3A | | -2.88 | 0.01584 |
| 204407_at | *TTF2* | | | transcription termination factor, RNA polymerase II | | -2.88 | 0.0265 |
| 215150_at | *YOD1* | | | YOD1 deubiquitinase | | -2.89 | 0.00082 |
| 237374_at |  | | |  | | -2.89 | 0.0009 |
| 211150_s_at | *DLAT* | | | dihydrolipoamide S-acetyltransferase | | -2.89 | 0.00095 |
| 222391_at | *TMEM30A* | | | transmembrane protein 30A | | -2.89 | 0.00254 |
| 225754_at | *AP1G1* | | | adaptor-related protein complex 1, gamma 1 subunit | | -2.89 | 0.00411 |
| 200798_x_at | *MCL1* | | | myeloid cell leukemia sequence 1 (BCL2-related) | | -2.89 | 0.005 |
| 241730_at | *MYNN* | | | myoneurin | | -2.89 | 0.00573 |
| 209296_at | *PPM1B* | | | protein phosphatase, Mg2+/Mn2+ dependent, 1B | | -2.89 | 0.00623 |
| 201084_s_at | *BCLAF1* | | | BCL2-associated transcription factor 1 | | -2.89 | 0.00676 |
| 1553646_at | *HDX* | | | highly divergent homeobox | | -2.89 | 0.00711 |
| 232278_s_at | *DEPDC1* | | | DEP domain containing 1 | | -2.89 | 0.00817 |
| 212569_at | *SMCHD1* | | | structural maintenance of chromosomes flexible hinge domain containing 1 | | -2.89 | 0.02546 |
| 209206_at |  | | |  | | -2.89 | 0.02744 |
| 218446_s_at | *TVP23B* | | | trans-golgi network vesicle protein 23 homolog B (S. cerevisiae) | | -2.89 | 0.0423 |
| 233050_at | *KIAA0408* | | | KIAA0408; SOGA family member 3 | | -2.89 | 0.04614 |
| 225485_at | *CEP41* | | | centrosomal protein 41kDa | | -2.9 | 2.4x10^-5^ |
| 221667_s_at | *HSPB8* | | | heat shock 22kDa protein 8 | | -2.9 | 0.00099 |
| 227370_at | *FAM171B* | | | family with sequence similarity 171, member B | | -2.9 | 0.00119 |
| 219023_at | *AP1AR* | | | adaptor-related protein complex 1 associated regulatory protein | | -2.9 | 0.00177 |
| 212812_at | *SERINC5* | | | serine incorporator 5 | | -2.9 | 0.0022 |
| 219017_at | *ETNK1* | | | ethanolamine kinase 1 | | -2.9 | 0.00388 |
| 210705_s_at | *TRIM5* | | | tripartite motif containing 5 | | -2.9 | 0.00397 |
| 201908_at | *DVL3* | | | dishevelled segment polarity protein 3 | | -2.9 | 0.00405 |
| 212720_at | *PAPOLA* | | | poly(A) polymerase alpha | | -2.9 | 0.00909 |
| 1554080_at | *RQCD1* | | | RCD1 required for cell differentiation1 homolog (S. pombe) | | -2.9 | 0.00971 |
| 225644_at | *CCDC117* | | | coiled-coil domain containing 117 | | -2.9 | 0.01136 |
| 220099_s_at |  | | |  | | -2.9 | 0.01757 |
| 237978_at |  | | |  | | -2.9 | 0.02597 |
| 221727_at | *SUB1* | | | SUB1 homolog (S. cerevisiae) | | -2.9 | 0.03384 |
| 201467_s_at | *NQO1* | | | NAD(P)H dehydrogenase, quinone 1 | | -2.91 | 0.00042 |
| 207165_at | *HMMR* | | | hyaluronan-mediated motility receptor (RHAMM) | | -2.91 | 0.00172 |
| 202987_at | *TRAF3IP2* | | | TRAF3 interacting protein 2 | | -2.91 | 0.00236 |
| 235795_at | *PAX6* | | | paired box 6 | | -2.91 | 0.00242 |
| 244103_at | *SDE2* | | | SDE2 telomere maintenance homolog (S. pombe) | | -2.91 | 0.00335 |
| 221770_at |  | | |  | | -2.91 | 0.00543 |
| 208731_at | *RAB2A* | | | RAB2A, member RAS oncogene family | | -2.91 | 0.00563 |
| 221505_at | *ANP32E* | | | acidic (leucine-rich) nuclear phosphoprotein 32 family, member E | | -2.91 | 0.00694 |
| 222553_x_at | *OXR1* | | | oxidation resistance 1 | | -2.91 | 0.00793 |
| 213049_at | *RALGAPA1* | | | Ral GTPase activating protein, alpha subunit 1 (catalytic) | | -2.91 | 0.00977 |
| 201712_s_at | *RANBP2* | | | RAN binding protein 2 | | -2.91 | 0.01109 |
| 228397_at | *TUG1* | | | taurine upregulated 1 (non-protein coding) | | -2.91 | 0.01162 |
| 213074_at | *PHIP* | | | pleckstrin homology domain interacting protein | | -2.91 | 0.01304 |
| 204793_at | *GPRASP1* | | | G protein-coupled receptor associated sorting protein 1 | | -2.91 | 0.0187 |
| 228401_at | *ATAD2* | | | ATPase family, AAA domain containing 2 | | -2.91 | 0.02013 |
| 212981_s_at | *FAM115A* | | | family with sequence similarity 115, member A; protein FAM115A-like | | -2.91 | 0.02316 |
| 221510_s_at | *GLS* | | | glutaminase | | -2.91 | 0.02432 |
| 235236_at |  | | |  | | -2.91 | 0.03806 |
| 225516_at | *SLC7A2* | | | solute carrier family 7 (cationic amino acid transporter, y+ system), member 2 | | -2.92 | 0.00016 |
| 233175_at | *ZNF460* | | | zinc finger protein 460 | | -2.92 | 0.00159 |
| 205909_at | *POLE2* | | | polymerase (DNA directed), epsilon 2, accessory subunit | | -2.92 | 0.00272 |
| 1554047_at | *TXNDC9* | | | thioredoxin domain containing 9 | | -2.92 | 0.00736 |
| 214155_s_at | *LARP4* | | | La ribonucleoprotein domain family, member 4 | | -2.92 | 0.00739 |
| 203403_s_at | *RNF6* | | | ring finger protein (C3H2C3 type) 6 | | -2.92 | 0.00842 |
| 203225_s_at | *RFK* | | | riboflavin kinase | | -2.92 | 0.01367 |
| 227418_at | *MSANTD4* | | | Myb/SANT-like DNA-binding domain containing 4 with coiled-coils | | -2.92 | 0.01607 |
| 225256_at |  | | |  | | -2.92 | 0.01808 |
| 209649_at | *STAM2* | | | signal transducing adaptor molecule (SH3 domain and ITAM motif) 2 | | -2.92 | 0.01832 |
| 216100_s_at | *TOR1AIP1* | | | torsin A interacting protein 1 | | -2.92 | 0.02257 |
| 200608_s_at | *RAD21* | | | RAD21 homolog (S. pombe) | | -2.92 | 0.03388 |
| 226794_at | *STXBP5* | | | syntaxin binding protein 5 (tomosyn) | | -2.92 | 0.03906 |
| 225634_at | *ZC3HAV1* | | | zinc finger CCCH-type, antiviral 1 | | -2.93 | 0.00049 |
| 219059_s_at | *LYVE1* | | | lymphatic vessel endothelial hyaluronan receptor 1 | | -2.93 | 0.00051 |
| 212408_at | *TOR1AIP1* | | | torsin A interacting protein 1 | | -2.93 | 0.00156 |
| 209397_at | *ME2* | | | malic enzyme 2, NAD(+)-dependent, mitochondrial | | -2.93 | 0.00169 |
| 1567361_at | *BDNF-AS* | | | BDNF antisense RNA | | -2.93 | 0.00257 |
| 208264_s_at | *EIF3J* | | | eukaryotic translation initiation factor 3, subunit J | | -2.93 | 0.00373 |
| 219258_at | *TIPIN* | | | TIMELESS interacting protein | | -2.93 | 0.0043 |
| 206795_at | *F2RL2* | | | coagulation factor II (thrombin) receptor-like 2 | | -2.93 | 0.00453 |
| 226135_at | *UHRF1BP1* | | | UHRF1 binding protein 1 | | -2.93 | 0.00471 |
| 222883_at | *SELRC1* | | | Sel1 repeat containing 1 | | -2.93 | 0.00738 |
| 242247_at | *METTL15* | | | methyltransferase like 15 | | -2.93 | 0.00756 |
| 230399_at |  | | |  | | -2.93 | 0.00963 |
| 212426_s_at | *YWHAQ* | | | tyrosine 3-monooxygenase/tryptophan 5-monooxygenase activation protein, theta polypeptide | | -2.93 | 0.01953 |
| 226921_at | *UBR1* | | | ubiquitin protein ligase E3 component n-recognin 1 | | -2.94 | 0.00212 |
| 209385_s_at | *PROSC* | | | proline synthetase co-transcribed homolog (bacterial) | | -2.94 | 0.00505 |
| 206314_at | *ZKSCAN7* | | | zinc finger with KRAB and SCAN domains 7 | | -2.94 | 0.00575 |
| 227105_at | *CSPP1* | | | centrosome and spindle pole associated protein 1 | | -2.94 | 0.00787 |
| 212293_at | *HIPK1* | | | homeodomain interacting protein kinase 1 | | -2.94 | 0.00981 |
| 228841_at | *LYRM7* | | | LYR motif containing 7 | | -2.94 | 0.01584 |
| 212094_at | *PEG10* | | | paternally expressed 10 | | -2.94 | 0.02843 |
| 243502_at | *GJC1* | | | gap junction protein, gamma 1, 45kDa | | -2.94 | 0.0464 |
| 1556190_s_at |  | | |  | | -2.95 | 0.00031 |
| 209537_at | *EXTL2* | | | exostosin-like glycosyltransferase 2 | | -2.95 | 0.00214 |
| 217574_at | *CDH8* | | | cadherin 8, type 2 | | -2.95 | 0.00374 |
| 231016_s_at | *ARNT* | | | aryl hydrocarbon receptor nuclear translocator | | -2.95 | 0.00793 |
| 226103_at | *NEXN* | | | nexilin (F actin binding protein) | | -2.95 | 0.00873 |
| 212410_at | *MICU2* | | | mitochondrial calcium uptake 2 | | -2.95 | 0.01135 |
| 218432_at | *FBXO3* | | | F-box protein 3 | | -2.95 | 0.01196 |
| 227627_at |  | | |  | | -2.95 | 0.01452 |
| 221568_s_at | *LIN7C* | | | lin-7 homolog C (C. elegans) | | -2.95 | 0.01467 |
| 209214_s_at | *EWSR1* | | | EWS RNA-binding protein 1 | | -2.95 | 0.01648 |
| 212450_at | *SECISBP2L* | | | SECIS binding protein 2-like | | -2.95 | 0.01676 |
| 204146_at | *RAD51AP1* | | | RAD51 associated protein 1 | | -2.95 | 0.0224 |
| 238743_at |  | | |  | | -2.96 | 0.00119 |
| 201351_s_at | *YME1L1* | | | YME1-like 1 ATPase | | -2.96 | 0.00219 |
| 217761_at | *ADI1* | | | acireductone dioxygenase 1 | | -2.96 | 0.00581 |
| 206102_at | *GINS1* | | | GINS complex subunit 1 (Psf1 homolog) | | -2.96 | 0.00827 |
| 228974_at | *ZNF677* | | | zinc finger protein 677 | | -2.96 | 0.00934 |
| 222587_s_at | *GALNT7* | | | UDP-N-acetyl-alpha-D-galactosamine:polypeptide N-acetylgalactosaminyltransferase 7 (GalNAc-T7) | | -2.96 | 0.01279 |
| 218454_at | *PLBD1* | | | phospholipase B domain containing 1 | | -2.96 | 0.01287 |
| 209642_at | *BUB1* | | | BUB1 mitotic checkpoint serine/threonine kinase | | -2.96 | 0.04011 |
| 209387_s_at | *TM4SF1* | | | transmembrane 4 L six family member 1 | | -2.97 | 0.00061 |
| 204235_s_at | *GULP1* | | | GULP, engulfment adaptor PTB domain containing 1 | | -2.97 | 0.00176 |
| 227577_at | *EXOC8* | | | exocyst complex component 8 | | -2.97 | 0.00223 |
| 211547_s_at | *PAFAH1B1* | | | platelet-activating factor acetylhydrolase 1b, regulatory subunit 1 (45kDa) | | -2.97 | 0.00276 |
| 225352_at | *SEC62* | | | SEC62 homolog (S. cerevisiae) | | -2.97 | 0.00391 |
| 211717_at | *ANKRD40* | | | ankyrin repeat domain 40 | | -2.97 | 0.00441 |
| 207724_s_at | *SPAST* | | | spastin | | -2.97 | 0.00668 |
| 226694_at | *AKAP2* | | | A kinase (PRKA) anchor protein 2; PALM2-AKAP2 readthrough | | -2.97 | 0.00791 |
| 234990_at | *CBX5* | | | chromobox homolog 5 | | -2.97 | 0.00846 |
| 223315_at | *NTN4* | | | netrin 4 | | -2.97 | 0.0097 |
| 212616_at | *CHD9* | | | chromodomain helicase DNA binding protein 9 | | -2.97 | 0.01049 |
| 207719_x_at | *CEP170* | | | centrosomal protein 170kDa | | -2.97 | 0.01529 |
| 202166_s_at | *PPP1R2* | | | protein phosphatase 1, regulatory (inhibitor) subunit 2 | | -2.97 | 0.01556 |
| 227095_at | *LEPROT* | | | leptin receptor overlapping transcript | | -2.97 | 0.02558 |
| 218027_at | *MRPL15* | | | mitochondrial ribosomal protein L15 | | -2.98 | 0.00086 |
| 225885_at | *EEA1* | | | early endosome antigen 1 | | -2.98 | 0.00286 |
| 234983_at |  | | |  | | -2.98 | 0.00338 |
| 204807_at | *TMEM5* | | | transmembrane protein 5 | | -2.98 | 0.00391 |
| 218989_x_at | *SLC30A5* | | | solute carrier family 30 (zinc transporter), member 5 | | -2.98 | 0.00437 |
| 202904_s_at | *LSM5* | | | LSM5 homolog, U6 small nuclear RNA associated (S. cerevisiae) | | -2.98 | 0.0051 |
| 223538_at | *SERF1A* | | | small EDRK-rich factor 1A (telomeric); small EDRK-rich factor 1B (centromeric) | | -2.98 | 0.0055 |
| 64883_at | *MOSPD2* | | | motile sperm domain containing 2 | | -2.98 | 0.00749 |
| 201019_s_at | *EIF1AX* | | | eukaryotic translation initiation factor 1A, X-linked; eukaryotic translation initiation factor 1A, X-chromosomal-like | | -2.98 | 0.00841 |
| 238761_at | *ELK4* | | | ELK4, ETS-domain protein (SRF accessory protein 1) | | -2.98 | 0.01158 |
| 242420_at |  | | |  | | -2.98 | 0.01176 |
| 226019_at | *OMA1* | | | OMA1 zinc metallopeptidase | | -2.98 | 0.01469 |
| 203049_s_at | *TTC37* | | | tetratricopeptide repeat domain 37 | | -2.98 | 0.01928 |
| 227621_at | *WTAP* | | | Wilms tumor 1 associated protein | | -2.98 | 0.0194 |
| 214960_at | *API5* | | | apoptosis inhibitor 5 | | -2.98 | 0.02067 |
| 208787_at | *MRPL3* | | | mitochondrial ribosomal protein L3 | | -2.99 | 0.00046 |
| 235652_at |  | | |  | | -2.99 | 0.00048 |
| 222040_at | *HNRNPA1* | | | heterogeneous nuclear ribonucleoprotein A1 | | -2.99 | 0.00078 |
| 227680_at | *ZNF326* | | | zinc finger protein 326 | | -2.99 | 0.00214 |
| 212381_at | *USP24* | | | ubiquitin specific peptidase 24 | | -2.99 | 0.00281 |
| 1569098_s_at | *TP53BP1* | | | tumor protein p53 binding protein 1 | | -2.99 | 0.00413 |
| 218987_at | *ATF7IP* | | | activating transcription factor 7 interacting protein | | -2.99 | 0.00444 |
| 206471_s_at | *PLXNC1* | | | plexin C1 | | -2.99 | 0.00629 |
| 237105_at |  | | |  | | -2.99 | 0.00741 |
| 225872_at | *SLC35F5* | | | solute carrier family 35, member F5 | | -2.99 | 0.00919 |
| 210203_at | *CNOT4* | | | CCR4-NOT transcription complex, subunit 4 | | -2.99 | 0.01075 |
| 212610_at | *PTPN11* | | | protein tyrosine phosphatase, non-receptor type 11 | | -2.99 | 0.0111 |
| 244811_at | *PHIP* | | | pleckstrin homology domain interacting protein | | -2.99 | 0.01339 |
| 220220_at | *LRRC37A4P* | | | leucine rich repeat containing 37, member A4, pseudogene | | -2.99 | 0.01354 |
| 218577_at | *LRRC40* | | | leucine rich repeat containing 40 | | -2.99 | 0.01763 |
| 222410_s_at | *SNX6* | | | sorting nexin 6 | | -2.99 | 0.02087 |
| 222320_at |  | | |  | | -3 | 0.00049 |
| 222204_s_at | *RRN3* | | | RRN3 RNA polymerase I transcription factor homolog (S. cerevisiae) | | -3 | 0.0016 |
| 1554513_s_at | *CEP89* | | | centrosomal protein 89kDa | | -3 | 0.00207 |
| 231866_at | *LNPEP* | | | leucyl/cystinyl aminopeptidase | | -3 | 0.00852 |
| 223331_s_at | *DDX20* | | | DEAD (Asp-Glu-Ala-Asp) box polypeptide 20 | | -3 | 0.00945 |
| 225429_at | *PPP6C* | | | protein phosphatase 6, catalytic subunit | | -3 | 0.01096 |
| 215772_x_at | *SUCLG2* | | | succinate-CoA ligase, GDP-forming, beta subunit | | -3 | 0.01394 |
| 218578_at | *CDC73* | | | cell division cycle 73 | | -3 | 0.01435 |
| 202534_x_at | *DHFR* | | | dihydrofolate reductase | | -3 | 0.02631 |
| 1556476_at |  | | |  | | -3.01 | 0.00047 |
| 218209_s_at | *RPRD1A* | | | regulation of nuclear pre-mRNA domain containing 1A | | -3.01 | 0.00217 |
| 200975_at | *PPT1* | | | palmitoyl-protein thioesterase 1 | | -3.01 | 0.00234 |
| 236856_x_at |  | | |  | | -3.01 | 0.00255 |
| 235203_at |  | | |  | | -3.01 | 0.00292 |
| 225477_s_at | *NR2C2* | | | nuclear receptor subfamily 2, group C, member 2 | | -3.01 | 0.00452 |
| 236487_at | *SCLT1* | | | sodium channel and clathrin linker 1 | | -3.01 | 0.00457 |
| 228543_at | *PET117* | | | PET117 homolog (S. cerevisiae) | | -3.01 | 0.00458 |
| 244347_at |  | | |  | | -3.01 | 0.00489 |
| 203799_at | *CD302* | | | CD302 molecule; LY75-CD302 readthrough | | -3.01 | 0.00512 |
| 202119_s_at | *CPNE3* | | | copine III | | -3.01 | 0.00951 |
| 212377_s_at | *NOTCH2* | | | notch 2 | | -3.01 | 0.00957 |
| 223310_x_at | *PNPLA8* | | | patatin-like phospholipase domain containing 8 | | -3.01 | 0.01 |
| 1561567_at |  | | |  | | -3.01 | 0.01391 |
| 208127_s_at | *SOCS5* | | | suppressor of cytokine signaling 5 | | -3.01 | 0.02557 |
| 201424_s_at | *CUL4A* | | | cullin 4A | | -3.01 | 0.02898 |
| 218092_s_at | *AGFG1* | | | ArfGAP with FG repeats 1 | | -3.01 | 0.03143 |
| 225712_at | *GEMIN5* | | | gem (nuclear organelle) associated protein 5 | | -3.02 | 0.00003 |
| 208114_s_at | *ISG20L2* | | | interferon stimulated exonuclease gene 20kDa-like 2 | | -3.02 | 0.00019 |
| 202948_at | *IL1R1* | | | interleukin 1 receptor, type I | | -3.02 | 0.0011 |
| 228323_at | *CASC5* | | | cancer susceptibility candidate 5 | | -3.02 | 0.00129 |
| 221556_at | *CDC14B* | | | cell division cycle 14B | | -3.02 | 0.00347 |
| 200686_s_at | *SRSF11* | | | serine/arginine-rich splicing factor 11 | | -3.02 | 0.00388 |
| 231852_at | *ERI1* | | | exoribonuclease 1 | | -3.02 | 0.00463 |
| 209787_s_at | *HMGN4* | | | high mobility group nucleosomal binding domain 4 | | -3.02 | 0.00542 |
| 1558943_x_at | *ZNF765* | | | zinc finger protein 765 | | -3.02 | 0.0057 |
| 231779_at | *IRAK2* | | | interleukin-1 receptor-associated kinase 2 | | -3.02 | 0.00692 |
| 205395_s_at | *MRE11A* | | | MRE11 meiotic recombination 11 homolog A (S. cerevisiae) | | -3.02 | 0.01101 |
| 222657_s_at | *UBE2W* | | | ubiquitin-conjugating enzyme E2W (putative) | | -3.02 | 0.01139 |
| 220926_s_at | *EDEM3* | | | ER degradation enhancer, mannosidase alpha-like 3 | | -3.02 | 0.01168 |
| 235648_at | *ZNF567* | | | zinc finger protein 567 | | -3.02 | 0.01708 |
| 226773_at | *PPM1K* | | | protein phosphatase, Mg2+/Mn2+ dependent, 1K | | -3.03 | 0.00174 |
| 208070_s_at | *REV3L* | | | REV3-like, polymerase (DNA directed), zeta, catalytic subunit | | -3.03 | 0.00369 |
| 1555758_a_at | *CDKN3* | | | cyclin-dependent kinase inhibitor 3 | | -3.03 | 0.00467 |
| 200749_at | *RAN* | | | RAN, member RAS oncogene family | | -3.03 | 0.0058 |
| 208374_s_at | *CAPZA1* | | | capping protein (actin filament) muscle Z-line, alpha 1 | | -3.03 | 0.00706 |
| 214660_at | *ITGA1* | | | integrin, alpha 1 | | -3.03 | 0.00982 |
| 238597_at | *ANKRD13C* | | | ankyrin repeat domain 13C | | -3.03 | 0.01021 |
| 243524_at |  | | |  | | -3.03 | 0.01204 |
| 204887_s_at | *PLK4* | | | polo-like kinase 4 | | -3.03 | 0.01993 |
| 222837_s_at | *NAA15* | | | N(alpha)-acetyltransferase 15, NatA auxiliary subunit | | -3.04 | 0.00013 |
| 215380_s_at | *GGCT* | | | gamma-glutamylcyclotransferase | | -3.04 | 0.00129 |
| 225676_s_at | *DCAF13* | | | DDB1 and CUL4 associated factor 13 | | -3.04 | 0.00153 |
| 218605_at | *TFB2M* | | | transcription factor B2, mitochondrial | | -3.04 | 0.00703 |
| 226683_at | *SNX18* | | | sorting nexin 18 | | -3.04 | 0.00808 |
| 237814_at |  | | |  | | -3.04 | 0.0101 |
| 200994_at | *IPO7* | | | importin 7 | | -3.04 | 0.01041 |
| 201486_at | *RCN2* | | | reticulocalbin 2, EF-hand calcium binding domain | | -3.04 | 0.01143 |
| 209707_at | *PIGK* | | | phosphatidylinositol glycan anchor biosynthesis, class K | | -3.04 | 0.01198 |
| 235158_at | *TMEM209* | | | transmembrane protein 209 | | -3.04 | 0.02025 |
| 203320_at | *SH2B3* | | | SH2B adaptor protein 3 | | -3.05 | 0.00005 |
| 205401_at | *AGPS* | | | alkylglycerone phosphate synthase | | -3.05 | 7.5x10^-5^ |
| 238823_at | *FMNL3* | | | formin-like 3 | | -3.05 | 0.001 |
| 235086_at | *THBS1* | | | thrombospondin 1 | | -3.05 | 0.00108 |
| 210145_at | *PLA2G4A* | | | phospholipase A2, group IVA (cytosolic, calcium-dependent) | | -3.05 | 0.00138 |
| 1553108_at | *C5orf24* | | | chromosome 5 open reading frame 24 | | -3.05 | 0.00289 |
| 1558250_s_at |  | | |  | | -3.05 | 0.00307 |
| 224692_at | *PPP1R15B* | | | protein phosphatase 1, regulatory subunit 15B | | -3.05 | 0.0048 |
| 228850_s_at | *SLIT2* | | | slit homolog 2 (Drosophila) | | -3.05 | 0.00952 |
| 203626_s_at | *SKP2* | | | S-phase kinase-associated protein 2, E3 ubiquitin protein ligase | | -3.05 | 0.01201 |
| 235331_x_at | *PCGF5* | | | polycomb group ring finger 5 | | -3.05 | 0.01914 |
| 219312_s_at | *ZBTB10* | | | zinc finger and BTB domain containing 10 | | -3.05 | 0.02227 |
| 210793_s_at | *NUP98* | | | nucleoporin 98kDa | | -3.06 | 0.00097 |
| 223397_s_at | *NIP7* | | | NIP7, nucleolar pre-rRNA processing protein | | -3.06 | 0.00165 |
| 209044_x_at | *SF3B4* | | | splicing factor 3b, subunit 4, 49kDa | | -3.06 | 0.00264 |
| 202923_s_at | *GCLC* | | | glutamate-cysteine ligase, catalytic subunit | | -3.06 | 0.00931 |
| 218352_at | *RCBTB1* | | | regulator of chromosome condensation (RCC1) and BTB (POZ) domain containing protein 1 | | -3.06 | 0.01017 |
| 201448_at | *TIA1* | | | TIA1 cytotoxic granule-associated RNA binding protein | | -3.06 | 0.01096 |
| 202438_x_at | *IDS* | | | iduronate 2-sulfatase | | -3.06 | 0.01569 |
| 1569180_at |  | | |  | | -3.06 | 0.019 |
| 219043_s_at | *PDCL3* | | | phosducin-like 3; phosducin-like 3 pseudogene 4 | | -3.07 | 0.00109 |
| 214801_at | *TOR1AIP2* | | | torsin A interacting protein 2 | | -3.07 | 0.00122 |
| 209585_s_at | *MINPP1* | | | multiple inositol-polyphosphate phosphatase 1 | | -3.07 | 0.00185 |
| 236356_at | *NDUFS1* | | | NADH dehydrogenase (ubiquinone) Fe-S protein 1, 75kDa (NADH-coenzyme Q reductase) | | -3.07 | 0.00587 |
| 242019_at | *CERS6* | | | ceramide synthase 6 | | -3.07 | 0.00604 |
| 202304_at | *FNDC3A* | | | fibronectin type III domain containing 3A | | -3.07 | 0.00667 |
| 206543_at | *SMARCA2* | | | SWI/SNF related, matrix associated, actin dependent regulator of chromatin, subfamily a, member 2 | | -3.07 | 0.01041 |
| 205235_s_at | *KIF20B* | | | kinesin family member 20B | | -3.07 | 0.04794 |
| 224918_x_at | *MGST1* | | | microsomal glutathione S-transferase 1 | | -3.08 | 4.8x10^-5^ |
| 218465_at | *TMEM33* | | | transmembrane protein 33 | | -3.08 | 0.00024 |
| 203721_s_at | *UTP18* | | | UTP18 small subunit (SSU) processome component homolog (yeast) | | -3.08 | 0.00126 |
| 201772_at | *AZIN1* | | | antizyme inhibitor 1 | | -3.08 | 0.00154 |
| 207766_at | *CDKL1* | | | cyclin-dependent kinase-like 1 (CDC2-related kinase) | | -3.08 | 0.00191 |
| 242408_at | *STYX* | | | serine/threonine/tyrosine interacting protein | | -3.08 | 0.00205 |
| 203764_at | *DLGAP5* | | | discs, large (Drosophila) homolog-associated protein 5 | | -3.08 | 0.00206 |
| 229492_at | *VANGL1* | | | VANGL planar cell polarity protein 1 | | -3.08 | 0.00336 |
| 234986_at | *GCLM* | | | glutamate-cysteine ligase, modifier subunit | | -3.08 | 0.00428 |
| 226025_at | *ANKRD28* | | | ankyrin repeat domain 28 | | -3.08 | 0.00429 |
| 236027_at | *SFR1* | | | SWI5-dependent recombination repair 1 | | -3.08 | 0.02054 |
| 201843_s_at | *EFEMP1* | | | EGF containing fibulin-like extracellular matrix protein 1 | | -3.08 | 0.03113 |
| 243606_at | *NXPE3* | | | neurexophilin and PC-esterase domain family, member 3 | | -3.09 | 0.00047 |
| 209567_at | *RRS1* | | | RRS1 ribosome biogenesis regulator homolog (S. cerevisiae) | | -3.09 | 0.0008 |
| 225978_at | *RIMKLB* | | | ribosomal modification protein rimK-like family member B | | -3.09 | 0.00106 |
| 243591_at |  | | |  | | -3.09 | 0.00235 |
| 221596_s_at | *RBM48* | | | RNA binding motif protein 48 | | -3.09 | 0.00274 |
| 214257_s_at | *SEC22B* | | | SEC22 vesicle trafficking protein homolog B (S. cerevisiae) (gene/pseudogene) | | -3.09 | 0.00286 |
| 236277_at |  | | |  | | -3.09 | 0.00416 |
| 1554014_at | *CHD2* | | | chromodomain helicase DNA binding protein 2; uncharacterized LOC100507217 | | -3.09 | 0.00608 |
| 242116_x_at |  | | |  | | -3.09 | 0.00611 |
| 225859_at | *XIAP* | | | X-linked inhibitor of apoptosis | | -3.09 | 0.00801 |
| 217745_s_at | *NAA50* | | | N(alpha)-acetyltransferase 50, NatE catalytic subunit | | -3.09 | 0.00807 |
| 201824_at | *RNF14* | | | ring finger protein 14 | | -3.09 | 0.00911 |
| 211960_s_at | *RAB7A* | | | RAB7A, member RAS oncogene family | | -3.09 | 0.00995 |
| 206100_at | *CPM* | | | carboxypeptidase M | | -3.1 | 0.00028 |
| 1553510_s_at | *RQCD1* | | | RCD1 required for cell differentiation1 homolog (S. pombe) | | -3.1 | 0.00035 |
| 217933_s_at | *LAP3* | | | leucine aminopeptidase 3 | | -3.1 | 0.001 |
| 238425_at | *PSMB2* | | | proteasome (prosome, macropain) subunit, beta type, 2 | | -3.1 | 0.00217 |
| 224352_s_at | *CFL2* | | | cofilin 2 (muscle) | | -3.1 | 0.00268 |
| 205264_at | *CD3EAP* | | | CD3e molecule, epsilon associated protein | | -3.1 | 0.00293 |
| 218930_s_at | *TMEM106B* | | | transmembrane protein 106B | | -3.1 | 0.00325 |
| 211754_s_at | *SLC25A17* | | | solute carrier family 25 (mitochondrial carrier; peroxisomal membrane protein, 34kDa), member 17 | | -3.1 | 0.00421 |
| 212298_at | *NRP1* | | | neuropilin 1 | | -3.1 | 0.00443 |
| 209314_s_at | *HBS1L* | | | HBS1-like (S. cerevisiae) | | -3.1 | 0.00713 |
| 228273_at | *PRR11* | | | proline rich 11 | | -3.1 | 0.00838 |
| 218228_s_at | *TNKS2* | | | tankyrase, TRF1-interacting ankyrin-related ADP-ribose polymerase 2 | | -3.1 | 0.01001 |
| 205083_at | *AOX1* | | | aldehyde oxidase 1 | | -3.1 | 0.01004 |
| 209551_at | *YIPF4* | | | Yip1 domain family, member 4 | | -3.1 | 0.01815 |
| 213070_at | *PIK3C2A* | | | phosphatidylinositol-4-phosphate 3-kinase, catalytic subunit type 2 alpha | | -3.1 | 0.01839 |
| 242434_at |  | | |  | | -3.11 | 0.00054 |
| 226806_s_at | *NFIA* | | | nuclear factor I/A | | -3.11 | 0.00135 |
| 218585_s_at | *DTL* | | | denticleless E3 ubiquitin protein ligase homolog (Drosophila) | | -3.11 | 0.00174 |
| 203811_s_at | *DNAJB4* | | | DnaJ (Hsp40) homolog, subfamily B, member 4 | | -3.11 | 0.00207 |
| 225878_at | *KIF1B* | | | kinesin family member 1B | | -3.11 | 0.00418 |
| 212649_at | *DHX29* | | | DEAH (Asp-Glu-Ala-His) box polypeptide 29 | | -3.11 | 0.00461 |
| 212526_at | *SPG20* | | | spastic paraplegia 20 (Troyer syndrome) | | -3.11 | 0.00529 |
| 1562305_x_at | *ZKSCAN3* | | | zinc finger with KRAB and SCAN domains 3 | | -3.11 | 0.0078 |
| 209512_at | *HSDL2* | | | hydroxysteroid dehydrogenase like 2 | | -3.11 | 0.00883 |
| 202783_at | *NNT* | | | nicotinamide nucleotide transhydrogenase | | -3.11 | 0.01091 |
| 1557411_s_at | *SLC25A43* | | | solute carrier family 25, member 43 | | -3.11 | 0.01817 |
| 238565_at |  | | |  | | -3.12 | 0.00029 |
| 222500_at | *PPIL1* | | | peptidylprolyl isomerase (cyclophilin)-like 1 | | -3.12 | 0.0006 |
| 209771_x_at | *CD24* | | | CD24 molecule | | -3.12 | 0.00074 |
| 216198_at | *ATF7IP* | | | activating transcription factor 7 interacting protein | | -3.12 | 0.00163 |
| 210396_s_at | *BOLA2* | | | bolA homolog 2 (E. coli); bolA-like protein 2-like; serine/threonine-protein kinase SMG1-like; smg-1 homolog, phosphatidylinositol 3-kinase-related kinase (C. elegans) pseudogene | | -3.12 | 0.00855 |
| 233952_s_at | *ZBTB21* | | | zinc finger and BTB domain containing 21 | | -3.12 | 0.00975 |
| 226001_at | *KLHL5* | | | kelch-like family member 5 | | -3.12 | 0.01039 |
| 211137_s_at | *ATP2C1* | | | ATPase, Ca++ transporting, type 2C, member 1 | | -3.12 | 0.01277 |
| 212652_s_at | *SNX4* | | | sorting nexin 4 | | -3.12 | 0.01497 |
| 210465_s_at | *SNAPC3* | | | small nuclear RNA activating complex, polypeptide 3, 50kDa | | -3.12 | 0.02419 |
| 209875_s_at | *SPP1* | | | secreted phosphoprotein 1 | | -3.13 | 0.00044 |
| 218694_at | *ARMCX1* | | | armadillo repeat containing, X-linked 1 | | -3.13 | 0.00109 |
| 209308_s_at | *BNIP2* | | | BCL2/adenovirus E1B 19kDa interacting protein 2 | | -3.13 | 0.00204 |
| 226837_at | *SPRED1* | | | sprouty-related, EVH1 domain containing 1 | | -3.13 | 0.00495 |
| 207520_at | *TROVE2* | | | TROVE domain family, member 2 | | -3.13 | 0.00522 |
| 201111_at | *CSE1L* | | | CSE1 chromosome segregation 1-like (yeast) | | -3.13 | 0.00551 |
| 209001_s_at | *ANAPC13* | | | anaphase promoting complex subunit 13 | | -3.13 | 0.00648 |
| 225732_at | *KLHL42* | | | kelch-like family member 42 | | -3.13 | 0.00855 |
| 218932_at | *ZNHIT6* | | | zinc finger, HIT-type containing 6 | | -3.13 | 0.00856 |
| 1554249_a_at | *ZNF638* | | | zinc finger protein 638; ZNF638 intronic transcript 1 (non-protein coding) | | -3.13 | 0.00856 |
| 225406_at | *TWSG1* | | | twisted gastrulation homolog 1 (Drosophila) | | -3.13 | 0.01349 |
| 224892_at | *BLOC1S6* | | | biogenesis of lysosomal organelles complex-1, subunit 6, pallidin | | -3.13 | 0.02322 |
| 229804_x_at | *CBWD1* | | | COBW domain containing 1; COBW domain containing 2; COBW domain containing 3; COBW domain containing 5; COBW domain containing 6; COBW domain-containing protein 1-like; COBW domain-containing protein 5-like | | -3.13 | 0.03402 |
| 201196_s_at | *AMD1* | | | adenosylmethionine decarboxylase 1 | | -3.14 | 0.0005 |
| 202892_at | *CDC23* | | | cell division cycle 23 | | -3.14 | 0.00227 |
| 227070_at | *GLT8D2* | | | glycosyltransferase 8 domain containing 2 | | -3.14 | 0.00349 |
| 201366_at | *ANXA7* | | | annexin A7 | | -3.14 | 0.0043 |
| 235385_at | *Mar-01* | | | membrane-associated ring finger (C3HC4) 1, E3 ubiquitin protein ligase | | -3.14 | 0.00432 |
| 219918_s_at | *ASPM* | | | asp (abnormal spindle) homolog, microcephaly associated (Drosophila) | | -3.14 | 0.00909 |
| 228745_at | *SGTB* | | | small glutamine-rich tetratricopeptide repeat (TPR)-containing, beta | | -3.14 | 0.01099 |
| 222849_s_at | *SCRN3* | | | secernin 3 | | -3.14 | 0.01386 |
| 222740_at | *ATAD2* | | | ATPase family, AAA domain containing 2 | | -3.14 | 0.01534 |
| 225766_s_at | *TNPO1* | | | transportin 1 | | -3.15 | 0.00059 |
| 213365_at | *ERI2* | | | ERI1 exoribonuclease family member 2 | | -3.15 | 0.00075 |
| 227517_s_at | *GAS5* | | | growth arrest-specific 5 (non-protein coding); small nucleolar RNA, C/D box 44; small nucleolar RNA, C/D box 47; small nucleolar RNA, C/D box 76; small nucleolar RNA, C/D box 77; small nucleolar RNA, C/D box 79; small nucleolar RNA, C/D box 80; small nucleolar RNA, C/D box 81 | | -3.15 | 0.00231 |
| 218239_s_at | *GTPBP4* | | | GTP binding protein 4 | | -3.15 | 0.00271 |
| 203405_at | *PSMG1* | | | proteasome (prosome, macropain) assembly chaperone 1 | | -3.15 | 0.00299 |
| 233365_at |  | | |  | | -3.15 | 0.00456 |
| 226989_at | *RGMB* | | | RGM domain family, member B | | -3.15 | 0.00721 |
| 229067_at |  | | |  | | -3.15 | 0.01198 |
| 221703_at | *BRIP1* | | | BRCA1 interacting protein C-terminal helicase 1 | | -3.15 | 0.01657 |
| 225887_at | *PROSER1* | | | proline and serine rich 1 | | -3.15 | 0.01685 |
| 1558739_at |  | | |  | | -3.15 | 0.01781 |
| 219342_at | *CASD1* | | | CAS1 domain containing 1 | | -3.15 | 0.03115 |
| 220817_at | *TRPC4* | | | transient receptor potential cation channel, subfamily C, member 4 | | -3.16 | 0.00025 |
| 214581_x_at | *TNFRSF21* | | | tumor necrosis factor receptor superfamily, member 21 | | -3.16 | 0.00047 |
| 1555471_a_at | *FMN2* | | | formin 2 | | -3.16 | 0.0012 |
| 235412_at | *ARHGEF7* | | | Rho guanine nucleotide exchange factor (GEF) 7 | | -3.16 | 0.00135 |
| 214764_at | *RRP15* | | | ribosomal RNA processing 15 homolog (S. cerevisiae) | | -3.16 | 0.00576 |
| 202353_s_at | *PSMD12* | | | proteasome (prosome, macropain) 26S subunit, non-ATPase, 12 | | -3.16 | 0.00596 |
| 231940_at | *ZNF529* | | | zinc finger protein 529 | | -3.16 | 0.0075 |
| 225836_s_at | *RHNO1* | | | RAD9-HUS1-RAD1 interacting nuclear orphan 1 | | -3.16 | 0.00821 |
| 209627_s_at | *OSBPL3* | | | oxysterol binding protein-like 3 | | -3.16 | 0.00859 |
| 238974_at | *C2orf69* | | | chromosome 2 open reading frame 69 | | -3.16 | 0.01594 |
| 235767_x_at | *PHAX* | | | phosphorylated adaptor for RNA export | | -3.16 | 0.01655 |
| 1554479_a_at | *CARD8* | | | caspase recruitment domain family, member 8 | | -3.16 | 0.02186 |
| 233401_at |  | | |  | | -3.17 | 0.00005 |
| 235771_at |  | | |  | | -3.17 | 0.00142 |
| 228695_at | *C8orf46* | | | chromosome 8 open reading frame 46 | | -3.17 | 0.00609 |
| 225368_at | *HIPK2* | | | homeodomain interacting protein kinase 2 | | -3.17 | 0.00628 |
| 222682_s_at | *FAM114A2* | | | family with sequence similarity 114, member A2 | | -3.17 | 0.00776 |
| 211733_x_at | *SCP2* | | | sterol carrier protein 2 | | -3.17 | 0.00845 |
| 201664_at | *SMC4* | | | structural maintenance of chromosomes 4 | | -3.17 | 0.0128 |
| 242312_x_at |  | | |  | | -3.17 | 0.02126 |
| 235918_x_at | *CEP97* | | | centrosomal protein 97kDa | | -3.17 | 0.04669 |
| 208506_at | *HIST1H3A* | | | histone cluster 1, H3a; histone cluster 1, H3b; histone cluster 1, H3c; histone cluster 1, H3d; histone cluster 1, H3e; histone cluster 1, H3f; histone cluster 1, H3g; histone cluster 1, H3h; histone cluster 1, H3i; histone cluster 1, H3j | | -3.18 | 0.00019 |
| 225164_s_at | *EIF2AK4* | | | eukaryotic translation initiation factor 2 alpha kinase 4 | | -3.18 | 0.01085 |
| 226550_at | *SLC9A7* | | | solute carrier family 9, subfamily A (NHE7, cation proton antiporter 7), member 7 | | -3.18 | 0.02145 |
| 204825_at | *MELK* | | | maternal embryonic leucine zipper kinase | | -3.19 | 0.0004 |
| 241687_at |  | | |  | | -3.19 | 0.00047 |
| 231577_s_at | *GBP1* | | | guanylate binding protein 1, interferon-inducible | | -3.19 | 0.00069 |
| 213226_at | *CCNA2* | | | cyclin A2 | | -3.19 | 0.00398 |
| 228810_at | *CCNYL1* | | | cyclin Y-like 1 | | -3.19 | 0.00484 |
| 215286_s_at | *PHTF2* | | | putative homeodomain transcription factor 2 | | -3.19 | 0.00541 |
| 244030_at | *STYX* | | | serine/threonine/tyrosine interacting protein | | -3.19 | 0.02397 |
| 212846_at | *RRP1B* | | | ribosomal RNA processing 1 homolog B (S. cerevisiae) | | -3.2 | 0.00095 |
| 217554_at |  | | |  | | -3.2 | 0.00119 |
| 232297_at | *KLHL5* | | | kelch-like family member 5 | | -3.2 | 0.00313 |
| 1555501_s_at | *RSRC1* | | | arginine/serine-rich coiled-coil 1 | | -3.2 | 0.00324 |
| 229665_at | *CSTF3* | | | cleavage stimulation factor, 3' pre-RNA, subunit 3, 77kDa | | -3.2 | 0.00443 |
| 219078_at | *GPATCH2* | | | G patch domain containing 2 | | -3.2 | 0.00495 |
| 218185_s_at | *ARMC1* | | | armadillo repeat containing 1 | | -3.2 | 0.00728 |
| 1554086_at | *TUBGCP3* | | | tubulin, gamma complex associated protein 3 | | -3.2 | 0.00844 |
| 204619_s_at | *VCAN* | | | versican | | -3.2 | 0.01262 |
| 205386_s_at | *MDM2* | | | MDM2 oncogene, E3 ubiquitin protein ligase | | -3.2 | 0.01746 |
| 226952_at | *EAF1* | | | ELL associated factor 1 | | -3.21 | 0.00166 |
| 202060_at | *CTR9* | | | Ctr9, Paf1/RNA polymerase II complex component, homolog (S. cerevisiae) | | -3.21 | 0.00332 |
| 241774_at | *FLJ31306* | | | uncharacterized LOC379025 | | -3.21 | 0.00339 |
| 235294_at | *SIKE1* | | | suppressor of IKBKE 1 | | -3.21 | 0.00586 |
| 212985_at | *APBB2* | | | amyloid beta (A4) precursor protein-binding, family B, member 2 | | -3.21 | 0.00614 |
| 200685_at | *SRSF11* | | | serine/arginine-rich splicing factor 11 | | -3.21 | 0.00727 |
| 1569472_s_at | *TTC3* | | | tetratricopeptide repeat domain 3; tetratricopeptide repeat domain 3 pseudogene 1 | | -3.21 | 0.00793 |
| 203213_at | *CDK1* | | | cyclin-dependent kinase 1 | | -3.21 | 0.0081 |
| 223381_at | *NUF2* | | | NUF2, NDC80 kinetochore complex component, homolog (S. cerevisiae) | | -3.21 | 0.01146 |
| 201300_s_at | *PRNP* | | | prion protein | | -3.21 | 0.0163 |
| 227027_at | *GFPT1* | | | glutamine--fructose-6-phosphate transaminase 1 | | -3.21 | 0.02482 |
| 242107_x_at | *TBX18* | | | T-box 18 | | -3.21 | 0.04464 |
| 209064_x_at | *PAIP1* | | | poly(A) binding protein interacting protein 1 | | -3.22 | 0.00101 |
| 218512_at | *WDR12* | | | WD repeat domain 12 | | -3.22 | 0.00109 |
| 231183_s_at |  | | |  | | -3.22 | 0.00179 |
| 230023_at | *NSUN4* | | | NOP2/Sun domain family, member 4 | | -3.22 | 0.00377 |
| 212761_at | *TCF7L2* | | | transcription factor 7-like 2 (T-cell specific, HMG-box) | | -3.22 | 0.00414 |
| 223256_at | *G2E3* | | | G2/M-phase specific E3 ubiquitin protein ligase | | -3.22 | 0.01046 |
| 202430_s_at | *PLSCR1* | | | phospholipid scramblase 1 | | -3.22 | 0.01195 |
| 1559067_a_at |  | | |  | | -3.22 | 0.04672 |
| 216533_at | *PCCA* | | | propionyl CoA carboxylase, alpha polypeptide | | -3.23 | 0.00064 |
| 201859_at | *SRGN* | | | serglycin | | -3.23 | 0.00151 |
| 230098_at | *PHF20L1* | | | PHD finger protein 20-like 1 | | -3.23 | 0.00356 |
| 242458_at | *RALGPS2* | | | Ral GEF with PH domain and SH3 binding motif 2 | | -3.23 | 0.00422 |
| 234405_s_at | *PHAX* | | | phosphorylated adaptor for RNA export | | -3.23 | 0.0049 |
| 218053_at | *PRPF40A* | | | PRP40 pre-mRNA processing factor 40 homolog A (S. cerevisiae) | | -3.23 | 0.00562 |
| 1555227_a_at | *MANEA* | | | mannosidase, endo-alpha | | -3.23 | 0.01253 |
| 242289_at |  | | |  | | -3.23 | 0.02372 |
| 228050_at | *UTP15* | | | UTP15, U3 small nucleolar ribonucleoprotein, homolog (S. cerevisiae) | | -3.24 | 0.00052 |
| 225583_at | *UXS1* | | | UDP-glucuronate decarboxylase 1 | | -3.24 | 0.00082 |
| 225415_at | *DTX3L* | | | deltex 3-like (Drosophila) | | -3.24 | 0.00111 |
| 203208_s_at | *MTFR1* | | | mitochondrial fission regulator 1 | | -3.24 | 0.00477 |
| 219080_s_at | *CTPS2* | | | CTP synthase 2 | | -3.24 | 0.00618 |
| 224437_s_at | *VTA1* | | | Vps20-associated 1 homolog (S. cerevisiae) | | -3.24 | 0.00838 |
| 212063_at | *CD44* | | | CD44 molecule (Indian blood group) | | -3.24 | 0.01219 |
| 223288_at | *USP38* | | | ubiquitin specific peptidase 38 | | -3.24 | 0.01326 |
| 225232_at | *MTMR12* | | | myotubularin related protein 12 | | -3.24 | 0.01574 |
| 201975_at | *CLIP1* | | | CAP-GLY domain containing linker protein 1 | | -3.24 | 0.02082 |
| 203216_s_at | *MYO6* | | | myosin VI | | -3.25 | 0.01019 |
| 222907_x_at | *TMEM50B* | | | transmembrane protein 50B | | -3.25 | 0.01124 |
| 225647_s_at | *CTSC* | | | cathepsin C | | -3.26 | 0.00036 |
| 235451_at | *SMAD5* | | | SMAD family member 5 | | -3.26 | 0.00498 |
| 212453_at | *KIAA1279* | | | KIAA1279 | | -3.26 | 0.00674 |
| 202309_at | *MTHFD1* | | | methylenetetrahydrofolate dehydrogenase (NADP+ dependent) 1, methenyltetrahydrofolate cyclohydrolase, formyltetrahydrofolate synthetase | | -3.27 | 0.00029 |
| 1557820_at | *AFG3L2* | | | AFG3 ATPase family member 3-like 2 (S. cerevisiae) | | -3.27 | 0.0011 |
| 226037_s_at | *TAF9B* | | | TAF9B RNA polymerase II, TATA box binding protein (TBP)-associated factor, 31kDa | | -3.27 | 0.00112 |
| 34764_at | *LARS2* | | | leucyl-tRNA synthetase 2, mitochondrial | | -3.27 | 0.0014 |
| 202742_s_at | *PRKACB* | | | protein kinase, cAMP-dependent, catalytic, beta | | -3.27 | 0.00315 |
| 218919_at | *ZFAND1* | | | zinc finger, AN1-type domain 1 | | -3.27 | 0.00468 |
| 219237_s_at | *DNAJB14* | | | DnaJ (Hsp40) homolog, subfamily B, member 14 | | -3.27 | 0.00995 |
| 203738_at | *C5orf22* | | | chromosome 5 open reading frame 22 | | -3.27 | 0.01124 |
| 204822_at | *TTK* | | | TTK protein kinase | | -3.27 | 0.01575 |
| 222679_s_at | *DCUN1D1* | | | DCN1, defective in cullin neddylation 1, domain containing 1 | | -3.27 | 0.01852 |
| 224850_at | *ATAD1* | | | ATPase family, AAA domain containing 1 | | -3.27 | 0.02809 |
| 209406_at | *BAG2* | | | BCL2-associated athanogene 2 | | -3.28 | 0.0004 |
| 207528_s_at | *SLC7A11* | | | solute carrier family 7 (anionic amino acid transporter light chain, xc- system), member 11 | | -3.28 | 0.00068 |
| 212830_at | *MEGF9* | | | multiple EGF-like-domains 9 | | -3.28 | 0.00073 |
| 205003_at | *DOCK4* | | | dedicator of cytokinesis 4 | | -3.28 | 0.00131 |
| 202265_at | *BMI1* | | | BMI1 polycomb ring finger oncogene; COMMD3-BMI1 readthrough | | -3.28 | 0.00194 |
| 202560_s_at | *CHTOP* | | | chromatin target of PRMT1 | | -3.28 | 0.00217 |
| 203622_s_at | *PNO1* | | | partner of NOB1 homolog (S. cerevisiae) | | -3.28 | 0.00283 |
| 221020_s_at | *SLC25A32* | | | solute carrier family 25 (mitochondrial folate carrier), member 32 | | -3.28 | 0.00481 |
| 221553_at | *MAGT1* | | | magnesium transporter 1 | | -3.28 | 0.00927 |
| 222680_s_at | *DTL* | | | denticleless E3 ubiquitin protein ligase homolog (Drosophila) | | -3.28 | 0.01292 |
| 235204_at | *ENTPD7* | | | ectonucleoside triphosphate diphosphohydrolase 7 | | -3.29 | 0.00076 |
| 229647_at | *NDUFS1* | | | NADH dehydrogenase (ubiquinone) Fe-S protein 1, 75kDa (NADH-coenzyme Q reductase) | | -3.29 | 0.00236 |
| 225153_at | *GFM1* | | | G elongation factor, mitochondrial 1 | | -3.29 | 0.00398 |
| 202909_at | *EPM2AIP1* | | | EPM2A (laforin) interacting protein 1 | | -3.29 | 0.0066 |
| 209623_at | *MCCC2* | | | methylcrotonoyl-CoA carboxylase 2 (beta) | | -3.29 | 0.00965 |
| 210466_s_at | *SERBP1* | | | SERPINE1 mRNA binding protein 1 | | -3.29 | 0.01009 |
| 221027_s_at | *PLA2G12A* | | | phospholipase A2, group XIIA | | -3.29 | 0.0172 |
| 1564520_s_at | *PRMT5* | | | protein arginine methyltransferase 5 | | -3.29 | 0.0176 |
| 203634_s_at | *CPT1A* | | | carnitine palmitoyltransferase 1A (liver) | | -3.3 | 0.00107 |
| 222011_s_at | *SNORA29* | | | small nucleolar RNA, H/ACA box 29; t-complex 1 | | -3.3 | 0.00112 |
| 231640_at |  | | |  | | -3.3 | 0.0013 |
| 204274_at | *EBAG9* | | | estrogen receptor binding site associated, antigen, 9 | | -3.3 | 0.002 |
| 238831_at | *TMEM33* | | | transmembrane protein 33 | | -3.3 | 0.00326 |
| 239116_at | *ANKRD10* | | | ankyrin repeat domain 10 | | -3.3 | 0.00474 |
| 225174_at | *DNAJC10* | | | DnaJ (Hsp40) homolog, subfamily C, member 10 | | -3.3 | 0.00656 |
| 203432_at | *TMPO* | | | thymopoietin | | -3.3 | 0.00983 |
| 204716_at | *CCDC6* | | | coiled-coil domain containing 6 | | -3.3 | 0.01144 |
| 1569289_at | *BIVM* | | | basic, immunoglobulin-like variable motif containing | | -3.3 | 0.01292 |
| 204634_at | *NEK4* | | | NIMA-related kinase 4 | | -3.3 | 0.03289 |
| 204170_s_at | *CKS2* | | | CDC28 protein kinase regulatory subunit 2 | | -3.31 | 0.00065 |
| 228966_at | *PANK2* | | | pantothenate kinase 2 | | -3.31 | 0.00187 |
| 209773_s_at | *RRM2* | | | ribonucleotide reductase M2 | | -3.31 | 0.00252 |
| 226537_at | *HINT3* | | | histidine triad nucleotide binding protein 3 | | -3.31 | 0.00484 |
| 212764_at | *LOC100996668* | | | uncharacterized LOC100996668; zinc finger E-box binding homeobox 1 | | -3.31 | 0.00684 |
| 244852_at | *DSEL* | | | dermatan sulfate epimerase-like | | -3.31 | 0.01171 |
| 231736_x_at | *MGST1* | | | microsomal glutathione S-transferase 1 | | -3.32 | 0.00077 |
| 1553956_at | *TMEM237* | | | transmembrane protein 237 | | -3.32 | 0.00172 |
| 225313_at | *FAM217B* | | | family with sequence similarity 217, member B | | -3.32 | 0.00403 |
| 212168_at | *RBM12* | | | RNA binding motif protein 12 | | -3.32 | 0.00409 |
| 205194_at | *PSPH* | | | phosphoserine phosphatase | | -3.32 | 0.00598 |
| 222108_at | *AMIGO2* | | | adhesion molecule with Ig-like domain 2 | | -3.32 | 0.00717 |
| 218478_s_at | *ZCCHC8* | | | zinc finger, CCHC domain containing 8 | | -3.32 | 0.00804 |
| 218349_s_at | *ZWILCH* | | | zwilch kinetochore protein | | -3.33 | 0.00129 |
| 203743_s_at | *TDG* | | | thymine-DNA glycosylase | | -3.33 | 0.00592 |
| 201297_s_at | *MOB1A* | | | MOB kinase activator 1A | | -3.33 | 0.00829 |
| 1554678_s_at | *HNRNPDL* | | | heterogeneous nuclear ribonucleoprotein D-like | | -3.33 | 0.0091 |
| 237746_at |  | | |  | | -3.33 | 0.02699 |
| 235354_s_at | *RSRC1* | | | arginine/serine-rich coiled-coil 1 | | -3.34 | 0.00152 |
| 209115_at | *UBA3* | | | ubiquitin-like modifier activating enzyme 3 | | -3.34 | 0.00215 |
| 208745_at | *ATP5L* | | | ATP synthase, H+ transporting, mitochondrial Fo complex, subunit G | | -3.34 | 0.00386 |
| 208694_at | *PRKDC* | | | protein kinase, DNA-activated, catalytic polypeptide | | -3.34 | 0.00641 |
| 201352_at | *YME1L1* | | | YME1-like 1 ATPase | | -3.34 | 0.0069 |
| 235740_at | *MCTP1* | | | multiple C2 domains, transmembrane 1 | | -3.34 | 0.00753 |
| 1554572_a_at | *SUV39H2* | | | suppressor of variegation 3-9 homolog 2 (Drosophila) | | -3.34 | 0.00962 |
| 1558636_s_at | *ADAMTS5* | | | ADAM metallopeptidase with thrombospondin type 1 motif, 5 | | -3.34 | 0.01477 |
| 221079_s_at | *METTL2A* | | | methyltransferase like 2A; methyltransferase like 2B | | -3.35 | 0.00371 |
| 209884_s_at | *SLC4A7* | | | solute carrier family 4, sodium bicarbonate cotransporter, member 7 | | -3.35 | 0.00708 |
| 225953_at | *RPRD1A* | | | regulation of nuclear pre-mRNA domain containing 1A | | -3.35 | 0.00964 |
| 220342_x_at | *EDEM3* | | | ER degradation enhancer, mannosidase alpha-like 3 | | -3.35 | 0.01032 |
| 209176_at | *SEC23IP* | | | SEC23 interacting protein | | -3.35 | 0.01142 |
| 227373_at | *ATXN1L* | | | ataxin 1-like | | -3.36 | 0.00383 |
| 236831_at | *CCDC50* | | | coiled-coil domain containing 50 | | -3.36 | 0.00555 |
| 215884_s_at | *UBQLN2* | | | ubiquilin 2 | | -3.36 | 0.00583 |
| 206095_s_at |  | | |  | | -3.36 | 0.01167 |
| 224875_at | *C5orf24* | | | chromosome 5 open reading frame 24 | | -3.36 | 0.02101 |
| 1555741_at | *MRAP* | | | melanocortin 2 receptor accessory protein | | -3.37 | 0.00032 |
| 203023_at | *NOP16* | | | NOP16 nucleolar protein | | -3.37 | 0.0005 |
| 219390_at | *FKBP14* | | | FK506 binding protein 14, 22 kDa | | -3.37 | 0.00166 |
| 226601_at | *SLC30A7* | | | solute carrier family 30 (zinc transporter), member 7 | | -3.37 | 0.00609 |
| 208079_s_at | *AURKA* | | | aurora kinase A | | -3.37 | 0.00665 |
| 223341_s_at | *SCOC* | | | short coiled-coil protein | | -3.37 | 0.00767 |
| 213154_s_at | *BICD2* | | | bicaudal D homolog 2 (Drosophila) | | -3.37 | 0.01567 |
| 229594_at | *SPTY2D1* | | | SPT2, Suppressor of Ty, domain containing 1 (S. cerevisiae) | | -3.37 | 0.03281 |
| 235088_at | *C4orf46* | | | chromosome 4 open reading frame 46 | | -3.37 | 0.04817 |
| 205976_at | *FASTKD2* | | | FAST kinase domains 2 | | -3.38 | 0.0051 |
| 203243_s_at | *PDLIM5* | | | PDZ and LIM domain 5 | | -3.38 | 0.0075 |
| 201427_s_at | *SEPP1* | | | selenoprotein P, plasma, 1 | | -3.38 | 0.00862 |
| 211747_s_at | *LSM5* | | | LSM5 homolog, U6 small nuclear RNA associated (S. cerevisiae) | | -3.38 | 0.01036 |
| 222673_x_at | *FAM122B* | | | family with sequence similarity 122B | | -3.38 | 0.02249 |
| 236032_at |  | | |  | | -3.39 | 0.0018 |
| 212245_at | *MCFD2* | | | multiple coagulation factor deficiency 2 | | -3.39 | 0.0022 |
| 239710_at | *FIGN* | | | fidgetin | | -3.39 | 0.00296 |
| 203102_s_at | *MGAT2* | | | mannosyl (alpha-1,6-)-glycoprotein beta-1,2-N-acetylglucosaminyltransferase | | -3.39 | 0.00441 |
| 211340_s_at | *MCAM* | | | melanoma cell adhesion molecule | | -3.39 | 0.00486 |
| 222714_s_at | *LACTB2* | | | lactamase, beta 2 | | -3.39 | 0.00502 |
| 219802_at | *PYROXD1* | | | pyridine nucleotide-disulphide oxidoreductase domain 1 | | -3.39 | 0.00847 |
| 225497_at | *ATE1* | | | arginyltransferase 1 | | -3.39 | 0.01062 |
| 208054_at | *HERC4* | | | HECT and RLD domain containing E3 ubiquitin protein ligase 4 | | -3.39 | 0.02448 |
| 218649_x_at | *NEMF* | | | nuclear export mediator factor | | -3.4 | 0.00353 |
| 212308_at | *CLASP2* | | | cytoplasmic linker associated protein 2 | | -3.4 | 0.01028 |
| 1553685_s_at | *SP1* | | | Sp1 transcription factor | | -3.4 | 0.0142 |
| 215708_s_at |  | | |  | | -3.4 | 0.03091 |
| 235267_at |  | | |  | | -3.4 | 0.03304 |
| 204256_at | *ELOVL6* | | | ELOVL fatty acid elongase 6 | | -3.41 | 0.00034 |
| 213374_x_at | *HIBCH* | | | 3-hydroxyisobutyryl-CoA hydrolase | | -3.41 | 0.00051 |
| 236223_s_at | *RIT1* | | | Ras-like without CAAX 1 | | -3.41 | 0.00097 |
| 225765_at | *TNPO1* | | | transportin 1 | | -3.41 | 0.00135 |
| 228063_s_at | *NAP1L5* | | | nucleosome assembly protein 1-like 5 | | -3.41 | 0.00142 |
| 218313_s_at | *GALNT7* | | | UDP-N-acetyl-alpha-D-galactosamine:polypeptide N-acetylgalactosaminyltransferase 7 (GalNAc-T7) | | -3.41 | 0.00383 |
| 213853_at | *DNAJC24* | | | DnaJ (Hsp40) homolog, subfamily C, member 24 | | -3.41 | 0.00425 |
| 222118_at | *CENPN* | | | centromere protein N | | -3.41 | 0.00489 |
| 242905_at | *PNO1* | | | partner of NOB1 homolog (S. cerevisiae) | | -3.41 | 0.00701 |
| 202666_s_at | *ACTL6A* | | | actin-like 6A | | -3.41 | 0.00864 |
| 225268_at | *KPNA4* | | | karyopherin alpha 4 (importin alpha 3) | | -3.41 | 0.01012 |
| 224793_s_at | *TGFBR1* | | | transforming growth factor, beta receptor 1 | | -3.41 | 0.0109 |
| 203211_s_at | *MTMR2* | | | myotubularin related protein 2 | | -3.42 | 0.00441 |
| 225028_at |  | | |  | | -3.42 | 0.00585 |
| 55872_at | *ZNF512B* | | | zinc finger protein 512B | | -3.42 | 0.01419 |
| 220085_at | *HELLS* | | | helicase, lymphoid-specific | | -3.43 | 0.00457 |
| 217540_at | *NXPE3* | | | neurexophilin and PC-esterase domain family, member 3 | | -3.43 | 0.00493 |
| 218353_at | *RGS5* | | | regulator of G-protein signaling 5 | | -3.43 | 0.00636 |
| 242685_at | *GTPBP8* | | | GTP-binding protein 8 (putative) | | -3.43 | 0.00868 |
| 209666_s_at | *CHUK* | | | conserved helix-loop-helix ubiquitous kinase | | -3.44 | 0.00459 |
| 212205_at | *H2AFV* | | | H2A histone family, member V | | -3.44 | 0.00557 |
| 223547_at | *JKAMP* | | | JNK1/MAPK8-associated membrane protein | | -3.44 | 0.00811 |
| 212499_s_at | *FCF1* | | | FCF1 rRNA-processing protein; mitogen-activated protein kinase 1 interacting protein 1-like | | -3.44 | 0.00874 |
| 207626_s_at | *SLC7A2* | | | solute carrier family 7 (cationic amino acid transporter, y+ system), member 2 | | -3.45 | 0.0005 |
| 225881_at | *SLC35B4* | | | solute carrier family 35, member B4 | | -3.45 | 0.00125 |
| 225267_at | *KPNA4* | | | karyopherin alpha 4 (importin alpha 3) | | -3.45 | 0.00194 |
| 202200_s_at | *SRPK1* | | | SRSF protein kinase 1 | | -3.45 | 0.0021 |
| 244661_at | *SOAT1* | | | sterol O-acyltransferase 1 | | -3.45 | 0.00407 |
| 223269_at |  | | |  | | -3.45 | 0.00899 |
| 34449_at | *CASP2* | | | caspase 2, apoptosis-related cysteine peptidase | | -3.45 | 0.01059 |
| 235016_at | *REEP3* | | | receptor accessory protein 3 | | -3.45 | 0.01208 |
| 228980_at |  | | |  | | -3.46 | 0.00063 |
| 238959_at | *LARP4* | | | La ribonucleoprotein domain family, member 4 | | -3.46 | 0.00205 |
| 222850_s_at | *DNAJB14* | | | DnaJ (Hsp40) homolog, subfamily B, member 14 | | -3.46 | 0.00217 |
| 227444_at | *ARMCX4* | | | armadillo repeat containing, X-linked 4 | | -3.46 | 0.00267 |
| 223258_s_at | *G2E3* | | | G2/M-phase specific E3 ubiquitin protein ligase | | -3.46 | 0.01263 |
| 212920_at | *REST* | | | RE1-silencing transcription factor | | -3.46 | 0.01346 |
| 204036_at | *LPAR1* | | | lysophosphatidic acid receptor 1 | | -3.46 | 0.01551 |
| 212651_at | *RHOBTB1* | | | Rho-related BTB domain containing 1 | | -3.47 | 0.00316 |
| 237939_at | *EPHA5* | | | EPH receptor A5 | | -3.47 | 0.0035 |
| 224047_at |  | | |  | | -3.47 | 0.00605 |
| 225326_at | *RBM27* | | | RNA binding motif protein 27 | | -3.47 | 0.00816 |
| 222497_x_at | *NMD3* | | | NMD3 homolog (S. cerevisiae) | | -3.47 | 0.00842 |
| 217834_s_at | *SYNCRIP* | | | synaptotagmin binding, cytoplasmic RNA interacting protein | | -3.47 | 0.00987 |
| 212511_at | *PICALM* | | | phosphatidylinositol binding clathrin assembly protein | | -3.47 | 0.01845 |
| 1553575_at | *ND6* | | | NADH dehydrogenase, subunit 6 (complex I) | | -3.48 | 0.0001 |
| 218883_s_at | *MLF1IP* | | | MLF1 interacting protein | | -3.48 | 0.0041 |
| 238474_at | *NUP43* | | | nucleoporin 43kDa | | -3.48 | 0.00508 |
| 227801_at | *TRIM59* | | | tripartite motif containing 59 | | -3.48 | 0.00683 |
| 207057_at | *SLC16A7* | | | solute carrier family 16, member 7 (monocarboxylic acid transporter 2) | | -3.48 | 0.00797 |
| 240712_s_at |  | | |  | | -3.48 | 0.00956 |
| 220992_s_at | *TRMT1L* | | | tRNA methyltransferase 1 homolog (S. cerevisiae)-like | | -3.48 | 0.01037 |
| 218334_at | *THOC7* | | | THO complex 7 homolog (Drosophila) | | -3.48 | 0.01811 |
| 219003_s_at | *MANEA* | | | mannosidase, endo-alpha | | -3.48 | 0.02096 |
| 204905_s_at | *EEF1E1* | | | eukaryotic translation elongation factor 1 epsilon 1 | | -3.49 | 0.00017 |
| 227971_at | *NRK* | | | Nik related kinase | | -3.49 | 0.00253 |
| 204700_x_at | *DIEXF* | | | digestive organ expansion factor homolog (zebrafish) | | -3.49 | 0.00403 |
| 231042_s_at |  | | |  | | -3.49 | 0.00455 |
| 209544_at | *RIPK2* | | | receptor-interacting serine-threonine kinase 2 | | -3.49 | 0.00666 |
| 242691_at |  | | |  | | -3.49 | 0.00727 |
| 1553150_at | *KDM1B* | | | lysine (K)-specific demethylase 1B | | -3.49 | 0.01309 |
| 222113_s_at | *EPS15L1* | | | epidermal growth factor receptor pathway substrate 15-like 1 | | -3.5 | 0.00183 |
| 214109_at | *LRBA* | | | LPS-responsive vesicle trafficking, beach and anchor containing | | -3.5 | 0.00281 |
| 209476_at | *TMX1* | | | thioredoxin-related transmembrane protein 1 | | -3.5 | 0.00761 |
| 235177_at | *METTL21A* | | | methyltransferase like 21A | | -3.51 | 0.00072 |
| 226847_at | *FST* | | | follistatin | | -3.51 | 0.00096 |
| 209811_at | *CASP2* | | | caspase 2, apoptosis-related cysteine peptidase | | -3.51 | 0.00121 |
| 235610_at | *ALKBH8* | | | alkB, alkylation repair homolog 8 (E. coli) | | -3.51 | 0.00314 |
| 224866_at | *FAR1* | | | fatty acyl CoA reductase 1 | | -3.51 | 0.00327 |
| 236422_at |  | | |  | | -3.51 | 0.00661 |
| 209187_at | *DR1* | | | down-regulator of transcription 1, TBP-binding (negative cofactor 2) | | -3.51 | 0.01 |
| 1552721_a_at | *FGF1* | | | fibroblast growth factor 1 (acidic) | | -3.51 | 0.02128 |
| 227545_at | *BARD1* | | | BRCA1 associated RING domain 1 | | -3.52 | 0.00533 |
| 209451_at | *TANK* | | | TRAF family member-associated NFKB activator | | -3.52 | 0.00783 |
| 219926_at | *POPDC3* | | | popeye domain containing 3 | | -3.53 | 0.00101 |
| 226003_at | *KIF21A* | | | kinesin family member 21A | | -3.53 | 0.00226 |
| 203584_at | *EMC2* | | | ER membrane protein complex subunit 2 | | -3.53 | 0.00256 |
| 1564637_a_at | *FAM98B* | | | family with sequence similarity 98, member B | | -3.53 | 0.00322 |
| 1558692_at | *C1orf85* | | | chromosome 1 open reading frame 85 | | -3.53 | 0.00468 |
| 206989_s_at | *SCAF11* | | | SR-related CTD-associated factor 11 | | -3.53 | 0.00597 |
| 223239_at | *GSKIP* | | | GSK3B interacting protein | | -3.53 | 0.00765 |
| 202118_s_at | *CPNE3* | | | copine III | | -3.53 | 0.01645 |
| 202886_s_at | *PPP2R1B* | | | protein phosphatase 2, regulatory subunit A, beta | | -3.54 | 0.00076 |
| 202314_at | *CYP51A1* | | | cytochrome P450, family 51, subfamily A, polypeptide 1; leucine-rich repeats and death domain containing 1 | | -3.54 | 0.00229 |
| 205347_s_at |  | | |  | | -3.54 | 0.00268 |
| 222787_s_at | *TMEM106B* | | | transmembrane protein 106B | | -3.54 | 0.00477 |
| 1553691_at | *B3GALNT2* | | | beta-1,3-N-acetylgalactosaminyltransferase 2 | | -3.54 | 0.0076 |
| 226834_at |  | | |  | | -3.55 | 0.00012 |
| 216837_at | *EPHA5* | | | EPH receptor A5 | | -3.55 | 0.00037 |
| 59705_at | *SCLY* | | | selenocysteine lyase | | -3.55 | 0.00048 |
| 225748_at | *LTV1* | | | LTV1 homolog (S. cerevisiae) | | -3.55 | 0.00117 |
| 212459_x_at | *SUCLG2* | | | succinate-CoA ligase, GDP-forming, beta subunit | | -3.55 | 0.00726 |
| 202577_s_at | *DDX19A* | | | DEAD (Asp-Glu-Ala-Asp) box polypeptide 19A | | -3.55 | 0.00899 |
| 213761_at | *MDM1* | | | Mdm1 nuclear protein homolog (mouse) | | -3.55 | 0.01935 |
| 217547_x_at | *ZNF675* | | | zinc finger protein 675 | | -3.55 | 0.02005 |
| 209377_s_at | *HMGN3* | | | high mobility group nucleosomal binding domain 3 | | -3.56 | 0.00122 |
| 228559_at | *CENPN* | | | centromere protein N | | -3.56 | 0.00239 |
| 201586_s_at | *LOC100996496* | | | uncharacterized LOC100996496; splicing factor proline/glutamine-rich | | -3.56 | 0.00253 |
| 224737_x_at | *CCAR1* | | | cell division cycle and apoptosis regulator 1 | | -3.56 | 0.00315 |
| 226760_at | *MBTPS2* | | | membrane-bound transcription factor peptidase, site 2 | | -3.56 | 0.00367 |
| 224315_at | *DDX20* | | | DEAD (Asp-Glu-Ala-Asp) box polypeptide 20 | | -3.56 | 0.00422 |
| 242122_at |  | | |  | | -3.56 | 0.01145 |
| 206026_s_at | *TNFAIP6* | | | tumor necrosis factor, alpha-induced protein 6 | | -3.56 | 0.01361 |
| 241912_at | *ZNF814* | | | zinc finger protein 814 | | -3.56 | 0.01449 |
| 1552472_a_at | *ACAP2* | | | ArfGAP with coiled-coil, ankyrin repeat and PH domains 2 | | -3.56 | 0.01536 |
| 231576_at |  | | |  | | -3.57 | 0.00166 |
| 1554113_a_at | *SLC4A8* | | | solute carrier family 4, sodium bicarbonate cotransporter, member 8 | | -3.57 | 0.0075 |
| 1555037_a_at | *IDH1* | | | isocitrate dehydrogenase 1 (NADP+), soluble | | -3.58 | 0.00092 |
| 206306_at | *RYR3* | | | ryanodine receptor 3 | | -3.58 | 0.0011 |
| 220651_s_at | *MCM10* | | | minichromosome maintenance complex component 10 | | -3.58 | 0.00145 |
| 201025_at | *EIF5B* | | | eukaryotic translation initiation factor 5B | | -3.58 | 0.00175 |
| 226591_at |  | | |  | | -3.58 | 0.00443 |
| 220327_at | *VGLL3* | | | vestigial like 3 (Drosophila) | | -3.58 | 0.01057 |
| 1569495_at | *SCLT1* | | | sodium channel and clathrin linker 1 | | -3.58 | 0.01079 |
| 209071_s_at | *RGS5* | | | regulator of G-protein signaling 5 | | -3.58 | 0.01318 |
| 204237_at | *GULP1* | | | GULP, engulfment adaptor PTB domain containing 1 | | -3.58 | 0.02047 |
| 220038_at |  | | |  | | -3.58 | 0.02537 |
| 205063_at | *GEMIN2* | | | gem (nuclear organelle) associated protein 2 | | -3.59 | 0.00045 |
| 212714_at | *LARP4* | | | La ribonucleoprotein domain family, member 4 | | -3.59 | 0.00048 |
| 223542_at | *ANKRD32* | | | ankyrin repeat domain 32 | | -3.59 | 0.00255 |
| 212476_at | *ACAP2* | | | ArfGAP with coiled-coil, ankyrin repeat and PH domains 2 | | -3.59 | 0.01378 |
| 1552658_a_at | *NAV3* | | | neuron navigator 3 | | -3.6 | 0.0007 |
| 229491_at | *SLC9B2* | | | solute carrier family 9, subfamily B (NHA2, cation proton antiporter 2), member 2 | | -3.6 | 0.00106 |
| 219459_at | *POLR3B* | | | polymerase (RNA) III (DNA directed) polypeptide B | | -3.6 | 0.00107 |
| 266_s_at | *CD24* | | | CD24 molecule | | -3.6 | 0.00213 |
| 220553_s_at | *PRPF39* | | | PRP39 pre-mRNA processing factor 39 homolog (S. cerevisiae) | | -3.6 | 0.008 |
| 209298_s_at | *ITSN1* | | | intersectin 1 (SH3 domain protein) | | -3.6 | 0.01399 |
| 225290_at | *ETNK1* | | | ethanolamine kinase 1 | | -3.61 | 0.00283 |
| 212230_at | *PPAP2B* | | | phosphatidic acid phosphatase type 2B | | -3.61 | 0.00349 |
| 215047_at | *TRIM58* | | | tripartite motif containing 58 | | -3.61 | 0.00835 |
| 219148_at | *PBK* | | | PDZ binding kinase | | -3.62 | 0.00503 |
| 226339_at | *TRUB1* | | | TruB pseudouridine (psi) synthase homolog 1 (E. coli) | | -3.62 | 0.00605 |
| 226510_at | *HEATR5A* | | | HEAT repeat containing 5A | | -3.62 | 0.00641 |
| 227718_at | *PURB* | | | purine-rich element binding protein B | | -3.62 | 0.00652 |
| 230029_x_at | *UBR3* | | | ubiquitin protein ligase E3 component n-recognin 3 (putative) | | -3.62 | 0.01002 |
| 225361_x_at | *FAM122B* | | | family with sequence similarity 122B | | -3.62 | 0.02498 |
| 205034_at | *CCNE2* | | | cyclin E2 | | -3.62 | 0.03114 |
| 239668_at |  | | |  | | -3.63 | 0.00066 |
| 222794_x_at | *MTPAP* | | | mitochondrial poly(A) polymerase | | -3.63 | 0.00169 |
| 46947_at | *GNL3L* | | | guanine nucleotide binding protein-like 3 (nucleolar)-like | | -3.63 | 0.00219 |
| 219973_at | *ARSJ* | | | arylsulfatase family, member J | | -3.63 | 0.01098 |
| 212634_at | *UFL1* | | | UFM1-specific ligase 1 | | -3.63 | 0.01159 |
| 226994_at | *DNAJA2* | | | DnaJ (Hsp40) homolog, subfamily A, member 2 | | -3.63 | 0.01786 |
| 222428_s_at | *LARS* | | | leucyl-tRNA synthetase | | -3.64 | 0.0034 |
| 201972_at | *ATP6V1A* | | | ATPase, H+ transporting, lysosomal 70kDa, V1 subunit A | | -3.64 | 0.00613 |
| 225344_at | *NCOA7* | | | nuclear receptor coactivator 7 | | -3.64 | 0.00899 |
| 211124_s_at | *KITLG* | | | KIT ligand | | -3.65 | 0.00118 |
| 241734_at | *SRFBP1* | | | serum response factor binding protein 1 | | -3.65 | 0.0021 |
| 208788_at | *ELOVL5* | | | ELOVL fatty acid elongase 5 | | -3.65 | 0.00212 |
| 214039_s_at | *LAPTM4B* | | | lysosomal protein transmembrane 4 beta | | -3.65 | 0.00345 |
| 52285_f_at | *CEP76* | | | centrosomal protein 76kDa | | -3.65 | 0.00349 |
| 208152_s_at | *DDX21* | | | DEAD (Asp-Glu-Ala-Asp) box helicase 21 | | -3.65 | 0.00421 |
| 204337_at | *RGS4* | | | regulator of G-protein signaling 4 | | -3.65 | 0.00497 |
| 242648_at | *KLHL8* | | | kelch-like family member 8 | | -3.65 | 0.00567 |
| 212213_x_at | *OPA1* | | | optic atrophy 1 (autosomal dominant) | | -3.65 | 0.00696 |
| 222825_at | *OTUD6B* | | | OTU domain containing 6B | | -3.65 | 0.00924 |
| 225158_at | *GFM1* | | | G elongation factor, mitochondrial 1 | | -3.66 | 0.0042 |
| 203881_s_at | *DMD* | | | dystrophin | | -3.66 | 0.00437 |
| 222811_at | *FTSJD1* | | | FtsJ methyltransferase domain containing 1 | | -3.66 | 0.00931 |
| 211220_s_at | *HSF2* | | | heat shock transcription factor 2 | | -3.66 | 0.01193 |
| 212797_at | *SORT1* | | | sortilin 1 | | -3.67 | 0.00157 |
| 204820_s_at | *BTN3A2* | | | butyrophilin, subfamily 3, member A2; butyrophilin, subfamily 3, member A3 | | -3.67 | 0.00182 |
| 225161_at | *GFM1* | | | G elongation factor, mitochondrial 1 | | -3.67 | 0.00732 |
| 241342_at | *TMEM65* | | | transmembrane protein 65 | | -3.67 | 0.01308 |
| 200989_at | *HIF1A* | | | hypoxia inducible factor 1, alpha subunit (basic helix-loop-helix transcription factor) | | -3.68 | 0.00012 |
| 203810_at | *DNAJB4* | | | DnaJ (Hsp40) homolog, subfamily B, member 4 | | -3.68 | 0.00631 |
| 201291_s_at | *TOP2A* | | | topoisomerase (DNA) II alpha 170kDa | | -3.68 | 0.00771 |
| 229194_at | *PCGF5* | | | polycomb group ring finger 5 | | -3.68 | 0.0181 |
| 1552359_at | *MCMDC2* | | | minichromosome maintenance domain containing 2 | | -3.69 | 0.00126 |
| 218397_at | *FANCL* | | | Fanconi anemia, complementation group L | | -3.69 | 0.00416 |
| 218139_s_at | *AP5M1* | | | adaptor-related protein complex 5, mu 1 subunit | | -3.69 | 0.00468 |
| 241404_at |  | | |  | | -3.7 | 0.00409 |
| 1552291_at | *PIGX* | | | phosphatidylinositol glycan anchor biosynthesis, class X | | -3.7 | 0.00553 |
| 243727_at | *CPNE8* | | | copine VIII | | -3.7 | 0.00662 |
| 203347_s_at | *MTF2* | | | metal response element binding transcription factor 2 | | -3.71 | 0.00557 |
| 218150_at | *ARL5A* | | | ADP-ribosylation factor-like 5A | | -3.71 | 0.00947 |
| 222906_at | *FLVCR1* | | | feline leukemia virus subgroup C cellular receptor 1 | | -3.71 | 0.01083 |
| 218499_at | *MST4* | | | serine/threonine protein kinase MST4 | | -3.72 | 0.00139 |
| 223374_s_at | *B3GALNT1* | | | beta-1,3-N-acetylgalactosaminyltransferase 1 (globoside blood group) | | -3.72 | 0.01136 |
| 231921_at | *DCAF17* | | | DDB1 and CUL4 associated factor 17 | | -3.72 | 0.0199 |
| 201324_at | *EMP1* | | | epithelial membrane protein 1 | | -3.73 | 0.00412 |
| 225716_at | *BRI3BP* | | | BRI3 binding protein | | -3.73 | 0.00705 |
| 212686_at | *PPM1H* | | | protein phosphatase, Mg2+/Mn2+ dependent, 1H | | -3.73 | 0.0079 |
| 222344_at |  | | |  | | -3.73 | 0.01095 |
| 225785_at | *REEP3* | | | receptor accessory protein 3 | | -3.73 | 0.01412 |
| 226109_at | *C21orf91* | | | chromosome 21 open reading frame 91 | | -3.74 | 0.0036 |
| 204493_at | *BID* | | | BH3 interacting domain death agonist | | -3.74 | 0.00377 |
| 226178_at | *SOCS4* | | | suppressor of cytokine signaling 4 | | -3.74 | 0.00507 |
| 224751_at | *C7orf73* | | | chromosome 7 open reading frame 73 | | -3.74 | 0.00584 |
| 204886_at | *PLK4* | | | polo-like kinase 4 | | -3.74 | 0.00648 |
| 229700_at | *ZNF738* | | | zinc finger protein 738 | | -3.74 | 0.02362 |
| 204120_s_at | *ADK* | | | adenosine kinase | | -3.74 | 0.02366 |
| 1552309_a_at | *NEXN* | | | nexilin (F actin binding protein) | | -3.75 | 0.00625 |
| 202543_s_at | *GMFB* | | | glia maturation factor, beta | | -3.75 | 0.00674 |
| 212928_at | *TSPYL4* | | | TSPY-like 4 | | -3.75 | 0.00689 |
| 235394_at | *PLAA* | | | phospholipase A2-activating protein | | -3.75 | 0.00773 |
| 204749_at | *NAP1L3* | | | nucleosome assembly protein 1-like 3 | | -3.76 | 0.00255 |
| 222466_s_at | *MRPL42* | | | mitochondrial ribosomal protein L42 | | -3.76 | 0.00557 |
| 238448_at | *MRPL19* | | | mitochondrial ribosomal protein L19 | | -3.76 | 0.00623 |
| 219293_s_at | *OLA1* | | | Obg-like ATPase 1 | | -3.76 | 0.00816 |
| 226860_at | *TMEM19* | | | transmembrane protein 19 | | -3.76 | 0.01529 |
| 228026_at | *SIKE1* | | | suppressor of IKBKE 1 | | -3.76 | 0.02904 |
| 237333_at | *RBBP4* | | | retinoblastoma binding protein 4 | | -3.77 | 0.00211 |
| 202503_s_at | *KIAA0101* | | | KIAA0101 | | -3.77 | 0.00466 |
| 209891_at | *SPC25* | | | SPC25, NDC80 kinetochore complex component | | -3.77 | 0.00698 |
| 228680_at | *KIF3A* | | | kinesin family member 3A | | -3.77 | 0.00797 |
| 213906_at | *MYBL1* | | | v-myb myeloblastosis viral oncogene homolog (avian)-like 1 | | -3.77 | 0.01105 |
| 1552660_a_at | *C5orf22* | | | chromosome 5 open reading frame 22 | | -3.78 | 0.00188 |
| 227278_at | *TAF13* | | | TAF13 RNA polymerase II, TATA box binding protein (TBP)-associated factor, 18kDa | | -3.78 | 0.00551 |
| 202437_s_at | *CYP1B1* | | | cytochrome P450, family 1, subfamily B, polypeptide 1 | | -3.78 | 0.01185 |
| 229544_at |  | | |  | | -3.79 | 0.01075 |
| 1556783_a_at |  | | |  | | -3.8 | 0.00706 |
| 229980_s_at | *SNX5* | | | sorting nexin 5 | | -3.8 | 0.0094 |
| 235117_at | *CHAC2* | | | ChaC, cation transport regulator homolog 2 (E. coli) | | -3.8 | 0.01606 |
| 201242_s_at | *ATP1B1* | | | ATPase, Na+/K+ transporting, beta 1 polypeptide | | -3.8 | 0.01633 |
| 228654_at | *SPIN4* | | | spindlin family, member 4 | | -3.8 | 0.03659 |
| 218738_s_at | *RNF138* | | | ring finger protein 138, E3 ubiquitin protein ligase | | -3.81 | 0.00176 |
| 204809_at | *CLPX* | | | ClpX caseinolytic peptidase X homolog (E. coli) | | -3.81 | 0.00198 |
| 1568191_at |  | | |  | | -3.81 | 0.00252 |
| 1561177_at |  | | |  | | -3.81 | 0.02216 |
| 209384_at | *PROSC* | | | proline synthetase co-transcribed homolog (bacterial) | | -3.82 | 0.0013 |
| 228638_at |  | | |  | | -3.83 | 0.01834 |
| 204602_at | *DKK1* | | | dickkopf WNT signaling pathway inhibitor 1 | | -3.85 | 0.00799 |
| 1552680_a_at | *CASC5* | | | cancer susceptibility candidate 5 | | -3.85 | 0.01188 |
| 226641_at | *ANKRD44* | | | ankyrin repeat domain 44 | | -3.85 | 0.01255 |
| 213238_at | *ATP10D* | | | ATPase, class V, type 10D | | -3.86 | 0.00166 |
| 224944_at | *TMPO* | | | thymopoietin | | -3.86 | 0.00296 |
| 224231_at | *PRO0471* | | | uncharacterized LOC28994 | | -3.86 | 0.00589 |
| 228370_at |  | | |  | | -3.87 | 0.00505 |
| 225674_at | *BCAP29* | | | B-cell receptor-associated protein 29 | | -3.87 | 0.01144 |
| 225470_at | *NUP35* | | | nucleoporin 35kDa | | -3.88 | 0.0019 |
| 242890_at |  | | |  | | -3.88 | 0.00248 |
| 202169_s_at | *AASDHPPT* | | | aminoadipate-semialdehyde dehydrogenase-phosphopantetheinyl transferase | | -3.88 | 0.00487 |
| 222771_s_at | *MYEF2* | | | myelin expression factor 2 | | -3.88 | 0.00888 |
| 232197_x_at | *ARSB* | | | arylsulfatase B | | -3.88 | 0.01038 |
| 223243_s_at | *EDEM3* | | | ER degradation enhancer, mannosidase alpha-like 3 | | -3.89 | 0.00522 |
| 227230_s_at | *KIAA1211* | | | KIAA1211 | | -3.89 | 0.00556 |
| 225302_at | *TMX3* | | | thioredoxin-related transmembrane protein 3 | | -3.89 | 0.00712 |
| 201088_at | *KPNA2* | | | karyopherin alpha 2 (RAG cohort 1, importin alpha 1) | | -3.89 | 0.01089 |
| 224151_s_at | *AK3* | | | adenylate kinase 3 | | -3.89 | 0.0111 |
| 218254_s_at | *SAR1B* | | | SAR1 homolog B (S. cerevisiae) | | -3.9 | 0.00178 |
| 1556186_s_at | *EMC1* | | | ER membrane protein complex subunit 1 | | -3.9 | 0.00564 |
| 209099_x_at | *JAG1* | | | jagged 1 | | -3.9 | 0.00575 |
| 202581_at | *HSPA1A* | | | heat shock 70kDa protein 1A; heat shock 70kDa protein 1B | | -3.91 | 0.00087 |
| 204641_at | *NEK2* | | | NIMA-related kinase 2 | | -3.91 | 0.00253 |
| 227766_at | *LIG4* | | | ligase IV, DNA, ATP-dependent | | -3.91 | 0.00505 |
| 227110_at | *HNRNPC* | | | heterogeneous nuclear ribonucleoprotein C (C1/C2) | | -3.91 | 0.00835 |
| 214949_at |  | | |  | | -3.91 | 0.01101 |
| 223065_s_at | *STARD3NL* | | | STARD3 N-terminal like | | -3.92 | 0.00585 |
| 226596_x_at |  | | |  | | -3.92 | 0.02438 |
| 210371_s_at | *RBBP4* | | | retinoblastoma binding protein 4 | | -3.93 | 0.0013 |
| 226106_at | *RNF141* | | | ring finger protein 141 | | -3.93 | 0.00177 |
| 208802_at | *SRP72* | | | signal recognition particle 72kDa | | -3.93 | 0.00494 |
| 234984_at | *NEDD1* | | | neural precursor cell expressed, developmentally down-regulated 1 | | -3.93 | 0.0094 |
| 209392_at | *ENPP2* | | | ectonucleotide pyrophosphatase/phosphodiesterase 2 | | -3.94 | 0.00077 |
| 209838_at | *COPS2* | | | COP9 signalosome subunit 2 | | -3.94 | 0.00447 |
| 204071_s_at | *TOPORS* | | | topoisomerase I binding, arginine/serine-rich, E3 ubiquitin protein ligase | | -3.95 | 0.0024 |
| 201083_s_at | *BCLAF1* | | | BCL2-associated transcription factor 1 | | -3.96 | 0.0026 |
| 238852_at | *PRRX1* | | | paired related homeobox 1 | | -3.96 | 0.00859 |
| 201889_at | *FAM3C* | | | family with sequence similarity 3, member C | | -3.96 | 0.01371 |
| 203775_at | *SLC25A13* | | | solute carrier family 25 (aspartate/glutamate carrier), member 13 | | -3.97 | 0.00136 |
| 1555274_a_at | *EPT1* | | | ethanolaminephosphotransferase 1 (CDP-ethanolamine-specific) | | -3.98 | 0.00139 |
| 202693_s_at | *STK17A* | | | serine/threonine kinase 17a | | -3.98 | 0.00314 |
| 218853_s_at | *MOSPD1* | | | motile sperm domain containing 1 | | -3.98 | 0.00562 |
| 200887_s_at | *STAT1* | | | signal transducer and activator of transcription 1, 91kDa | | -3.99 | 0.00094 |
| 213149_at | *DLAT* | | | dihydrolipoamide S-acetyltransferase | | -3.99 | 0.00475 |
| 223766_at |  | | |  | | -4 | 0.00937 |
| 228189_at | *BAG4* | | | BCL2-associated athanogene 4 | | -4 | 0.01617 |
| 223830_s_at | *TRIM5* | | | tripartite motif containing 5 | | -4 | 0.02926 |
| 239392_s_at | *POGK* | | | pogo transposable element with KRAB domain | | -4.03 | 0.0087 |
| 244162_at |  | | |  | | -4.03 | 0.01906 |
| 225994_at | *CPSF2* | | | cleavage and polyadenylation specific factor 2, 100kDa | | -4.04 | 0.00172 |
| 229299_at | *NADK2* | | | NAD kinase 2, mitochondrial | | -4.04 | 0.01685 |
| 223089_at | *VEZT* | | | vezatin, adherens junctions transmembrane protein | | -4.05 | 0.00314 |
| 230416_at |  | | |  | | -4.05 | 0.00467 |
| 212820_at | *DMXL2* | | | Dmx-like 2 | | -4.06 | 0.0062 |
| 225535_s_at | *TIMM23* | | | translocase of inner mitochondrial membrane 23 homolog (yeast); translocase of inner mitochondrial membrane 23 homolog B (yeast) | | -4.07 | 0.00292 |
| 217678_at | *SLC7A11* | | | solute carrier family 7 (anionic amino acid transporter light chain, xc- system), member 11 | | -4.08 | 0.00276 |
| 239960_x_at | *LYRM7* | | | LYR motif containing 7 | | -4.08 | 0.00628 |
| 225944_at | *NLN* | | | neurolysin (metallopeptidase M3 family) | | -4.09 | 0.00447 |
| 227280_s_at | *CCNYL1* | | | cyclin Y-like 1 | | -4.09 | 0.00517 |
| 228785_at |  | | |  | | -4.09 | 0.00608 |
| 212847_at |  | | |  | | -4.09 | 0.01967 |
| 201744_s_at | *LUM* | | | lumican | | -4.1 | 0.00077 |
| 212824_at | *FUBP3* | | | far upstream element (FUSE) binding protein 3 | | -4.1 | 0.0057 |
| 1553721_at | *RNF152* | | | ring finger protein 152 | | -4.11 | 0.0013 |
| 242140_at | *ERVK3-1* | | | endogenous retrovirus group K3, member 1 | | -4.12 | 0.00336 |
| 219787_s_at | *ECT2* | | | epithelial cell transforming sequence 2 oncogene | | -4.12 | 0.00462 |
| 202230_s_at | *CHERP* | | | calcium homeostasis endoplasmic reticulum protein | | -4.12 | 0.00703 |
| 206579_at | *ZKSCAN8* | | | zinc finger with KRAB and SCAN domains 8 | | -4.12 | 0.00767 |
| 230078_at | *RAPGEF6* | | | Rap guanine nucleotide exchange factor (GEF) 6 | | -4.12 | 0.01982 |
| 1554885_a_at |  | | |  | | -4.13 | 0.00126 |
| 212449_s_at | *LYPLA1* | | | lysophospholipase I | | -4.13 | 0.00198 |
| 224691_at | *UHMK1* | | | U2AF homology motif (UHM) kinase 1 | | -4.13 | 0.00991 |
| 219935_at | *ADAMTS5* | | | ADAM metallopeptidase with thrombospondin type 1 motif, 5 | | -4.13 | 0.02542 |
| 209903_s_at | *ATR* | | | ataxia telangiectasia and Rad3 related | | -4.14 | 0.00046 |
| 204728_s_at | *WDHD1* | | | WD repeat and HMG-box DNA binding protein 1 | | -4.14 | 0.00188 |
| 216268_s_at | *JAG1* | | | jagged 1 | | -4.15 | 0.00542 |
| 1558700_s_at | *ZNF260* | | | zinc finger protein 260 | | -4.15 | 0.00612 |
| 224723_x_at |  | | |  | | -4.15 | 0.00954 |
| 218542_at | *CEP55* | | | centrosomal protein 55kDa | | -4.15 | 0.01525 |
| 226541_at | *FBXO30* | | | F-box protein 30 | | -4.16 | 0.00217 |
| 232071_at | *MRPL19* | | | mitochondrial ribosomal protein L19 | | -4.16 | 0.04346 |
| 241362_at | *SOGA1* | | | suppressor of glucose, autophagy associated 1 | | -4.17 | 0.00975 |
| 222731_at | *ZDHHC2* | | | zinc finger, DHHC-type containing 2 | | -4.17 | 0.01671 |
| 228853_at | *STYX* | | | serine/threonine/tyrosine interacting protein | | -4.18 | 0.00265 |
| 233252_s_at | *STRBP* | | | spermatid perinuclear RNA binding protein | | -4.18 | 0.00346 |
| 225684_at | *SKA2* | | | spindle and kinetochore associated complex subunit 2 | | -4.18 | 0.01086 |
| 204127_at | *RFC3* | | | replication factor C (activator 1) 3, 38kDa | | -4.19 | 0.00043 |
| 223319_at | *GPHN* | | | gephyrin | | -4.19 | 0.00115 |
| 212709_at | *NUP160* | | | nucleoporin 160kDa | | -4.19 | 0.00539 |
| 203870_at | *USP46* | | | ubiquitin specific peptidase 46 | | -4.19 | 0.00724 |
| 212306_at | *CLASP2* | | | cytoplasmic linker associated protein 2 | | -4.2 | 0.00288 |
| 243887_at | *MRPL30* | | | mitochondrial ribosomal protein L30 | | -4.2 | 0.00448 |
| 213907_at | *EEF1E1* | | | eukaryotic translation elongation factor 1 epsilon 1 | | -4.21 | 2.7x10^-5^ |
| 224960_at | *SCYL2* | | | SCY1-like 2 (S. cerevisiae) | | -4.21 | 0.00308 |
| 201873_s_at | *ABCE1* | | | ATP-binding cassette, sub-family E (OABP), member 1 | | -4.22 | 0.00072 |
| 204092_s_at | *AURKA* | | | aurora kinase A | | -4.22 | 0.00424 |
| 214981_at | *POSTN* | | | periostin, osteoblast specific factor | | -4.23 | 0.00022 |
| 204286_s_at | *PMAIP1* | | | phorbol-12-myristate-13-acetate-induced protein 1 | | -4.23 | 0.00884 |
| 227947_at | *PHACTR2* | | | phosphatase and actin regulator 2 | | -4.23 | 0.01891 |
| 230205_at | *ZNF561* | | | zinc finger protein 561 | | -4.24 | 0.00048 |
| 218332_at | *BEX1* | | | brain expressed, X-linked 1 | | -4.24 | 0.00133 |
| 211675_s_at | *MDFIC* | | | MyoD family inhibitor domain containing | | -4.24 | 0.00146 |
| 204557_s_at | *DZIP1* | | | DAZ interacting zinc finger protein 1 | | -4.24 | 0.00258 |
| 204823_at | *NAV3* | | | neuron navigator 3 | | -4.28 | 0.00395 |
| 1562303_at | *ZKSCAN3* | | | zinc finger with KRAB and SCAN domains 3 | | -4.28 | 0.01922 |
| 227158_at | *DTD2* | | | D-tyrosyl-tRNA deacylase 2 (putative) | | -4.31 | 0.00155 |
| 224961_at | *SCYL2* | | | SCY1-like 2 (S. cerevisiae) | | -4.31 | 0.00475 |
| 225492_at | *TMEM33* | | | transmembrane protein 33 | | -4.31 | 0.00608 |
| 235722_at | *SYNJ2BP* | | | synaptojanin 2 binding protein | | -4.31 | 0.01018 |
| 208828_at | *POLE3* | | | polymerase (DNA directed), epsilon 3, accessory subunit | | -4.32 | 0.00071 |
| 206055_s_at | *SNRPA1* | | | small nuclear ribonucleoprotein polypeptide A' | | -4.32 | 0.00635 |
| 213294_at | *EIF2AK2* | | | eukaryotic translation initiation factor 2-alpha kinase 2 | | -4.32 | 0.00819 |
| 204405_x_at | *DIMT1* | | | DIM1 dimethyladenosine transferase 1 homolog (S. cerevisiae) | | -4.33 | 0.00111 |
| 222624_s_at | *ZNF639* | | | zinc finger protein 639 | | -4.33 | 0.00246 |
| 226399_at | *DNAJB14* | | | DnaJ (Hsp40) homolog, subfamily B, member 14 | | -4.33 | 0.00286 |
| 201436_at | *EIF4E* | | | eukaryotic translation initiation factor 4E | | -4.33 | 0.00791 |
| 242445_at | *FGD4* | | | FYVE, RhoGEF and PH domain containing 4 | | -4.35 | 0.00195 |
| 201437_s_at | *EIF4E* | | | eukaryotic translation initiation factor 4E | | -4.35 | 0.00512 |
| 223335_at | *TMEM69* | | | transmembrane protein 69 | | -4.36 | 0.00433 |
| 207117_at | *ZNF117* | | | zinc finger protein 117 | | -4.37 | 0.00832 |
| 225973_at | *TAP2* | | | transporter 2, ATP-binding cassette, sub-family B (MDR/TAP) | | -4.39 | 0.00022 |
| 227239_at | *FAM126A* | | | family with sequence similarity 126, member A | | -4.39 | 0.00533 |
| 222490_at |  | | |  | | -4.41 | 0.00568 |
| 1569516_at |  | | |  | | -4.42 | 0.00153 |
| 210293_s_at | *SEC23B* | | | Sec23 homolog B (S. cerevisiae) | | -4.42 | 0.00396 |
| 212279_at | *TMEM97* | | | transmembrane protein 97 | | -4.43 | 0.00078 |
| 214975_s_at | *MTMR1* | | | myotubularin related protein 1 | | -4.43 | 0.01764 |
| 229861_at |  | | |  | | -4.44 | 0.00062 |
| 210868_s_at | *ELOVL6* | | | ELOVL fatty acid elongase 6 | | -4.44 | 0.00156 |
| 226617_at | *ARL5A* | | | ADP-ribosylation factor-like 5A | | -4.44 | 0.00495 |
| 228238_at | *GAS5* | | | growth arrest-specific 5 (non-protein coding); small nucleolar RNA, C/D box 44; small nucleolar RNA, C/D box 47; small nucleolar RNA, C/D box 76; small nucleolar RNA, C/D box 77; small nucleolar RNA, C/D box 79; small nucleolar RNA, C/D box 80; small nucleolar RNA, C/D box 81 | | -4.45 | 0.00197 |
| 209709_s_at | *HMMR* | | | hyaluronan-mediated motility receptor (RHAMM) | | -4.45 | 0.01048 |
| 235121_at | *ZNF542* | | | zinc finger protein 542 | | -4.46 | 0.00506 |
| 1555878_at | *RPS24* | | | ribosomal protein S24 | | -4.46 | 0.01187 |
| 219934_s_at | *SULT1E1* | | | sulfotransferase family 1E, estrogen-preferring, member 1 | | -4.47 | 0.00015 |
| 202859_x_at | *IL8* | | | interleukin 8 | | -4.47 | 0.01903 |
| 1567379_at |  | | |  | | -4.47 | 0.02075 |
| 201468_s_at | *NQO1* | | | NAD(P)H dehydrogenase, quinone 1 | | -4.49 | 0.00024 |
| 217267_s_at | *RAB7A* | | | RAB7A, member RAS oncogene family | | -4.49 | 0.00047 |
| 238075_at | *CHEK1* | | | checkpoint kinase 1 | | -4.49 | 0.00754 |
| 222765_x_at | *ESF1* | | | ESF1, nucleolar pre-rRNA processing protein, homolog (S. cerevisiae) | | -4.49 | 0.01181 |
| 217599_s_at | *MDFIC* | | | MyoD family inhibitor domain containing | | -4.5 | 0.00222 |
| 1554679_a_at | *LAPTM4B* | | | lysosomal protein transmembrane 4 beta | | -4.52 | 0.00105 |
| 203343_at | *UGDH* | | | UDP-glucose 6-dehydrogenase | | -4.53 | 0.01093 |
| 203224_at | *RFK* | | | riboflavin kinase | | -4.54 | 0.00203 |
| 201435_s_at | *EIF4E* | | | eukaryotic translation initiation factor 4E | | -4.54 | 0.00366 |
| 218039_at | *NUSAP1* | | | nucleolar and spindle associated protein 1 | | -4.54 | 0.00585 |
| 202422_s_at | *ACSL4* | | | acyl-CoA synthetase long-chain family member 4 | | -4.55 | 0.01283 |
| 240277_at |  | | |  | | -4.56 | 0.00961 |
| 235030_at | *NXPE3* | | | neurexophilin and PC-esterase domain family, member 3 | | -4.57 | 0.00106 |
| 1567380_at |  | | |  | | -4.57 | 0.0054 |
| 213320_at | *PRMT3* | | | protein arginine methyltransferase 3 | | -4.58 | 0.00489 |
| 1559481_at | *CHIC1* | | | cysteine-rich hydrophobic domain 1 | | -4.59 | 0.02034 |
| 203595_s_at | *IFIT5* | | | interferon-induced protein with tetratricopeptide repeats 5 | | -4.6 | 0.00366 |
| 227268_at | *RNFT1* | | | ring finger protein, transmembrane 1 | | -4.6 | 0.0071 |
| 209181_s_at | *RABGGTB* | | | Rab geranylgeranyltransferase, beta subunit; small nucleolar RNA, C/D box 45A; small nucleolar RNA, C/D box 45B; small nucleolar RNA, C/D box 45C | | -4.61 | 0.00141 |
| 235004_at | *RBM24* | | | RNA binding motif protein 24 | | -4.61 | 0.01194 |
| 235238_at | *SHC4* | | | SHC (Src homology 2 domain containing) family, member 4 | | -4.64 | 0.00036 |
| 225580_at | *MRPL50* | | | mitochondrial ribosomal protein L50 | | -4.64 | 0.00118 |
| 224428_s_at | *CDCA7* | | | cell division cycle associated 7 | | -4.64 | 0.00133 |
| 202907_s_at | *NBN* | | | nibrin | | -4.64 | 0.0038 |
| 201292_at | *TOP2A* | | | topoisomerase (DNA) II alpha 170kDa | | -4.64 | 0.01687 |
| 201742_x_at | *SRSF1* | | | serine/arginine-rich splicing factor 1 | | -4.65 | 0.00287 |
| 1553106_at | *C5orf24* | | | chromosome 5 open reading frame 24 | | -4.65 | 0.00536 |
| 211078_s_at | *STK3* | | | serine/threonine kinase 3 | | -4.67 | 0.01894 |
| 218566_s_at | *CHORDC1* | | | cysteine and histidine-rich domain (CHORD) containing 1 | | -4.68 | 0.0026 |
| 231534_at | *CDK1* | | | cyclin-dependent kinase 1 | | -4.69 | 0.00034 |
| 223087_at | *ECHDC1* | | | enoyl CoA hydratase domain containing 1 | | -4.69 | 0.00107 |
| 218182_s_at | *CLDN1* | | | claudin 1 | | -4.71 | 0.00136 |
| 229075_at | *SPATA5* | | | spermatogenesis associated 5 | | -4.72 | 0.00872 |
| 232235_at | *DSEL* | | | dermatan sulfate epimerase-like | | -4.76 | 0.00424 |
| 212282_at | *TMEM97* | | | transmembrane protein 97 | | -4.77 | 0.0011 |
| 233970_s_at | *TRMT6* | | | tRNA methyltransferase 6 homolog (S. cerevisiae) | | -4.77 | 0.00248 |
| 236622_at | *PIGM* | | | phosphatidylinositol glycan anchor biosynthesis, class M | | -4.77 | 0.00294 |
| 225171_at | *ARHGAP18* | | | Rho GTPase activating protein 18 | | -4.81 | 0.00276 |
| 1565939_at | *C5orf22* | | | chromosome 5 open reading frame 22 | | -4.87 | 0.00074 |
| 217851_s_at | *SLMO2* | | | slowmo homolog 2 (Drosophila) | | -4.87 | 0.00354 |
| 227038_at | *SGMS2* | | | sphingomyelin synthase 2 | | -4.87 | 0.00683 |
| 226150_at | *PPAPDC1B* | | | phosphatidic acid phosphatase type 2 domain containing 1B | | -4.88 | 0.00392 |
| 204770_at | *TAP2* | | | transporter 2, ATP-binding cassette, sub-family B (MDR/TAP) | | -4.89 | 0.00088 |
| 1558014_s_at | *FAR1* | | | fatty acyl CoA reductase 1 | | -4.91 | 0.01314 |
| 218842_at | *RPAP3* | | | RNA polymerase II associated protein 3 | | -4.92 | 0.00204 |
| 236140_at | *GCLM* | | | glutamate-cysteine ligase, modifier subunit | | -4.93 | 0.00637 |
| 209803_s_at | *PHLDA2* | | | pleckstrin homology-like domain, family A, member 2 | | -4.93 | 0.0255 |
| 226302_at | *ATP8B1* | | | ATPase, aminophospholipid transporter, class I, type 8B, member 1 | | -4.95 | 0.01054 |
| 204058_at | *ME1* | | | malic enzyme 1, NADP(+)-dependent, cytosolic | | -4.97 | 0.00219 |
| 218793_s_at | *SCML1* | | | sex comb on midleg-like 1 (Drosophila) | | -4.98 | 0.00106 |
| 234304_s_at | *IPO11* | | | importin 11; IPO11-LRRC70 readthrough | | -5.01 | 0.00466 |
| 205046_at | *CENPE* | | | centromere protein E, 312kDa | | -5.02 | 0.01171 |
| 205097_at | *SLC26A2* | | | solute carrier family 26 (sulfate transporter), member 2 | | -5.04 | 0.01356 |
| 202270_at | *GBP1* | | | guanylate binding protein 1, interferon-inducible | | -5.05 | 0.00815 |
| 214590_s_at | *UBE2D1* | | | ubiquitin-conjugating enzyme E2D 1 | | -5.05 | 0.01084 |
| 206976_s_at | *HSPH1* | | | heat shock 105kDa/110kDa protein 1 | | -5.06 | 0.00043 |
| 217986_s_at | *BAZ1A* | | | bromodomain adjacent to zinc finger domain, 1A | | -5.06 | 0.0026 |
| 218351_at | *COMMD8* | | | COMM domain containing 8 | | -5.06 | 0.00512 |
| 209944_at | *ZNF410* | | | zinc finger protein 410 | | -5.08 | 0.00477 |
| 226022_at | *SASH1* | | | SAM and SH3 domain containing 1 | | -5.08 | 0.00497 |
| 209674_at | *CRY1* | | | cryptochrome 1 (photolyase-like) | | -5.11 | 0.00096 |
| 242191_at | *NBPF10* | | | neuroblastoma breakpoint family, member 10 | | -5.12 | 0.00182 |
| 225114_at | *AGPS* | | | alkylglycerone phosphate synthase | | -5.15 | 0.0015 |
| 224959_at | *SLC26A2* | | | solute carrier family 26 (sulfate transporter), member 2 | | -5.17 | 0.00274 |
| 219037_at | *RRP15* | | | ribosomal RNA processing 15 homolog (S. cerevisiae) | | -5.18 | 0.00471 |
| 222077_s_at | *RACGAP1* | | | Rac GTPase activating protein 1 | | -5.19 | 0.00282 |
| 225892_at | *IREB2* | | | iron-responsive element binding protein 2 | | -5.19 | 0.0165 |
| 217985_s_at | *BAZ1A* | | | bromodomain adjacent to zinc finger domain, 1A | | -5.2 | 0.00159 |
| 223618_at | *FMN2* | | | formin 2 | | -5.29 | 0.00333 |
| 212568_s_at | *DLAT* | | | dihydrolipoamide S-acetyltransferase | | -5.3 | 0.00049 |
| 229504_at |  | | |  | | -5.31 | 0.00221 |
| 208051_s_at | *PAIP1* | | | poly(A) binding protein interacting protein 1 | | -5.34 | 0.00671 |
| 222962_s_at | *MCM10* | | | minichromosome maintenance complex component 10 | | -5.35 | 0.00483 |
| 243916_x_at | *UBLCP1* | | | ubiquitin-like domain containing CTD phosphatase 1 | | -5.4 | 0.00232 |
| 218513_at | *TMA16* | | | translation machinery associated 16 homolog (S. cerevisiae) | | -5.4 | 0.00688 |
| 204026_s_at | *ZWINT* | | | ZW10 interacting kinetochore protein | | -5.41 | 0.00111 |
| 225847_at | *NCEH1* | | | neutral cholesterol ester hydrolase 1 | | -5.44 | 0.00661 |
| 223556_at | *HELLS* | | | helicase, lymphoid-specific | | -5.45 | 0.02306 |
| 203362_s_at | *MAD2L1* | | | MAD2 mitotic arrest deficient-like 1 (yeast) | | -5.49 | 0.01374 |
| 210261_at | *KCNK2* | | | potassium channel, subfamily K, member 2 | | -5.5 | 0.00068 |
| 204444_at | *KIF11* | | | kinesin family member 11 | | -5.5 | 0.01332 |
| 202069_s_at | *IDH3A* | | | isocitrate dehydrogenase 3 (NAD+) alpha | | -5.52 | 0.00073 |
| 222608_s_at | *ANLN* | | | anillin, actin binding protein | | -5.55 | 0.00546 |
| 228729_at | *CCNB1* | | | cyclin B1 | | -5.61 | 0.01007 |
| 230003_at | *SLC16A7* | | | solute carrier family 16, member 7 (monocarboxylic acid transporter 2) | | -5.63 | 0.00631 |
| 230559_x_at | *FGD4* | | | FYVE, RhoGEF and PH domain containing 4 | | -5.67 | 0.01099 |
| 226534_at | *KITLG* | | | KIT ligand | | -5.72 | 0.00042 |
| 203925_at | *GCLM* | | | glutamate-cysteine ligase, modifier subunit | | -5.77 | 0.00057 |
| 60794_f_at | *ZNF814* | | | zinc finger protein 814 | | -5.79 | 0.00362 |
| 237241_at | *ECT2* | | | epithelial cell transforming sequence 2 oncogene | | -5.81 | 0.00087 |
| 223403_s_at | *POLR1B* | | | polymerase (RNA) I polypeptide B, 128kDa | | -5.87 | 0.00731 |
| 218252_at | *CKAP2* | | | cytoskeleton associated protein 2 | | -5.9 | 0.00649 |
| 1553749_at | *FAM76B* | | | family with sequence similarity 76, member B | | -5.94 | 0.00239 |
| 219279_at | *DOCK10* | | | dedicator of cytokinesis 10 | | -5.95 | 0.00031 |
| 213293_s_at | *TRIM22* | | | tripartite motif containing 22 | | -5.96 | 0.00232 |
| 213704_at | *RABGGTB* | | | Rab geranylgeranyltransferase, beta subunit; small nucleolar RNA, C/D box 45A; small nucleolar RNA, C/D box 45B; small nucleolar RNA, C/D box 45C | | -6.14 | 0.00863 |
| 226600_at | *TMTC3* | | | transmembrane and tetratricopeptide repeat containing 3 | | -6.21 | 0.00393 |
| 238963_at | *RBM18* | | | RNA binding motif protein 18 | | -6.23 | 0.00267 |
| 235907_at | *TMEM33* | | | transmembrane protein 33 | | -6.24 | 0.00269 |
| 221561_at | *SOAT1* | | | sterol O-acyltransferase 1 | | -6.41 | 0.00518 |
| 219908_at | *DKK2* | | | dickkopf WNT signaling pathway inhibitor 2 | | -6.5 | 0.0045 |
| 221617_at | *TAF9B* | | | TAF9B RNA polymerase II, TATA box binding protein (TBP)-associated factor, 31kDa | | -6.52 | 0.00031 |
| 231784_s_at | *DCAF13* | | | DDB1 and CUL4 associated factor 13 | | -6.52 | 0.00333 |
| 222402_at | *POMP* | | | proteasome maturation protein | | -6.81 | 0.00731 |
| 201890_at | *RRM2* | | | ribonucleotide reductase M2 | | -7.01 | 0.00513 |
| 217632_at | *GNL3L* | | | guanine nucleotide binding protein-like 3 (nucleolar)-like | | -7.03 | 0.00414 |
| 204720_s_at | *DNAJC6* | | | DnaJ (Hsp40) homolog, subfamily C, member 6 | | -7.06 | 0.00585 |
| 221616_s_at | *TAF9B* | | | TAF9B RNA polymerase II, TATA box binding protein (TBP)-associated factor, 31kDa | | -7.11 | 0.00206 |
| 1565162_s_at | *MGST1* | | | microsomal glutathione S-transferase 1 | | -7.39 | 0.00534 |
| 1568574_x_at | *SPP1* | | | secreted phosphoprotein 1 | | -8.86 | 0.00043 |
